# The dichotomous behavior of allylsilanes in the additions to platinum α,β-unsaturated carbenes

**DOI:** 10.1039/d5sc03784k

**Published:** 2025-08-26

**Authors:** Jeff P. Costello, Jacob P. Garber, Khoi Q. Huynh, Eric M. Ferreira

**Affiliations:** a Department of Chemistry, University of Georgia Athens Georgia 30602 USA emferr@uga.edu

## Abstract

The observation of allylsilane dual reactivity with α,β-unsaturated platinum carbenes is described. The reaction pathways are controlled by the nature of the catalytic conditions. Under nonpolar conditions, a (3 + 2) cycloaddition is favored to provide decorated tricyclic indole and benzofuran products. Alternatively, the application of Lewis basic solvents enables the formation of C2-allylated indoles and benzofurans. An array of heterocyclic products are afforded in good yields, and the process illustrates the distinct and dichotomous reactivity of allylsilanes. The mechanistically divergent outcomes can be attributed to solvent effects on the respective intermediate stabilizations.

## Abstract 2

Divergently-reactive interceptions of α,β-unsaturated platinum carbenes with allylsilanes have been demonstrated. Catalytic conditions control the selective formation of either tricyclic heterocyclic products or C-2 allylated heterocyclic products.

## Graphical abstract

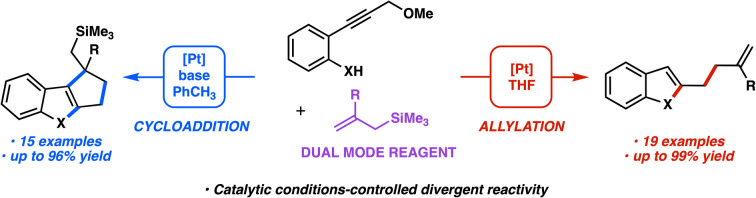

## Introduction

**Fig. 1:**
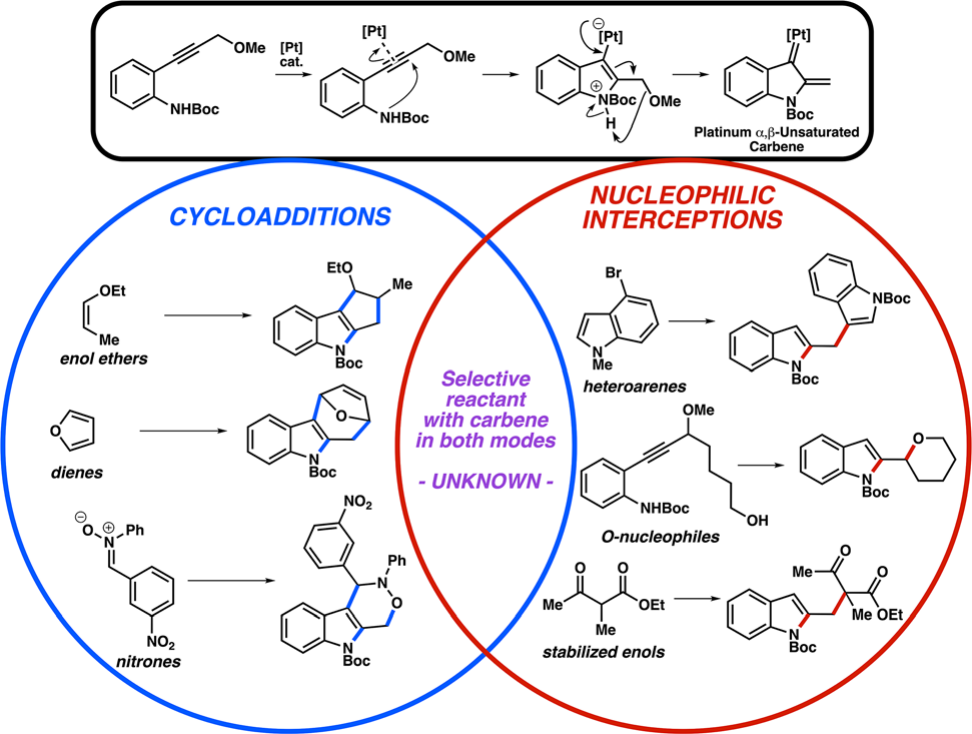
Summary of cycloaddition and nucleophilic interception reactivity of Pt α,β-unsaturated carbenes.

The development of catalytic complexity-building transformations is essential to efficient synthetic design. The capacity of a single catalyst to induce multiple bond-forming events can facilitate the rapid construction of molecules of interest.^1^ Due to the abundance of heterocycles in pharmaceutical agents and natural products, heterocyclizations coupled to additional processes can be advantageous toward the synthesis of medicinally relevant architectures.^2^ We and others have been investigating the formation of heterocycles *via* alkyne activation through platinum π-acid catalysis.^3^ These cyclization processes are coupled to additional events, specifically proceeding *via* the formation of α,β-unsaturated carbene intermediates (Fig. 1, top).^4–6^ The carbene intermediates have been shown to give diverging reactivity, depending primarily on the nature of the second reactant. Pi systems will generally undergo cycloadditions;^4^ examples comprise (2 + 3), (4 + 3), and (3 + 3) processes with enol ethers, dienes, and nitrones, respectively (Fig. 1, circled in blue). Electron-rich heterocycles, heteroatoms, or stabilized enols can instead add as nucleophiles at the β-position of the carbene (Fig. 1, circled in red).^5^

**Fig. 2:**
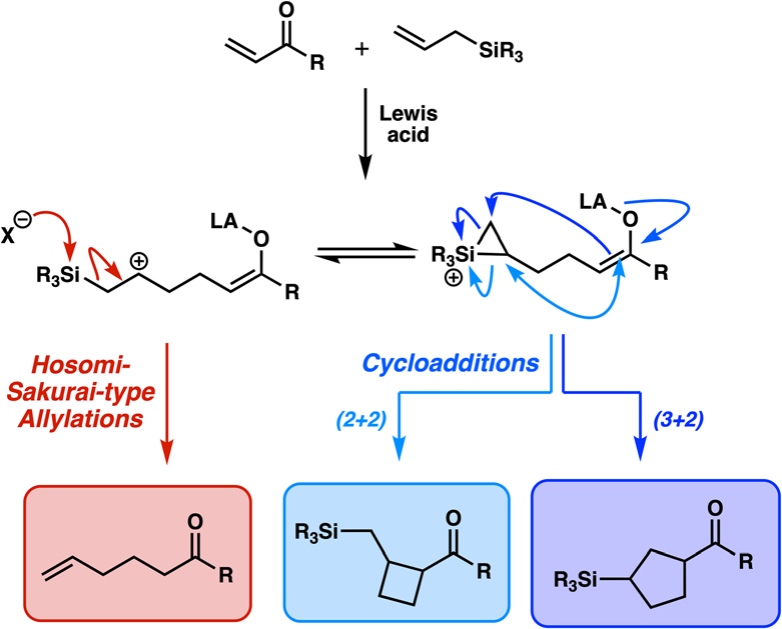
Allylsilane competency as allyl addition sources and cycloaddition partners.

We envisioned a system where these platinum carbenes can be trapped by a reactant that can exhibit both cycloaddition and nucleophilic behavior—*i.e.*, an intersection of the Venn diagram in Fig. 1. One possible reagent class that could potentially engage in this fashion is allylsilanes, as their capacity as dual reagents has been previously observed. Considering α,β-unsaturated carbonyl compounds as example electrophiles, it is well appreciated that allylsilanes will conjugately add as formal allyl nucleophiles (*i.e.*, Hosomi–Sakurai-type reactions, Fig. 2 (bottom left)).^7,8^ Reports in the 1990s by Knölker, Danheiser, and others demonstrated that allylsilanes could also behave as two- or three-atom cycloaddition partners with similar unsaturated carbonyl compounds (Fig. 2, bottom right).^9,10^ With reactants possessing more bulky aliphatic substituents on the silicon atom, it was observed that the cycloaddition would be the dominant process in this reaction. The divergence in reactivity was attributed to steric effects; increased alkyl substitution inhibits nucleophilic attack on the bridging siliranium intermediate,^11^ and thus steers the transformation toward intramolecular ring closure.^12^ Allylsilane dual reactivity has been observed more recently in multiple settings, such as in additions to alkylidene oxindoles,^13^ and alternative electrophiles such as homo-Nazarov cyclization intermediates^14^ and spiroepoxyoxindoles.^15^

**Fig. 3:**
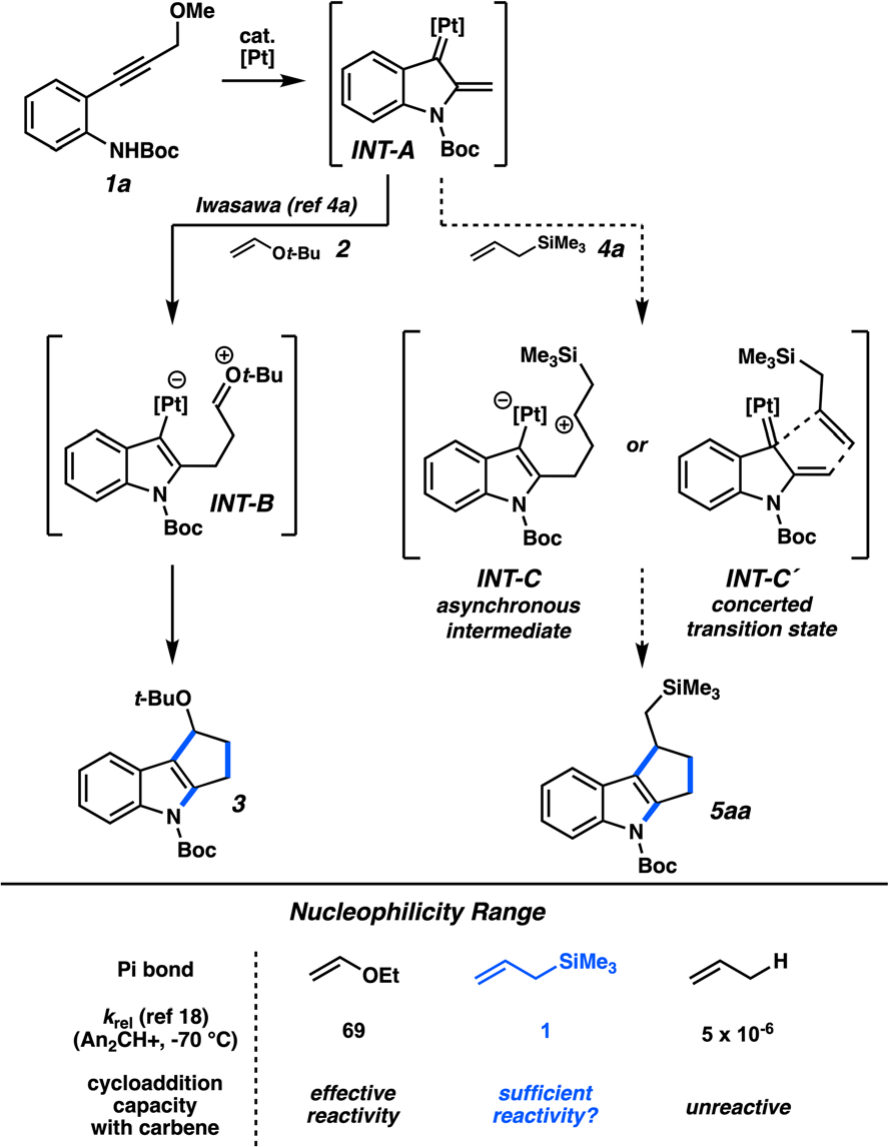
Comparison of enol ether mode of reactivity to proposed allylsilane reactivity.

The reactive platinum α,β-unsaturated carbene is an electrophile; the electron rich reaction partners in the prototypical cycloadditions and nucleophilic interceptions in Fig. 1 are indicative of the electrophilic nature of the carbene. In the seminal report from Iwasawa and coworkers,^4*a*^ they demonstrated that enol ethers were effective π-systems to participate in these carbene-based (3 + 2) cycloadditions (*e.g.*, 1a + 2 → 3), where the carbene unit is the three-atom component. With hopes of broadening the capacity of the (3 + 2) cycloaddition, we hypothesized that allylsilanes would be able to participate in similar reactions. The π-nucleophilicity of allylic silanes is well appreciated;^7^ it has been noted in quantification studies that replacing an allylic hydrogen with a trimethylsilyl group can increase the double bond nucleophilicity by a factor of 200 000.^16^ Further, there exists some ambiguity about the concertedness of the mechanism in these carbene cycloadditions across varying pi systems. Computational studies indicated that cycloadditions with enol ethers proceed in a stepwise fashion (*i.e.*, INT-B, Fig. 3).^17^ With respect to allylsilanes, a more asynchronous addition would presumably be enhanced by the hyperconjugative stabilization of the adjacent C–Si bond (*i.e.*, INT-C). Regardless, either this mechanism or a potential concerted mode of addition (INT-C′) would represent a reactivity profile consistent with the nucleophilicity of enol ethers (Fig. 3, below). The lesser nucleophilicity of allylic silanes,^18^ however, presents uncertainty whether they too would be competent reactants.

**Fig. 4:**
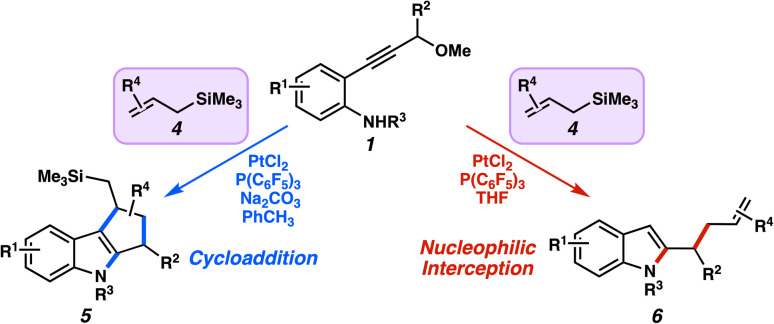
Divergent reactivity of allylsilanes in the trapping of the α,β-unsaturated platinum carbene.

Analogously, the allylsilane could also participate in nucleophilic additions to the α,β-unsaturated carbene—i.e., the Hosomi–Sakurai-type reactivity. The demonstration of π-bond nucleophiles in the Pt carbene manifold by us and others offers credence to the use of allylsilanes in this context. Taken together, it is conceivable that allylsilanes could display dual reactivity with the platinum α,β-unsaturated carbenes. In this report, we describe the realization of this concept, where heterocyclizations are followed by cycloadditions or nucleophilic interceptions depending chiefly on the catalytic conditions (Fig. 4).

## Results and discussion

Our initial analysis of allylsilane reactivity in the α,β-unsaturated carbene manifold is presented in Table 1. Indeed, we found that the combination of alkyne 1a and allyl trimethylsilane (4a) using catalytic Zeise's Dimer ([(C_2_H_4_)PtCl_2_]_2_, 2.5 mol%) afforded cycloaddduct 5aa in 49% yield (entry 1). We also observed small quantities of compound 7aa, reflective of an ipso substitution of the alkene.^19^ Various parameters were evaluated to maximize the formation of the cycloadduct in selective fashion. Solvent variation was unsuccessful toward this goal (entries 2–5). Lewis acidic additives, which had been fruitful in improving dicarbonyl nucleophile addition, were ineffective here (entries 6, 7). Varying the catalyst led to mixed results, where PtCl_2_ plus alkene/diene ligands^20^ modestly altered but did not improve the reaction. Attempts to increase the reactivity of the Pt catalyst with an addition of a silver salt^21^ halted the reaction altogether (entry 13). We found a notable boost when turning to an electron-deficient phosphine, however. The yield of cycloadduct 5aa improved to 64% when using PtCl_2_ with P(C_6_F_5_)_3_ (refs. 22 and 23) as an added ligand (entry 16), perhaps increasing the electrophilicity and thus reactivity of the carbene intermediate. Added Na_2_CO_3_ also increased the overall yield, and with raising the reaction temperature to 110 °C, (3 + 2) cycloadduct 5aa was obtained in 93% yield (entry 24), with minimal formation of the ipso product. A 2 : 1 ratio of phosphine to Pt was optimal, although altering the ratio was only modestly consequential (entries 25, 26). Less than 5 equiv. of the allylsilane reactant could be employed in this cycloaddition, although the yields were somewhat compromised (entries 27, 28).

**Table 1:** Optimization of (3 + 2) cycloaddition conditions

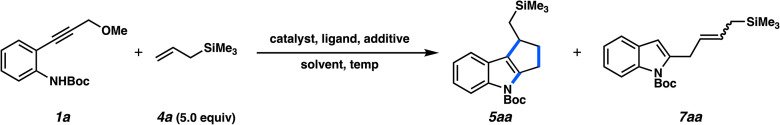							
Entry	Catalyst (5 mol% Pt)	Ligand (mol%)	Additive (equiv.)	Solvent, temp (°C)	*t* (h)	Yield^a^5aa (%)	Yield^a^7aa (%)
1	[(C_2_H_4_)PtCl_2_]_2_	—	—	PhCH_3_, 80	3	49	18
2	[(C_2_H_4_)PtCl_2_]_2_	—	—	Benzene, 80	3	28	9
3	[(C_2_H_4_)PtCl_2_]_2_	—	—	DCE, 80	19	14	7
4	[(C_2_H_4_)PtCl_2_]_2_	—	—	CH_3_CN, 80	19	0	0
5	[(C_2_H_4_)PtCl_2_]_2_	—	—	MeOH, 80	19	0	0^b^
6	[(C_2_H_4_)PtCl_2_]_2_	—	MgCl_2_ (1.0)	PhCH_3_, 80	2	41	18
7	[(C_2_H_4_)PtCl_2_]_2_	—	Cu(OTf)_2_ (1.0)	PhCH_3_, 80	3	20	10
8	PtCl_2_	1-Octene (100)	—	PhCH_3_, 100	3	36	9
9	PtCl_2_	Methyl acrylate (100)	—	PhCH_3_, 100	3	38	11
10	PtCl_2_	Styrene (100)	—	PhCH_3_, 100	3	46	11
11	PtCl_2_	Norbornadiene (100)	—	PhCH_3_, 100	48	25	8
12	PtCl_2_	COD (100)	—	PhCH_3_, 100	48	10	3
13	PtCl_2_	Styrene (100)	AgBF_4_ (0.1)	PhCH_3_, 100	20	0	0
14	PtCl_2_	PPh_3_ (10)	—	PhCH_3_, 80	22	11	<5
15	PtCl_2_	P(OPh)_3_ (10)	—	PhCH_3_, 80	22	24	11
16	PtCl_2_	P(C_6_F_5_)_3_ (10)	—	PhCH_3_, 80	2	64	2
17	[(C_2_H_4_)PtCl_2_]_2_	P(C_6_F_5_)_3_ (10)	—	PhCH_3_, 80	18	52	1
18	PtCl_2_	P(C_6_F_5_)_3_ (10)	Li_2_CO_3_ (1.5)	PhCH_3_, 80	2	62	<5
19	PtCl_2_	P(C_6_F_5_)_3_ (10)	Na_2_CO_3_ (1.5)	PhCH_3_, 80	1	87	<5
20	PtCl_2_	P(C_6_F_5_)_3_ (10)	K_2_CO_3_ (1.5)	PhCH_3_, 80	20	50	<5
21	PtCl_2_	P(C_6_F_5_)_3_ (10)	Cs_2_CO_3_ (1.5)	PhCH_3_, 80	20	14	<5
22	PtCl_2_	P(C_6_F_5_)_3_ (10)	NaHCO_3_ (1.5)	PhCH_3_, 80	2	56	<5
23	PtCl_2_	P(C_6_F_5_)_3_ (10)	Na_2_CO_3_ (1.5)	PhCH_3_, 80	5	81	0
24	PtCl_2_	P(C_6_F_5_)_3_ (10)	Na_2_CO_3_ (1.5)	PhCH_3_, 110	1	93	<5
25	PtCl_2_	P(C_6_F_5_)_3_ (5)	Na_2_CO_3_ (1.5)	PhCH_3_, 110	1	80	<5
26	PtCl_2_	P(C_6_F_5_)_3_ (15)	Na_2_CO_3_ (1.5)	PhCH_3_, 110	4	91	<5
27^c^	PtCl_2_	P(C_6_F_5_)_3_ (10)	Na_2_CO_3_ (1.5)	PhCH_3_, 110	1	69	<5
28^d^	PtCl_2_	P(C_6_F_5_)_3_ (10)	Na_2_CO_3_ (1.5)	PhCH_3_, 110	1	75	<5

a NMR yield based on vanillin as an internal standard.

b Indole 8 observed as sole product.

c 1.2 equiv 4a used.

d 2.5 equiv 4a used.
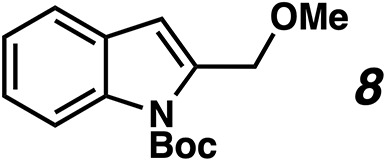

**Fig. 5:**
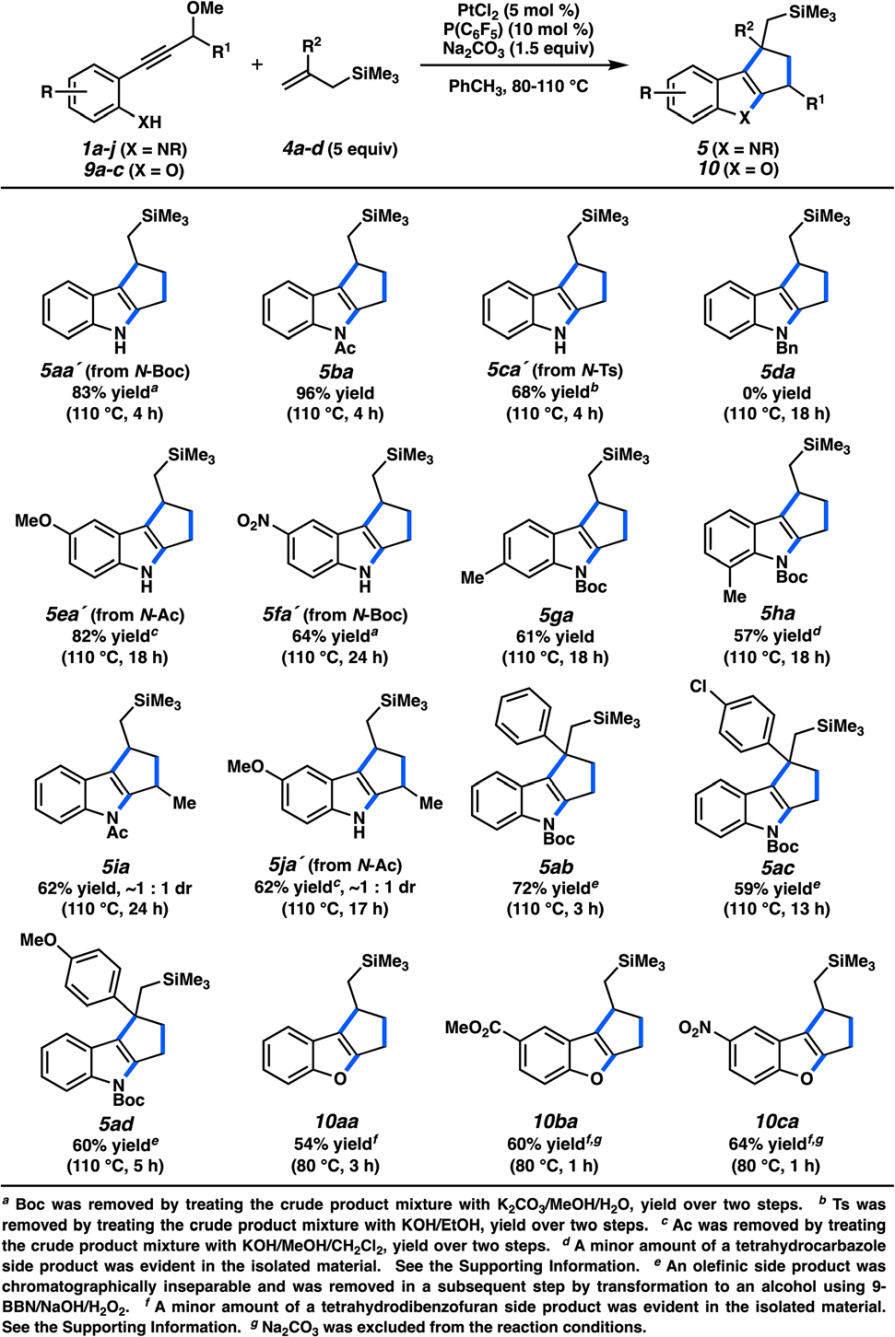
Scope of (3 + 2) cycloaddition with allylsilane partners.

The scope of this cycloaddition is illustrated in Fig. 5. Differentially-protected nitrogen atoms were competent heterocyclization nucleophiles, as long as the protecting group tempered the basicity of the nitrogen.^24^*N*-alkyl anilines were not compatible (*e.g.*, 5da), presumably due to competitive coordination with the Pt catalyst. Functionalized arenes with multiple substitution patterns were tolerated (cycloadducts 5ea–5ha, 5ja). The β-carbon of the unsaturated carbene could be substituted, although essentially no diastereoselectivity was observed in the addition (5ia, 5ja). More substituted allyl groups were accommodated; for example, 2-aryl-substituted allylsilanes were competent cycloaddition partners (5ab–5ad). Finally, annulated benzofurans could also be synthesized *via* this method (10aa–10ca). Consistent with our hypothesis, the silyl moiety was critical, as standard alkenes (*i.e.*, non-silylmethylene substituted) were generally ineffective.^25^

**Fig. 6:**
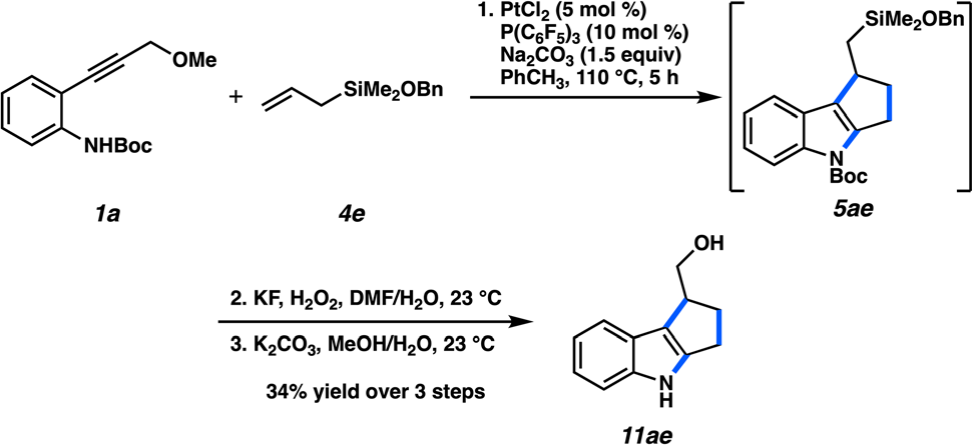
Diversification of cycloadduct to primary alcohol.

Because of the reliance of this (3 + 2) cycloaddition on the silicon group, it would be advantageous for the overall process if the silicon-bearing cycloadduct could be converted to a more common functionality after the cycloaddition. An alkyl–SiMe_3_ group cannot be trivially transformed, however, and methods typically require strongly electrophilic reagents that would be incompatible with electron-rich heteroarenes.^26,27^ To that end, we tested allyldimethylsiloxane 4e (Fig. 6). Gratifyingly, the cycloaddition was productive and indole 5ae was formed, which could be directly subjected to a successful Tamao–Fleming oxidation. After Boc deprotection, the resulting alcohol (11ae) was afforded in a 34% yield over three steps. Although the three-step yield is modest, this technique allows for the conversion to a useful synthetic handle.^28^

**Fig. 7:**
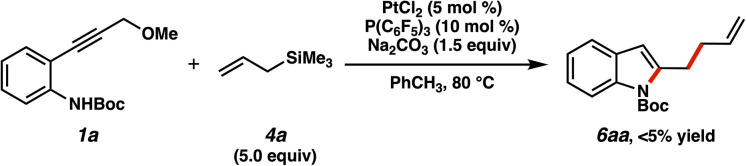
Observation of direct allyl addition product.

With an effective method for cycloaddition in hand, we turned our attention to allylation. As discussed in the Introduction, in the α,β-unsaturated carbene manifold we and others had observed that nucleophiles (*e.g.*, indoles, β-dicarbonyls) could attack at the β-position of the α,β-unsaturated carbene intermediate.^5^ With respect to allylation, we observed a promising lead in our initial scan of these reactions using allylic silane 4a, as represented in Table 1. In a couple of cases, we had detected small quantities (<5%) of an allylated product (6aa, Fig. 7), corroborating the notion that allylation was conceivable. Prompted by this finding, we further evaluated these reactions to see if we could steer the reaction preference heavily toward allylation. It was anticipated that systems which facilitated desilylation (*i.e.*, more ionic/polarized conditions) would shift the transformation in this allylative direction.

Relative to the solvents screened in Table 1, comparatively more polar media were effective toward forming the product of allyl addition (indole 6aa), as shown in Table 2.^29^ Although DMF gave exclusive formation of indole 6aa (entry 5), the yield was low and THF appeared to be a better choice for further optimization (entry 1). A slight improvement was found when switching from Zeise's dimer to PtCl_2_ (entry 7), avoiding the formation of substitution byproduct 7aa. Most ligands (alkenes, PPh_3_, P(OPh)_3_, CO) were either ineffectual or detrimental to reactivity (entries 8–12), but again the use of P(C_6_F_5_)_3_ provided a marked improvement in the formation of indole 6aa (entry 13). Raising the temperature appeared to compromise selectivity in this optimization study (entry 14).^30^ Added base or fluoride source^31^ arrested the reaction altogether (entries 15–17). A higher catalyst loading (10 mol% Pt, 20 mol% P) only marginally improved the yield (entry 18). Ultimately, we determined that the PtCl_2_/P(C_6_F_5_)_3_ catalyst system in THF with no additives was preferred for forming compound 6aa with the optimal combination of yield and product selectivity (entries 13–14). Similar to the cycloaddition conditions, the 2 : 1 ratio of phosphine to Pt provided the best performance (entries 14–16). Using fewer equivalents of allylsilane was tolerated, albeit with more prominent formation of side product 5aa (entry 21).

**Table 2:** Optimization of allylation conditions

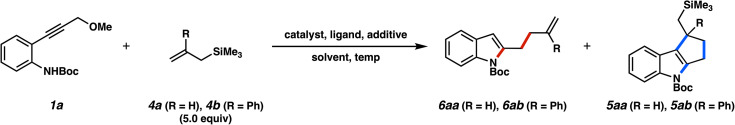								
Entry	Catalyst (5 mol%)	R	Ligand (mol%)	Additive (1.0 equiv.)	Solvent, temp (°C)	*t* (h)	Yield^a^6 (%)	Yield^a^5 (%)
1^b^	[(C_2_H_4_)PtCl_2_]_2_	H	—	—	THF, 23	3	43	16
2^b^	[(C_2_H_4_)PtCl_2_]_2_	H	—	—	1,4-Dioxane, 23	5	14	18
3^b^	[(C_2_H_4_)PtCl_2_]_2_	H	—	—	MTBE, 23	3	14	22
4^b^	[(C_2_H_4_)PtCl_2_]_2_	H	—	—	EtOAc, 40	5	22	15
5	[(C_2_H_4_)PtCl_2_]_2_	H	—	—	DMF, 100	24	10	0
6^b^	[(C_2_H_4_)PtCl_2_]_2_	H	—	Na_2_CO_3_	THF, 23	36	35	13
7	PtCl_2_	H	—	—	THF, 23	6	47	19
8	PtCl_2_	H	Methyl acrylate (100)	—	THF, 23	20	50	18
9	PtCl_2_	H	ArCH=CH_2_ (100) (Ar: 3,5-(F_3_C)_2_C_6_H_3_)	—	THF, 23	19	50	20
10	PtCl_2_	H	CO (1 atm)	—	THF, 23	21	0	0
11	PtCl_2_	H	PPh_3_ (10)	—	THF, 50	24	0	0
12	PtCl_2_	H	P(OPh)_3_ (10)	—	THF, 23	18	48	13
13	PtCl_2_	H	P(C_6_F_5_)_3_ (10)	—	THF, 23	21	69	15
14	PtCl_2_	H	P(C_6_F_5_)_3_ (10)	—	THF, 60	18	50	21
15	PtCl_2_	H	P(C_6_F_5_)_3_ (5)	—	THF, 60	18	44	24
16	PtCl_2_	H	P(C_6_F_5_)_3_ (15)	—	THF, 60	18	53	17
17	PtCl_2_	H	P(C_6_F_5_)_3_ (10)	Na_2_CO_3_	THF, 50	11	0	0
18	PtCl_2_	H	P(C_6_F_5_)_3_ (10)	TBAF	THF, 23	24	0	0
19	PtCl_2_	H	P(C_6_F_5_)_3_ (10)	CsF	THF, 50	24	0	0
20	PtCl_2_^c^	H	P(C_6_F_5_)_3_ (20)	—	THF, 23	20	77	15
21^d^	PtCl_2_	H	P(C_6_F_5_)_3_ (10)	—	THF, 23	21	64	29
22	PtCl_2_	Ph	P(C_6_F_5_)_3_ (10)	—	THF, 23	19	95	0

a NMR yield based on vanillin as an internal standard.

b Ipso substitution product 7aa was observed in <15% yield.

c 10 mol% Pt.

d 1.2 equiv. 4a used.

**Fig. 8:**
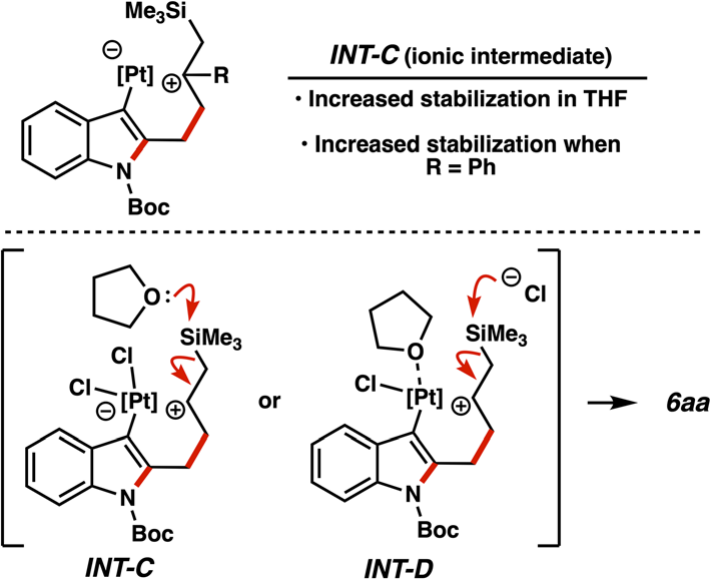
Proposed solvent effects on reactivity.

We believe the impact of this switch in catalytic conditions on reactivity is primarily attributable to the ethereal solvent facilitating desilylation of INT-C/INT-C′ (Fig. 3, *vide supra*). THF should be improving stabilization of polarized intermediates relative to toluene, thus allowing the transformation to be more asynchronous in nature and increasing the prominence of INT-C (Fig. 8). Subsequent desilylation *via* donation into the silicon atom could then occur, either directly by THF or indirectly by chloride dissociation from the platinum intermediate *via*INT-D.^32^ Solvent effects on analogous desilylations have been observed.^15*b*,33^ Direct solvent-mediated desilylation has been invoked previously in allylsilane additions to highly electrophilic alkenes,^34^ and computational studies on palladium-mediated desilylation of allylic silanes have corroborated the combined enhancing effects of polar solvents and halide ions.^35^ It is worth noting that when 1-phenylallyltrimethylsilane was employed in the THF-based conditions, the yield and preference for the allylation product markedly increased (Table 2, entry 20), giving further evidence to the prevalence and stabilization of cationic species. Solvent effects were previously observed by us in this carbene manifold;^36^ the differences between a silicon and hydrogen shift were dictated almost entirely by the nature of the solvent, where the latter process could also be attributed to the intermediacy of polarized intermediates.

**Fig. 9:**
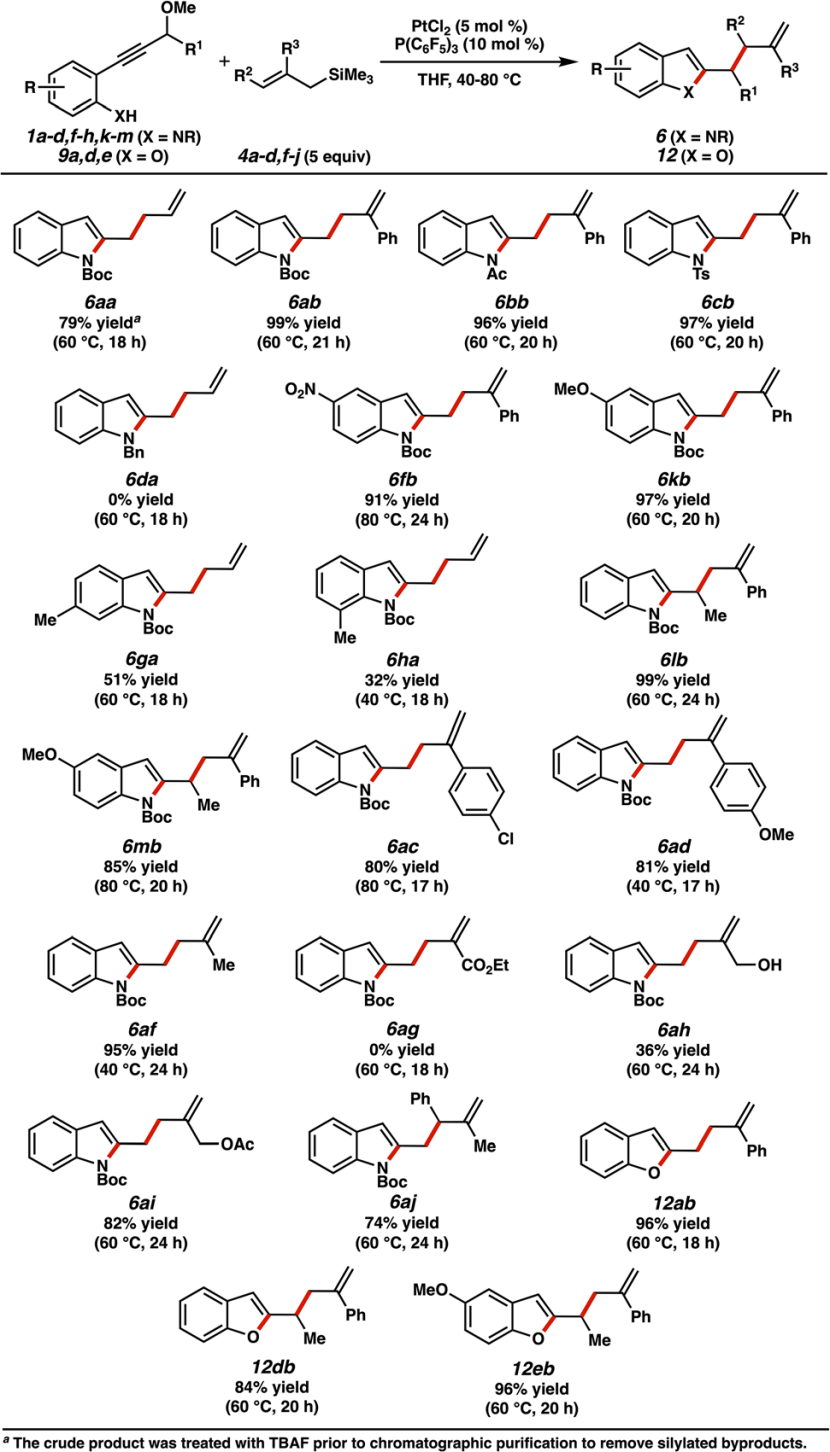
Scope of allylation with allylsilane nucleophiles.

The scope of allylation products is shown in Fig. 9. Similar to the cycloaddition process, variations of the alkyne precursor were highly tolerated. Differentially protected indoles were afforded in good yields (6bb, 6cb), unless the aniline nitrogen was too basic (*e.g.*, 6da). Arene substitutions were again accommodated (6fb, 6kb, 6ga, 6ha), although 7-Me indole product 6ha was afforded in diminished yield.^37^ Substituents at the β-position of the carbene (6lb, 6mb) did not deter reactivity. Several different allylic silanes were effective allylating agents. The lack of formation of product 6ag reflects the diminished nucleophilicity of the ester-substituted allylsilane species. Additionally, alcohol 6ah was afforded in more modest yield, presumably due to competitive hydroxyl interception of potential cationic intermediates of the allylation mechanism.^38^ The high yield of acetate analog 6ai would be consistent with this hypothesis. Indole 6aj is notable, demonstrating that a terminally substituted allylsilane can undergo nucleophilic addition. As before, allylated benzofurans could also be successfully synthesized *via* this method (12ab, 12db, 12eb).

**Fig. 10:**
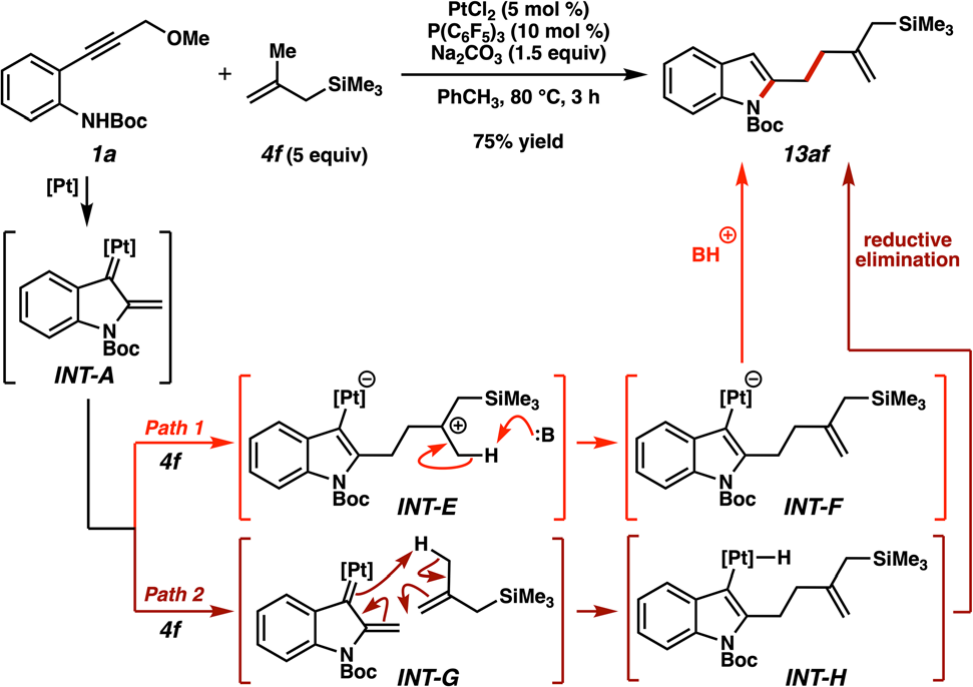
Ene-type reactivity observed with trimethyl(2-methylallyl)silane under cycloaddition conditions.

It should be noted that not all of the allylsilanes examined were able to participate in both direct allylation and (3 + 2) cycloadditions effectively. Namely, trimethyl(2-methylallyl)silane was effective in the allylation (*i.e.*, 6af, Fig. 9) but not in the cycloaddition. Instead, an ene-type reaction product was observed under the cycloaddition conditions.^39^ Newly-substituted allylsilane 13af was produced when alkyne 1a and allylsilane 4f were treated under the Na_2_CO_3_/PhCH_3_ conditions (Fig. 10). Presumably, these basic conditions instead induce a deprotonation from cationic intermediate INT-E at high temperatures (Path 1), followed by protodemetalation. An alternative ene-like mechanism followed by reductive elimination is also conceivable (Path 2), although the methyl substituent stabilization of intermediate INT-E plus the feasibility of a deprotonative pathway encourages us to favor Pathway A.

**Fig. 11:**
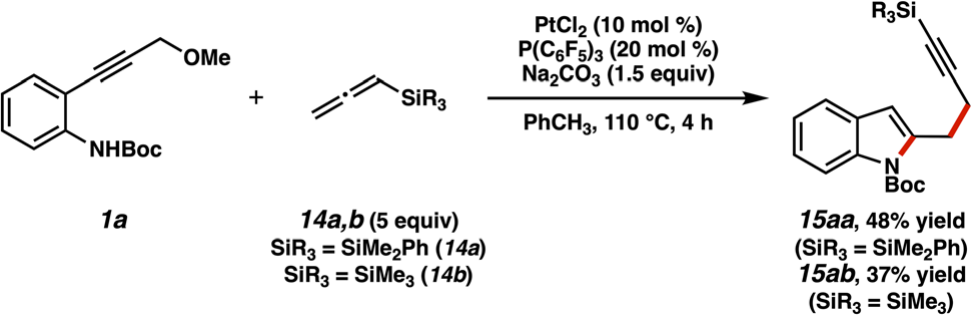
Utilization of allenylsilanes in trapping of the α,β-unsaturated platinum carbene.

We were also able to show that other silicon-bearing nucleophiles could participate in this reaction (Fig. 11). When allenylsilanes, which have been utilized in previous Lewis acid and π-acid chemistry,^40^ were applied in this setting, propargylic indole products were formed. The yields were moderate, however, and required higher loadings of the catalyst and ligand.^41^ The diminished reactivity can be rationalized by product inhibition, where the silylalkyne product may competitively coordinate the platinum center and thus suppresses productive catalysis. The increased relative formation of PhMe_2_Si-substituted product (15aa) is consistent with this rationale.^42^

**Fig. 12:**
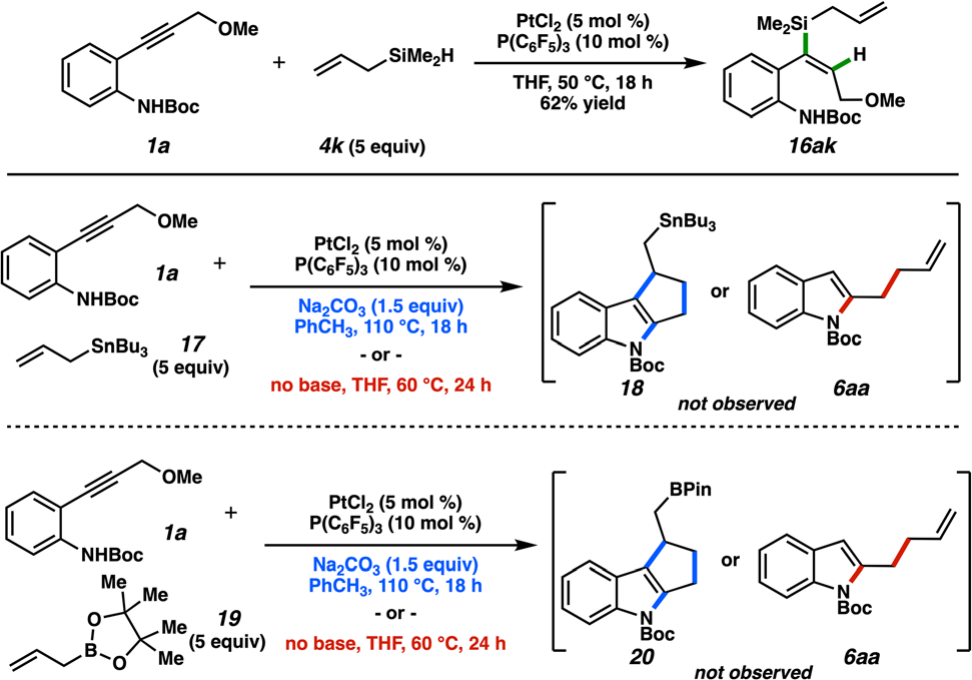
Hydrosilylation product and unsuccessful allylation alternatives.

Fig. 12 depicts additional interesting observations related to this reactivity system. Allylsilane 4f could not be used in this transformation; platinum-catalyzed alkyne hydrosilylation^43^ was the only process observed.^44^ Allylic stannane and borane reagents can demonstrate similar reactivity profiles as allylic silanes with electrophilic species (*e.g.*, allyl addition to aldehydes).^45^ Tributylstannane 17 and pinacolborane 19, however, were unproductive in allylation or cycloaddition, again underscoring the unique behavior of allylic silanes in this platinum manifold.

## Conclusions

In summary, we have demonstrated the divergent reactivity of allylsilanes in the catalytic α,β-unsaturated platinum carbene manifold. Following the generation of the reactive intermediate, we are able to control the mode of addition of the allylic silane by selection of conditions. In a nonpolar solvent, a cycloaddition process is favored that constructs tricyclic indole and benzofuran products. Alternatively, when the solvent employed can stabilize more polarized intermediates, desilylation becomes favored and C-2 allylated indole and benzofuran products are accessed as the major outputs of the transformation. Current efforts are directed toward exploring this dual reactivity with alternative reactants in conjunction with the carbene intermediate.

## Author contributions

Conceptualization: E. M. F. Investigation: J. P. C., J. P. G., and K. Q. H. Methodology: J. P. C., J. P. G., and K. Q. H. Funding acquisition: E. M. F. Project administration: E. M. F. Supervision: E. M. F. Writing – original draft: K. Q. H. Writing – review & editing: J. P. C., J. P. G., and E. M. F.

## Conflicts of interest

There are no conflicts to declare.

## Supplementary Material

SC-OLF-D5SC03784K-s001

## Data Availability

Supplementary Information: Data for this article, including experimental procedures and spectroscopic data, are available in the provided SI See DOI: https://doi.org/10.1039/d5sc03784k.
