# Supplementary material for: The dichotomous behavior of allylsilanes in the additions to platinum α,β-unsaturated carbenes
Source: Chem Sci. 2025 Aug 26;16(38):17744–52. doi: 10.1039/d5sc03784k (PMC12406950; doi:10.1039/d5sc03784k)
Supplement: SC-016-D5SC03784K-s001 [file SC-016-D5SC03784K-s001.pdf]

Supplementary Information (SI) for Chemical Science.
This journal is © The Royal Society of Chemistry 2025

# Supporting Information

**The Dichotomous Behavior of Allylsilanes in the Additions to Platinum α,β-Unsaturated Carbenes**

Jeff P. Costello, Jacob P. Garber, Khoi Q. Huynh, and Eric M. Ferreira*

*Department of Chemistry, University of Georgia, Athens, GA 30602, United States*

emferr@uga.edu

# Table of Contents

**Materials and Methods** ........ S3
**General Experimental Procedures for Cycloaddition and Allylation** ........ S4
**Platinum-Catalyzed (3+2) Cycloaddition Scope** ........ S5
**Platinum-Catalyzed Allylation Scope** ........ S16
**Miscellaneous Experiments** ........ S28
**Reaction Optimization** ........ S35
**Preparation of Aniline and Phenol Starting Materials** ........ S42
**Preparation of Allylic Silane Starting Materials** ........ S50
**Additional Starting Material Syntheses** ........ S56
**References** ........ S58
**$^{1}$H and $^{13}$C NMR Spectra Compilation** ........ S59

# Materials and Methods

Reactions were performed under an argon atmosphere unless otherwise noted. Tetrahydrofuran, diethyl ether, dichloromethane and toluene were purified by passing through activated alumina columns. 1,4-Dioxane was distilled over sodium/benzophenone. Triethylamine was distilled over $CaH_2$. All other solvents used were ACS grade and used as received, and all other reagents were used as received unless otherwise noted. Zeise's dimer ($[(C_2H_4)PtCl_2]_2$) was purchased from Strem Chemical Company. All other commercially available chemicals were purchased from Alfa Aesar (Ward Hill, MA), Sigma-Aldrich (St. Louis, MO), Oakwood Products, (West Columbia, SC), or TCI America (Portland, OR). Qualitative TLC analysis was performed on 250 mm thick, 60 Å, glass backed, F254 silica (Silicycle, Quebec City, Canada). Visualization was accomplished with UV light and exposure to $KMnO_4$ stain solution followed by heating. Flash chromatography was performed using Silicycle silica gel (230-400 mesh). $^1$H NMR spectra were acquired on either a Varian 400 Mercury Plus (at 400 MHz), a Bruker AVANCE III HD NMR (at 400 MHz), or a Varian INOVA (at 500 MHz), or an Agilent DD2 NMR (at 600 MHz), and are reported relative to $SiMe_4$ (δ 0.00). $^{13}$C NMR spectra were acquired on a Varian 400 Mercury Plus (at 100 MHz), a Bruker AVANCE III HD NMR (at 100 MHz), or Varian INOVA (at 125 MHz) and are reported relative to $SiMe_4$ (δ 0.00). All IR spectra were obtained as thin films with a Nicolet iS-100 FT-IR and are reported in wavenumbers (ν). High resolution mass spectrometry (HRMS) data were acquired via electrospray ionization (ESI) using either a ThermoFisher Orbitrap Q-Exactive or a ThermoFisher Orbitrap Elite, the latter at the Proteomics and Mass Spectrometry Facility at the University of Georgia. Analytical GC data were acquired on an Agilent Technologies 7820A using a DB-5 column (30 m x 0.320 mm) from Agilent Technologies.

# General Experimental Procedures for Cycloaddition and Allylation

## General Procedure A: Pt-Catalyzed (3+2) Cycloadditions

To a solution of $P(C_6F_5)_3$ (10 mol %) in toluene (1/2 of total reaction volume) at 23 °C was added $PtCl_2$ (5 mol %), and the resulting solution was stirred for 15 min. This catalyst solution was then added to a prestirred solution of substrate (1.0 equiv) and allylic trimethylsilane (5.0 equiv) in toluene (1/2 of total reaction volume, 0.1 M in substrate final concentration) at 23 °C. $Na_2CO_3$ (1.5 equiv) was then added, and the resulting mixture was stirred at 80 - 110 °C and monitored by TLC. Upon completion, the reaction mixture was cooled to room temperature and filtered through $SiO_2$ plug, eluting with 1:1 EtOAc/hexanes (approx. 3x reaction volume). The solvent was then removed by rotary evaporation, and the resulting residue was purified by flash chromatography on $SiO_2$.

## General Procedure B: Pt-Catalyzed (3+2) Cycloadditions (without $Na_2CO_3$ added)

To a solution of $P(C_6F_5)_3$ (10 mol %) in toluene (1/2 of total reaction volume) at 23 °C was added $PtCl_2$ (5 mol %), and the resulting solution was stirred for 15 min. A prestirred solution of allylic trimethylsilane (5.0 equiv) in toluene (1/2 of total reaction volume, 0.1 M in substrate final concentration) was added to the catalyst solution at 23 °C. The substrate (1.0 equiv) was then added, and the resulting mixture was stirred at 80 °C and monitored by TLC. Upon completion, the solution was cooled to room temperature and filtered through $SiO_2$ plug, eluting with 1:1 EtOAc/hexanes (approx. 3x reaction volume). The solvent was then removed by rotary evaporation, and the resulting residue was purified by flash chromatography on $SiO_2$.

## General Procedure C: Pt-Catalyzed Allylations

To a solution of $P(C_6F_5)_3$ (10 mol %) in THF (1/2 of total reaction volume) at 23 °C was added $PtCl_2$ (5 mol %), and the resulting solution was stirred for 15 min. This catalyst solution was then added to a prestirred solution of the substrate (1.0 equiv) and allylic trimethylsilane (5.0 equiv) in THF (1/2 of total reaction volume, 0.1 M in substrate final concentration) at 23 °C. The resulting mixture was stirred at 60 - 80 °C and monitored by TLC. Upon completion, the reaction mixture was cooled to room temperature and filtered through a $SiO_2$ plug, eluting with 1:1 EtOAc/hexanes (approx. 3x reaction volume). The solvent was then removed by rotary evaporation, and the resulting residue was purified by flash chromatography on $SiO_2$.

# Platinum-Catalyzed (3+2) Cycloaddition Scope

### *Figure 5 (reproduced).*

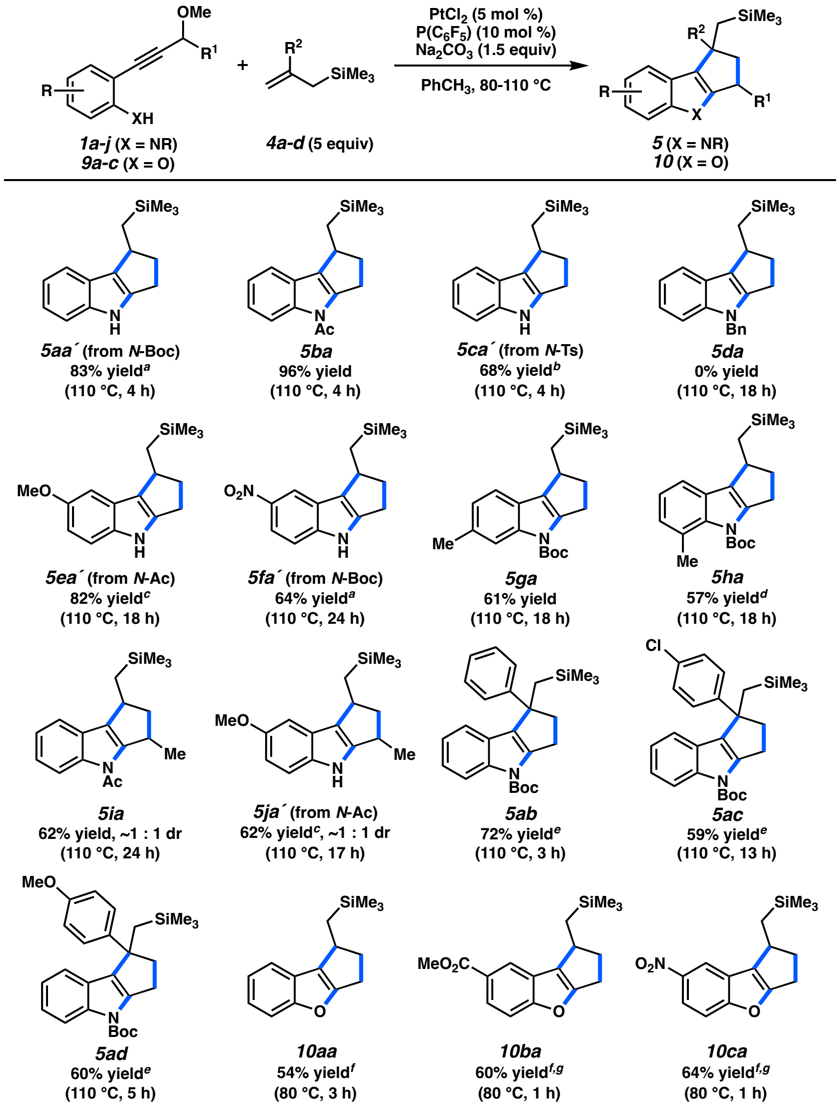


[a] Boc was removed by treating the crude product mixture with $K_2CO_3$/MeOH/$H_2O$, yield over two steps. [b] Ts was removed by treating the crude product mixture with KOH/EtOH, yield over two steps. [c] Ac was removed by treating the crude product mixture with KOH/MeOH/$CH_2Cl_2$, yield over two steps. [d] A minor amount of a tetrahydrocarbazole side product was evident in the isolated material. See the Supporting Information. [e] An olefinic side product was chromatographically inseparable and was removed in a subsequent step by transformation to an alcohol using 9-BBN/NaOH/$H_2O_2$. [f] A minor amount of a tetrahydrodibenzofuran side product was evident in the isolated material. See the Supporting Information. [g] $Na_2CO_3$ was excluded from the reaction conditions.

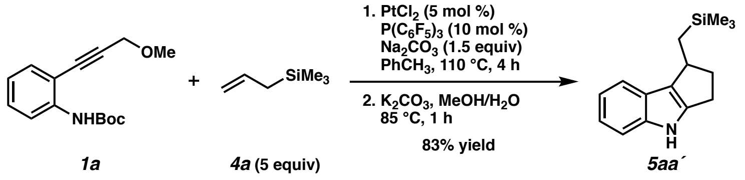

**Indole 5aa′.** To a solution of $P(C_6F_5)_3$ (10.0 mg, 0.0188 mmol) in toluene (0.95 mL) at 23 °C was added $PtCl_2$ (2.5 mg, 0.00940 mmol), and the resulting solution was stirred for 15 min. This catalyst solution was then added to a prestirred solution of alkyne **1a** (49.2 mg, 0.188 mmol) and allyltrimethylsilane (**4a**, 149 µL, 0.940 mmol) in toluene (0.95 mL, 0.1 M in substrate final concentration) at 23 °C. $Na_2CO_3$ (29.9 mg, 0.282 mmol) was then added, and the resulting mixture was stirred in a 110 °C heating block for 4 h. Upon completion, the reaction mixture was cooled to room temperature and filtered through a $SiO_2$ plug (0.5 x 3 cm), eluting with 1:1 EtOAc/hexanes (5 mL). The solvent was removed by rotary evaporation, and the resulting residue was dissolved in a 3:1 $MeOH/H_2O$ mixture (2.50 mL). $K_2CO_3$ (52.0 mg, 0.376 mmol) was added, and the resulting mixture was stirred in an 85 °C heating block for 1 h. Upon reaction completion, the solution was allowed to cool to room temperature, and MeOH was removed by rotary evaporation. EtOAc (5 mL) was added, and the phases were separated. The aqueous phase was extracted with EtOAc (2 x 5 mL), and the combined organic phases were wash with brine (5 mL) and dried over $MgSO_4$. The mixture was concentrated in vacuo, and the resulting residue was purified by flash chromatography (3:1 hexanes/EtOAc w/ 2% $Et_3N$ eluent) to afford indole **5aa′** (38.0 mg, 83% yield, $R_f$ = 0.39 in 3:1 hexanes/EtOAc w/ 2% $Et_3N$) as a colorless oil. The spectroscopic data for indole **5aa′** were in agreement with those collected in the synthesis of indole **5ca′** (see below). Decomposition of NMR samples was best prevented by briefly passing argon over the sample solution.

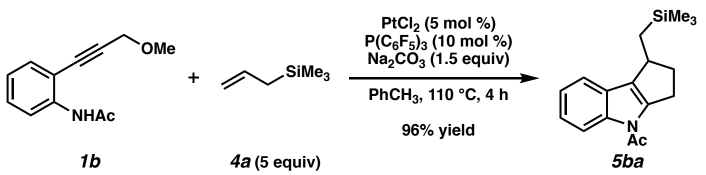


**Indole 5ba.** To a solution of $P(C_6F_5)_3$ (13.3 mg, 0.0249 mmol) in toluene (1.25 mL) at 23 °C was added $PtCl_2$ (3.3 mg, 0.0125 mmol), and the resulting solution was stirred for 15 min. This catalyst solution was then added to a prestirred solution of alkyne **1b** (50.7 mg, 0.249 mmol) and allyltrimethylsilane (**4a**, 199 µL, 1.25 mmol) in toluene (1.25 mL, 0.1 M in substrate final concentration) at 23 °C. $Na_2CO_3$ (39.6 mg, 0.374 mmol) was then added, and the resulting mixture was stirred in a 110 °C heating block for 4 h. Upon reaction completion, the reaction mixture was cooled to room temperature and filtered through a $SiO_2$ plug (0.5 x 3 cm), eluting with 1:1 EtOAc/hexanes (5 mL). The solvent was removed by rotary evaporation, and the resulting residue was purified by flash chromatography (9:1 hexanes/EtOAc eluent) to afford indole **5ba** (68.5 mg, 96% yield, $R_f$ = 0.34 in 9:1 hexanes/EtOAc) as a white solid.

**Data for Indole 5ba.**
**$^1$H NMR** (400 MHz, $CDCl_3$): δ 8.42 (d, $J$ = 7.7 Hz, 1H), 7.45 (d, $J$ = 7.4 Hz, 1H), 7.29 – 7.21 (comp. m, 2H), 3.36 – 3.30 (m, 1H), 3.14 (dddd, $J$ = 12.2, 9.1, 4.0, 2.5 Hz, 1H), 3.04 (app. dt, $J$ = 14.8, 7.4 Hz, 1H), 2.74 (app. dtd, $J$ = 12.6, 8.4, 4.0 Hz, 1H), 2.56 (s, 3H), 2.06 (app. ddt, $J$ = 12.6, 9.1, 6.2 Hz, 1H), 1.38 (dd, $J$ = 14.7, 2.5 Hz, 1H), 0.75 (dd, $J$ = 14.7, 12.2 Hz, 1H), 0.09 (s, 9H).
**$^{13}$C NMR** (100 MHz, $CDCl_3$): δ 169.2, 141.0, 140.5, 132.7, 126.6, 123.9, 123.5, 118.2, 117.5, 37.3, 34.2, 29.9, 25.4, 23.6, -0.5.
**IR** (film): 2945, 2859, 1700, 1477, 1449, 1379, 1349, 1314, 1244, 1198, 1033, 862, 838, 754 $cm^{-1}$.
**HRMS** ($ESI^+$): *m/z* calc'd for $(M + H)^+$ $[C_{17}H_{23}NOSi + H]^+$: 286.1622, found 286.1621.

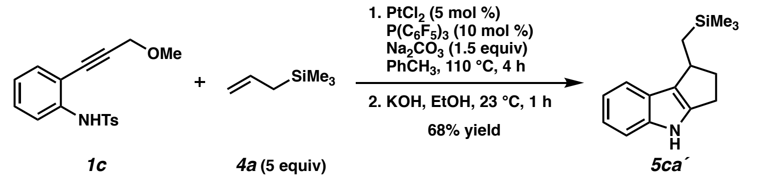


**Indole 5ca´.** To a solution of $P(C_6F_5)_3$ (9.0 mg, 0.0170 mmol) in toluene (0.85 mL) at 23 °C was added $PtCl_2$ (2.3 mg, 0.00850 mmol), and the resulting solution was stirred for 15 min. This catalyst solution was then added to a prestirred solution of alkyne **1c** (53.7 mg, 0.170 mmol) and allyltrimethylsilane (**4a**, 135 µL, 0.850 mmol) in toluene (0.85 mL, 0.1 M in substrate final concentration) at 23 °C. $Na_2CO_3$ (27.0 mg, 0.255 mmol) was then added, and the resulting mixture was stirred in a 110 °C heating block for 4 h. Upon completion, the reaction mixture was cooled to room temperature and filtered through a $SiO_2$ plug (0.5 x 3 cm), eluting with 1:1 EtOAc/hexanes (5 mL). The solvent was removed by rotary evaporation, and the resulting residue was dissolved in EtOH (0.76 mL). KOH (0.128 g, 2.28 mmol) was then added, and the resulting mixture was stirred at 23 °C for 1 h. Upon reaction completion, $Et_2O$ (3 mL) was added, and the reaction mixture was filtered through a celite plug, eluting with $Et_2O$ (3 mL). The filtrate was concentrated in vacuo, and the resulting residue was purified by flash chromatography (3:1 hexanes/EtOAc w/ 2% $Et_3N$ eluent), affording indole **5ca´** (28.2 mg, 68% yield, $R_f$ = 0.39 in 3:1 hexanes/EtOAc w/ 2% $Et_3N$) as a colorless oil. Decomposition of NMR samples was best prevented by briefly passing argon over the sample solution.

**Data for Indole 5ca´.**
**$^1$H NMR** (400 MHz, $CDCl_3$): 7.75 (br. s, 1H), 7.51 (d, *J* = 7.7 Hz, 1H), 7.30 (d, *J* = 7.4 Hz, 1H), 7.12 – 7.05 (comp. m, 2H), 3.45 – 3.35 (m, 1H), 2.89 (dddd, *J* = 12.1, 9.2, 3.9, 2.6 Hz, 1H), 2.82 (app. dt, *J* = 14.7, 7.3 Hz, 1H), 2.72 (app. dtd, *J* = 12.4, 7.4, 3.9 Hz, 1H), 2.04 (app. ddt, *J* = 12.4, 9.2, 6.2 Hz, 1H), 1.45 (dd, *J* = 14.7, 2.6 Hz, 1H), 0.79 (dd, *J* = 14.7, 12.1 Hz, 1H), 0.08 (s, 9H).
**$^{13}$C NMR** (100 MHz, $CDCl_3$): δ 142.1, 141.1, 125.9, 124.4, 120.5, 119.5, 118.2, 111.5, 38.4, 34.8, 25.8, 24.3, -0.5.
**IR** (film): 3408, 2951, 2876, 2360, 2342 cm$^{-1}$.
**HRMS** (ESI$^+$): *m/z* calc'd for $(M + H)^+$ $[C_{15}H_{21}NSi + H]^+$: 244.1516, found 244.1518.

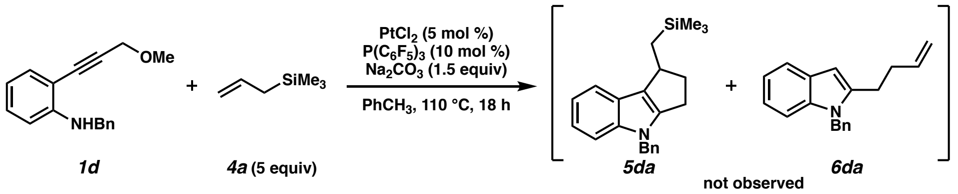


**Indoles 5da/6da.** To a solution of $P(C_6F_5)_3$ (5.3 mg, 0.0100 mmol) in toluene (0.50 mL) at 23 °C was added $PtCl_2$ (1.3 mg, 0.00500 mmol), and the resulting solution was stirred for 15 min. The catalyst solution was then added to a prestirred solution of alkyne **1d** (25.1 mg, 0.100 mmol) and allyltrimethylsilane **4a** (79.5 µL, 0.500 mmol) in toluene (0.50 mL, 0.1 M in substrate final concentration) at 23 °C. $Na_2CO_3$ (15.9 mg, 0.150 mmol) was then added, and the resulting mixture was stirred in a 110 °C heating block. After 18 h, the reaction mixture was cooled to room temperature and filtered through a $SiO_2$ plug (0.5 x 3 cm), eluting with 1:1 EtOAc/hexanes (4 mL). The solvent was removed by rotary evaporation, and the resulting crude residue was analyzed by $^1$H NMR. Cycloadduct **5da** was not observed, nor was potential allylation product **6da**.

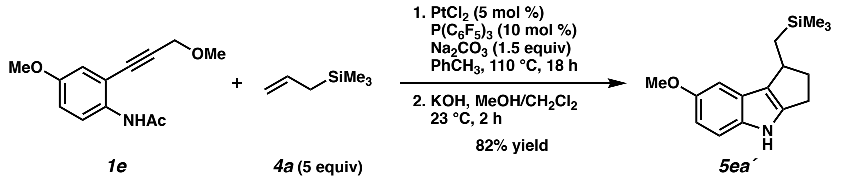

**Indole 5ea′.** To a solution of $P(C_6F_5)_3$ (11.4 mg, 0.0214 mmol) in toluene (1.07 mL) at 23 °C was added $PtCl_2$ (2.8 mg, 0.0107 mmol), and the resulting solution was stirred for 15 min. This catalyst solution was then added to a prestirred solution of alkyne **1e** (49.9 mg, 0.214 mmol) and allyltrimethylsilane (**4a**, 170 µL, 1.07 mmol) in toluene (1.07 mL, 0.1 M in substrate final concentration) at 23 °C. $Na_2CO_3$ (34.0 mg, 0.321 mmol) was then added, and the resulting mixture was stirred in a 110 °C heating block for 18 h. The reaction mixture was cooled to room temperature and filtered through a $SiO_2$ plug (0.5 x 3 cm), eluting with 1:1 EtOAc/hexanes (5 mL). The solvent was removed by rotary evaporation, and the resulting residue was dissolved in a 2:1 $MeOH/CH_2Cl_2$ mixture (1.65 mL). KOH (60.0 mg, 1.07 mmol) was added, and the resulting mixture was stirred at 23 °C for 2 h. Upon reaction completion, the mixture was diluted with $CH_2Cl_2$ (5 mL) and washed sequentially with sat. aq. $NH_4Cl$ (5 mL) and brine (5 mL), and then dried over $MgSO_4$. The mixture was concentrated in vacuo, and the resulting residue was purified by flash chromatography (3:1 hexanes/EtOAc w/ 2% $Et_3N$ eluent) to afford indole **5ea′** (48.0 mg, 82% yield, $R_f$ = 0.30 in 3:1 hexanes/EtOAc w/ 2% $Et_3N$) as a colorless oil. Decomposition of NMR samples was best prevented by briefly passing argon over the sample solution.

**Data for Indole 5ea′.**
**$^1$H NMR** (400 MHz, $CDCl_3$): δ 7.18 (d, $J$ = 8.8 Hz, 1H), 6.97 (d, $J$ = 2.1 Hz, 1H), 6.74 (dd, $J$ = 8.8, 2.1 Hz, 1H), 3.86 (s, 3H), 3.39 – 3.31 (m, 1H), 2.89 – 2.76 (comp. m, 2H), 2.71 (app. dtd, $J$ = 12.3, 8.0, 4.1 Hz, 1H), 2.02 (app. ddt, $J$ = 12.3, 8.8, 6.0 Hz, 1H), 1.41 (dd, $J$ = 14.6, 2.6 Hz, 1H), 0.78 (dd, $J$ = 14.6, 11.6 Hz, 1H), 0.09 (s, 9H).
**$^{13}$C NMR** (100 MHz, $CDCl_3$): δ 154.0, 143.1, 136.1, 125.6, 124.8, 111.9, 109.7, 101.2, 56.2, 38.4, 34.7, 25.7, 24.2, -0.4.
**IR** (film): 3405, 2952, 2361, 2341, 1664 cm$^{-1}$.
**HRMS** (ESI$^+$): *m/z* calc'd for $(M + H)^+$ $[C_{16}H_{23}NOSi + H]^+$: 274.1622, found 274.1625.

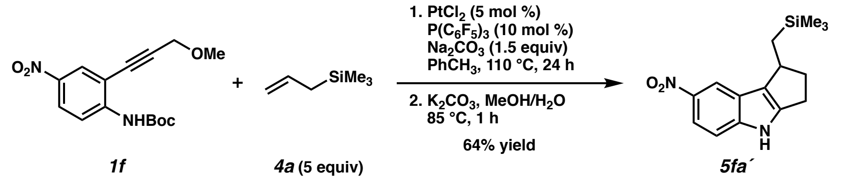

**Indole 5fa′.** To a solution of $P(C_6F_5)_3$ (9.6 mg, 0.0181 mmol) in toluene (0.90 mL) at 23 °C was added $PtCl_2$ (2.4 mg, 0.00905 mmol), and the resulting solution was stirred for 15 min. This catalyst solution was then added to a prestirred solution of alkyne **if** (55.4 mg, 0.181 mmol) and allyltrimethylsilane (**4a**, 144 µL, 0.905 mmol) in toluene (0.90 mL, 0.1 M in substrate final concentration) at 23 °C. $Na_2CO_3$ (28.8 mg, 0.272 mmol) was then added, and the resulting mixture was stirred in a 110 °C heating block for 24 h. Upon completion, the reaction mixture was cooled to room temperature and filtered through a $SiO_2$ plug (0.5 x 3 cm), eluting with 1:1 EtOAc/hexanes (5 mL). The solvent was removed by rotary evaporation, and the resulting residue was dissolved in a 3:1 $MeOH/H_2O$ mixture (2.41 mL). $K_2CO_3$ (50.0 mg, 0.362 mmol) was added, and the resulting mixture was stirred in an 85 °C heating block for 1 h. Upon reaction completion, the solution was allowed to cool to room temperature, and MeOH was removed by rotary evaporation. EtOAc (5 mL) was added, and the phases were separated. The aqueous phase was extracted with EtOAc (2 x 5 mL), and the combined organic phases were washed with brine (5 mL) and dried over

$MgSO_4$. The mixture was concentrated in vacuo, and the resulting residue was purified by flash chromatography (9:1 hexanes/EtOAc w/ 2% $Et_3N$ eluent) to afford indole **5fa′** (33.6 mg, 64% yield, $R_f$ = 0.23 in 9:1 hexanes/EtOAc in 2% $Et_3N$) as a colorless oil. Decomposition of NMR samples was best prevented by briefly passing argon over the sample solution.

**Data for Indole 5fa′.**
**$^1$H NMR** (400 MHz, $CDCl_3$): δ 8.44 (d, $J$ = 2.4 Hz, 1H), 8.26 (br. s, 1H), 8.00 (dd, $J$ = 9.0, 2.4 Hz, 1H), 7.30 (d, $J$ = 9.0 Hz, 1H), 3.47 – 3.36 (m, 1H), 2.97 – 2.89 (m, 1H), 2.86 (app. dt, $J$ = 14.6, 7.4 Hz, 1H), 2.76 (app dtd, $J$ = 12.6, 8.4, 4.0 Hz, 1H), 2.08 (app. ddt, $J$ = 12.6, 9.0, 6.6 Hz, 1H), 1.44 (dd, $J$ = 14.6, 2.6 Hz, 1H), 0.79 (dd, $J$ = 14.6, 11.8 Hz, 1H), 0.10 (s, 9H).
**$^{13}$C NMR** (100 MHz, $CDCl_3$): δ 145.7, 144.1, 141.6, 128.1, 123.6, 116.4, 115.2, 111.2, 38.2, 34.9, 25.8, 24.3, -0.5.
**IR** (film): 3368, 2951, 2852, 2349, 2283 $cm^{-1}$.
**HRMS** ($ESI^+$): $m/z$ calc'd for $(M + H)^+$ $[C_{15}H_{20}N_2O_2Si + H]^+$: 289.1367, found 289.1371.

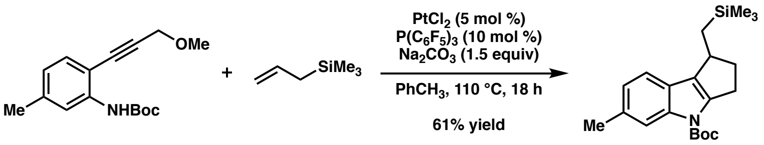


*1g* *4a* (5 equiv) *5ga*

**Indole 5ga.** To a solution of $P(C_6F_5)_3$ (10.6 mg, 0.0200 mmol) in toluene (1.00 mL) at 23 °C was added $PtCl_2$ (2.7 mg, 0.0100 mmol), and the resulting solution was stirred for 15 min. This catalyst solution was then added to a prestirred solution of alkyne **1g** (55.1 mg, 0.200 mmol) and allyltrimethylsilane (**4a**, 159 µL, 1.00 mmol) in toluene (1.00 mL, 0.1 M in substrate final concentration) at 23 °C. $Na_2CO_3$ (31.8 mg, 0.300 mmol) was then added, and the resulting mixture was stirred in a 110 °C heating block for 18 h. Upon completion, the reaction mixture was cooled to room temperature and filtered through a $SiO_2$ plug (0.5 x 3 cm), eluting with 1:1 EtOAc/hexanes (5 mL). The solvent was removed by rotary evaporation, and the resulting residue was purified by flash chromatography (4:1 hexanes/$CH_2Cl_2$ eluent) to afford indole **5ga** (43.6 mg, 61% yield, $R_f$ = 0.51 in 19:1 hexanes/EtOAc) as a white solid.

**Data for Indole 5ga.**
**$^1$H NMR** (400 MHz, $CDCl_3$): δ 8.02 (s, 1H), 7.30 (d, $J$ = 8.0 Hz, 1H), 7.01 (d, $J$ = 8.0 Hz, 1H), 3.32-3.25 (comp. m, 1H), 3.09 (dddd, $J$ = 12.1, 9.3, 4.1, 2.3 Hz, 1H), 2.97 (app. dt, $J$ = 15.2, 7.1 Hz, 1H), 2.65 (app. dtd, $J$ = 13.0, 8.4, 4.3 Hz, 1H), 2.45 (s, 3H), 1.97 (app. ddt, $J$ = 12.5, 9.4, 6.0 Hz, 1H), 1.63 (s, 9H), 1.35 (dd, $J$ = 14.7, 2.5 Hz, 1H), 0.73 (dd, $J$ = 14.7, 11.8 Hz, 1H), 0.08 (s, 9H).
**$^{13}$C NMR** (100 MHz, $CDCl_3$): δ 150.3, 141.5, 140.9, 132.8, 130.1, 124.2, 123.8, 117.8, 116.4, 82.9, 37.1, 34.5, 29.0, 28.4, 23.8, 22.1, -0.5.
**IR** (film): 2949, 2863, 1724, 1611, 1367, 1155, 1110, 858, 833, 768 $cm^{-1}$.
**HRMS** ($ESI^+$): $m/z$ calc'd for $(M + H)^+$ $[C_{21}H_{31}NO_2Si + H]^+$: 358.2197, found 358.2185.

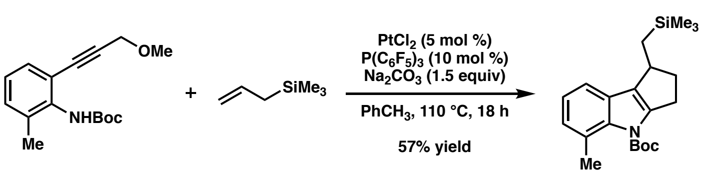


*1h* *4a* (5 equiv) *5ha*

**Indole 5ha.** To a solution of $P(C_6F_5)_3$ (10.6 mg, 0.0200 mmol) in toluene (1.00 mL) at 23 °C was added $PtCl_2$ (2.7 mg, 0.0100 mmol), and the resulting solution was stirred for 15 min. This catalyst solution was then added to a prestirred solution of alkyne **1h** (55.1 mg, 0.200 mmol) and allyltrimethylsilane (**4a**, 159 μL, 1.00 mmol) in toluene (1.00 mL, 0.1 M in substrate final concentration) at 23 °C. $Na_2CO_3$ (31.8 mg, 0.300 mmol) was then added, and the resulting mixture was stirred in a 110 °C heating block for 18 h. Upon completion, the reaction mixture was cooled to room temperature and filtered through a $SiO_2$ plug (0.5 x 3 cm), eluting with 1:1 EtOAc/hexanes (5 mL). The solvent was removed by rotary evaporation, and the resulting residue was purified by flash chromatography (4:1 hexanes/$CH_2Cl_2$ eluent) to afford indole **5ha** (40.8 mg, 57% yield, $R_f$ = 0.49 in 19:1 hexanes/EtOAc) as a colorless oil. (*Note*: a minor amount of a tetrahydrocarbazole side product was present in the isolated material, evidenced by the $^{13}$C NMR signal at -3.3 ppm.)

**Data for Indole 5ha.**
$^1$**H NMR** (400 MHz, $CDCl_3$): δ 7.27 (d, *J* = 8.1 Hz, 1H), 7.12 (app. t, *J* = 7.5 Hz, 1H), 7.01 (d, *J* = 7.4 Hz, 1H), 3.36-3.27 (comp. m, 1H), 3.06 (dddd, *J* = 11.8, 9.1, 3.9, 2.1 Hz, 1H), 2.94 (app. dt, *J* = 14.8, 7.2 Hz, 1H), 2.66 (app. dtd, *J* = 12.9, 8.0, 4.3 Hz, 1H), 2.54 (s, 3H), 1.98 (app. ddt, *J* = 12.6, 9.5, 6.3 Hz, 1H), 1.61 (s, 9H), 1.36 (dd, *J* = 14.6, 2.5 Hz, 1H), 0.75 (dd, *J* = 14.6, 12.1 Hz, 1H), 0.08 (s, 9H).
$^{13}$**C NMR** (100 MHz, $CDCl_3$): δ 149.9, 143.4, 139.7, 129.8, 127.9, 126.4, 126.1, 123.0, 115.9, 82.8, 36.9, 34.7, 29.3, 28.3, 23.8, 21.9, -0.5.
**IR** (film): 2951, 2931, 1740, 1455, 1347, 1321, 1159, 1095, 860, 742 $cm^{-1}$.
**HRMS** ($ESI^+$): *m/z* calc'd for $(M + H)^+$ $[C_{21}H_{31}NO_2Si + H]^+$: 358.2197, found 358.2185.

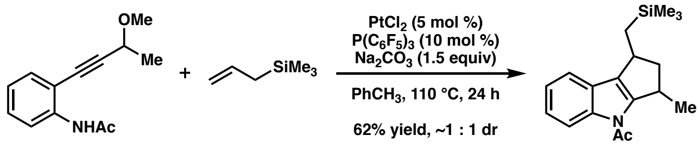


*1i* *4a* (5 equiv) *5ia*

**Indole 5ia.** To a solution of $P(C_6F_5)_3$ (12.2 mg, 0.0230 mmol) in toluene (1.15 mL) at 23 °C was added $PtCl_2$ (3.1 mg, 0.0115 mmol), and the resulting solution was stirred for 15 min. This catalyst solution was then added to a prestirred solution of alkyne **1i** (50.0 mg, 0.230 mmol) and allyltrimethylsilane (**4a**, 183 μL, 1.15 mmol) in toluene (1.15 mL, 0.1 M in substrate final concentration) at 23 °C. $Na_2CO_3$ (36.6 mg, 0.345 mmol) was then added, and the resulting mixture was stirred in a 110 °C heating block for 24 h. Upon completion, the reaction mixture was cooled to room temperature and filtered through a $SiO_2$ plug (0.5 x 3 cm), eluting with 1:1 EtOAc/hexanes (4 mL). The solvent was removed by rotary evaporation, and the resulting residue was purified by flash chromatography (9:1 hexanes/EtOAc eluent) to afford indole **5ia** (42.6 mg, 62% yield, approx. 1:1 dr, $R_f$ = 0.22 in 9:1 hexanes/EtOAc) as a colorless oil.

**Data for Indole 5ia.**
$^1$**H NMR** (400 MHz, $CDCl_3$, mixture of both diastereomers): δ 8.27 (d, *J* = 7.3 Hz, 0.5H), 8.17 (d, *J* = 8.0 Hz, 0.5H), 7.52 – 7.48 (m, 0.5H), 7.43 – 7.39 (m, 0.5H), 7.29 – 7.21 (comp. m, 2H), 3.47 (app. q, *J* = 7.1 Hz, 1H), 3.43 – 3.36 (m, 0.5H), 3.22 (app. tt, *J* = 8.8, 2.2 Hz, 0.5H), 3.00 (app. dt, *J* = 13.0, 8.8 Hz, 0.5H), 2.66 (s, 1.5H), 2.65 (s, 1.5H), 2.36 – 2.20 (comp. m, 1H), 1.78 (app. dt, *J* = 13.0, 2.2 Hz, 0.5H), 1.54 (dd, *J* = 14.6, 2.6 Hz, 0.5H), 1.37 (d, *J* = 6.8 Hz, 1.5H), 1.33 (dd, *J* = 14.8, 2.2 Hz, 0.5H), 1.24 (d, *J* = 6.8 Hz, 1.5H), 0.75 (dd, *J* = 14.6, 12.4 Hz, 0.5H), 0.70 (dd, *J* = 14.8, 12.1 Hz, 0.5H), 0.12 (s, 4.5H), 0.10 (s, 4.5H).
$^{13}$**C NMR** (100 MHz, $CDCl_3$, mixture of both diastereomers): δ 169.0, 168.9, 146.2, 146.1, 141.03, 141.01, 132.5, 131.4, 126.9, 126.5, 123.94, 123.87, 123.4, 123.3, 118.7, 118.6, 117.0, 116.6, 47.1, 45.2, 36.8, 36.7, 32.82, 32.75, 25.6, 25.4, 25.0, 23.6, 23.4, 20.7, -0.5, -0.6.
**IR** (film): 2953, 2897, 2348, 2283, 1702 $cm^{-1}$.

**HRMS** ($ESI^+$): *m/z* calc'd for $(M + H)^+$ $[C_{18}H_{25}NOSi + H]^+$: 300.1778, found 300.1782.

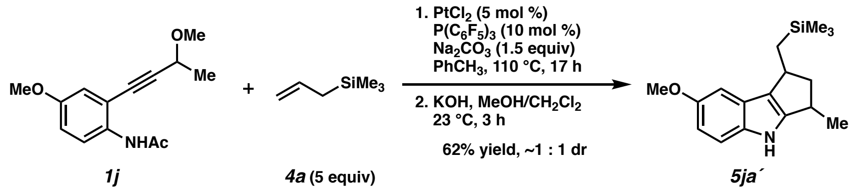


**Indole 5ja´.** To a solution of $P(C_6F_5)_3$ (10.1 mg, 0.0189 mmol) in toluene (0.95 mL) at 23 °C was added $PtCl_2$ (2.5 mg, 0.00945 mmol), and the resulting solution was stirred for 15 min. This catalyst solution was then added to a prestirred solution of alkyne **1j** (46.7 mg, 0.189 mmol) and allyltrimethylsilane (**4a**, 150 µL, 0.945 mmol) in toluene (0.95 mL, 0.1 M in substrate final concentration) at 23 °C. $Na_2CO_3$ (30.1 mg, 0.284 mmol) was then added, and the resulting mixture was stirred in a 110 °C heating block for 17 h. Upon completion, the reaction mixture was cooled to room temperature and filtered through a $SiO_2$ plug (0.5 x 3 cm), eluting with 1:1 EtOAc/hexanes (5 mL). The solvent was removed by rotary evaporation, and the resulting residue was dissolved in a 2:1 $MeOH/CH_2Cl_2$ mixture (1.45 mL). KOH (53.0 mg, 0.945 mmol) was added, and the resulting mixture was stirred at 23 °C for 3 h. Upon completion, the mixture was diluted with $CH_2Cl_2$ (5 mL), washed sequentially with sat. aq. $NH_4Cl$ (5 mL) and brine (5 mL), and then dried over $MgSO_4$. The mixture was concentrated in vacuo, and the resulting residue was purified by flash chromatography (3:1 hexanes/EtOAc w/ 2% $Et_3N$ eluent) to afford indole **5ja´** (33.8 mg, 62% yield, approx. 1:1 dr, $R_f$ = 0.41 in 3:1 hexanes/EtOAc w/ 2% $Et_3N$) as a colorless oil. Decomposition of NMR samples was best prevented by briefly passing argon over the sample solution.

**Data for Indole 5ja´.**
**$^1$H NMR** (400 MHz, $CDCl_3$, mixture of both diastereomers): δ 7.61 (br. s, 1H), 7.18 (app. d, *J* = 8.7 Hz, 1H), 7.00 (d, *J* = 2.1 Hz, 0.5H), 6.96 (d, *J* = 2.3 Hz, 0.5H) 6.75 (app. dd, *J* = 8.7, 2.3 Hz, 1H), 3.86 (app. s, 3H), 3.45 – 3.36 (m, 0.5H), 3.30 – 3.23 (comp. m, 1.5H), 2.90 (app. dt, *J* = 12.4, 7.2 Hz, 0.5H), 2.27 (app. td, *J* = 6.9, 1.9 Hz, 1H), 1.60 – 1.50 (comp. m, 1H), 1.32 (dd, *J* = 14.7, 2.8 Hz, 0.5H), 1.31 (d, *J* = 6.7 Hz, 1.5H), 1.23 (d, *J* = 7.0 Hz, 1.5H), 0.76 (dd, *J* = 15.2, 11.3 Hz, 0.5H), 0.75 (dd, *J* = 14.7, 11.6 Hz, 0.5H), 0.10 (s, 4.5H), 0.08 (s, 4.5H).
**$^{13}$C NMR** (100 MHz, $CDCl_3$, mixture of both diastereomers): δ 154.1, 154.0, 147.8, 147.6, 136.2, 136.1, 125.0, 124.8, 124.64, 124.60, 112.1, 112.0, 109.9, 109.8, 101.37, 101.37, 56.3, 56.2, 48.5, 47.6, 34.6, 33.8, 33.4, 33.0, 24.7, 24.1, 20.54, 20.51, -0.4, -0.5.
**IR** (film): 2953, 2360, 2342, 1728, 1475, 1458, 1370, 1323, 1248, 1222, 1166, 1112, 837, 765 $cm^{-1}$.
**HRMS** ($ESI^+$): *m/z* calc'd for $(M + H)^+$ $[C_{17}H_{25}NOSi + H]^+$: 288.1778, found 288.1783.

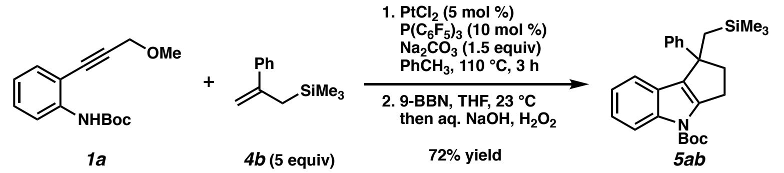


**Indole 5ab.** To a solution of $P(C_6F_5)_3$ (10.2 mg, 0.0192 mmol) in toluene (0.95 mL) at 23 °C was added $PtCl_2$ (2.6 mg, 0.00960 mmol), and the resulting solution was stirred for 15 min. This catalyst solution was then added to a prestirred solution of alkyne **1a** (50.2 mg, 0.192 mmol) and allylic trimethylsilane **4b** (183 mg, 0.960 mmol) in toluene (0.95 mL, 0.1 M in substrate final concentration) at 23 °C. $Na_2CO_3$ (30.5 mg, 0.288 mmol) was then added, and the resulting mixture was stirred in a 110 °C heating block for 3 h. Upon completion, the reaction mixture was cooled to room temperature and filtered through a $SiO_2$ plug (0.5 x 3

cm), eluting with 1:1 EtOAc/hexanes (5 mL). The solvent was removed by rotary evaporation, and the resulting residue was purified by flash chromatography (9:1 hexanes/EtOAc eluent) to afford a mixture of indole cycloadduct **5ab** and alkene products.

To the resulting mixture of indole products (assume 0.192 mmol) in THF (1.92 mL) was added 9-BBN (70.3 mg, 0.576 mmol) at 23 °C in an argon filled box. The mixture was stirred at 23 °C for 14 h. 3 N NaOH (0.640 mL, 0.3 M relative to substrate) and 30% $H_2O_2$ (0.640 mL, 0.3 M relative to substrate) were then added sequentially, and the resulting mixture was stirred at 23 °C for 3 h. Upon completion, the solution was diluted with brine (5 mL) and extracted with EtOAc (3 x 5 mL). The combined organic layers were dried over $MgSO_4$, and the solvent was removed by rotary evaporation. The resulting residue was purified by flash chromatography (9:1 hexanes/EtOAc eluent), providing indole **5ab** (58.2 mg, 72% yield, $R_f$ = 0.38 in 9:1 hexanes/EtOAc) as a colorless oil.

**Data for Indole 5ab.**
**$^{1}$H NMR** (400 MHz, $CDCl_3$): δ 8.19 (d, *J* = 8.2 Hz, 1H), 7.42 – 7.38 (comp. m, 2H), 7.37 (d, *J* = 7.7 Hz, 1H), 7.24 (d, *J* = 7.3 Hz, 2H), 7.22 – 7.11 (comp. m, 3H), 3.15 (app. t, *J* = 7.0 Hz, 2H), 2.79 (app. dt, *J* = 13.3, 6.5 Hz, 1H), 2.71 (app. dt, *J* = 13.3, 7.4 Hz, 1H), 1.84 (d, *J* = 14.8 Hz, 1H), 1.66 (s, 9H), 1.58 (d, *J* = 14.8 Hz, 1H), -0.21 (s, 9H).
**$^{13}$C NMR** (100 MHz, $CDCl_3$): δ 151.2, 150.2, 130.8, 128.34, 128.29, 126.8, 126.5, 126.0, 125.7, 123.1, 122.7, 119.2, 116.0, 83.4, 49.9, 45.4, 31.3, 28.7, 28.4, 0.3.
**IR** (film): 3053, 2951, 2360, 2341, 1732 $cm^{-1}$.
**HRMS** ($ESI^+$): *m/z* calc'd for $(M + Na)^+$ $[C_{26}H_{33}NO_2Si + Na]^+$: 442.2173, found 442.2168.

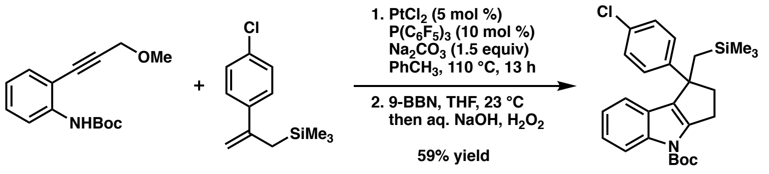

*1a* *4c* (5 equiv) *5ac*

**Indole 5ac.** To a solution of $P(C_6F_5)_3$ (9.5 mg, 0.0179 mmol) in toluene (0.90 mL) at 23 °C was added $PtCl_2$ (2.4 mg, 0.00895 mmol), and the resulting solution was stirred for 15 min. This catalyst solution was then added to a prestirred solution of alkyne **1a** (46.7 mg, 0.179 mmol) and allylic trimethylsilane **4c** (201 mg, 0.895 mmol) in toluene (0.90 mL, 0.1 M in substrate final concentration) at 23 °C. $Na_2CO_3$ (28.5 mg, 0.269 mmol) was then added, and the resulting mixture was stirred in a 110 °C heating block for 13 h. Upon completion, the reaction mixture was cooled to room temperature and filtered through a $SiO_2$ plug (0.5 x 3 cm), eluting with 1:1 EtOAc/hexanes (5 mL). The solvent was removed by rotary evaporation, and the resulting residue was purified by flash chromatography (9:1 hexanes/EtOAc eluent) to afford a mixture of indole cycloadduct **5ac** and alkene products.

To the resulting mixture of indole products (assume 0.179 mmol) in THF (1.80 mL) was added 9-BBN (65.5 mg, 0.537 mmol) at 23 °C in an argon filled box. The mixture was stirred at 23 °C for 14 h. 3 N NaOH (0.597 mL, 0.3 M relative to substrate) and 30% $H_2O_2$ (0.597 mL, 0.3 M relative to substrate) were then added sequentially, and the resulting mixture was stirred at 23 °C for 3 h. Upon completion, the solution was diluted with brine (5 mL) and extracted with EtOAc (3 x 5 mL). The combined organic layers were dried over $MgSO_4$, and the solvent was removed by rotary evaporation. The resulting residue was purified by flash chromatography (9:1 hexanes/EtOAc eluent), providing indole **5ac** (47.7 mg, 59% yield, $R_f$ = 0.46 in 9:1 hexanes/EtOAc) as a colorless oil.

**Data for Indole 5ac.**

$^{1}$**H NMR** (400 MHz, $CDCl_3$): δ 8.18 (d, *J* = 7.7 Hz, 1H), 7.32 – 7.28 (comp. m, 3H), 7.23 (app. t, *J* = 7.3 Hz, 1H), 7.20 (d, *J* = 8.6 Hz, 2H), 7.16 (app. t, *J* = 7.3 Hz, 1H), 3.15 (app. t, *J* = 7.0 Hz, 2H), 2.70 (app. t, *J* = 7.0 Hz, 2H), 1.78 (d, *J* = 14.7 Hz, 1H), 1.66 (s, 9H), 1.56 (d, *J* = 14.7 Hz, 1H), -0.22 (s, 9H).
$^{13}$**C NMR** (100 MHz, $CDCl_3$): δ 150.1, 149.9, 131.4, 130.3, 128.5, 128.31, 128.26, 127.5, 126.4, 123.2, 122.8, 119.0, 116.1, 83.5, 49.5, 45.5, 31.1, 28.7, 28.4, 0.3.
**IR** (film): 3036, 2953, 2372, 2347, 1732 $cm^{-1}$.
**HRMS** ($ESI^+$): *m/z* calc'd for $(M + Na)^+$ $[C_{26}H_{32}ClNO_2Si + Na]^+$: 476.1783, found 476.1792.

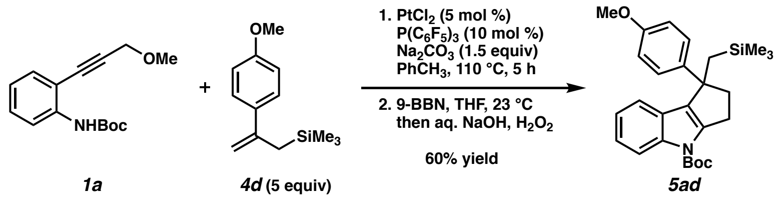


**Indole 5ad.** To a solution of $P(C_6F_5)_3$ (10.4 mg, 0.0195 mmol) in toluene (0.98 mL) at 23 °C was added $PtCl_2$ (2.6 mg, 0.00975 mmol), and the resulting solution was stirred for 15 min. This catalyst solution was then added to a prestirred solution of alkyne **1a** (51.0 mg, 0.195 mmol) and allylic trimethylsilane **4d** (215 mg, 0.975 mmol) in toluene (0.98 mL, 0.1 M in substrate final concentration) at 23 °C. $Na_2CO_3$ (31.1 mg, 0.293 mmol) was then added, and the resulting mixture was stirred in a 110 °C heating block for 5 h. Upon completion, the reaction mixture was cooled to room temperature and filtered through a $SiO_2$ plug (0.5 x 3 cm), eluting with 1:1 EtOAc/hexanes (5 mL). The solvent was removed by rotary evaporation, and the resulting residue was purified by flash chromatography (9:1 hexanes/EtOAc eluent) to afford a mixture of indole cycloadduct **5ad** and alkene products.

To the resulting mixture of indole products (assume 0.195 mmol) in THF (1.96 mL) was added 9-BBN (71.4 mg, 0.585 mmol) at 23 °C in an argon filled box. The mixture was stirred at 23 °C for 14 h. 3 N NaOH (0.650 mL, 0.3 M relative to substrate) and 30% $H_2O_2$ (0.650 mL, 0.3 M relative to substrate) were added sequentially, and the resulting mixture was stirred at 23 °C for 3 h. Upon completion, the solution was diluted with brine (5 mL) and extracted with EtOAc (3 x 5 mL). The combined organic layers were dried over $MgSO_4$, and the solvent was removed by rotary evaporation. The resulting residue was purified by flash chromatography (9:1 hexanes/EtOAc eluent), providing indole **5ad** (53.0 mg, 60% yield, $R_f$ = 0.47 in 9:1 hexanes/EtOAc) as a colorless oil.

**Data for Indole 5ad.**
$^{1}$**H NMR** (400 MHz, $CDCl_3$): δ 8.19 (d, *J* = 8.2 Hz, 1H), 7.37 (d, *J* = 7.3 Hz, 1H), 7.31 (d, *J* = 8.8 Hz, 2H), 7.23 (app. t, *J* = 7.7 Hz, 1H), 7.17 (app. t, *J* = 7.3 Hz, 1H), 6.79 (d, *J* = 8.8 Hz, 2H), 3.76 (s, 3H), 3.14 (app. t, *J* = 7.1 Hz, 2H), 2.76 (app. dt, *J* = 13.2, 6.5 Hz, 1H), 2.69 (app. dt, *J* = 13.2, 7.7 Hz, 1H), 1.82 (d, *J* = 14.7 Hz, 1H), 1.66 (s, 9H), 1.57 (d, *J* = 14.7 Hz, 1H), -0.20 (s, 9H).
$^{13}$**C NMR** (100 MHz, $CDCl_3$): δ 157.6, 150.2, 143.4, 140.2, 131.1, 127.6, 127.0, 126.7, 123.0, 122.7, 119.1, 116.0, 113.5, 83.3, 55.3, 49.2, 45.5, 31.4, 28.7, 28.4, 0.3.
**IR** (film): 3035, 2951, 2898, 2370, 1731 $cm^{-1}$.
**HRMS** ($ESI^+$): *m/z* calc'd for $(M + H)^+$ $[C_{27}H_{35}NO_3Si + H]^+$: 450.2459, found 450.2464.

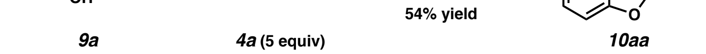

**Benzofuran 10aa.** To a solution of $P(C_6F_5)_3$ (16.0 mg, 0.0300 mmol) in toluene (1.50 mL) at 23 °C was added $PtCl_2$ (4.0 mg, 0.0150 mmol), and the resulting solution was stirred for 15 min. This catalyst solution was then added to a prestirred solution of alkyne **9a** (48.7 mg, 0.300 mmol) and allyltrimethylsilane (**4a**, 238 μL, 1.50 mmol) in toluene (1.50 mL, 0.1 M in substrate final concentration) at 23 °C. $Na_2CO_3$ (47.7 mg, 0.450 mmol) was then added, and the resulting mixture was stirred in an 80 °C heating block for 3 h. Upon completion, the reaction mixture was cooled to room temperature and filtered through a $SiO_2$ plug (0.5 x 3 cm), eluting with 1:1 EtOAc/hexanes (5 mL). The solvent was removed by rotary evaporation, and the resulting residue was purified by flash chromatography (9:1 hexanes/EtOAc eluent) to afford benzofuran **10aa** (39.8 mg, 54% yield, $R_f$ = 0.57 in 19:1 hexanes/EtOAc) as a colorless oil. (*Note*: A minor amount of a tetrahydrodibenzofuran side product was present in the isolated material, evidenced by the $^{13}C$ NMR signal at -3.4 ppm.)

**Data for Benzofuran 10aa.**
**$^1$H NMR** (400 MHz, $CDCl_3$): δ 7.47 – 7.38 (comp. m, 2H), 7.21 – 7.14 (comp. m, 2H), 3.37 – 3.27 (m, 1H), 2.93 – 2.65 (comp. m, 3H), 2.07 (app. ddt, *J* = 12.2, 8.6, 6.1 Hz, 1H), 1.34 (dd, *J* = 14.7, 2.9 Hz, 1H), 0.79 (dd, *J* = 14.7, 11.5 Hz, 1H), 0.09 (s, 9H).
**$^{13}$C NMR** (100 MHz, $CDCl_3$): δ 161.3, 160.5, 127.2, 126.3, 122.53, 122.46, 118.7, 111.9, 37.2, 33.2, 25.2, 24.0, -0.5.
**IR** (film): 2952, 2857, 1633, 1519, 1488, 1446, 1248, 1189, 1099, 985, 860, 836, 742 $cm^{-1}$.
**HRMS** ($ESI^+$): *m/z* calc'd for $(M + H)^+$ $[C_{15}H_{20}OSi + H]^+$: 245.1356 found 245.1348.

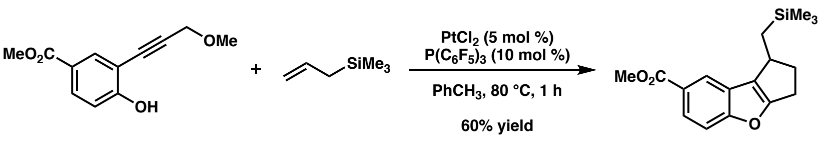


***9b*** ***4a*** (5 equiv) ***10ba***

**Benzofuran 10ba.** To a solution of $P(C_6F_5)_3$ (12.2 mg, 0.0229 mmol) in toluene (1.15 mL) at 23 °C was added $PtCl_2$ (3.1 mg, 0.0115 mmol), and the resulting solution was stirred for 15 min. A prestirred solution of allyltrimethylsilane (**4a**, 183 μL, 1.15 mmol) in toluene (1.15 mL, 0.1 M in substrate final concentration) was added to the catalyst solution at 23 °C. Alkyne **9b** (50.5 mg, 0.229 mmol) was then added, and the resulting mixture was stirred in an 80 °C heating block for 1 h. Upon completion, as determined by TLC, the reaction mixture was cooled to room temperature and filtered through a $SiO_2$ plug (0.5 x 3 cm), eluting with 1:1 EtOAc/hexanes (5 mL). The solvent was removed by rotary evaporation, and the resulting residue was purified by flash chromatography (19:1 hexanes/$Et_2O$ eluent) to afford benzofuran **10ba** (41.2 mg, 60% yield, $R_f$ = 0.48 in 19:1 hexanes/$Et_2O$) as a yellow oil. (*Note*: a minor amount of a tetrahydrodibenzofuran side product was present in the isolated material, evidenced by the $^{13}C$ NMR signal at -3.3 ppm.)

**Data for Benzofuran 10ba.**
**$^1$H NMR** (400 MHz, $CDCl_3$): δ 8.16 (s, 1H), 7.91 (d, *J* = 8.5 Hz, 1H), 7.42 (d, *J* = 8.5 Hz, 1H), 3.94 (s, 3H), 3.40 – 3.27 (m, 1H), 2.95 – 2.68 (comp. m, 3H), 2.09 (app. ddt, *J* = 12.2, 8.7, 6.1 Hz, 1H), 1.38 (dd, *J* = 14.6, 2.9 Hz, 1H), 0.78 (dd, *J* = 14.7, 11.5 Hz, 1H), 0.09 (s, 9H).
**$^{13}$C NMR** (100 MHz, $CDCl_3$): δ 167.7, 163.1, 162.9, 127.6, 126.2, 124.7, 124.5, 120.9, 111.7, 52.2, 37.0, 33.2, 25.3, 23.9, -0.5.
**IR** (film): 2951, 2860, 1722, 1613, 1433, 1285, 1144, 1017, 726 $cm^{-1}$.
**HRMS** ($ESI^+$): *m/z* calc'd for $(M + H)^+$ $[C_{17}H_{22}O_3Si + H]^+$: 303.1411 found 303.1389.

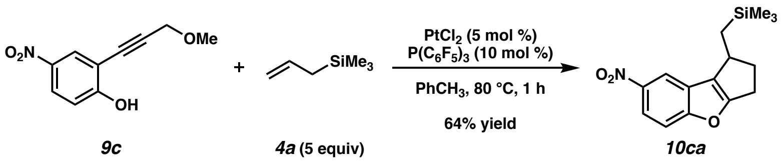

**Benzofuran 10ca.** To a solution of $P(C_6F_5)_3$ (13.3 mg, 0.0249 mmol) in toluene (1.25 mL) at 23 °C was added $PtCl_2$ (3.3 mg, 0.0125 mmol), and the resulting solution was stirred for 15 min. A prestirred solution of allyltrimethylsilane (**4a**, 199 µL, 1.25 mmol) in toluene (1.25 mL, 0.1 M in substrate final concentration) was added to the catalyst solution at 23 °C. Alkyne **9c** (51.6 mg, 0.249 mmol) was then added, and the resulting mixture was stirred in an 80 °C heating block for 1 h. Upon completion, as determined by TLC, the reaction mixture was cooled to room temperature and filtered through a $SiO_2$ plug (0.5 x 3 cm), eluting with 1:1 EtOAc/hexanes (5 mL). The solvent was removed by rotary evaporation, and the resulting residue was purified by flash chromatography (19:1 hexanes/$Et_2O$ eluent) to afford benzofuran **10ca** (46.0 mg, 64% yield, $R_f$ = 0.45 in 19:1 hexanes/$Et_2O$) as a light-yellow solid. (*Note*: A minor amount of a tetrahydrodibenzofuran side product was present in the isolated material, most evidenced by the $^{13}C$ NMR signal at -3.3 ppm.)

**Data for Benzofuran 10ca.**
**$^{1}$H NMR** (400 MHz, $CDCl_3$): δ 8.35 (d, *J* = 2.3 Hz, 1H), 8.13 (dd, *J* = 9.1, 2.3 Hz, 1H), 7.47 (d, *J* = 9.1 Hz, 1H), 3.40 – 3.30 (m, 1H), 2.99 – 2.68 (comp. m, 3H), 2.11 (app. ddt, *J* = 12.2, 8.8, 6.2 Hz, 1H), 1.35 (dd, *J* = 14.6, 2.8 Hz, 1H), 0.80 (dd, *J* = 14.6, 11.6 Hz, 1H), 0.10 (s, 9H).
**$^{13}$C NMR** (100 MHz, $CDCl_3$): δ 165.0, 163.3, 143.9, 128.1, 126.5, 118.6, 115.0, 112.0, 37.0, 33.2, 25.4, 24.0, -0.5.
**IR** (film): 2951, 2860, 2409, 2033, 1516, 1336, 1227, 1162, 1017 $cm^{-1}$.
**HRMS** ($ESI^+$): *m/z* calc'd for $(M + Na)^+$ $[C_{15}H_{19}NO_3Si + Na]^+$: 312.1026 found 312.1008.

# Platinum-Catalyzed Allylation Scope

### *Figure 9 (reproduced).*

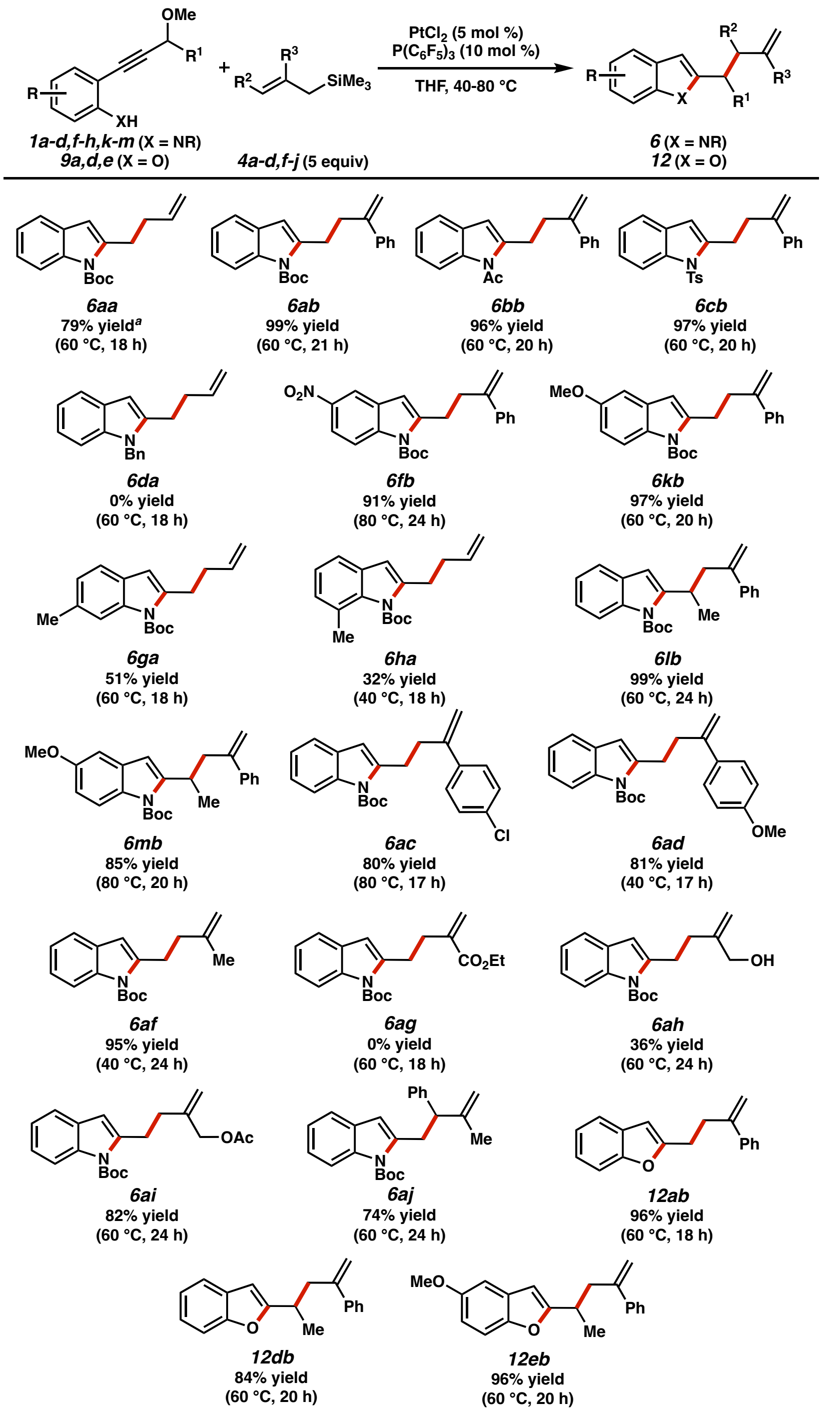


[a] The crude product was treated with TBAF prior to chromatographic purification to remove silylated byproducts.

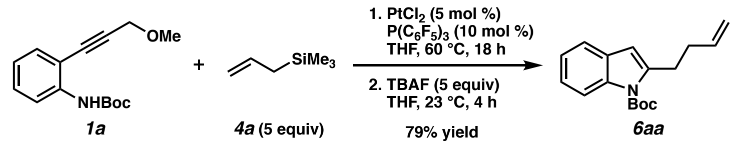


**Indole 6aa.** To a solution of $P(C_6F_5)_3$ (10.1 mg, 0.0190 mmol) in THF (0.95 mL) at 23 °C was added $PtCl_2$ (2.5 mg, 0.00950 mmol), and the resulting solution was stirred for 15 min. This catalyst solution was then added to a solution of alkyne **1a** (49.6 mg, 0.190 mmol) and allyltrimethylsilane (**4a**, 151 µL, 0.950 mmol) in THF (0.95 mL, 0.1 M in substrate final concentration) at 23 °C. The resulting mixture was heated in a 60 °C heating block and stirred for 18 h. The reaction mixture was then cooled to room temperature and filtered through a $SiO_2$ plug (0.5 x 3 cm), eluting with 1:1 hexanes/EtOAc (5 mL). The solvent was removed by rotary evaporation, and the resulting residue was dissolved in THF (1.90 mL) and stirred at 23 °C. TBAF (0.950 mL, 1.0 M in THF, 0.950 mmol) was then added dropwise, and the resulting mixture was stirred for 4 h. Upon completion, sat. aq. $NH_4Cl$ (2 mL) was added to the reaction mixture, and the THF was removed by rotary evaporation. EtOAc (5 mL) was added to the mixture, and the phases were separated. The aqueous phase was extracted with EtOAc (2 x 5 mL), and the combined organic phases were washed with brine (5 mL) and dried over $MgSO_4$. The solvent was removed via rotary evaporation, and the resulting residue was purified by flash chromatography (9:1 hexanes/EtOAc eluent) to afford indole **6aa** (40.9 mg, 79% yield, $R_f$ = 0.41 in 9:1 hexanes/EtOAc eluent) as a colorless oil.

**Data for Indole 6aa.**
**$^1$H NMR** (400 MHz, $CDCl_3$): δ 8.11 (d, $J$ = 8.1 Hz, 1H), 7.46 (d, $J$ = 7.3 Hz, 1H), 7.23 (app. t, $J$ = 8.1 Hz, 1H), 7.19 (app. t, $J$ = 7.3 Hz, 1H), 6.37 (s, 1H), 5.93 (ddt, $J$ = 17.2, 10.2, 7.0 Hz, 1H), 5.11 (d, $J$ = 17.2 Hz, 1H), 5.02 (d, $J$ = 10.2 Hz, 1H), 3.12 (t, $J$ = 7.3 Hz, 2H), 2.48 (app. q, $J$ = 7.0 Hz, 2H), 1.69 (s, 9H).
**$^{13}$C NMR** (100 MHz, $CDCl_3$): δ 150.7, 141.7, 138.0, 136.8, 129.4, 123.4, 122.7, 119.9, 115.7, 115.3, 107.4, 83.9, 33.1, 29.7, 28.4.
**IR** (film): 3238, 2978, 2360, 2204, 1732 $cm^{-1}$.
**HRMS** ($ESI^+$) *m/z* calc'd for $(M + H)^+$ $[C_{17}H_{21}NO_2 + H]^+$: 272.1645, found 272.1647.

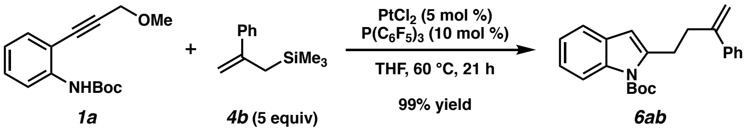


**Indole 6ab.** To a solution of $P(C_6F_5)_3$ (6.9 mg, 0.0130 mmol) in THF (0.65 mL) at 23 °C was added $PtCl_2$ (1.7 mg, 0.00650 mmol), and the resulting solution was stirred for 15 min. This catalyst solution was then added to a solution of alkyne **1a** (33.9 mg, 0.130 mmol) and allylic trimethylsilane **4b** (124 mg, 0.650 mmol) in THF (0.65 mL, 0.1 M in substrate final concentration) at 23 °C. The resulting mixture was stirred in a 60 °C heating block for 21 h. Upon completion, the reaction mixture was cooled to room temperature and filtered through a $SiO_2$ plug (0.5 x 3 cm), eluting with 1:1 EtOAc/hexanes (5 mL). The solvent was removed by rotary evaporation, and the resulting residue was purified by flash chromatography (9:1 hexanes/EtOAc eluent) to afford indole **6ab** (44.7 mg, 99% yield, $R_f$ = 0.53 in 9:1 hexanes/EtOAc) as a colorless oil.

**Data for Indole 6ab.**
**$^1$H NMR** (400 MHz, $CDCl_3$): δ 8.12 (d, $J$ = 8.0 Hz, 1H), 7.48 – 7.44 (comp. m, 3H), 7.36 (app. t, $J$ = 7.3 Hz, 2H), 7.30 (t, $J$ = 7.3 Hz, 1H), 7.25 (app. t, $J$ = 7.7 Hz, 1H), 7.20 (app. t, $J$ = 7.4 Hz, 1H), 6.37 (s, 1H), 5.35 (s, 1H), 5.15 (s, 1H), 3.19 (t, $J$ = 7.8 Hz, 2H), 2.94 (t, $J$ = 7.8 Hz, 2H), 1.65 (s, 9H).
**$^{13}$C NMR** (100 MHz, $CDCl_3$): δ 150.6, 147.8, 141.7, 141.3, 136.8, 129.4, 128.5, 127.6, 126.3, 123.4, 122.7, 119.9, 115.7, 112.9, 107.4, 83.8, 34.6, 29.2, 28.4.

**IR** (film): 2977, 2927, 2360, 2342, 1732, 1474, 1455, 1369, 1327, 1159, 1116, 1087, 746, 701 $cm^{-1}$.
**HRMS** ($ESI^+$) *m/z* calc'd for $(M + H)^+$ $[C_{23}H_{25}NO_2 + H]^+$: 348.1958, found 348.1958.

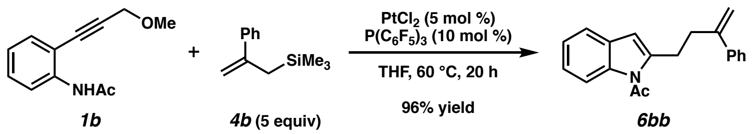

**Indole 6bb.** To a solution of $P(C_6F_5)_3$ (13.3 mg, 0.0249 mmol) in THF (1.25 mL) at 23 °C was added $PtCl_2$ (3.3 mg, 0.0125 mmol), and the resulting solution was stirred for 15 min. This catalyst solution was then added to a solution of alkyne **1b** (50.6 mg, 0.249 mmol) and allylic trimethylsilane **4b** (238 mg, 1.25 mmol) in THF (1.25 mL, 0.1 M in substrate final concentration) at 23 °C. The resulting mixture was stirred in a 60 °C heating block for 20 h. Upon completion, the reaction mixture was cooled to room temperature and filtered through a $SiO_2$ plug (0.5 x 3 cm), eluting with 1:1 EtOAc/hexanes (8 mL). The solvent was removed by rotary evaporation, and the resulting residue was purified by flash chromatography (4:1 hexanes/EtOAc eluent) to afford indole **6bb** (69.0 mg, 96% yield, $R_f$ = 0.48 in 4:1 hexanes/EtOAc) as a white solid.

**Data for Indole 6bb.**
**$^1$H NMR** (400 MHz, $CDCl_3$): δ 7.78 (d, *J* = 7.8 Hz, 1H), 7.51 – 7.45 (comp. m, 3H), 7.35 (app. t, *J* = 7.4 Hz, 2H), 7.29 (t, *J* = 7.4 Hz, 1H), 7.25 (app. t, *J* = 7.3 Hz, 1H), 7.23 (app. t, *J* = 7.3 Hz, 1H), 6.42 (s, 1H), 5.34 (s, 1H), 5.15 (s, 1H), 3.19 (t, *J* = 7.8 Hz, 2H), 2.94 (t, *J* = 7.8 Hz, 2H), 2.76 (s, 3H).
**$^{13}$C NMR** (100 MHz, $CDCl_3$): δ 170.4, 147.6, 142.4, 141.0, 136.3, 130.1, 128.5, 127.6, 126.3, 123.6, 123.1, 120.5, 114.7, 113.2, 108.8, 34.8, 29.8, 27.9.
**IR** (film): 2923, 2852, 2360, 2342, 1702, 1459, 1370, 1299, 1209, 741, 702 $cm^{-1}$.
**HRMS** ($ESI^+$) *m/z* calc'd for $(M + H)^+$ $[C_{20}H_{19}NO + H]^+$: 290.1539, found 290.1540.

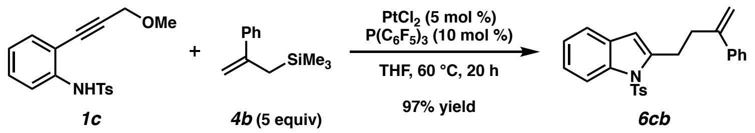

**Indole 6cb.** To a solution of $P(C_6F_5)_3$ (8.3 mg, 0.0156 mmol) in THF (0.78 mL) at 23 °C was added $PtCl_2$ (2.1 mg, 0.00780 mmol), and the resulting solution was stirred for 15 min. This catalyst solution was then added to a solution of alkyne **1c** (49.1 mg, 0.156 mmol) and allylic trimethylsilane **4b** (148 mg, 0.780 mmol) in THF (0.78 mL, 0.1 M in substrate final concentration) at 23 °C. The resulting mixture was stirred in a 60 °C heating block for 20 h. Upon completion, the reaction mixture was cooled to room temperature and filtered through a $SiO_2$ plug (0.5 x 3 cm), eluting with 1:1 EtOAc/hexanes (6 mL). The solvent was removed by rotary evaporation, and the resulting residue was purified by flash chromatography (4:1 hexanes/EtOAc eluent) to afford indole **6cb** (60.9 mg, 97% yield, $R_f$ = 0.54 in 4:1 hexanes/EtOAc) as a colorless oil.

**Data for Indole 6cb.**
**$^1$H NMR** (400 MHz, $CDCl_3$): δ 8.18 (d, *J* = 8.2 Hz, 1H), 7.55 (d, *J* = 8.4 Hz, 2H), 7.44 (d, *J* = 7.3 Hz, 2H), 7.41 (d, *J* = 7.3 Hz, 1H), 7.35 (app. t, *J* = 7.2 Hz, 2H), 7.31 (t, *J* = 7.2 Hz, 1H), 7.27 (app. t, *J* = 7.8 Hz, 1H), 7.21 (app. t, *J* = 7.3 Hz, 1H), 7.12 (d, *J* = 8.4 Hz, 2H), 6.39 (s, 1H), 5.34 (s, 1H), 5.14 (s, 1H), 3.16 (t, *J* = 7.6 Hz, 2H), 2.98 (t, *J* = 7.6 Hz, 2H), 2.31 (s, 3H).
**$^{13}$C NMR** (100 MHz, $CDCl_3$): δ 147.4, 144.7, 141.5, 140.9, 137.3, 136.1, 129.89, 129.87, 128.5, 127.6, 126.4, 126.3, 124.1, 123.6, 120.3, 115.0, 113.4, 109.3, 34.8, 28.2, 21.7.
**IR** (film): 2923, 2852, 1629, 1597, 1494, 1597, 1494, 1452, 1368, 1188, 1146, 1091, 748, 706, 668 $cm^{-1}$.

**HRMS** (ESI$^+$) *m/z* calc'd for $(M + H)^+$ $[C_{25}H_{23}NO_2S + H]^+$: 402.1522, found 402.1523.

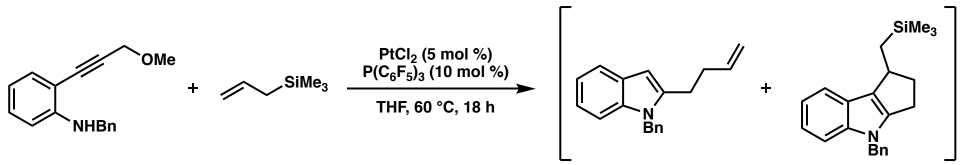


*1d* *4a* (5 equiv) *6da* *5da*
not observed

**Indoles 6da/5da.** To a solution of $P(C_6F_5)_3$ (5.3 mg, 0.0100 mmol) in THF (0.50 mL) at 23 °C was added $PtCl_2$ (1.3 mg, 0.00500 mmol), and the resulting solution was stirred for 15 min. The catalyst solution was then added to a prestirred solution of alkyne **1d** (25.1 mg, 0.100 mmol) and allyltrimethylsilane **4a** (79.5 µL, 0.500 mmol) in THF (0.50 mL, 0.1 M in substrate final concentration) at 23 °C. The resulting mixture was stirred in a 60 °C heating block. After 18 h, the reaction mixture was cooled to room temperature and filtered through a $SiO_2$ plug (0.5 x 3 cm), eluting with 1:1 EtOAc/hexanes (4 mL). The solvent was removed by rotary evaporation, and the resulting crude residue was analyzed by $^1$H NMR. Allylation product **6da** was not observed, nor was potential cycloaddition product **5da**.

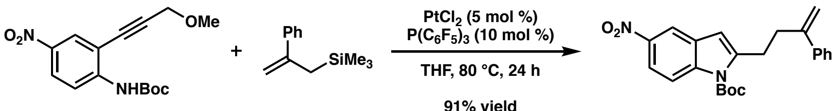


*1f* *4b* (5 equiv) *6fb*

**Indole 6fb.** To a solution of $P(C_6F_5)_3$ (5.3 mg, 0.0100 mmol) in THF (0.50 mL) at 23 °C was added $PtCl_2$ (1.3 mg, 0.00500 mmol), and the resulting solution was stirred for 15 min. This catalyst solution was then added to a solution of alkyne **1f** (30.7 mg, 0.100 mmol) and allylic trimethylsilane **4b** (95.2 mg, 0.500 mmol) in THF (0.50 mL, 0.1 M in substrate final concentration) at 23 °C. The resulting mixture was stirred in an 80 °C heating block for 24 h. Upon completion, the reaction mixture was cooled to room temperature and filtered through a $SiO_2$ plug (0.5 x 3 cm), eluting with 1:1 EtOAc/hexanes (5 mL). The solvent was removed by rotary evaporation, and the resulting residue was purified by flash chromatography (9:1 hexanes/EtOAc eluent) to afford indole **6fb** (35.9 mg, 91% yield, $R_f$ = 0.30 in 9:1 hexanes/EtOAc) as a pale yellow solid.

**Data for Indole 6fb.**
**$^1$H NMR** (400 MHz, $CDCl_3$): δ 8.35 (d, *J* = 2.3 Hz, 1H), 8.19 (d, *J* = 9.2 Hz, 1H), 8.12 (dd, *J* = 9.2, 2.3 Hz, 1H), 7.44 – 7.40 (m, 2H), 7.34 (app. t, *J* = 7.3 Hz, 2H), 7.29 (t, *J* = 7.3 Hz, 1H), 6.47 (s, 1H), 5.34 (s, 1H), 5.12 (s, 1H), 3.18 (t, *J* = 7.7 Hz, 2H), 2.94 (t, *J* = 7.7 Hz, 2H), 1.65 (s, 9H).
**$^{13}$C NMR** (100 MHz, $CDCl_3$): δ 149.9, 147.5, 144.9, 143.7, 141.0, 140.0, 129.2, 128.6, 127.8, 126.3, 118.8, 116.0, 115.7, 113.3, 107.8, 85.4, 34.4, 29.2, 28.2.
**IR** (film): 2926, 2853, 1740, 1629, 1570, 1519, 1447, 1371, 1344, 1324, 1152, 1091, 893, 769, 702 cm$^{-1}$.
**HRMS** (ESI$^+$) *m/z* calc'd for $(M + H)^+$ $[C_{23}H_{24}N_2O_4 + H]^+$: 393.1809, found 393.1809.

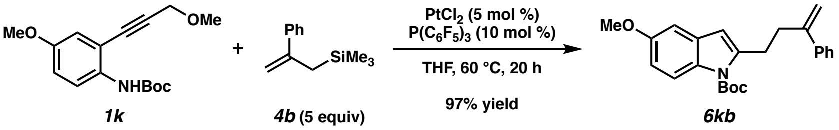


*1k* *4b* (5 equiv) *6kb*

**Indole 6kb.** To a solution of $P(C_6F_5)_3$ (6.0 mg, 0.0112 mmol) in THF (0.55 mL) at 23 °C was added $PtCl_2$ (1.5 mg, 0.00560 mmol), and the resulting solution was stirred for 15 min. This catalyst solution was then added to a solution of alkyne **1k** (32.7 mg, 0.112 mmol) and allylic trimethylsilane **4b** (107 mg, 0.560 mmol) in THF (0.55 mL, 0.1 M in substrate final concentration) at 23 °C. The resulting mixture was stirred in a 60 °C heating block for 20 h. Upon completion, the reaction mixture was cooled to room temperature and filtered through a $SiO_2$ plug (0.5 x 3 cm), eluting with 1:1 EtOAc/hexanes (6 mL). The solvent was removed by rotary evaporation, and the resulting residue was purified by flash chromatography (9:1 hexanes/EtOAc eluent) to afford indole **6kb** (41.0 mg, 97% yield, $R_f$ = 0.39 in 9:1 hexanes/EtOAc) as a colorless oil.

**Data for Indole 6kb.**
**$^{1}$H NMR** (400 MHz, $CDCl_3$): δ 7.99 (d, $J$ = 9.0 Hz, 1H), 7.47 – 7.43 (m, 2H), 7.35 (app. t, $J$ = 7.3 Hz, 2H), 7.29 (t, $J$ = 7.3 Hz, 1H), 6.93 (d, $J$ = 2.5 Hz, 1H), 6.84 (dd, $J$ = 9.0, 2.5 Hz, 1H), 6.29 (s, 1H), 5.34 (s, 1H), 5.13 (s, 1H), 3.85 (s, 3H), 3.16 (t, $J$ = 7.8 Hz, 2H), 2.91 (t, $J$ = 7.8 Hz, 2H), 1.63 (s, 9H).
**$^{13}$C NMR** (100 MHz, $CDCl_3$): δ 155.9, 150.5, 147.8, 142.4, 141.3, 131.4, 130.2, 128.5, 127.6, 126.3, 116.5, 112.9, 111.8, 107.3, 102.6, 83.7, 55.8, 34.6, 29.3, 28.4.
**IR** (film): 2926, 2852, 1729, 1616, 1478, 1449, 1370, 1314, 1218, 1163, 1121, 1088, 1036, 895, 849, 778, 702 $cm^{-1}$.
**HRMS** ($ESI^+$) *m/z* calc'd for $(M + H)^+$ $[C_{24}H_{27}NO_3 + H]^+$: 378.2064, found 378.2066.

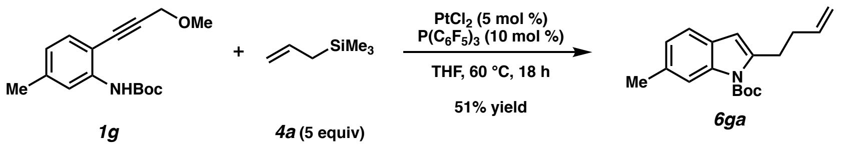


**Indole 6ga.** To a solution of $P(C_6F_5)_3$ (10.6 mg, 0.0200 mmol) in THF (1.00 mL) at 23 °C was added $PtCl_2$ (2.7 mg, 0.0100 mmol), and the resulting solution was stirred for 15 min. This catalyst solution was then added to a solution of alkyne **1g** (55.1 mg, 0.200 mmol) and allyltrimethylsilane (**4a**, 159 μL, 1.00 mmol) in THF (1.00 mL, 0.1 M in substrate final concentration) at 23 °C. The resulting mixture was stirred in a 60 °C heating block for 18 h. Upon completion, the reaction mixture was cooled to room temperature and filtered through a $SiO_2$ plug (0.5 x 3 cm), eluting with 1:1 EtOAc/hexanes (8 mL). The solvent was removed by rotary evaporation, and the resulting residue was purified by flash chromatography (19:1 hexanes/EtOAc eluent) to afford indole **6ga** (28.9 mg, 51% yield, $R_f$ = 0.58 in 19:1 hexanes/EtOAc) as a colorless oil.

**Data for Indole 6ga.**
**$^{1}$H NMR** (600 MHz, $CDCl_3$): δ 7.98 (s, 1H), 7.33 (d, $J$ = 7.9 Hz, 1H), 7.02 (d, $J$ = 7.9 Hz, 1H), 6.31 (s, 1H), 5.92 (ddt, $J$ = 16.8, 10.3, 6.7 Hz, 1H), 5.09 (app. dq, $J$ = 17.2, 1.5 Hz, 1H), 5.02 (app. dq, $J$ = 10.7, 1.3 Hz, 1H), 3.07 (t, $J$ = 7.7 Hz, 2H), 2.48-2.42 (comp. m, 2H), 2.46 (s, 3H), 1.68 (s, 9H).
**$^{13}$C NMR** (100 MHz, $CDCl_3$): δ 150.8, 140.8, 138.0, 137.3, 133.3, 127.1, 124.1, 119.4, 116.1, 115.2, 107.4, 83.7, 33.2, 29.8, 28.4, 22.2.
**IR** (film): 2977, 2850, 1730, 1369, 1330, 1150, 1126, 1079, 820, 769 $cm^{-1}$.
**HRMS** ($ESI^+$): *m/z* calc'd for $(M + Na)^+$ $[C_{18}H_{23}NO_2 + Na]^+$: 308.1621, found 308.1613.

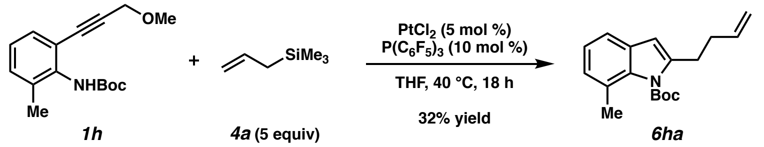

**Indole 6ha.** To a solution of $P(C_6F_5)_3$ (10.6 mg, 0.0200 mmol) in THF (1.00 mL) at 23 °C was added $PtCl_2$ (2.7 mg, 0.0100 mmol), and the resulting solution was stirred for 15 min. This catalyst solution was then added to a solution of alkyne **1h** (55.1 mg, 0.200 mmol) and allyltrimethylsilane (**4a**, 159 µL, 1.00 mmol) in THF (1.00 mL, 0.1 M in substrate final concentration) at 23 °C. The resulting mixture was stirred in a 40 °C heating block for 18 h. Upon completion, the reaction mixture was cooled to room temperature and filtered through a $SiO_2$ plug (0.5 x 3 cm), eluting with 1:1 EtOAc/hexanes (8 mL). The solvent was removed by rotary evaporation, and the resulting residue was purified by flash chromatography (19:1 hexanes/EtOAc eluent) to afford indole **6ha** (18.2 mg, 32% yield, $R_f$ = 0.41 in 19:1 hexanes/EtOAc) as a colorless oil.

**Data for Indole 6ha.**
**$^1$H NMR** (400 MHz, $CDCl_3$): δ 7.31 (d, *J* = 7.3 Hz, 1H), 7.09 (app. t, *J* = 7.5 Hz, 1H), 7.01 (d, *J* = 7.5 Hz, 1H), 6.31 (s, 1H), 5.93 (ddt, *J* = 16.7, 10.3, 6.6 Hz, 1H), 5.11 (app. dq, *J* = 17.0, 1.7 Hz, 1H), 5.04 (app. dq, *J* = 10.7, 1.6 Hz, 1H), 2.95 (t, *J* = 7.5 Hz, 2H), 2.54-2.43 (comp. m, 2H), 2.46 (s, 3H), 1.65 (s, 9H).
**$^{13}$C NMR** (100 MHz, $CDCl_3$): δ 150.9, 140.9, 137.8, 136.0, 130.2, 126.1, 124.1, 122.6, 117.7, 115.3, 105.7, 84.1, 33.1, 28.6, 28.1, 20.9.
**IR** (film): 2977, 2931, 1736, 1519, 1487, 1316, 1155, 1087, 958, 742 $cm^{-1}$.
**HRMS** ($ESI^+$): $(M + Na)^+$ $[C_{18}H_{23}NO_2 + Na]^+$: 308.1621, found 308.1611.

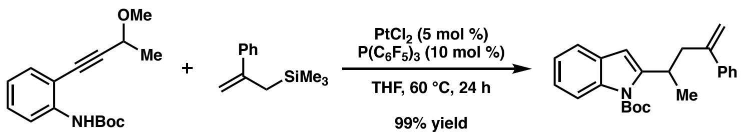

*1l* *4b* (5 equiv) *6lb*

**Indole 6lb.** To a solution of $P(C_6F_5)_3$ (5.7 mg, 0.0107 mmol) in THF (0.55 mL) at 23 °C was added $PtCl_2$ (1.4 mg, 0.00535 mmol), and the resulting solution was stirred for 15 min. This catalyst solution was then added to a solution of alkyne **1l** (29.4 mg, 0.107 mmol) and allylic trimethylsilane **4b** (102 mg, 0.535 mmol) in THF (0.55 mL, 0.1 M in substrate final concentration) at 23 °C. The resulting mixture was stirred in a 60 °C heating block for 24 h. Upon completion, the reaction mixture was cooled to room temperature and filtered through a $SiO_2$ plug (0.5 x 3 cm), eluting with 1:1 EtOAc/hexanes (5 mL). The solvent was removed by rotary evaporation, and the resulting residue was purified by flash chromatography (9:1 hexanes/EtOAc eluent) to afford indole **6lb** (38.7 mg, 99% yield, $R_f$ = 0.37 in 9:1 hexanes/EtOAc) as a colorless oil.

**Data for Indole 6lb.**
**$^1$H NMR** (400 MHz, $CDCl_3$): δ 8.08 (d, *J* = 8.0 Hz, 1H), 7.47 (d, *J* = 7.3 Hz, 1H), 7.43 – 7.39 (d, *J* = 7.4 Hz, 2H), 7.31 (app. t, *J* = 7.3 Hz, 2H), 7.27 – 7.24 (m, 1H), 7.23 (app. t, *J* = 7.7 Hz, 1H), 7.18 (app. t, *J* = 7.3 Hz, 1H), 6.44 (s, 1H), 5.31 (s, 1H), 5.14 (s, 1H), 3.80 – 3.70 (m, 1H), 3.18 (dd, *J* = 14.1, 4.9 Hz, 1H), 2.64 (dd, *J* = 14.1 Hz, 9.5 Hz, 1H), 1.52 (s, 9H), 1.21 (d, *J* = 6.7 Hz, 3H).
**$^{13}$C NMR** (100 MHz, $CDCl_3$): δ 150.5, 147.6, 147.0, 141.2, 136.8, 129.4, 128.4, 127.5, 126.6, 123.4, 122.7, 119.9, 115.6, 114.7, 105.5, 83.8, 42.5, 31.0, 28.1, 21.0.
**IR** (film): 2977, 2928, 1731, 1489, 1455, 1328, 1249, 1158, 1249, 1158, 1113, 1078, 779, 746, 699 $cm^{-1}$.
**HRMS** ($ESI^+$) *m/z* calc'd for $(M + H)^+$ $[C_{24}H_{27}NO_2 + H]^+$: 362.2115, found 362.2115.

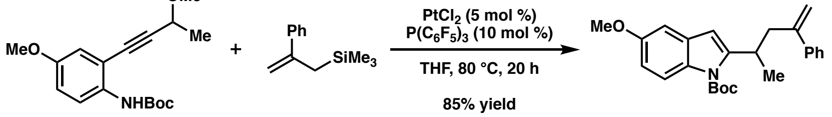

*1m* *4b* (5 equiv) *6mb*

**Indole 6mb.** To a solution of $P(C_6F_5)_3$ (5.3 mg, 0.00989 mmol) in THF (0.50 mL) at 23 °C was added $PtCl_2$ (1.3 mg, 0.00495 mmol), and the resulting solution was stirred for 15 min. This catalyst solution was then added to a solution of alkyne **1m** (30.2 mg, 0.0989 mmol) and allylic trimethylsilane **4b** (94.2 mg, 0.495 mmol) in THF (0.50 mL, 0.1 M in substrate final concentration) at 23 °C. The resulting mixture was stirred in an 80 °C heating block for 20 h. Upon completion, the reaction mixture was cooled to room temperature and filtered through a $SiO_2$ plug (0.5 x 3 cm), eluting with 1:1 EtOAc/hexanes (5 mL). The solvent was removed by rotary evaporation, and the resulting residue was purified by flash chromatography (9:1 hexanes/EtOAc eluent) to afford indole **6mb** (32.9 mg, 85% yield, $R_f$ = 0.34 in 9:1 hexanes/EtOAc) as a colorless oil.

**Data for Indole 6mb.**
**$^1$H NMR** (400 MHz, $CDCl_3$): δ 7.97 (d, $J$ = 9.0 Hz, 1H), 7.42 – 7.38 (m, 2H), 7.31 (app. t, $J$ = 7.2 Hz, 2H), 7.25 (t, $J$ = 7.2 Hz, 1H), 6.94 (d, $J$ = 2.6 Hz, 1H), 6.83 (dd, $J$ = 9.0, 2.6 Hz, 1H), 6.36 (s, 1H), 5.31 (s, 1H), 5.13 (s, 1H), 3.84 (s, 3H), 3.78 – 3.68 (m, 1H), 3.15 (dd, $J$ = 14.1, 4.9 Hz, 1H), 2.63 (dd, $J$ = 14.1, 9.5 Hz, 1H), 1.51 (s, 9H), 1.20 (d, $J$ = 6.7 Hz, 3H).
**$^{13}$C NMR** (100 MHz, $CDCl_3$): δ 155.9, 150.5, 148.4, 147.0, 141.3, 131.5, 130.2, 128.4, 127.5, 126.6, 116.5, 114.7, 111.8, 105.4, 102.7, 83.6, 55.8, 42.5, 31.1, 28.1, 21.0.
**IR** (film): 2926, 2852, 2360, 1728, 1617, 1477, 1449, 1371, 1335, 1313, 1218, 1164, 1120, 1078, 849, 779, 700 $cm^{-1}$.
**HRMS** ($ESI^+$) $m/z$ calc'd for $(M + H)^+$ $[C_{25}H_{29}NO_3 + H]^+$: 392.2220, found 392.2220.

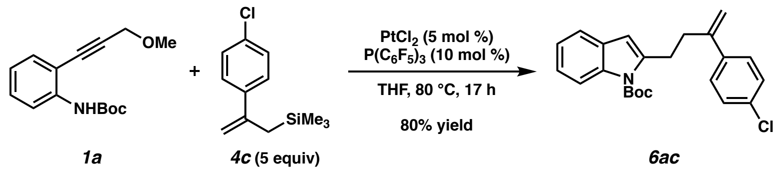


**Indole 6ac.** To a solution of $P(C_6F_5)_3$ (8.8 mg, 0.0166 mmol) in THF (0.85 mL) at 23 °C was added $PtCl_2$ (2.2 mg, 0.00830 mmol), and the resulting solution was stirred for 15 min. This catalyst solution was then added to a solution of alkyne **1a** (43.5 mg, 0.166 mmol) and allylic trimethylsilane **4c** (187 mg, 0.830 mmol) in THF (0.85 mL, 0.1 M in substrate final concentration) at 23 °C. The resulting mixture was stirred in an 80 °C heating block for 17 h. Upon completion, the reaction mixture was cooled to room temperature and filtered through a $SiO_2$ plug (0.5 x 3 cm), eluting with 1:1 EtOAc/hexanes (5 mL). The solvent was removed by rotary evaporation, and the resulting residue was purified by flash chromatography (9:1 hexanes/EtOAc eluent) to afford indole **6ac** (51.0 mg, 80% yield, $R_f$ = 0.51 in 9:1 hexanes/EtOAc) as a colorless oil.

**Data for Indole 6ac.**
**$^1$H NMR** (400 MHz, $CDCl_3$): δ 8.08 (d, $J$ = 8.0 Hz, 1H), 7.45 (d, $J$ = 7.4 Hz, 1H), 7.38 (d, $J$ = 8.5 Hz, 2H), 7.30 (d, $J$ = 8.5 Hz, 2H), 7.24 (app. t, $J$ = 7.7 Hz, 1H), 7.19 (app. t, $J$ = 7.4 Hz, 1H), 6.34 (s, 1H), 5.32 (s, 1H), 5.14 (s, 1H), 3.16 (t, $J$ = 7.8 Hz, 2H), 2.88 (t, $J$ = 7.8 Hz, 2H), 1.65 (s, 9H).
**$^{13}$C NMR** (100 MHz, $CDCl_3$): δ 150.6, 146.7, 141.4, 139.6, 136.7, 133.4, 129.3, 128.6, 127.6, 123.5, 122.8, 119.9, 115.7, 113.5, 107.5, 83.9, 34.6, 29.2, 28.4.
**IR** (film): 2926, 1734, 1559, 1541, 1507 1490, 1457, 1369, 1327, 1158, 1116, 1087 $cm^{-1}$.
**HRMS** ($ESI^+$) $m/z$ calc'd for $(M + H)^+$ $[C_{23}H_{24}ClNO_2 + H]^+$: 382.1568, found 382.1568.

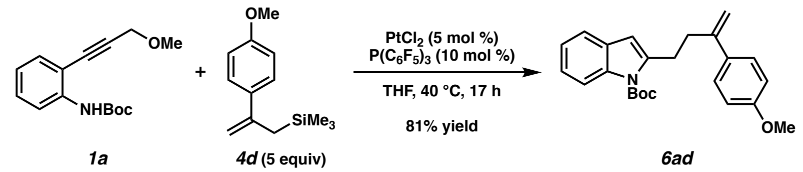

**Indole 6ad.** To a solution of $P(C_6F_5)_3$ (8.4 mg, 0.0158 mmol) in THF (0.80 mL) at 23 °C was added $PtCl_2$ (2.1 mg, 0.00790 mmol), and the resulting solution was stirred for 15 min. This catalyst solution was then added to a solution of alkyne **1a** (41.3 mg, 0.158 mmol) and allylic trimethylsilane **4d** (174 mg, 0.790 mmol) in THF (0.80 mL, 0.1 M in substrate final concentration) at 23 °C. The resulting mixture was stirred in a 40 °C heating block for 17 h. Upon completion, the reaction mixture was cooled to room temperature and filtered through a $SiO_2$ plug (0.5 x 3 cm), eluting with 1:1 EtOAc/hexanes (5 mL). The solvent was removed by rotary evaporation, and the resulting residue was purified by flash chromatography (9:1 hexanes/EtOAc eluent) to afford indole **6ad** (48.6 mg, 81% yield, $R_f$ = 0.44 in 9:1 hexanes/EtOAc) as a colorless oil.

**Data for Indole 6ad.**
$^1$**H NMR** (400 MHz, $CDCl_3$): δ 8.11 (d, *J* = 8.0 Hz, 1H), 7.45 (d, *J* = 7.4 Hz, 1H), 7.40 (d, *J* = 8.6 Hz, 2H), 7.24 (app. t, *J* = 7.7 Hz, 1H), 7.19 (app. t, *J* = 7.4 Hz, 1H), 6.88 (d, *J* = 8.6 Hz, 2H), 6.35 (s, 1H), 5.27 (s, 1H), 5.05 (s, 1H), 3.82 (s, 3H), 3.18 (t, *J* = 7.8 Hz, 1H), 2.89 (t, *J* = 7.8 Hz, 1H), 1.65 (s, 9H).
$^{13}$**C NMR** (100 MHz, $CDCl_3$): δ 159.2, 150.6, 147.1, 141.8, 136.8, 133.7, 129.4, 127.3, 123.4, 122.7, 119.8, 115.7, 113.8, 111.3, 107.4, 83.8, 55.4, 34.7, 29.3, 28.3.
**IR** (film): 2933, 1731, 1608, 1512, 1455, 1369, 1327, 1303, 1248, 1158, 1116, 1086, 1034, 835, 808, 769, 747 cm$^{-1}$.
**HRMS** (ESI$^+$) *m/z* calc'd for $(M + H)^+$ $[C_{24}H_{27}NO_3 + H]^+$: 378.2064, found 378.2064.

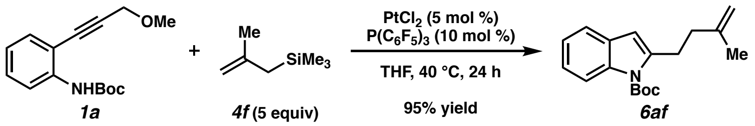

**Indole 6af.** To a solution of $P(C_6F_5)_3$ (6.4 mg, 0.0121 mmol) in THF (0.60 mL) at 23 °C was added $PtCl_2$ (1.6 mg, 0.00605 mmol), and the resulting solution was stirred for 15 min. This catalyst solution was then added to a solution of alkyne **1a** (31.6 mg, 0.121 mmol) and allylic trimethylsilane **4f** (77.6 mg, 0.605 mmol) in THF (0.60 mL, 0.1 M in substrate final concentration) at 23 °C. The resulting mixture was stirred in a 40 °C heating block for 24 h. Upon completion, the reaction mixture was cooled to room temperature and filtered through a $SiO_2$ plug (0.5 x 3 cm), eluting with 1:1 EtOAc/hexanes (5 mL). The solvent was removed by rotary evaporation, and the resulting residue was purified by flash chromatography (19:1 hexanes/EtOAc eluent) to afford indole **6af** (32.7 mg, 95% yield, $R_f$ = 0.37 in 19:1 hexanes/EtOAc) as a colorless oil.

**Data for Indole 6af.**
$^1$**H NMR** (400 MHz, $CDCl_3$): δ 8.12 (d, *J* = 8.0 Hz, 1H), 7.46 (d, *J* = 7.3 Hz, 1H), 7.24 (app. t, *J* = 7.7 Hz, 1H), 7.19 (app. t, *J* = 7.3 Hz, 1H), 6.38 (s, 1 H), 4.80 (s, 1H), 4.79 (s, 1H), 3.17 (t, *J* = 8.0 Hz, 2H), 2.43 (t, *J* = 8.0 Hz, 2H), 1.82 (s, 3H), 1.70 (s, 9H).
$^{13}$**C NMR** (100 MHz, $CDCl_3$): δ 150.7, 145.2, 142.0, 136.8, 129.4, 123.4, 122.7, 119.8, 115.7, 110.4, 107.1, 83.8, 37.0, 28.5, 28.4, 22.9.
**IR** (film): 2977, 2932, 1732, 1475, 1370, 1326, 1254, 1215, 1159, 1116, 1088, 804, 769, 746 cm$^{-1}$.
**HRMS** (ESI$^+$) *m/z* calc'd for $(M + Na)^+$ $[C_{18}H_{23}NO_2 + Na]^+$: 308.1621, found 308.1621.

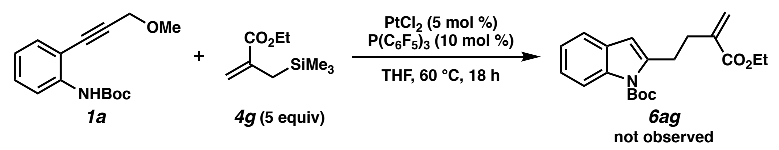


**Indole 6ag.** To a solution of $P(C_6F_5)_3$ (2.7 mg, 0.00501 mmol) in THF (0.25 mL) at 23 °C was added $PtCl_2$ (0.7 mg, 0.00251 mmol), and resulting solution was stirred for 15 min. This catalyst solution was then added to a solution of alkyne **1a** (13.1 mg, 0.0501 mmol) and allylic trimethylsilane **4g** (46.8 mg, 0.251 mmol) in THF (0.25 mL, 0.1 M in substrate final concentration) at 23 °C. The resulting mixture was heated in a 60 °C heating block and stirred for 18 h. The resulting reaction mixture was cooled to room temperature and filtered through a $SiO_2$ plug (0.5 x 3 cm), eluting with 1:1 hexanes/EtOAc (4 mL). The solvent was removed by rotary evaporation and the resulting residue was analyzed by $^1$H NMR. Product **6ag** was not observed.

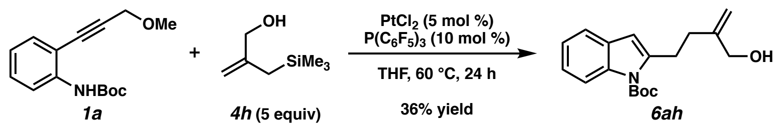


**Indole 6ah.** To a solution of $P(C_6F_5)_3$ (6.7 mg, 0.0126 mmol) in THF (0.65 mL) at 23 °C was added $PtCl_2$ (1.7 mg, 0.00630 mmol), and the resulting solution was stirred for 15 min. This catalyst solution was then added to a solution of alkyne **1a** (32.8 mg, 0.126 mmol) and allylic trimethylsilane **4h** (90.9 mg, 0.630 mmol) in THF (0.65 mL, 0.1 M in substrate final concentration) at 23 °C. The resulting mixture was stirred in a 60 °C heating block for 24 h. Upon completion, the reaction mixture was cooled to room temperature and filtered through a $SiO_2$ plug (0.5 x 3 cm), eluting with 1:1 EtOAc/hexanes (5 mL). The solvent was removed by rotary evaporation, and the resulting residue was purified by flash chromatography (1:1 hexanes/EtOAc eluent) to afford indole **6ah** (13.7 mg, 36% yield, $R_f$ = 0.56 in 1:1 hexanes/EtOAc) as a colorless oil.

**Data for Indole 6ah.**
**$^1$H NMR** (400 MHz, $CDCl_3$): δ 8.06 (d, $J$ = 7.7 Hz, 1H), 7.45 (d, $J$ = 7.3 Hz, 1H), 7.23 (app. t, $J$ = 7.3 Hz, 1H), 7.19 (app. t, $J$ = 7.3 Hz, 1H), 6.38 (s, 1H), 5.08 (s, 1H), 4.96 (s, 1H), 4.16 (d, $J$ = 5.2 Hz, 2H), 3.19 (t, $J$ = 7.9 Hz, 2H), 2.48 (t, $J$ = 7.9 Hz, 2H), 1.78 (t, $J$ = 5.2 Hz, 1H), 1.69 (s, 9H).
**$^{13}$C NMR** (100 MHz, $CDCl_3$): δ 150.8, 148.3, 141.8, 136.6, 129.4, 123.5, 122.8, 119.9, 115.7, 110.4, 107.5, 84.1, 66.2, 32.6, 28.6, 28.4.
**IR** (film): 3419, 2927, 2360, 2342, 1732, 1568, 1455, 1370, 1327, 1255, 1214, 1158, 1116, 1088, 898, 769, 747 cm$^{-1}$.
**HRMS** (ESI$^+$) *m/z* calc'd for $(M + H)^+$ $[C_{18}H_{23}NO_3 + H]^+$: 302.1751, found 302.1752.

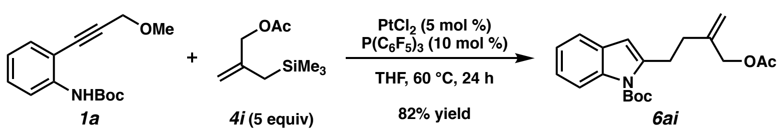


**Indole 6ai.** To a solution of $P(C_6F_5)_3$ (9.7 mg, 0.0182 mmol) in THF (0.90 mL) at 23 °C was added $PtCl_2$ (2.4 mg, 0.00910 mmol), and the resulting solution was stirred for 15 min. This catalyst solution was then added to a solution of alkyne **1a** (47.5 mg, 0.182 mmol) and allylic trimethylsilane **4i** (170 mg, 0.910 mmol) in THF (0.90 mL, 0.1 M in substrate final concentration) at 23 °C. The resulting mixture was stirred in a

60 °C heating block for 24 h. Upon completion, the reaction mixture was cooled to room temperature and filtered through a $SiO_2$ plug (0.5 x 3 cm), eluting with 1:1 EtOAc/hexanes (5 mL). The solvent was removed by rotary evaporation, and the resulting residue was purified by flash chromatography (9:1 hexanes/EtOAc eluent) to afford indole **6ai** (51.0 mg, 82% yield, $R_f$ = 0.32 in 9:1 hexanes/EtOAc) as a colorless oil.

**Data for Indole 6ai.**
**$^1$H NMR** (500 MHz, $CDCl_3$): δ 8.11 (d, $J$ = 8.0 Hz, 1H), 7.48 (d, $J$ = 7.4 Hz, 1H), 7.27 (app. t, $J$ = 7.7 Hz, 1H), 7.22 (app. t, $J$ = 7.3 Hz, 1H), 6.40 (s, 1H), 5.14 (s, 1H), 5.07 (s, 1H), 4.62 (s, 2H), 3.22 (t, $J$ = 7.9 Hz, 2H), 2.50 (t, $J$ = 7.9 Hz, 2H), 2.12 (s, 3H), 1.71 (s, 9H).
**$^{13}$C NMR** (125 MHz, $CDCl_3$): δ 170.9, 150.6, 143.2, 141.4, 136.7, 129.4, 123.5, 122.8, 119.9, 115.7, 113.1, 107.5, 83.9, 67.2, 32.5, 28.4, 28.3, 21.1.
**IR** (film): 2925, 2854, 2359, 1732, 1652, 1455, 1370, 1328, 1228, 1159, 1116, 1088, 1026, 769, 746 cm$^{-1}$.
**HRMS** (ESI$^+$) *m/z* calc'd for $(M + H)^+$ $[C_{20}H_{25}NO_4 + H]^+$: 344.1856, found 344.1857.

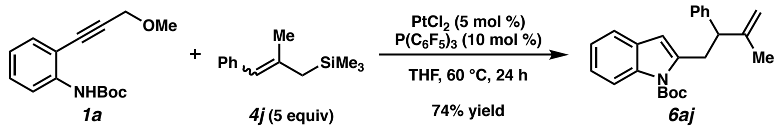


**Indole 6aj.** To a solution of $P(C_6F_5)_3$ (6.2 mg, 0.0116 mmol) in THF (0.60 mL) at 23 °C was added $PtCl_2$ (1.5 mg, 0.00580 mmol), and the resulting solution was stirred for 15 min. This catalyst solution was then added to a solution of alkyne **1a** (30.4 mg, 0.116 mmol) and allylic trimethylsilane **4j** (119 mg, 0.580 mmol) in THF (0.60 mL, 0.1 M in substrate final concentration) at 23 °C. The resulting mixture was stirred in a 60 °C heating block for 24 h. Upon completion, the reaction mixture was cooled to room temperature and filtered through a $SiO_2$ plug (0.5 x 3 cm), eluting with 1:1 EtOAc/hexanes (5 mL). The solvent was removed by rotary evaporation, and the resulting residue was purified by flash chromatography (19:1 hexanes/EtOAc eluent) to afford indole **6aj** (31.1 mg, 74% yield, $R_f$ = 0.32 in 19:1 hexanes/EtOAc) as a colorless oil.

**Data for Indole 6aj.**
**$^1$H NMR** (400 MHz, $CDCl_3$): δ 8.08 (d, $J$ = 8.2 Hz, 1H), 7.35 (d, $J$ = 7.3 Hz, 1H), 7.23 (d, $J$ = 7.4 Hz, 2H), 7.21 – 7.16 (comp. m, 4H), 7.14 (app. t, $J$ = 7.3 Hz, 1H), 6.10 (s, 1H), 4.97 (s, 1H), 4.88 (s, 1H), 3.72 (app. t, $J$ = 7.4 Hz, 1H), 3.62 (dd, $J$ = 15.5, 6.8 Hz, 1H), 3.40 (dd, $J$ = 15.5, 8.0 Hz, 1H), 1.66 (s, 9H), 1.65 (s, 3H).
**$^{13}$C NMR** (100 MHz, $CDCl_3$): δ 150.8, 147.4, 142.9, 140.0, 136.7, 129.3, 128.4, 128.0, 126.5, 123.3, 122.6, 119.9, 115.6, 111.2, 108.9, 83.9, 51.8, 34.1, 28.4, 21.6.
**IR** (film): 2976, 2927, 2360, 2341, 1731, 1492, 1474, 1454, 1327, 1255, 1215, 1158, 1116, 1089, 890, 769, 746 cm$^{-1}$.
**HRMS** (ESI$^+$) *m/z* calc'd for $(M + H)^+$ $[C_{24}H_{27}NO_2 + H]^+$: 362.2115, found 362.2115.

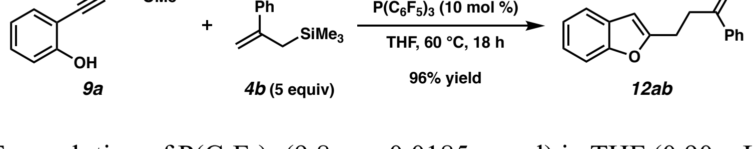


**Benzofuran 12ab.** To a solution of $P(C_6F_5)_3$ (9.8 mg, 0.0185 mmol) in THF (0.90 mL) at 23 °C was added $PtCl_2$ (2.5 mg, 0.00925 mmol), and the resulting solution was stirred for 15 min. This catalyst solution was then added to a solution of alkyne **9a** (30.0 mg, 0.185 mmol) and allylic trimethylsilane **4b** (176 mg, 0.925 mmol) in THF (0.90 mL, 0.1 M in substrate final concentration) at 23 °C. The resulting mixture was stirred in a 60 °C heating block for 18 h. Upon completion, the reaction mixture was cooled to room temperature

and filtered through a $SiO_2$ plug (0.5 x 3 cm), eluting with 1:1 EtOAc/hexanes (5 mL). The solvent was removed by rotary evaporation, and the resulting residue was purified by flash chromatography (19:1 hexanes/EtOAc eluent) to afford benzofuran **12ab** (44.3 mg, 96% yield, $R_f$ = 0.51 in 19:1 hexanes/EtOAc) as a colorless oil.

**Data for Benzofuran 12ab.**
**$^1$H NMR** (400 MHz, $CDCl_3$): δ 7.48 (d, *J* = 7.3 Hz, 1H), 7.45 (d, *J* = 7.3 Hz, 2H), 7.42 (d, *J* = 7.7 Hz, 1H), 7.36 (app. t, *J* = 7.3 Hz, 2H), 7.30 (t, *J* = 7.3 Hz, 1H), 7.22 (app. t, *J* = 7.3 Hz, 1H), 7.19 (app. t, *J* = 7.3 Hz, 1H), 6.38 (s, 1H), 5.33 (s, 1H), 5.13 (s, 1H), 3.01 – 2.90 (comp. m, 4H).
**$^{13}$C NMR** (100 MHz, $CDCl_3$): δ 158.7, 154.8, 147.2, 140.8, 129.0, 128.6, 127.7, 126.3, 123.3, 122.5, 120.4, 113.3, 110.9, 102.3, 33.6, 27.7.
**IR** (film): 2924, 2852, 2360, 2342, 2489, 1455, 1252, 897, 778, 749, 701 cm$^{-1}$.
**HRMS** (ESI$^+$) *m/z* calc'd for $(M + H)^+$ $[C_{18}H_{16}O + H]^+$: 249.1274, found 249.1275.

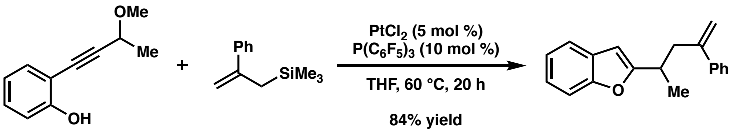


*9d* *4b* (5 equiv) *12db*

**Benzofuran 12db.** To a solution of $P(C_6F_5)_3$ (13.9 mg, 0.0262 mmol) in THF (1.30 mL) at 23 °C was added $PtCl_2$ (3.5 mg, 0.0131 mmol), and the resulting solution was stirred for 15 min. This catalyst solution was then added to a solution of alkyne **9d** (46.2 mg, 0.262 mmol) and allylic trimethylsilane **4b** (249 mg, 1.31 mmol) in THF (1.30 mL, 0.1 M in substrate final concentration) at 23 °C. The resulting mixture was stirred in a 60 °C heating block for 20 h. Upon completion, the reaction mixture was cooled to room temperature and filtered through a $SiO_2$ plug (0.5 x 3 cm), eluting with 1:1 EtOAc/hexanes (10 mL). The solvent was removed by rotary evaporation, and the resulting residue was purified by flash chromatography (9:1 hexanes/EtOAc eluent) to afford benzofuran **12db** (57.9 mg, 84% yield, $R_f$ = 0.54 in 19:1 hexanes/EtOAc) as a colorless oil.

**Data for Benzofuran 12db.**
**$^1$H NMR** (400 MHz, $CDCl_3$): δ 7.48 (d, *J* = 7.3 Hz, 1H), 7.46 – 7.41 (comp. m, 3H), 7.35 (app. t, *J* = 7.3 Hz, 2H), 7.29 (t, *J* = 7.3 Hz, 1H), 7.22 (app. t, *J* = 7.3 Hz, 1H), 7.18 (app. t, *J* = 7.3 Hz, 1H), 6.34 (s, 1H), 5.30 (d, *J* = 1.3 Hz, 1H), 5.08 (d, *J* = 1.0 Hz, 1H), 3.19 (ddd, *J* = 14.0, 5.9, 1.3 Hz, 1H), 3.05 (app. sextet, *J* = 6.8 Hz, 1H), 2.65 (ddd, *J* = 14.0, 8.7, 1.0 Hz, 1H), 1.29 (d, *J* = 6.8 Hz, 3H).
**$^{13}$C NMR** (100 MHz, $CDCl_3$): δ 163.3, 154.7, 146.3, 140.7, 128.9, 128.5, 127.6, 126.5, 123.3, 122.5, 120.4, 114.9, 110.9, 101.0, 41.6, 32.2, 18.4.
**IR** (film): 2966, 2926, 1628, 1599, 1454, 1254, 1168, 938, 899, 798, 778, 749, 701 cm$^{-1}$.
**HRMS** (ESI$^+$) *m/z* calc'd for $(M + H)^+$ $[C_{19}H_{18}O + H]^+$: 263.1430, found 263.1430.

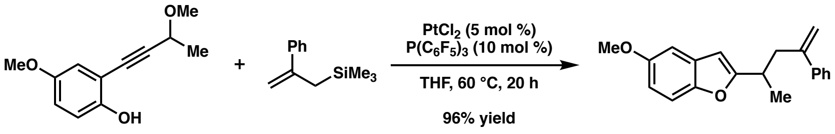


*9e* *4b* (5 equiv) *12eb*

**Benzofuran 12eb.** To a solution of $P(C_6F_5)_3$ (9.1 mg, 0.0171 mmol) in THF (0.85 mL) at 23 °C was added $PtCl_2$ (2.3 mg, 0.00855 mmol), and the resulting solution was stirred for 15 min. This catalyst solution was then added to a solution of alkyne **9e** (35.2 mg, 0.171 mmol) and allylic trimethylsilane **4b** (163 mg, 0.855

mmol) in THF (0.85 mL, 0.1 M in substrate final concentration) at 23 °C. The resulting mixture was stirred in a 60 °C heating block for 20 h. Upon completion, the reaction mixture was cooled to room temperature and filtered through $SiO_2$ plug (0.5 x 3 cm), eluting with 1:1 EtOAc/hexanes (8 mL). The solvent was removed by rotary evaporation, and the resulting residue was purified by flash chromatography (9:1 hexanes/EtOAc eluent) to afford benzofuran **12eb** (48.2 mg, 96% yield, $R_f$ = 0.47 in 19:1 hexanes/EtOAc) as a colorless oil.

**Data for Benzofuran 12eb.**
**$^1$H NMR** (400 MHz, $CDCl_3$): δ 7.45 – 7.41 (m, 2H), 7.34 (app. t, *J* = 7.2 Hz, 2H), 7.30 (d, *J* = 8.8 Hz, 1H), 7.29 (t, *J* = 7.2 Hz, 1H), 6.95 (d, *J* = 2.6 Hz, 1H), 6.81 (dd, *J* = 8.8, 2.6 Hz, 1H), 6.27 (s, 1H), 5.29 (d, *J* = 1.2 Hz, 1H), 5.07 (d, *J* = 0.9 Hz, 1H), 3.83 (s, 3H), 3.16 (ddd, *J* = 14.0, 5.9, 1.2 Hz, 1H), 3.02 (app. sextet, *J* = 6.9 Hz, 1H), 2.63 (ddd, *J* = 14.0, 8.7, 0.9 Hz, 1H), 1.27 (d, *J* = 6.9 Hz, 3H).
**$^{13}$C NMR** (100 MHz, $CDCl_3$): δ 164.2, 155.8, 149.6, 146.3, 140.7, 129.4, 128.5, 127.6, 126.5, 114.9, 111.6, 111.2, 103.3, 101.2, 56.1, 41.6, 32.2, 18.4.
**IR** (film): 2928, 2625, 1601, 1477, 1448, 1205, 1179, 1031, 836, 779, 702 $cm^{-1}$.
**HRMS** ($ESI^+$) *m/z* calc'd for $(M + H)^+$ $[C_{20}H_{20}O_2 + H]^+$: 293.1536, found 293.1536.

# Miscellaneous Experiments

## *(3+2) Cycloaddition/Tamao-Fleming Oxidation Sequence*

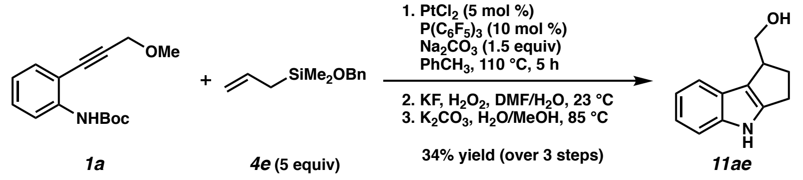


**Indole 11ae.** To a solution of $P(C_6F_5)_3$ (10.1 mg, 0.0190 mmol) in toluene (0.95 mL) at 23 °C was added $PtCl_2$ (2.5 mg, 0.00950 mmol), and the resulting solution was stirred for 15 min. The catalyst solution was then added to a prestirred solution of alkyne **1a** (49.6 mg, 0.190 mmol) and allylsilane **4e** (196 mg, 0.950 mmol) in toluene (0.95 mL, 0.1 M in substrate final concentration) at 23 °C. $Na_2CO_3$ (30.2 mg, 0.285 mmol) was then added, and the resulting mixture was stirred in a 110 °C heating block for 5 h. Upon completion, the reaction mixture was cooled to room temperature and filtered through a $SiO_2$ plug (0.5 x 3 cm), eluting with 1:1 EtOAc/hexanes (5 mL). The solvent was removed by rotary evaporation, and the resulting residue was purified by flash chromatography (9:1 hexanes/EtOAc eluent). The resulting residue was dissolved in DMF (0.38 mL). KF (44.2 mg, 0.760 mmol) and $H_2O_2$ (0.233 mL, 30% w/w solution in $H_2O$, 2.28 mmol) were added sequentially, and the resulting mixture was stirred at 23 °C for 23 h. Upon reaction completion, $H_2O$ (2 mL) and $Et_2O$ (5 mL) were added, and the phases were separated. The aqueous phase was extracted with $Et_2O$ (2 x 10 mL), and the combined organic phases were sequentially washed with $H_2O$ (20 mL), 10% aq. LiCl (10 mL) and brine (10 mL), and then dried over $MgSO_4$. The mixture was concentrated in vacuo, and the resulting residue was dissolved in a 3:1 $H_2O$/MeOH mixture (2.53 mL). $K_2CO_3$ (52.5 mg, 0.380 mmol) was added, and the resulting mixture was stirred in an 85 °C heating block for 2 h. Upon reaction completion, the reaction mixture was allowed to cool to room temperature, and MeOH was removed via rotary evaporation. EtOAc (5 mL) was added and the layers were separated. The aqueous phase was extracted with EtOAc (2 x 5 mL), and the combined organic phases were washed with brine (5 mL) and dried over $MgSO_4$. The mixture was concentrated in vacuo, and the resulting residue was purified by flash chromatography (2:1 hexanes/EtOAc eluent w/ 2% $Et_3N$) to afford indole **11ae** (12.1 mg, 34% yield, $R_f$ = 0.21 in 2:1 hexanes/EtOAc w/ 2% $Et_3N$) as a colorless oil. Decomposition of NMR samples was best prevented by briefly passing argon over the sample solution.

**Data for Indole 11ae.**
**$^1$H NMR** (400 MHz, $CDCl_3$): δ 7.93 (br. s, 1H), 7.51 (d, $J$ = 8.2 Hz, 1H), 7.32 (d, $J$ = 7.3 Hz, 1H), 7.15 – 7.06 (comp. m, 2H), 3.90 (app. dd, $J$ = 10.5, 5.4 Hz, 1H), 3.80 (app. dd, $J$ = 10.5, 5.4 Hz, 1H), 3.56 – 3.48 (m, 1H), 3.14 – 3.06 (br. m, 1H), 3.01 – 2.91 (m, 1H), 2.89 – 2.79 (m, 1H), 2.70 (app. dtd, $J$ = 12.3, 8.6, 5.5 Hz, 1H), 2.35 (app. ddt, $J$ = 12.3, 9.1, 4.7 Hz, 1H).
**$^{13}$C NMR** (100 MHz, $C_6D_6$): δ 152.1, 141.6, 125.3, 121.0, 120.1, 119.2, 111.7, 66.8, 42.2, 32.4, 25.2.
**IR** (film): 3468, 3392, 2951, 2876, 2360.
**HRMS** ($ESI^+$): *m/z* calc'd for $(M + H)^+$ $[C_{12}H_{13}NO + H]^+$ 188.1070, found 188.1066.

## *Ene-type Reactivity with Methallyl Silane*

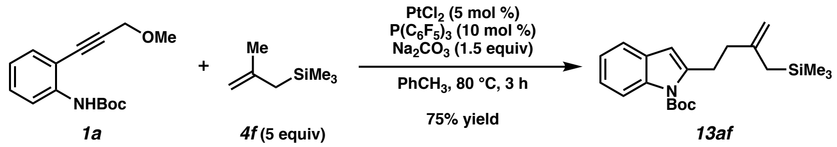


**Indole 13af.** To a solution of $P(C_6F_5)_3$ (9.6 mg, 0.0181 mmol) in toluene (0.90 mL) at 23 °C was added $PtCl_2$ (2.4 mg, 0.00905 mmol), and the resulting solution was stirred for 15 min. This catalyst solution was then added to a prestirred solution of alkyne **1a** (47.3 mg, 0.181 mmol) and allylic trimethylsilane **4f** (116 mg, 0.905 mmol) in toluene (0.90 mL, 0.1 M in substrate final concentration) at 23 °C. $Na_2CO_3$ (28.8 mg, 0.272 mmol) was then added, and the resulting mixture was stirred in an 80 °C heating block for 3 h. Upon completion, the reaction mixture was cooled to room temperature and filtered through a $SiO_2$ plug (0.5 x 3 cm), eluting with 1:1 EtOAc/hexanes (5 mL). The solvent was removed by rotary evaporation, and the resulting residue was purified by flash chromatography (9:1 hexanes/EtOAc eluent) to afford indole **13af** (48.3 mg, 75% yield, $R_f$ = 0.46 in 19:1 hexanes/EtOAc) as a pale yellow oil.

**Data for Indole 13af.**
**$^1$H NMR** (400 MHz, $CDCl_3$): δ 8.10 (d, *J* = 8.1 Hz, 1H), 7.46 (d, *J* = 7.3 Hz, 1H), 7.25 – 7.17 (comp. m, 2H), 6.37 (s, 1H), 4.70 (s, 1H), 4.61 (s, 1H), 3.17 (t, *J* = 8.1 Hz, 2H), 2.37 (t, *J* = 8.1 Hz, 2H), 1.69 (s, 9H), 1.62 (s, 2H), 0.05 (s, 9H);
**$^{13}$C NMR** (100 MHz, $CDCl_3$): δ 150.7, 147.0, 142.3, 136.8, 129.5, 123.4, 122.7, 119.8, 115.7, 107.4, 107.2, 83.8, 37.5, 28.8, 28.4, 27.4, -1.1;
**IR** (film): 2928, 2854, 1733, 1455, 1369, 1326, 1249, 1158, 1116, 1087, 833, 746 cm$^{-1}$;
**HRMS** (ESI$^+$): *m/z* calc'd for $(M + H)^+$ $[C_{21}H_{31}NO_2Si + H]^+$: 358.2197, found 358.2197.

*Allenylsilane Additions*

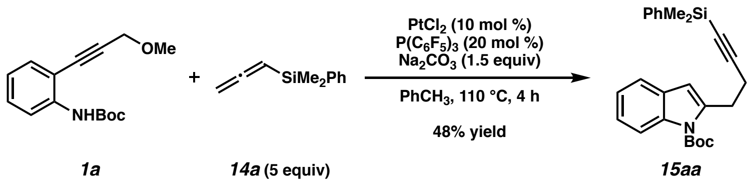


**Indole 15aa.** To a solution of $P(C_6F_5)_3$ (10.6 mg, 0.0200 mmol) in toluene (0.50 mL) at 23 °C was added $PtCl_2$ (2.7 mg, 0.0100 mmol), and the resulting solution was stirred for 15 min. This catalyst solution was then added to a prestirred solution of alkyne **1a** (26.1 mg, 0.100 mmol) and allenylsilane **14a** (87.2 mg, 0.500 mmol) in toluene (0.50 mL, 0.1 M in substrate final concentration) at 23 °C. $Na_2CO_3$ (15.9 mg, 0.150 mmol) was then added, and the resulting mixture was stirred in a 110 °C heating block for 4 h. Upon completion, as determined by TLC, the reaction mixture was cooled to room temperature and filtered through a $SiO_2$ plug (0.5 x 3 cm), eluting with 1:1 EtOAc/hexanes (5 mL). The solvent was removed by rotary evaporation, and the resulting residue was purified by flash chromatography (99:1 hexanes/$Et_2O$ eluent) to afford indole **15aa** (19.3 mg, 48% yield, $R_f$ = 0.25 in 99:1 hexanes/$Et_2O$) as a colorless oil.

**Data for Indole 15aa.**
**$^1$H NMR** (400 MHz, $CDCl_3$): δ 8.11 (d, *J* = 8.2 Hz, 1H), 7.59 (d, *J* = 7.0 Hz, 2H), 7.46 (d, *J* = 7.6 Hz, 1H), 7.39-7.28 (comp. m, 3H), 7.23 (app. t, *J* = 7.8 Hz, 1H), 7.20 (app. t, *J* = 7.2 Hz, 1H), 6.43 (s, 1H), 3.27 (t, *J* = 7.4 Hz, 2H), 2.69 (t, *J* = 7.4 Hz, 2H), 1.69 (s, 9H), 0.37 (s, 6H).
**$^{13}$C NMR** (100 MHz, $CDCl_3$): δ 150.6, 139.9, 137.6, 136.8, 133.8, 129.4, 129.3, 127.9, 123.7, 122.9, 120.1, 115.8, 108.5, 108.3, 84.1, 83.6, 29.8, 28.4, 20.4, -0.6.
**IR** (film): 2976, 2931, 2177, 1737, 1483, 1333, 1217, 1076, 880, 768 cm$^{-1}$.

**HRMS** ($ESI^+$): *m/z* calc'd for $(M + Na)^+$ $[C_{25}H_{29}NO_2Si + Na]^+$: 426.1860, found 426.1845.

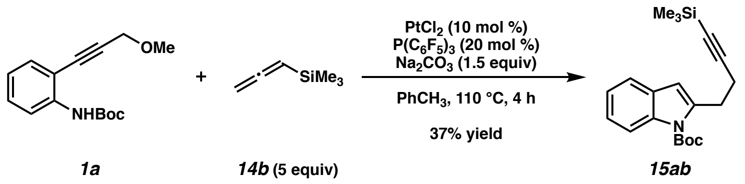


**Indole 15ab.** To a solution of $P(C_6F_5)_3$ (10.6 mg, 0.0200 mmol) in toluene (0.50 mL) at 23 °C was added $PtCl_2$ (2.7 mg, 0.0100 mmol), and the resulting solution was stirred for 15 min. This catalyst solution was then added to a prestirred solution of alkyne **1a** (26.1 mg, 0.100 mmol) and allenylsilane **14b** (56.1 mg, 0.500 mmol) in toluene (0.50 mL, 0.1 M in substrate final concentration) at 23 °C. $Na_2CO_3$ (15.9 mg, 0.150 mmol) was then added, and the resulting mixture was stirred in a 110 °C heating block for 4 h. Upon completion, as determined by TLC, the reaction mixture was cooled to room temperature and filtered through a $SiO_2$ plug (0.5 x 3 cm), eluting with 1:1 EtOAc/hexanes (5 mL). The solvent was removed by rotary evaporation, and the resulting residue was purified by flash chromatography (99:1 hexanes/$Et_2O$ eluent) to afford indole **15ab** (12.6 mg, 37% yield, $R_f$ = 0.26 in 99:1 hexanes/$Et_2O$) as a colorless oil.

**Data for Indole 15ab.**
**$^1$H NMR** (400 MHz, $CDCl_3$): δ 8.11 (d, *J* = 8.2 Hz, 1H), 7.46 (d, *J* = 7.6 Hz, 1H), 7.24 (app. t, *J* = 7.8 Hz, 1H), 7.19 (app. t, *J* = 7.4 Hz, 1H), 6.41 (s, 1H), 3.24 (t, *J* = 7.5 Hz, 2H), 2.62 (t, *J* = 7.5 Hz, 2H), 1.69 (s, 9H), 0.13 (s, 9H).
**$^{13}$C NMR** (100 MHz, $CDCl_3$): δ 150.6, 140.0, 136.8, 129.3, 123.7, 122.8, 120.0, 115.8, 108.3, 106.5, 85.6, 84.1, 29.9, 28.4, 20.3, 0.2.
**IR** (film): 2975, 2931, 2402, 2179, 1737, 1431, 1335, 1314, 1307, 1129, 1017, 871, 726 $cm^{-1}$.
**HRMS** ($ESI^+$): *m/z* calc'd for $(M + Na)^+$ $[C_{20}H_{27}NO_2Si + Na]^+$: 364.1703, found 364.1688.

*Test of Allyl Benzyl Ether as Alkene Reactant*

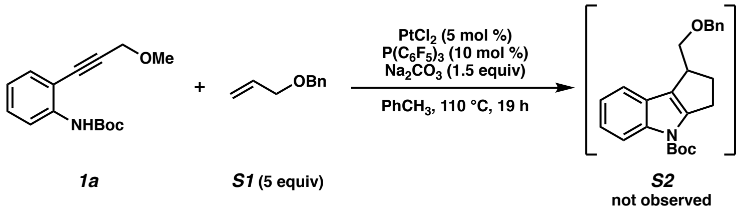


**Indole S2**. To a solution of $P(C_6F_5)_3$ (2.8 mg, 0.00528 mmol) in toluene (0.26 mL) at 23 °C was added $PtCl_2$ (0.7 mg, 0.00264 mmol), and the resulting solution was stirred for 15 min. The catalyst solution was then added to a prestirred solution of alkyne **1a** (13.8 mg, 0.0528 mmol) and allyl ether **S1** (39.1 mg, 0.264 mmol) in toluene (0.26 mL, 0.1 M in substrate final concentration) at 23 °C. $Na_2CO_3$ (8.4 mg, 0.0792 mmol) was then added, and the resulting mixture was stirred in a 110 °C heating block. After 19 h, the reaction mixture was cooled to room temperature and filtered through a $SiO_2$ plug (0.5 x 3 cm), eluting with 1:1 EtOAc/hexanes (4 mL). The solvent was removed by rotary evaporation, and the resulting crude residue was analyzed by $^1$H NMR, showing no formation of indole **S2**.

*Test of Si–H Reactant – Hydrosilylation Observation*

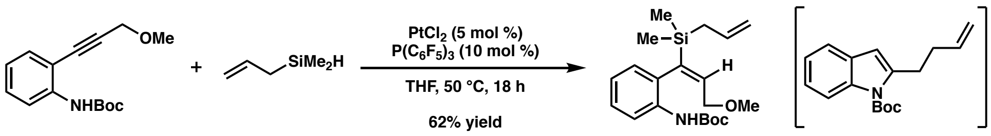


**Alkene 16ak.** To a solution of $P(C_6F_5)_3$ (10.2 mg, 0.0191 mmol) in THF (0.96 mL) at 23 °C was added $PtCl_2$ (2.5 mg, 0.00955 mmol), and the resulting solution was stirred for 15 min. The catalyst solution was then added to a prestirred solution of alkyne **1a** (50.0 mg, 0.191 mmol) and allylic silane **4k** (136 µL, 0.955 mmol) in THF (0.95 mL, 0.1 M in substrate final concentration) at 23 °C. The resulting mixture was stirred in a 50 °C heating block for 18 h. Upon completion, the reaction mixture was cooled to room temperature and filtered through a $SiO_2$ plug (0.5 x 3 cm), eluting with 1:1 EtOAc/hexanes (5 mL). The solvent was then removed by rotary evaporation, and the resulting residue was purified by flash chromatography (9:1 hexanes/EtOAc eluent) to afford (*E*)-alkene **16ak** (42.6 mg, 62% yield, $R_f$ = 0.42 in 9:1 hexanes/EtOAc) as a colorless oil.

**Data for (*E*)-Alkene 16ak.**
**$^1$H NMR** (400 MHz, $CDCl_3$): δ 7.97 (d, *J* = 7.8 Hz, 1H), 7.21 (app. t, *J* = 7.6 Hz, 1H), 6.99 (app. t, *J* = 7.6 Hz, 1H), 6.79 (d, *J* = 7.6 Hz, 1H), 6.45 (br. s, 1H), 6.33 (t, *J* = 5.6 Hz, 1H), 5.78 – 5.66 (m, 1H), 4.855 (d, *J* = 12.4 Hz, 1H), 4.851 (d, *J* = 15.7 Hz, 1H), 3.71 (app. dq, *J* = 12.8, 5.6 Hz, 2H), 3.25 (s, 3H), 1.58 (d, *J* = 7.8 Hz, 2H), 1.49 (s, 9H), 0.07 (s, 6H).
**$^{13}$C NMR** (100 MHz, $CDCl_3$): δ 152.9. 142.6, 142.1, 134.3, 134.2, 129.8, 127.6, 127.3, 122.8, 119.6, 114.1, 80.5, 70.6, 58.6, 28.5, 22.6, -3.7, -3.8.
**IR** (film): 3422, 2977, 2926, 2348, 2286, 1733 cm$^{-1}$.
**HRMS** (ESI$^+$): *m/z* calc'd for $(M + H)^+$ $[C_{20}H_{31}NO_3Si + H]^+$: 362.2146, found 362.2148.

The E geometry of alkene **16ak** was confirmed through 2D NOESY NMR analysis.

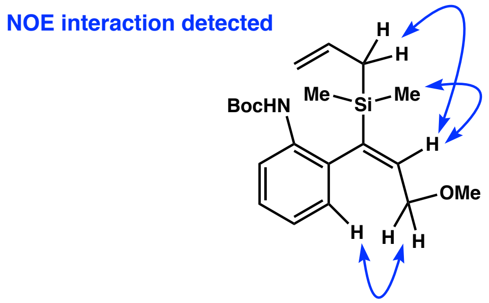


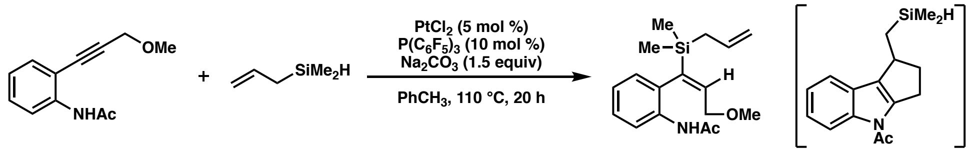


**Alkene 16bk.** To a solution of $P(C_6F_5)_3$ (13.1 mg, 0.0246 mmol) in THF (1.3 mL) at 23 °C was added $PtCl_2$ (3.3 mg, 0.0123 mmol), and the resulting solution was stirred for 15 min. The catalyst solution was then added to a prestirred solution of alkyne **1b** (50.0 mg, 0.246 mmol) and allylic silane **4k** (175 µL, 1.23 mmol) in THF (1.2 mL, 0.1 M in substrate final concentration) at 23 °C. $Na_2CO_3$ (39.1 mg, 0.369 mmol) was then added, and the resulting mixture was stirred in a 110 °C heating block for 20 h. Upon completion,

the reaction mixture was cooled to room temperature and filtered through a $SiO_2$ plug (0.5 x 3 cm), eluting with 1:1 EtOAc/hexanes (5 mL). The solvent was removed by rotary evaporation, and the resulting crude residue was analyzed by $^1$H NMR. A hydrosilylation product similar to compound **16ak** was observed (i.e., **16bk**, *N*-Ac instead of *N*-Boc).

*Tests of Allylboranes and Allylstannanes*

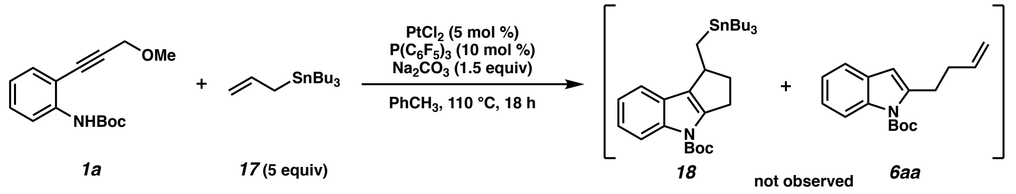


**Indoles 18/6aa.** To a solution of $P(C_6F_5)_3$ (5.3 mg, 0.0100 mmol) in toluene (0.50 mL) at 23 °C was added $PtCl_2$ (1.3 mg, 0.00500 mmol), and the resulting solution was stirred for 15 min. The catalyst solution was then added to a prestirred solution of alkyne **1a** (26.1 mg, 0.100 mmol) and allylstannane **17** (155 µL, 0.500 mmol) in toluene (0.50 mL, 0.1 M in substrate final concentration) at 23 °C. $Na_2CO_3$ (15.9 mg, 0.150 mmol) was then added, and the resulting mixture was stirred in a 110 °C heating block. After 18 h, the reaction mixture was cooled to room temperature and filtered through a $SiO_2$ plug (0.5 x 3 cm), eluting with 1:1 EtOAc/hexanes (4 mL). The solvent was removed by rotary evaporation, and the resulting crude residue was analyzed by $^1$H NMR. Cycloadduct **18** was not observed, nor was potential allylation product **6aa**.

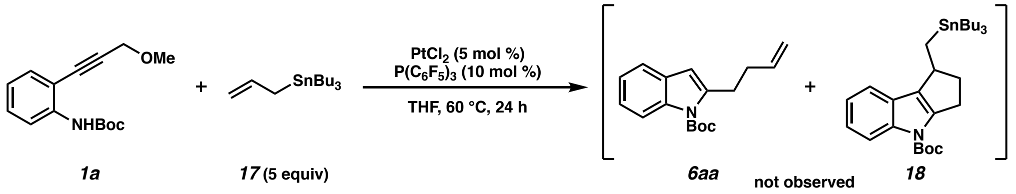


**Indoles 6aa/18.** To a solution of $P(C_6F_5)_3$ (5.3 mg, 0.0100 mmol) in THF (0.50 mL) at 23 °C was added $PtCl_2$ (1.3 mg, 0.00500 mmol), and the resulting solution was stirred for 15 min. The catalyst solution was then added to a prestirred solution of alkyne **1a** (26.1 mg, 0.100 mmol) and allylstannane **17** (155 µL, 0.500 mmol) in THF (0.50 mL, 0.1 M in substrate final concentration) at 23 °C. The resulting mixture was stirred in a 60 °C heating block. After 24 h, the reaction mixture was cooled to room temperature and filtered through a $SiO_2$ plug (0.5 x 3 cm), eluting with 1:1 EtOAc/hexanes (4 mL). The solvent was removed by rotary evaporation, and the resulting crude residue was analyzed by $^1$H NMR. Allylation product **6aa** was not observed, nor was potential cycloadduct **18**.

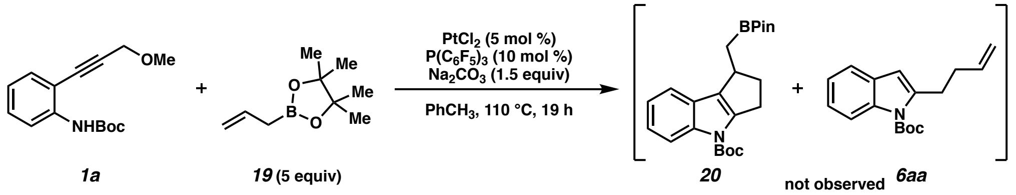


**Indoles 20/6aa.** To a solution of $P(C_6F_5)_3$ (2.7 mg, 0.00501 mmol) in toluene (0.25 mL) at 23 °C was added $PtCl_2$ (0.7 mg, 0.00251 mmol), and the resulting solution was stirred for 15 min. The catalyst solution was

then added to a prestirred solution of alkyne **1a** (13.1 mg, 0.0501 mmol) and allylboronate **19** (47.1 µL, 0.251 mmol) in toluene (0.25 mL, 0.1 M in substrate final concentration) at 23 °C. $Na_2CO_3$ (8.0 mg, 0.0752 mmol) was then added, and the resulting mixture was stirred in a 110 °C heating block. After 19 h, the reaction mixture was cooled to room temperature and filtered through a $SiO_2$ plug (0.5 x 3 cm), eluting with 1:1 EtOAc/hexanes (4 mL). The solvent was removed by rotary evaporation, and the resulting crude residue was analyzed by $^1$H NMR. Cycloadduct **20** was not observed, nor was potential allylation product **6aa**.

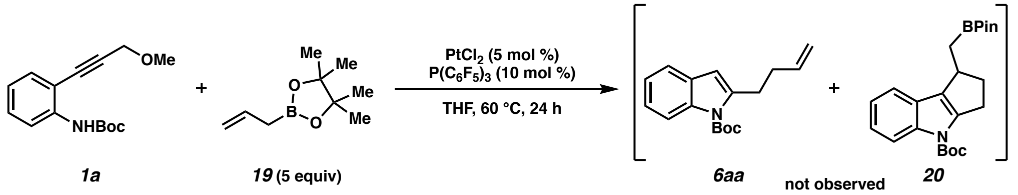


**Indoles 6aa/20.** To a solution of $P(C_6F_5)_3$ (2.7 mg, 0.00501 mmol) in THF (0.25 mL) at 23 °C was added $PtCl_2$ (0.7 mg, 0.00251 mmol), and the resulting solution was stirred for 15 min. The catalyst solution was then added to a prestirred solution of alkyne **1a** (13.1 mg, 0.0501 mmol) and allylboronate **19** (47.1 µL, 0.251 mmol) in THF (0.25 mL, 0.1 M in substrate final concentration) at 23 °C. The resulting mixture was stirred in a 60 °C heating block. After 24 h, the reaction mixture was cooled to room temperature and filtered through a $SiO_2$ plug (0.5 x 3 cm), eluting with 1:1 EtOAc/hexanes (4 mL). The solvent was removed by rotary evaporation, and the resulting crude residue was analyzed by $^1$H NMR. Allylation product **6aa** was not observed, nor was potential cycloadduct **20**.

*Confirmation of Tetrahydrodibenzofuran Side Product (see pages S12-S14)*

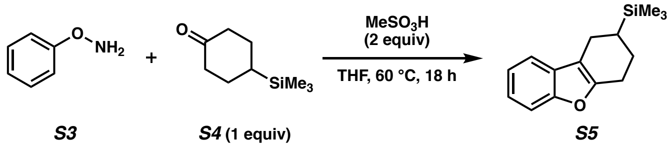


**Tetrahydrodibenzofuran S5.** Benzofuran **S5** was synthesized according to the procedure of Contiero and Jones.[1] To a solution of amine **S3** (100 mg, 0.916 mmol) in THF (1.83 mL) at 60 °C were added cyclohexanone **S4** (156 mg, 0.916 mmol) and methanesulfonic acid (0.119 mL, 1.83 mmol) sequentially. The reaction mixture was monitored by TLC until completion, and the solvent was then removed by rotary evaporation. The resulting residue was purified by flash chromatography (40:1 hexanes/EtOAc eluent) to afford tetrahydrodibenzofuran **S5** ($R_f$ = 0.72 in 9:1 hexanes/EtOAc) as a colorless oil.

**Data for Tetrahydrodibenzofuran S5.**
**$^1$H NMR** (400 MHz, $CDCl_3$): δ 7.44 – 7.38 (comp. m, 2H), 7.21 – 7.17 (comp. m, 2H), 2.83 – 2.75 (m, 1H), 2.75 – 2.64 (comp. m, 2H), 2.44 (app. t, *J* = 12.9 Hz, 1H), 2.10 (app. d, *J* = 12.4 Hz, 1H), 1.63 (m, 1H), 1.04 – 0.94 (m, 1H), 0.07 (s, 9H).
**$^{13}$C NMR** (100 MHz, $CDCl_3$): δ 154.5, 154.3, 128.8, 123.0, 122.2, 118.4, 113.5, 110.9, 24.7, 24.1, 21.8, 21.5, -3.2.
**IR** (film): 2919, 2841, 1642, 1451, 1304, 1247, 1211, 1175, 1115, 1039, 1009, 968, 880, 739, 691 cm$^{-1}$.
**HRMS** (ESI$^+$): *m/z* calc'd for $(M + H)^+$ $[C_{15}H_{20}OSi + H]^+$: 245.1356, found 245.1355.

## *Identification of Allylation Byproduct Ether*

The formation of $Me_3SiOMe$ was confirmed by GC analysis of a standard reaction mixture (i.e., alkyne **1a** + allylsilane **4a**) and comparison to an authentic sample. The ether is labile and readily hydrolyzes to form MeOH.

*GC Trace: Authentic sample of $Me_3SiOMe$ in $CH_2Cl_2$ and THF.* (Method: 30 °C for 30 min, then ramp 5 °C/min to 200 °C.)

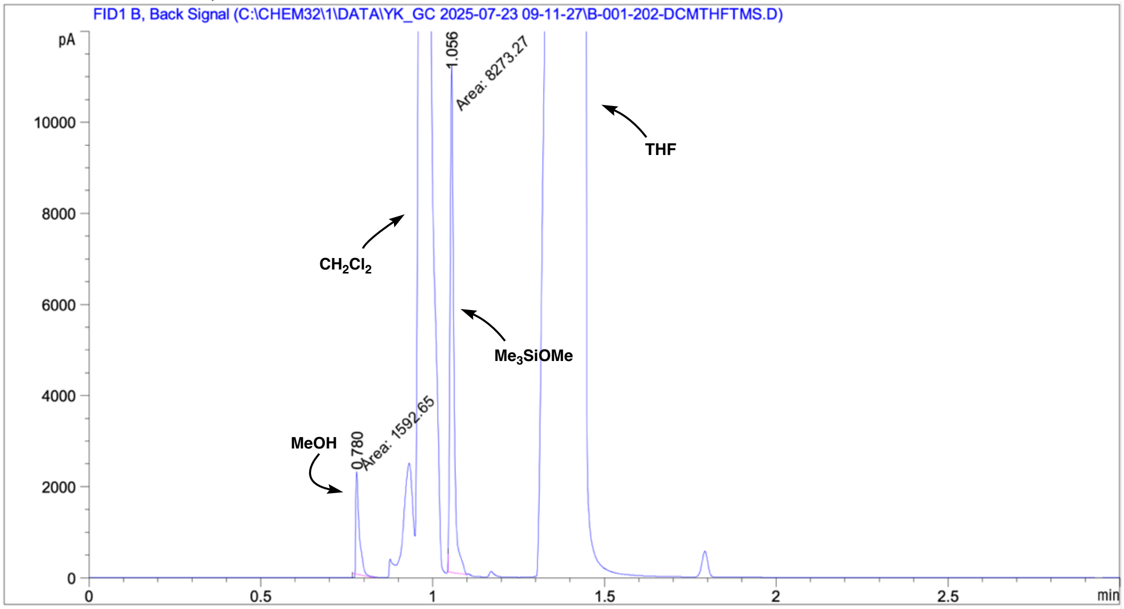


*GC Trace: Pt-catalyzed allylation.* (Method: 30 °C for 30 min, then ramp 5 °C/min to 300 °C.)

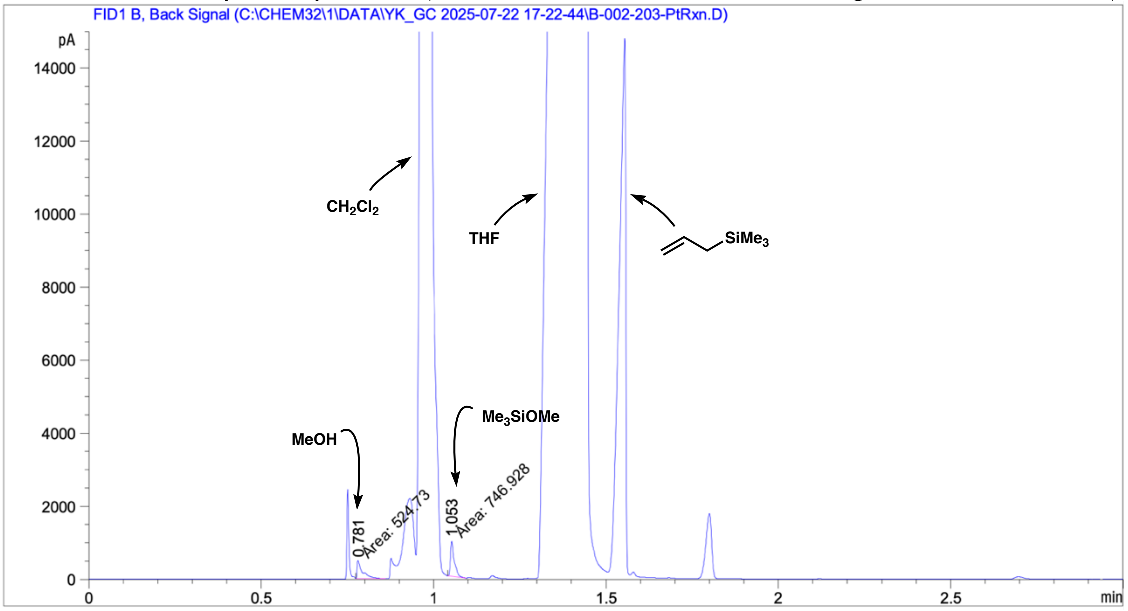

# Reaction Optimization

*Table S1. Optimization of (3+2) cycloaddition conditions.*

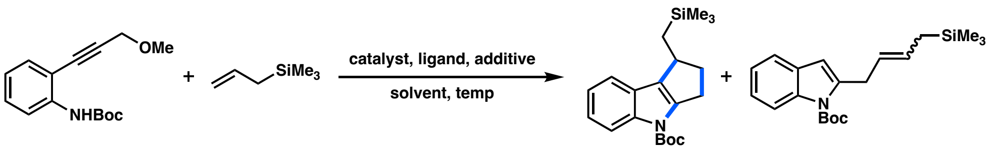


***1a*** ***4a*** **(5.0 equiv)** ***5aa*** ***7aa***

| Entry | Catalyst (5 mol % Pt) | Ligand (mol %) | Additive (equiv) | Solvent, temp (°C) | t (h) | Yield *5aa* (%)[a] | Yield *7aa* (%)[a] |
|---|---|---|---|---|---|---|---|
| 1 | $[(C_2H_4)PtCl_2]_2$ | — | — | $PhCH_3$, 80 | 3 | 49 | 18 |
| 2 | $[(C_2H_4)PtCl_2]_2$ | — | — | benzene, 80 | 3 | 28 | 9 |
| 3 | $[(C_2H_4)PtCl_2]_2$ | — | — | DCE, 80 | 19 | 14 | 7 |
| 4 | $[(C_2H_4)PtCl_2]_2$ | — | — | $CH_3CN$, 80 | 19 | 0 | 0 |
| 5 | $[(C_2H_4)PtCl_2]_2$ | — | — | MeOH, 80 | 19 | 0 | 0[b] |
| 6 | $[(C_2H_4)PtCl_2]_2$ | — | $MgCl_2$ (1.0) | $PhCH_3$, 80 | 2 | 41 | 18 |
| 7 | $[(C_2H_4)PtCl_2]_2$ | — | $Cu(OTf)_2$ (1.0) | $PhCH_3$, 80 | 3 | 20 | 10 |
| 8 | $[(C_2H_4)PtCl_2]_2$ | — | AgOAc (1.0) | $PhCH_3$, 100 | 20 | 0 | 0 |
| 9 | $PtCl_2(PhCN)_2$ | — | — | $PhCH_3$, 100 | 24 | 0 | 0 |
| 10 | $[(dppp)Pt(PhCN)_2](BF_4)_2$ | — | — | $PhCH_3$, 100 | 24 | 0 | 0 |
| 11 | $PtCl_2$ | 1-octene (100) | — | $PhCH_3$, 100 | 3 | 36 | 9 |
| 12 | $PtCl_2$ | methyl acrylate (100) | — | $PhCH_3$, 100 | 3 | 38 | 11 |
| 13 | $PtCl_2$ | styrene (100) | — | $PhCH_3$, 100 | 3 | 46 | 11 |
| 14 | $PtCl_2$ | norbornadiene (100) | — | $PhCH_3$, 100 | 48 | 25 | 8 |
| 15 | $PtCl_2$ | COD (100) | — | $PhCH_3$, 100 | 48 | 10 | 3 |
| 16 | $PtCl_2$ | 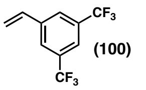  (100) | — | $PhCH_3$, 100 | 2 | 48 | 13 |
| 17 | $PtCl_2$ | styrene (100) | $AgBF_4$ (0.1) | $PhCH_3$, 100 | 20 | 0 | 0 |
| 18 | $PtCl_2$ | $PPh_3$ (10) | — | $PhCH_3$, 80 | 22 | 11 | <5 |
| 19 | $PtCl_2$ | $P(OPh)_3$ (10) | — | $PhCH_3$, 80 | 22 | 24 | 11 |
| 20 | $PtCl_2$ | $P(C_6F_5)_3$ (10) | — | $PhCH_3$, 80 | 2 | 64 | 2 |
| 21 | $[(C_2H_4)PtCl_2]_2$ | $P(C_6F_5)_3$ (10) | — | $PhCH_3$, 80 | 18 | 52 | 1 |
| 22 | $PtCl_2$ | $P(C_6F_5)_3$ (10) | $Li_2CO_3$ (1.5) | $PhCH_3$, 80 | 2 | 62 | <5 |
| 23 | $PtCl_2$ | $P(C_6F_5)_3$ (10) | $Na_2CO_3$ (1.5) | $PhCH_3$, 80 | 1 | 87 | <5 |
| 24 | $PtCl_2$ | $P(C_6F_5)_3$ (10) | $K_2CO_3$ (1.5) | $PhCH_3$, 80 | 20 | 50 | <5 |
| 25 | $PtCl_2$ | $P(C_6F_5)_3$ (10) | $Cs_2CO_3$ (1.5) | $PhCH_3$, 80 | 20 | 14 | <5 |
| 26 | $PtCl_2$ | $P(C_6F_5)_3$ (10) | $NaHCO_3$ (1.5) | $PhCH_3$, 80 | 2 | 56 | <5 |
| 27 | $PtCl_2$ | $P(C_6F_5)_3$ (10) | $Na_2CO_3$ (1.0) | $PhCH_3$, 80 | 5 | 81 | 0 |
| 28 | $PtCl_2$ | $P(C_6F_5)_3$ (10) | $Na_2CO_3$ (1.5) | $PhCH_3$, 110 | 1 | 93 | <5 |
| 29 | $PtCl_2$ | $P(C_6F_5)_3$ (5) | $Na_2CO_3$ (1.5) | $PhCH_3$, 110 | 1 | 80 | <5 |
| 30 | $PtCl_2$ | $P(C_6F_5)_3$ (15) | $Na_2CO_3$ (1.5) | $PhCH_3$, 110 | 4 | 91 | <5 |
| 31[c] | $PtCl_2$ | $P(C_6F_5)_3$ (10) | $Na_2CO_3$ (1.5) | $PhCH_3$, 110 | 1 | 69 | <5 |
| 32[d] | $PtCl_2$ | $P(C_6F_5)_3$ (10) | $Na_2CO_3$ (1.5) | $PhCH_3$, 110 | 1 | 75 | <5 |
| 33 | $PtCl_2$[e] | $P(C_6F_5)_3$ (6.0) | $Na_2CO_3$ (1.5) | $PhCH_3$, 80 | 10 | 70 | 0 |

[a] NMR yield based on vanillin as an internal standard. [b] Indole *8* observed as sole product. [c] 1.2 equiv *4a* used. [d] 2.5 equiv *4a* used. [e] 3 mol % Pt.

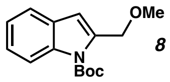


***8***

*Entries 1-5:* To a solution of alkyne **1a** (13.1 mg, 0.0501 mmol) and allyltrimethylsilane (**4a**, 39.8 μL, 0.251 mmol) in the listed solvent (0.50 mL, 0.1 M with respect to alkyne **1a**) at 23 °C was added $[PtCl_2(C_2H_4)]_2$ (0.7 mg, 0.00125 mmol). The resulting mixture was then stirred in an 80 °C heating block and monitored by TLC. After the listed time, the reaction mixture was cooled to room temperature and then filtered through a $SiO_2$ plug (0.5 x 3 cm), eluting with 1:1 EtOAc/hexanes (5 mL). The filtrate was concentrated in vacuo, and the resulting crude residue was analyzed by $^1$H NMR using vanillin as a standard.

For entry 5, indole **8** was the only observed product.

**Data for indole 8.**
**$^1$H NMR** (400 MHz, $CDCl_3$): δ 8.10 (d, *J* = 8.2 Hz, 1H), 7.51 (d, *J* = 7.5 Hz, 1H), 7.29 – 7.19 (comp. m, 2H), 6.64 (s, 1H), 4.81 (s, 2H), 3.48 (s, 3H), 1.69 (s, 9H).
**$^{13}$C NMR** (100 MHz, $CDCl_3$): δ 150.5, 138.1, 136.9, 129.3, 124.0, 122.9, 120.5, 115.7, 108.3, 84.2, 69.6, 58.7, 28.4.
**IR** (film): 2983, 2934, 2450, 2358, 1730, 1309, 1251, 1217, 1169, 1115, 1043, 871 cm$^{-1}$.
**HRMS** (ESI$^+$): *m/z* calc'd for $(M + Na)^+$ $[C_{15}H_{19}NO_3 + Na]^+$: 284.1257, found 284.1255.

*Entries 6-8:* To a solution of alkyne **1a** (13.1 mg, 0.0501 mmol) and allyltrimethylsilane (**4a**, 39.8 μL, 0.251 mmol) in toluene (0.50 mL, 0.1 M with respect to alkyne **1a**) at 23 °C was added $[PtCl_2(C_2H_4)]_2$ (0.7 mg, 0.00125 mmol). The listed additive was then added (1.0 equiv to alkyne **1a**), and the resulting mixture was stirred in an 80 – 100 °C heating block and monitored by TLC. After the listed time, the reaction mixture was cooled to room temperature and then filtered through a $SiO_2$ plug (0.5 x 3 cm), eluting with 1:1 EtOAc/hexanes (5 mL). The filtrate was concentrated in vacuo, and the resulting crude residue was analyzed by $^1$H NMR using vanillin as a standard.

*Entries 9, 10:* To a solution of alkyne **1a** (13.1 mg, 0.0501 mmol) and allyltrimethylsilane (**4a**, 39.8 μL, 0.251 mmol) in toluene (0.50 mL, 0.1 M with respect to alkyne **1a**) at 23 °C was added the listed Pt source (5 mol % relative to alkyne **1a**). The resulting mixture was stirred in a 100 °C heating block and monitored by TLC. After the listed time, the reaction mixture was cooled to room temperature and then filtered through a $SiO_2$ plug (0.5 x 3 cm), eluting with 1:1 EtOAc/hexanes (5 mL). The filtrate was concentrated in vacuo, and the resulting crude residue was analyzed by $^1$H NMR using vanillin as a standard.

*Entries 11-16:* To a solution of the listed alkene ligand (20 equiv relative to Pt catalyst) in toluene (0.25 mL) at 23 °C was added $PtCl_2$ (0.7 mg, 0.00251 mmol), and the resulting catalyst mixture was stirred for 15 min. This catalyst mixture was then added to a prestirred solution of alkyne **1a** (13.1 mg, 0.0501 mmol) and allyltrimethylsilane (**4a**, 39.8 μL, 0.251 mmol) in toluene (0.25 mL, 0.1 M total volume with respect to alkyne **1a**) at 23 °C. The resulting mixture was stirred in a 100 °C heating block and monitored by TLC. After the listed time, the reaction mixture was cooled to room temperature and then filtered through a $SiO_2$ plug (0.5 x 3 cm), eluting with 1:1 EtOAc/hexanes (5 mL). The filtrate was concentrated in vacuo, and the resulting crude residue was analyzed by $^1$H NMR using vanillin as a standard.

*Entry 17:* To a solution of styrene (5.8 μL, 0.0501 mmol) in toluene (0.25 mL) at 23 °C was added $PtCl_2$ (0.7 mg, 0.00251 mmol), and the resulting catalyst mixture was stirred for 15 min. This catalyst mixture was then added to a prestirred solution of alkyne **1a** (13.1 mg, 0.0501 mmol) and allyltrimethylsilane (**4a**, 39.8 μL, 0.251 mmol) in toluene (0.25 mL, 0.1 M total volume with respect to alkyne **1a**) at 23 °C. $AgBF_4$ (1.0 mg, 0.00501 mmol) was then added, and the resulting mixture was stirred in a 100 °C heating block and monitored by TLC. After 20 h, the reaction mixture was cooled to room temperature and then filtered through a $SiO_2$ plug (0.5 x 3 cm), eluting with 1:1 EtOAc/hexanes (5 mL). The filtrate was concentrated in vacuo, and the resulting crude residue was analyzed by $^1$H NMR using vanillin as a standard.

*Entries 18-20:* To a solution of the listed phosphorus ligand (2 equiv relative to Pt catalyst) in toluene (0.25 mL) at 23 °C was added $PtCl_2$ (0.7 mg, 0.00251 mmol), and the resulting catalyst mixture was stirred for

15 min. This catalyst mixture was then added to a prestirred solution of alkyne **1a** (13.1 mg, 0.0501 mmol) and allyltrimethylsilane (**4a**, 39.8 µL, 0.251 mmol) in toluene (0.25 mL, 0.1 M total volume with respect to alkyne **1a**) at 23 °C. The resulting mixture was stirred in an 80 °C heating block and monitored by TLC. After the listed time, the reaction mixture was cooled to room temperature and then filtered through a $SiO_2$ plug (0.5 x 3 cm), eluting with 1:1 EtOAc/hexanes (5 mL). The filtrate was concentrated in vacuo, and the resulting crude residue was analyzed by $^1H$ NMR using vanillin as a standard.

*Entry 21:* To a solution of $P(C_6F_5)_3$ (2.7 mg, 0.00501 mmol) in toluene (0.25 mL) at 23 °C was added $[PtCl_2(C_2H_4)]_2$ (0.7 mg, 0.00125 mmol), and the resulting catalyst mixture was stirred for 15 min. This catalyst mixture was then added to a prestirred solution of alkyne **1a** (13.1 mg, 0.0501 mmol) and allyltrimethylsilane (**4a**, 39.8 µL, 0.251 mmol) in toluene (0.25 mL, 0.1 M total volume with respect to alkyne **1a**) at 23 °C. The resulting mixture was stirred in an 80 °C heating block and monitored by TLC. After 18 h, the reaction mixture was cooled to room temperature and then filtered through a $SiO_2$ plug (0.5 x 3 cm), eluting with 1:1 EtOAc/hexanes (5 mL). The filtrate was concentrated in vacuo, and the resulting crude residue was analyzed by $^1H$ NMR using vanillin as a standard.

*Entries 22-28:* To a solution of $P(C_6F_5)_3$ (2.7 mg, 0.00501 mmol) in toluene (0.25 mL) at 23 °C was added $PtCl_2$ (0.7 mg, 0.00251 mmol), and the resulting catalyst mixture was stirred for 15 min. This catalyst mixture was then added to a prestirred solution of alkyne **1a** (13.1 mg, 0.0501 mmol) and allyltrimethylsilane (**4a**, 39.8 µL, 0.251 mmol) in toluene (0.25 mL, 0.1 M total volume with respect to alkyne **1a**) at 23 °C. The listed additive was then added (1.0-1.5 equiv to alkyne **1a**), and the resulting mixture was stirred in an 80 – 110 °C heating block and monitored by TLC. After the listed time, the reaction mixture was cooled to room temperature and then filtered through a $SiO_2$ plug (0.5 x 3 cm), eluting with 1:1 EtOAc/hexanes (5 mL). The filtrate was concentrated in vacuo, and the resulting crude residue was analyzed by $^1H$ NMR using vanillin as a standard.

*Entry 29:* To a solution of $P(C_6F_5)_3$ (1.3 mg, 0.00251 mmol) in toluene (0.25 mL) at 23 °C was added $PtCl_2$ (0.7 mg, 0.00251 mmol), and the resulting catalyst mixture was stirred for 15 min. This catalyst mixture was then added to a prestirred solution of alkyne **1a** (13.1 mg, 0.0501 mmol) and allyltrimethylsilane (**4a**, 39.8 µL, 0.251 mmol) in toluene (0.25 mL, 0.1 M total volume with respect to alkyne **1a**) at 23 °C. $Na_2CO_3$ (8.0 mg, 0.0752 mmol) was then added, and the resulting mixture was stirred in a 110 °C heating block and monitored by TLC. After 1 h, the reaction mixture was cooled to room temperature and then filtered through a $SiO_2$ plug (0.5 x 3 cm), eluting with 1:1 EtOAc/hexanes (5 mL). The filtrate was concentrated in vacuo, and the resulting crude residue was analyzed by $^1H$ NMR using vanillin as a standard.

*Entry 30:* To a solution of $P(C_6F_5)_3$ (7.8 mg, 0.0147 mmol) in toluene (0.49 mL) at 23 °C was added $PtCl_2$ (1.3 mg, 0.00490 mmol), and the resulting catalyst mixture was stirred for 15 min. This catalyst mixture was then added to a prestirred solution of alkyne **1a** (25.6 mg, 0.0980 mmol) and allyltrimethylsilane (**4a**, 77.9 µL, 0.490 mmol) in toluene (0.49 mL, 0.1 M total volume with respect to alkyne **1a**) at 23 °C. $Na_2CO_3$ (15.6 mg, 0.147 mmol) was then added, and the resulting mixture was stirred in a 110 °C heating block and monitored by TLC. After 4 h, the reaction mixture was cooled to room temperature and then filtered through a $SiO_2$ plug (0.5 x 3 cm), eluting with 1:1 EtOAc/hexanes (5 mL). The filtrate was concentrated in vacuo, and the resulting crude residue was analyzed by $^1H$ NMR using vanillin as a standard.

*Entry 31:* To a solution of $P(C_6F_5)_3$ (2.7 mg, 0.00501 mmol) in toluene (0.25 mL) at 23 °C was added $PtCl_2$ (0.7 mg, 0.00251 mmol), and the resulting catalyst mixture was stirred for 15 min. This catalyst mixture was then added to a prestirred solution of alkyne **1a** (13.1 mg, 0.0501 mmol) and allyltrimethylsilane (**4a**, 9.6 µL, 0.0601 mmol) in toluene (0.25 mL, 0.1 M total volume with respect to alkyne **1a**) at 23 °C. $Na_2CO_3$ (8.0 mg, 0.0752 mmol) was then added, and the resulting mixture was stirred in a 110 °C heating block and monitored by TLC. After 1 h, the reaction mixture was cooled to room temperature and then was filtered through a $SiO_2$ plug (0.5 x 3 cm), eluting with 1:1 EtOAc/hexanes (5 mL). The filtrate was concentrated in vacuo, and the resulting crude residue was analyzed by $^1H$ NMR using vanillin as a standard.

*Entry 32:* To a solution of $P(C_6F_5)_3$ (2.7 mg, 0.00501 mmol) in toluene (0.25 mL) at 23 °C was added $PtCl_2$ (0.7 mg, 0.00251 mmol), and the resulting catalyst mixture was stirred for 15 min. This catalyst mixture was then added to a prestirred solution of alkyne **1a** (13.1 mg, 0.0501 mmol) and allyltrimethylsilane (**4a**, 19.9 µL, 0.0125 mmol) in toluene (0.25 mL, 0.1 M total volume with respect to alkyne **1a**) at 23 °C. $Na_2CO_3$ (8.0 mg, 0.0752 mmol) was then added, and the resulting mixture was stirred in a 110 °C heating block and monitored by TLC. After 1 h, the reaction mixture was cooled to room temperature and then was filtered through a $SiO_2$ plug (0.5 x 3 cm), eluting with 1:1 EtOAc/hexanes (5 mL). The filtrate was concentrated in vacuo, and the resulting crude residue was analyzed by $^1H$ NMR using vanillin as a standard.

*Entry 33:* To a solution of $P(C_6F_5)_3$ (1.6 mg, 0.00301 mmol) in toluene (0.25 mL) at 23 °C was added $PtCl_2$ (0.4 mg, 0.00150 mmol), and the resulting catalyst mixture was stirred for 15 min. This catalyst mixture was then added to a prestirred solution of alkyne **1a** (13.1 mg, 0.0501 mmol) and allyltrimethylsilane (**4a**, 39.8 µL, 0.251 mmol) in toluene (0.25 mL, 0.1 M total volume with respect to alkyne **1a**) at 23 °C. $Na_2CO_3$ (8.0 mg, 0.0752 mmol) was then added, and the resulting mixture was stirred in an 80 °C heating block and monitored by TLC. After 1 h, the reaction mixture was cooled to room temperature and then filtered through a $SiO_2$ plug (0.5 x 3 cm), eluting with 1:1 EtOAc/hexanes (5 mL). The filtrate was concentrated in vacuo, and the resulting crude residue was analyzed by $^1H$ NMR using vanillin as a standard.

*Table S2. Optimization of allylation conditions.*

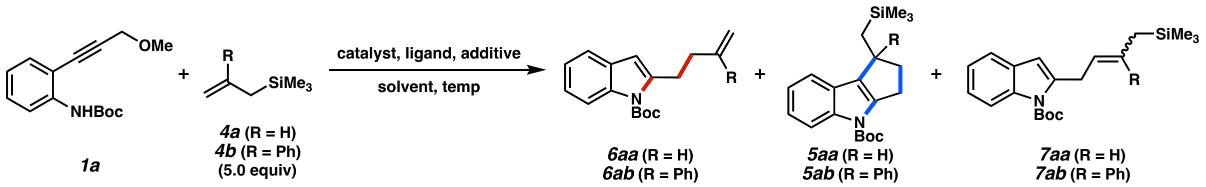


| Entry | Catalyst (5 mol % Pt) | R | Ligand (mol %) | Additive (1.0 equiv) | Solvent, temp (°C) | t (h) | Yield *6* (%)[a] | Yield *5* (%)[a] | Yield *7* (%)[a] |
|---|---|---|---|---|---|---|---|---|---|
| 1 | $[(C_2H_4)PtCl_2]_2$ | H | – | – | THF, 23 | 3 | 43 | 16 | 11 |
| 2 | $[(C_2H_4)PtCl_2]_2$ | H | – | – | 1,4-dioxane, 23 | 5 | 14 | 18 | 14 |
| 3 | $[(C_2H_4)PtCl_2]_2$ | H | – | – | MTBE, 23 | 3 | 14 | 22 | 12 |
| 4 | $[(C_2H_4)PtCl_2]_2$ | H | – | – | EtOAc, 40 | 5 | 22 | 15 | 6 |
| 5 | $[(C_2H_4)PtCl_2]_2$ | H | – | – | DMF, 100 | 24 | 10 | 0 | 0 |
| 6 | $[(C_2H_4)PtCl_2]_2$ | H | – | – | $CH_3NO_2$, 80 | 3 | 15 | 12 | 0 |
| 7 | $[(C_2H_4)PtCl_2]_2$ | H | – | $Na_2CO_3$ | THF, 23 | 36 | 35 | 13 | 9 |
| 8 | $PtCl_2(PhCN)_2$ | H | – | – | THF, 50 | 24 | 14 | 7 | 0 |
| 9 | $[(dppp)Pt(PhCN)_2](BF_4)_2$ | H | – | – | THF, 50 | 24 | 0 | 0 | 0 |
| 10 | $PtCl_2$ | H | – | – | THF, 23 | 6 | 47 | 19 | 0 |
| 11 | $PtCl_2$ | H | methyl acrylate (100) | – | THF, 23 | 20 | 50 | 18 | 0 |
| 12 | $PtCl_2$ | H | $ArCH=CH_2$ (100) (Ar: 3,5-$(F_3C)_2C_6H_3$) | – | THF, 23 | 19 | 50 | 20 | 0 |
| 13 | $PtCl_2$ | H | CO (1 atm) | – | THF, 23 | 21 | 0 | 0 | 0 |
| 14 | $PtCl_2$ | H | $PPh_3$ (10) | – | THF, 50 | 24 | 0 | 0 | 0 |
| 15 | $PtCl_2$ | H | $P(OPh)_3$ (10) | – | THF, 23 | 18 | 48 | 13 | 0 |
| 16 | $PtCl_2$ | H | $P(C_6F_5)_3$ (10) | – | THF, 23 | 21 | 69 | 15 | 0 |
| 17 | $PtCl_2$ | H | $P(C_6F_5)_3$ (10) | – | THF, 50 | 21 | 60 | 18 | 0 |
| 18 | $PtCl_2$ | H | $P(C_6F_5)_3$ (10) | – | THF, 60 | 18 | 58 | 21 | 0 |
| 19 | $PtCl_2$ | H | $P(C_6F_5)_3$ (5) | – | THF, 60 | 18 | 44 | 24 | 0 |
| 20 | $PtCl_2$ | H | $P(C_6F_5)_3$ (15) | – | THF, 60 | 18 | 53 | 17 | 0 |
| 21 | $PtCl_2$ | H | $P(C_6F_5)_3$ (10) | $Na_2CO_3$ | THF, 50 | 11 | 0 | 0 | 0 |
| 22 | $PtCl_2$ | H | $P(C_6F_5)_3$ (10) | TBAF | THF, 23 | 24 | 0 | 0 | 0 |
| 23 | $PtCl_2$ | H | $P(C_6F_5)_3$ (10) | CsF | THF, 50 | 24 | 0 | 0 | 0 |
| 24 | $PtCl_2$[b] | H | $P(C_6F_5)_3$ (20) | – | THF, 23 | 20 | 77 | 15 | 0 |
| 25[c] | $PtCl_2$ | H | $P(C_6F_5)_3$ (10) | – | THF, 23 | 21 | 64 | 29 | 0 |
| 26 | $PtCl_2$ | Ph | $P(C_6F_5)_3$ (10) | – | THF, 23 | 19 | 95 | 0 | 0 |

[a] NMR yield based on vanillin as an internal standard. [b] 10 mol % Pt. [c] 1.2 equiv *4a* used.

*Entries 1-6:* To a solution of alkyne **1a** (13.1 mg, 0.0501 mmol) and allyltrimethylsilane (**4a**, 39.8 µL, 0.251 mmol) in the listed solvent (0.50 mL, 0.1 M with respect to alkyne **1a**) at 23 °C was added $[PtCl_2(C_2H_4)]_2$ (0.7 mg, 0.00125 mmol). The resulting mixture was stirred either at 23 °C or in a heating block at the listed temperature and monitored by TLC. After the listed time, the reaction mixture was cooled to room temperature and filtered through a $SiO_2$ plug (0.5 x 3 cm), eluting with 1:1 EtOAc/hexanes (5 mL). The filtrate was concentrated in vacuo, and the resulting crude residue was analyzed by $^1H$ NMR using vanillin as a standard.

*Entry 7:* To a solution of alkyne **1a** (13.1 mg, 0.0501 mmol) and allyltrimethylsilane (**4a**. 39.8 µL, 0.251 mmol) in THF (0.50 mL, 0.1 M with respect to alkyne **1a**) at 23 °C was added $[PtCl_2(C_2H_4)]_2$ (0.7 mg, 0.00125 mmol). $Na_2CO_3$ (5.3 mg, 0.0501 mmol) was then added, and the resulting mixture was stirred at 23 °C and monitored by TLC. After 36 h, the reaction mixture was filtered through a $SiO_2$ plug (0.5 x 3

cm), eluting with 1:1 EtOAc/hexanes (5 mL). The filtrate was concentrated in vacuo, and the resulting crude residue was analyzed by $^1$H NMR using vanillin as a standard.

*Entries 8,9:* To a solution of alkyne **1a** (13.1 mg, 0.0501 mmol) and allyltrimethylsilane (**4a**, 39.8 μL, 0.251 mmol) in THF (0.50 mL, 0.1 M with respect to alkyne **1a**) at 23 °C was added the listed Pt source (5 mol % relative to alkyne **1a**). The resulting mixture was stirred in a 50 °C heating block and monitored by TLC. After 24 h, the reaction mixture was cooled to room temperature and then filtered through a $SiO_2$ plug (0.5 x 3 cm), eluting with 1:1 EtOAc/hexanes (5 mL). The filtrate was concentrated in vacuo, and the resulting crude residue was analyzed by $^1$H NMR using vanillin as a standard.

*Entry 10:* To a solution of alkyne **1a** (13.1 mg, 0.0501 mmol) and allyltrimethylsilane (**4a**, 39.8 μL, 0.251 mmol) in THF (0.50 mL, 0.1 M with respect to alkyne **1a**) at 23 °C was added $PtCl_2$ (0.7 mg, 0.00251 mmol). The resulting mixture was stirred at 23 °C and monitored by TLC. After 6 h, the reaction mixture was filtered through a $SiO_2$ plug (0.5 x 3 cm), eluting with 1:1 EtOAc/hexanes (5 mL). The filtrate was concentrated in vacuo, and the resulting crude residue was analyzed by $^1$H NMR using vanillin as a standard.

*Entries 11,12:* To a solution of the listed alkene ligand (20 equiv relative to Pt catalyst) in THF (0.25 mL) at 23 °C was added $PtCl_2$ (0.7 mg, 0.00251 mmol), and the resulting catalyst mixture was stirred for 15 min. This catalyst mixture was then added to a prestirred solution of alkyne **1a** (13.1 mg, 0.0501 mmol) and allyltrimethylsilane (**4a**, 39.8 μL, 0.251 mmol) in THF (0.25 mL, 0.1 M total volume with respect to alkyne **1a**) at 23 °C. The resulting mixture was stirred at 23 °C and monitored by TLC. After the listed time, the reaction mixture was filtered through a $SiO_2$ plug (0.5 x 3 cm), eluting with 1:1 EtOAc/hexanes (5 mL). The filtrate was concentrated in vacuo, and the resulting crude residue was analyzed by $^1$H NMR using vanillin as a standard.

*Entry 13:* Into a solution of $PtCl_2$ (0.7 mg, 0.00251 mmol) in THF (0.25 mL) was bubbled CO (1 atm) for 1 minute at 23 °C, and the resulting catalyst mixture was stirred for an additional 15 min at this same temperature. This catalyst mixture was then added to a prestirred solution of alkyne **1a** (13.1 mg, 0.0501 mmol) and allyltrimethylsilane (**4a**, 39.8 μL, 0.251 mmol) in THF (0.25 mL, 0.1 M total volume with respect to alkyne **1a**, also under a CO atmosphere) at 23 °C. The resulting mixture was stirred at 23 °C and monitored by TLC. After 21 h, the reaction mixture was filtered through a $SiO_2$ plug (0.5 x 3 cm), eluting with 1:1 EtOAc/hexanes (5 mL). The filtrate was concentrated in vacuo, and the resulting crude residue was analyzed by $^1$H NMR using vanillin as a standard.

*Entries 14-17:* To a solution of the listed phosphorus ligand (2 x Pt catalyst mmol) in THF (0.25 mL) at 23 °C was added $PtCl_2$ (0.7 mg, 0.00251 mmol), and the resulting catalyst mixture was stirred for 15 min. This catalyst mixture was then added to a prestirred solution of alkyne **1a** (13.1 mg, 0.0501 mmol) and allyltrimethylsilane (**4a**, 39.8 μL, 0.251 mmol) in THF (0.25 mL, 0.1 M total volume with respect to alkyne **1a**) at 23 °C. The resulting mixture was stirred at 23 – 50 °C and monitored by TLC. After the listed time, the reaction mixture was cooled to room temperature and filtered through a $SiO_2$ plug (0.5 x 3 cm), eluting with 1:1 EtOAc/hexanes (5 mL). The filtrate was concentrated in vacuo, and the resulting crude residue was analyzed by $^1$H NMR using vanillin as a standard.

*Entries 18-20:* To a solution of $P(C_6F_5)_3$ (5-15 mol %) in THF (0.49 mL) at 23 °C was added $PtCl_2$ (1.3 mg, 0.00490 mmol), and the resulting catalyst mixture was stirred for 15 min. This catalyst mixture was then added to a prestirred solution of alkyne **1a** (25.6 mg, 0.0980 mmol) and allyltrimethylsilane (**4a**, 77.9 μL, 0.490 mmol) in THF (0.49 mL, 0.1 M total volume with respect to alkyne **1a**) at 23 °C. The resulting mixture was stirred in a 60 °C heating block and monitored by TLC. After 18 h, the reaction mixture was cooled to room temperature and filtered through a $SiO_2$ plug (0.5 x 3 cm), eluting with 1:1 EtOAc/hexanes (5 mL). The filtrate was concentrated in vacuo, and the resulting crude residue was analyzed by $^1$H NMR using vanillin as a standard.

*Entries 21-23:* To a solution of $P(C_6F_5)_3$ (2.7 mg, 0.00501 mmol) in THF (0.25 mL) at 23 °C was added $PtCl_2$ (0.7 mg, 0.00251 mmol), and the resulting catalyst mixture was stirred for 15 min. This catalyst mixture was then added to a prestirred solution of alkyne **1a** (13.1 mg, 0.0501 mmol) and allyltrimethylsilane (**4a**, 39.8 μL, 0.251 mmol) in THF (0.25 mL, 0.1 M total volume with respect to alkyne **1a**) at 23 °C. The listed additive was then added (1.0 equiv relative to alkyne **1a**), and the resulting mixture was stirred at 23 – 50 °C and monitored by TLC. After the listed time, the reaction mixture was cooled to room temperature filtered through a $SiO_2$ plug (0.5 x 3 cm), eluting with 1:1 EtOAc/hexanes (5 mL). The filtrate was concentrated in vacuo, and the resulting crude residue was analyzed by $^1$H NMR using vanillin as a standard.

*Entry 24:* To a solution of $P(C_6F_5)_3$ (5.3 mg, 0.0100 mmol) in THF (0.25 mL) at 23 °C was added $PtCl_2$ (1.3 mg, 0.00501 mmol), and the resulting catalyst mixture was stirred for 15 min. This catalyst mixture was then added to a prestirred solution of alkyne **1a** (13.1 mg, 0.0501 mmol) and allyltrimethylsilane (**4a**, 39.8 μL, 0.251 mmol) in THF (0.25 mL, 0.1 M total volume with respect to alkyne **1a**) at 23 °C. The resulting mixture was stirred at 23 °C and monitored by TLC. After 20 h, the reaction mixture was filtered through a $SiO_2$ plug (0.5 x 3 cm), eluting with 1:1 EtOAc/hexanes (5 mL). The filtrate was concentrated in vacuo, and the resulting crude residue was analyzed by $^1$H NMR using vanillin as a standard.

*Entry 25:* To a solution of $P(C_6F_5)_3$ (2.7 mg, 0.00501 mmol) in THF (0.25 mL) at 23 °C was added $PtCl_2$ (0.7 mg, 0.00251 mmol), and the resulting catalyst mixture was stirred for 15 min. This catalyst mixture was then added to a prestirred solution of alkyne **1a** (13.1 mg, 0.0501 mmol) and allyltrimethylsilane (**4a**, 9.6 μL, 0.0601 mmol) in THF (0.25 mL, 0.1 M total volume with respect to alkyne **1a**) at 23 °C. The resulting mixture was stirred at 23 °C and monitored by TLC. After 21 h, the reaction mixture was filtered through a $SiO_2$ plug (0.5 x 3 cm), eluting with 1:1 EtOAc/hexanes (5 mL). The filtrate was concentrated in vacuo, and the resulting crude residue was analyzed by $^1$H NMR using vanillin as a standard.

*Entry 26:* To a solution of $P(C_6F_5)_3$ (2.7 mg, 0.00501 mmol) in THF (0.25 mL) at 23 °C was added $PtCl_2$ (0.7 mg, 0.00251 mmol), and the resulting catalyst mixture was stirred for 15 min. This catalyst mixture was then added to a prestirred solution of alkyne **1a** (13.1 mg, 0.0501 mmol) and trimethyl(2-phenylallyl)silane (**4b**, 47.8 mg, 0.251 mmol) in THF (0.25 mL, 0.1 M total volume with respect to alkyne **1a**) at 23 °C. The resulting mixture was stirred at 23 °C and monitored by TLC. After 6 h, the reaction mixture was filtered through a $SiO_2$ plug (0.5 x 3 cm), eluting with 1:1 EtOAc/hexanes (5 mL). The filtrate was concentrated in vacuo, and the resulting crude residue was analyzed by $^1$H NMR using vanillin as a standard.

# Preparation of Aniline and Phenol Starting Materials

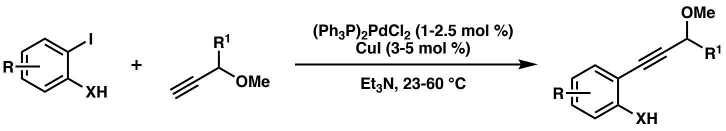


**General procedure for the Sonogashira cross coupling reaction.** Aryl iodide (1.0 equiv), propargylic ether (1.1-1.25 equiv), $PdCl_2(PPh_3)_3$ (1-2.5 mol %), and CuI (3-5 mol %) were combined in $Et_3N$ (0.33 M). Degassing with argon was performed via bubbling for 15 min. The resulting reaction mixture was stirred at the indicated temperature (23 or 60 °C) and monitored by TLC. Upon completion, the reaction mixture was filtered through a $SiO_2$ plug, eluting with EtOAc (3x reaction volume). The filtrate was concentrated via rotary evaporation, and the resulting residue was purified by flash chromatography on $SiO_2$.

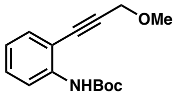


*1a*

**Alkyne 1a.** Prepared according to the procedure described by Saito and coworkers.[2]

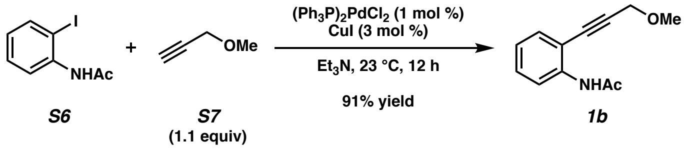


**Alkyne 1b.** To a mixture of 2-iodoacetanilide (**S6**, 1.00 g, 3.83 mmol) in $Et_3N$ (11.6 mL) at 23 °C was added propargylic ether **S7** (295 mg, 4.21 mmol), and the mixture was degassed with argon via bubbling for 15 min. CuI (21.9 mg, 0.115 mmol) was added, followed by $PdCl_2(PPh_3)_2$ (26.9 mg, 0.0383 mmol), and the resulting solution was stirred at 23 °C for 12 h. After this time, the reaction mixture was filtered through a $SiO_2$ plug (3 x 1.5 cm), eluting with EtOAc (35 mL). The filtrate was concentrated via rotary evaporation and the resulting residue was purified by flash chromatography (3:1 hexanes/EtOAc eluent), providing alkyne **1b** (708 mg, 91% yield, $R_f$ = 0.27 in 3:1 hexanes/EtOAc) as a colorless oil. Observed spectroscopic data for alkyne **1b** were in accordance with reported data.[3]

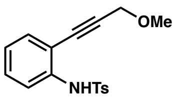


*1c*

**Alkyne 1c.** Prepared according to the procedure described by Shen and Lu.[4]

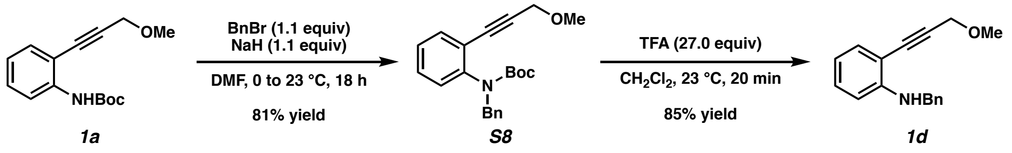

***N*-Bn aniline 1d.** To a solution of *N*-Boc aniline **1a** (250 mg, 0.957 mmol) in DMF (7.05 mL) at 0 ºC was added NaH (42.1 mg, 60% dispersion in mineral oil, 1.05 mmol) in four portions over 5 min. The resulting suspension was stirred at 0 °C for 15 min. Benzyl bromide (0.125 mL, 1.05 mmol) was then added at 0 °C. The ice bath was removed, and the reaction mixture was stirred overnight at 23 °C. Upon completion, the reaction mixture was poured into sat. aqueous $NH_4Cl$ (7 mL) and transferred to a separatory funnel. The layers were separated, and the aqueous phase was extracted with EtOAc (3 x 15 mL). The combined organic phases were sequentially washed with $H_2O$ (2 x 15 mL), sat. aqueous $NaHCO_3$ (15 mL), 5% aqueous LiCl (15 ml), and brine (15 mL). The combined phases were dried over $MgSO_4$, filtered, and concentrated by rotary evaporation to afford *N*-Bn, *N*-Boc aniline **S8** (274 mg, 81% yield, $R_f$ = 0.43 in 9:1 hexanes/EtOAc) as a yellow oil. The *N*-Bn aniline was sufficiently pure to be carried on to the subsequent transformation.

*N*-Bn, *N*-Boc aniline **S8** (274 mg, 0.780 mmol) was dissolved in $CH_2Cl_2$ (4.97 mL) at 23 °C, and to this solution was added TFA (1.61 mL, 21.0 mmol). The resulting mixture was stirred for 20 min at 23 ºC. Upon reaction completion as determined by TLC, the volatile materials were removed by rotary evaporation. The resulting residue was diluted with EtOAc (10 mL) and sat. aqueous $NaHCO_3$ (30 mL), and the mixture was transferred to a separatory funnel. The layers were separated, and the aqueous phase was extracted with EtOAc (2 x 10 mL). The combined organic phases were washed with brine (10 mL), dried over $Na_2SO_4$, and concentrated in vacuo to afford a crude yellow oil. The crude material was purified with flash column chromatography (9:1 hexanes/EtOAc eluent), providing *N*-Bn aniline **1d** (167 mg, 85% yield, $R_f$ = 0.35 in 9:1 hexanes/EtOAc) as a colorless oil.

**Data for Alkyne 1d.**
**$^1$H NMR** (400 MHz, $CDCl_3$): δ 7.39 – 7.30 (comp. m, 5H), 7.29 – 7.24 (comp. m, 1H), 7.15 (app. td, *J* = 7.7, 1.5 Hz, 1H), 6.63 (app. t, *J* = 7.6 Hz, 1H), 6.56 (d, *J* = 8.3 Hz, 1H), 5.11 (br. s, 1H), 4.43 (s, 2H), 4.37 (s, 2H), 3.42 (s, 3H).
**$^{13}$C NMR** (100 MHz, $CDCl_3$): δ 149.1, 139.2, 132.7, 130.3, 128.8, 127.4, 127.3, 116.7, 110.1, 107.1, 91.0, 83.3, 60.7, 57.7, 47.8.
**IR** (film): 3309, 2940, 2860, 2818, 1597, 1511, 1236, 1088 $cm^{-1}$.
**HRMS** ($ESI^+$) *m/z* calc'd for $(M + H)^+$ $[C_{17}H_{17}NO + H]^+$: 252.1383, found 252.1377.

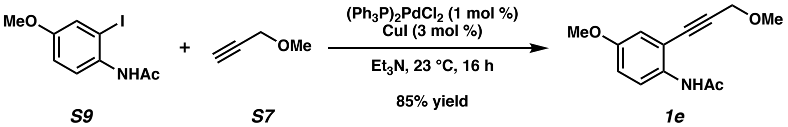

*S9* *S7* (1.1 equiv) *1e*

**Alkyne 1e.** To a mixture of 2-iodo-4-methoxyacetanilide (**S9**, 170 mg, 0.584 mmol) in $Et_3N$ (1.77 mL) at 23 °C was added propargylic ether **S7** (45.0 mg, 0.642 mmol), and the mixture was degassed with argon via bubbling for 15 min. CuI (3.3 mg, 0.0175 mmol) was added, followed by $PdCl_2(PPh_3)_2$ (4.1 mg, 0.00584 mmol), and the resulting reaction mixture was stirred at 23 °C for 16 h. Upon completion, the reaction mixture was filtered through a $SiO_2$ plug (3 x 1.5 cm), eluting with EtOAc (6.0 mL). The filtrate was concentrated via rotary evaporation, and the resulting residue was purified by flash chromatography (1:1 hexanes/EtOAc eluent), providing alkyne **1e** (116 mg, 85% yield, $R_f$ = 0.26 in 1:1 hexanes/EtOAc) as a light-orange solid.

**Data for Alkyne 1e.**
**$^1$H NMR** (400 MHz, $CDCl_3$): δ 8.24 (d, *J* = 9.3 Hz, 1H), 7.67 (br. s, 1H), 6.94 (s, 1H), 6.91 (d, *J* = 9.3 Hz, 1H), 4.39 (s, 2H), 3.78 (s, 3H), 3.48 (s, 3H), 2.20 (s. 3H).
**$^{13}$C NMR** (100 MHz, $CDCl_3$): δ 168.0, 155.4, 132.9, 121.4, 116.5, 116.1, 112.7, 92.0, 81.9, 60.5, 58.0, 55.7, 24.9.

**IR** (film): 3229, 2933, 2841, 1658, 1500, 1212, 1181 cm$^{-1}$.
**HRMS** (ESI$^+$) *m/z* calc'd for $(M + Na)^+$ $[C_{13}H_{15}NO_3 + Na]^+$: 256.0944, found 256.0936.

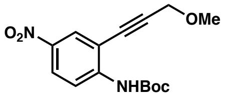

*1f*

**Alkyne 1f.** Prepared according to the procedure described by Allegretti and coworkers.[5]

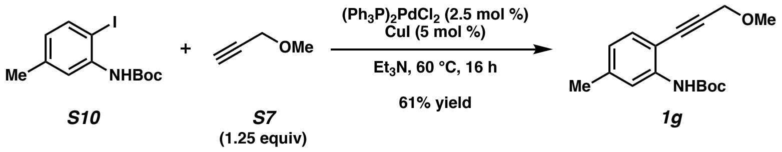


**Alkyne 1g.** To a solution of aryl iodide **S10** (693 mg, 2.08 mmol**)** in $Et_3N$ (6.3 mL) was added $Pd(PPh_3)_2Cl_2$ (36.5 mg, 0.0520 mmol) and CuI (19.8 mg, 0.104 mmol) at 23 ºC, and the mixture was degassed with argon via bubbling for 15 min. The reaction mixture was heated to 60 ºC, and then alkyne **S7** (182 mg, 2.60 mmol) was added. The reaction mixture was then stirred at 60 ºC for 16 h. Upon completion as determined by TLC, the reaction mixture was cooled to room temperature and then filtered through a $SiO_2$ plug (4.5 x 1.5 cm), eluting with EtOAc (40 mL). The filtrate was concentrated via rotary evaporation, and the resulting crude residue was purified by silica gel chromatography (9:1 hexanes/EtOAc eluent) to afford alkyne **1g** (348 mg, 61% yield, $R_f$ = 0.27 in 19:1 hexanes/EtOAc) as a light yellow oil.

**Data for Alkyne 1g.**
**$^1$H NMR** (400 MHz, $CDCl_3$): δ 7.99 (s, 1H), 7.27 (d, *J* = 8.1 Hz, 1H), 7.19 (br. s, 1H), 6.77 (d, *J* = 8.1 Hz, 1H), 4.39 (s, 2H), 3.47 (s, 3H), 2.34 (s, 3H), 1.53 (s, 9H).
**$^{13}$C NMR** (100 MHz, $CDCl_3$): δ 152.6, 140.6, 139.7, 132.0, 123.1, 118.2, 107.7, 91.3, 82.2, 80.9, 60.6, 57.8, 28.4, 22.1.
**IR** (film): 3406, 2977, 2928, 1729, 1522, 1240, 1096, 1048, 810, 766 cm$^{-1}$.
**HRMS** (ESI$^+$) *m/z* calc'd for $(M + Na)^+$ $[C_{16}H_{21}NO_3 + Na]^+$: 298.1414, found 298.1402.

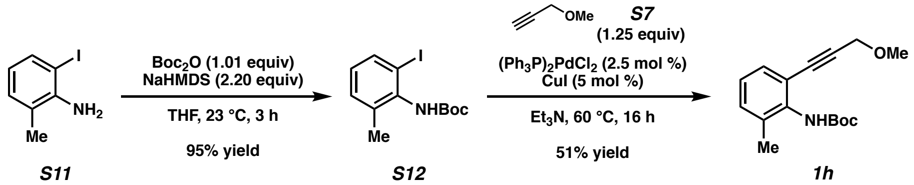


**Alkyne 1h.** To a solution of aniline **S11** (614 mg, 2.63 mmol) in THF (2.3 mL) at 23 ºC was added a solution of NaHMDS (2.89 mL, 2.0 M in THF, 5.79 mmol). A solution of $Boc_2O$ (580 mg, 2.66 mmol) in THF (2.3 mL) was added over 15 minutes at 23 ºC, and the resulting reaction mixture was stirred at 23 C for 3 h. Upon completion as determined by TLC**,** the THF was removed via rotary evaporation, and the resulting residue was diluted with sat. aqueous $NH_4Cl$ (5 mL) and extracted with EtOAc (3 x 25 mL). The organic layers were combined, washed with brine (25 mL), and dried over anhydrous $MgSO_4$. The solvent was removed by rotary evaporation to afford *N*-Boc aniline **S12** (834 mg, 95% yield, $R_f$ = 0.25 in 19:1 hexanes/EtOAc) as a dark red oil. The *N*-Boc aniline was sufficiently pure to be carried on to the subsequent transformation.

To a solution of *N*-Boc aniline **S12** (834 mg, 2.50 mmol**)** in $Et_3N$ (7.6 mL) was added $Pd(PPh_3)_2Cl_2$ (43.9 mg, 0.0625 mmol) and CuI (23.8 mg, 0.125 mmol) at 23 ºC. The solution was then degassed with argon via bubbling for 15 minutes. The reaction mixture was heated to 60 ºC, and then alkyne **S7** (219 mg, 3.13 mmol) was added. The reaction mixture was stirred at 60 ºC for 16 h. Upon completion as determined by TLC, the reaction mixture was cooled to room temperature and then filtered through a $SiO_2$ plug (4.5 x 1.5 cm), eluting with EtOAc (40 mL). The filtrate was concentrated via rotary evaporation, and the resulting crude residue was purified by silica gel chromatography (9:1 hexanes/EtOAc eluent) to afford alkyne **1h** (350 mg, 51% yield, $R_f$ = 0.22 in 9:1 hexanes/EtOAc) as a yellow-orange solid.

**Data for Alkyne 1h.**
**$^1$H NMR** (400 MHz, $CDCl_3$): δ 7.30 (d, *J* = 7.8 Hz, 1H), 7.20 (d, *J* = 7.7 Hz, 1H), 7.09 (app. t, *J* = 7.7 Hz, 1H), 6.28 (br. s, 1H), 4.36 (s, 2H), 3.46 (s, 3H), 2.30 (s, 3H), 1.49 (s, 9H).
**$^{13}$C NMR** (100 MHz, $CDCl_3$): δ 153.6, 137.0, 135.9, 131.4, 130.1, 126.3, 120.2, 89.9, 83.5, 80.4, 60.6, 57.7, 28.4, 18.7.
**IR** (film): 3300, 2977, 2928, 1703, 1493, 1365, 1161, 1099, 903, 784 $cm^{-1}$.
**HRMS** ($ESI^+$) *m/z* calc'd for $(M + Na)^+$ $[C_{16}H_{21}NO_3 + Na]^+$: 298.1414, found 298.1408.

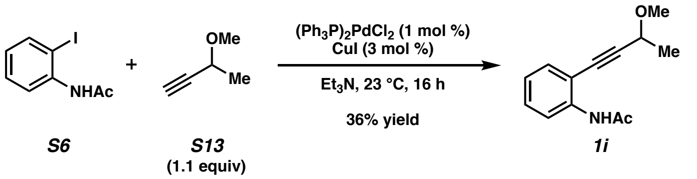


**Alkyne 1i.** To a mixture of 2-iodoacetanilide (**S6**, 300 mg, 1.15 mmol) in $Et_3N$ (3.48 mL) at 23 °C was added propargylic ether **S13** (107 mg, 1.27 mmol), and the mixture was degassed with argon via bubbling for 15 min. CuI (6.6 mg, 0.0345 mmol) was added, followed by $PdCl_2(PPh_3)_2$ (8.1 mg, 0.0115 mmol), and the resulting reaction mixture was stirred at 23 °C for 16 h. Upon completion, the reaction mixture was filtered through a $SiO_2$ plug (3 x 1.5 cm), eluting with EtOAc (10.0 mL). The filtrate was concentrated via rotary evaporation, and the resulting residue was purified by flash chromatography (1:1 hexanes/EtOAc eluent), providing alkyne **1i** (90.0 mg, 36% yield, $R_f$ = 0.25 in 1:1 hexanes/EtOAc) as light-orange solid.

**Data for Alkyne 1i.**
**$^1$H NMR** (400 MHz, $CDCl_3$): δ 8.38 (d, *J* = 8.5 Hz, 1H), 7.87 (br. s, 1H), 7.41 (d, *J* =7.5 Hz, 1H), 7.34 (app. t, *J* = 7.8 Hz, 1H), 7.04 (app. t, *J* = 7.5 Hz, 1H), 4.37 (q, *J* = 6.7 Hz, 1H), 3.49 (s, 3H), 2.22 (s, 3H), 1.57 (d, *J* = 6.7 Hz, 3H).
**$^{13}$C NMR** (100 MHz, $CDCl_3$): δ 168.2, 139.2, 131.8, 129.9, 123.4, 119.4, 111.3, 96.5, 80.5, 67.5, 56.6, 25.0, 22.3.
**IR** (film): 3248, 2981, 2926, 2818, 1665, 1536, 1210, 1115 $cm^{-1}$.
**HRMS** ($ESI^+$) *m/z* calc'd for $(M + Na)^+$ $[C_{13}H_{15}NO_2 + Na]^+$: 240.0995, found 240.0988.

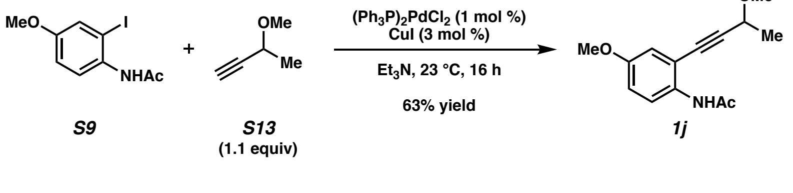


**Alkyne 1j.** To a mixture of 2-iodo-4-methoxyacetanilide (**S9**, 170 mg, 0.584 mmol) in $Et_3N$ (1.77 mL) at 23 °C was added propargylic ether **S13** (54.0 mg, 0.642 mmol), and the mixture was degassed with argon

via bubbling for 15 min. CuI (3.3 mg, 0.0175 mmol) was added, followed by $PdCl_2(PPh_3)_2$ (4.1 mg, 0.00584 mmol), and the resulting reaction mixture was stirred at 23 °C for 16 h. Upon completion, the reaction mixture was filtered through a $SiO_2$ plug (3 x 1.5 cm), eluting with EtOAc (6.0 mL). The filtrate was concentrated via rotary evaporation, and the resulting residue was purified by flash chromatography (1:1 hexanes/EtOAc eluent), providing alkyne **1j** (91.2 mg, 63% yield, $R_f$ = 0.28 in 1:1 hexanes/EtOAc) as a light-orange solid.

**Data for Alkyne 1j.**
**$^1$H NMR** (400 MHz, $CDCl_3$): δ 8.23 (d, $J$ = 9.1 Hz, 1H), 7.67 (br. s, 1H), 6.93 (s, 1H), 6.90 (d, $J$ = 9.1 Hz, H), 4.36 (q, $J$ = 6.7 Hz, 1H), 3.78 (s, 3H), 3.49 (s, 3H), 2.19 (s, 3H), 1.56 (d, $J$ = 6.7 Hz, 3H).
**$^{13}$C NMR** (100 MHz, $CDCl_3$): δ 167.9, 155.4, 132.9, 121.3, 116.4, 116.1, 112.8, 96.2, 80.6, 67.5, 56.7, 55.7, 24.8, 22.3.
**IR** (film): 3290, 2984, 2934, 1657, 1530, 1254, 1138, 871 $cm^{-1}$.
**HRMS** (ESI$^+$) *m/z* calc'd for $(M + Na)^+$ $[C_{14}H_{17}NO_3 + Na]^+$: 270.1101, found 270.1094.

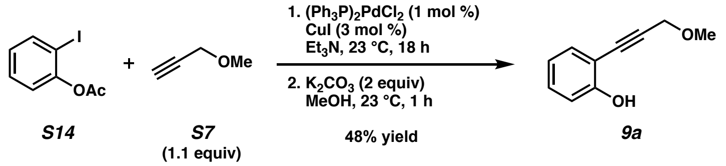


**Alkyne 9a.** To a mixture of 2-iodophenyl acetate (**S14**, 1.34 g, 5.11 mmol) in $Et_3N$ (15.5 mL) at 23 °C was added propargylic ether **S7** (394 mg, 5.63 mmol), and the mixture was degassed with argon via bubbling for 15 min. CuI (29.1 mg, 0.153 mmol) was added, followed by $PdCl_2(PPh_3)_2$ (35.9 mg, 0.0511 mmol), and the resulting reaction mixture was stirred for 18 h. Upon completion, the reaction mixture was filtered through a $SiO_2$ plug (3 x 1.5 cm), eluting with EtOAc (50 mL). The filtrate was concentrated via rotary evaporation, and the resulting residue was taken up in MeOH (25.6 mL). $K_2CO_3$ (1.41 g, 10.2 mmol) was added at 23 °C, and the reaction mixture was stirred for 1 h. Upon completion, the reaction mixture was diluted with $H_2O$ (25 mL) and extracted with EtOAc (3 x 40 mL). The combined organic layers were washed with brine (15 mL), dried over $MgSO_4$, and the solvent was removed by rotary evaporation. The resulting residue was purified by flash chromatography (4:1 hexanes/EtOAc eluent), providing alkyne **9a** (398 mg, 48% yield, $R_f$ = 0.29 in 4:1 hexanes/EtOAc) as a colorless oil. The spectroscopic data for alkyne **9a** were in accordance with the published values.[6]

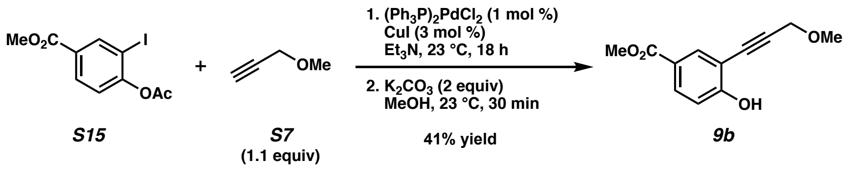


**Alkyne 9b.** To a mixture of methyl 4-acetoxy-3-iodobenzoate (**S15**, 750 mg, 2.34 mmol) in $Et_3N$ (7.09 mL) at 23 °C was added propargylic ether **S7** (180 mg, 2.57 mmol), and the mixture was degassed with argon via bubbling for 15 min. CuI (13.4 mg, 0.0702 mmol) was added, followed by $PdCl_2(PPh_3)_2$ (16.4 mg, 0.0234 mmol), and the resulting reaction mixture was stirred for 18 h. Upon completion, the reaction mixture was filtered through a $SiO_2$ plug (3 x 1.5 cm), eluting with EtOAc (25 mL). The filtrate was concentrated via rotary evaporation, and the resulting residue was taken up in MeOH (11.7 mL). $K_2CO_3$ (647 mg, 4.68 mmol) was added at 23 °C, and the reaction mixture was stirred for 30 min. Upon completion, the reaction mixture was diluted with $H_2O$ (15 mL) and extracted with EtOAc (3 x 15 mL). The combined organic layers were washed with brine (10 mL), dried over $MgSO_4$, and the solvent was

removed by rotary evaporation. The resulting residue was purified by flash chromatography (3:1 hexanes/EtOAc eluent), providing alkyne **9b** (212 mg, 41% yield, $R_f$ = 0.26 in 3:1 hexanes/EtOAc) as a colorless oil.

**Data for Alkyne 9b.**
**$^1$H NMR** (400 MHz, $CDCl_3$): δ 8.08 (s, 1H), 7.95 (d, *J* = 8.7 Hz, 1H), 6.99 (d, *J* = 8.7 Hz, 1H), 6.13 (s, 1H), 4.39 (s, 2H), 3.89 (s, 3H) 3.47 (s, 3H).
**$^{13}$C NMR** (100 MHz, $CDCl_3$): δ 166.2, 160.6, 134.3, 132.5, 122.8, 115.0, 109.3, 93.0, 79.7, 60.5, 58.1, 52.2.
**IR** (film): 3305, 2997, 2951, 2624, 1721, 1499, 1302, 1235, 1125, 1105 cm$^{-1}$.
**HRMS** ($ESI^+$) *m/z* calc'd for $(M + Na)^+$ $[C_{12}H_{12}O_4 + Na]^+$: 243.0628, found 243.0620.

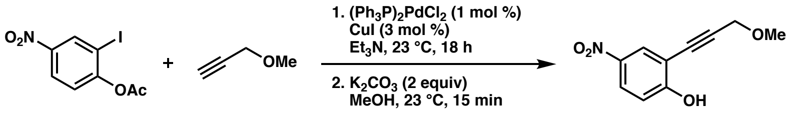


*S16* *S7* (1.1 equiv) 21% yield *9c*

**Alkyne 9c.** To a mixture of 2-iodo-4-nitrophenyl acetate (**S16**, 1.50 g, 4.88 mmol) in $Et_3N$ (14.8 mL) at 23 °C was added propargylic ether **S7** (376 mg, 5.37 mmol), and the mixture was degassed with argon via bubbling for 15 min. CuI (27.8 mg, 0.146 mmol) was added, followed by $PdCl_2(PPh_3)_2$ (34.3 mg, 0.0489 mmol), and the resulting reaction mixture was stirred for 18 h. Upon completion, the reaction mixture was filtered through a $SiO_2$ plug (3 x 1.5 cm), eluting with EtOAc (50 mL). The filtrate was concentrated via rotary evaporation, and the resulting residue was taken up in MeOH (24.4 mL). $K_2CO_3$ (1.35 g, 9.76 mmol) was added at 23 °C, and the reaction mixture was stirred for 15 min. Upon completion, the reaction mixture was diluted with $H_2O$ (25 mL) and extracted with EtOAc (3 x 40 mL). The combined organic layers were washed with brine (15 mL), dried over $MgSO_4$, and the solvent was removed by rotary evaporation. The resulting residue was purified by flash chromatography (3:1 hexanes/EtOAc eluent), providing alkyne **9c** (210 mg, 21% yield, $R_f$ = 0.20 in 3:1 hexanes/EtOAc) as a colorless oil.

**Data for Alkyne 9c.**
**$^1$H NMR** (400 MHz, $CDCl_3$): δ 8.29 (d, *J* = 2.7 Hz, 1H), 8.16 (dd, *J* = 9.1, 2.7 Hz, 1H), 7.05 (d, *J* = 9.1 Hz, 1H), 6.48 (br. s, 1H), 4.40 (s, 2H), 3.48 (s, 3H).
**$^{13}$C NMR** (100 MHz, $CDCl_3$): δ 161.9, 141.3, 128.3, 126.6, 115.6, 109.9, 94.5, 78.5, 60.4, 58.3.
**IR** (film): 3304, 2997, 2940, 2772, 1524, 1342, 1297, 1171, 1130, 1023 cm$^{-1}$.
**HRMS** ($ESI^+$) *m/z* calc'd for $(M + Na)^+$ $[C_{10}H_9NO_4 + Na]^+$: 230.0424, found 230.0417.

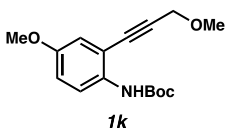


*1k*

**Alkyne 1k.** Prepared according to the procedure described by Allegretti and coworkers.[5]

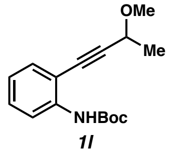


*1l*

**Alkyne 1l.** Prepared according to the procedure described by Saito and coworkers.[2]

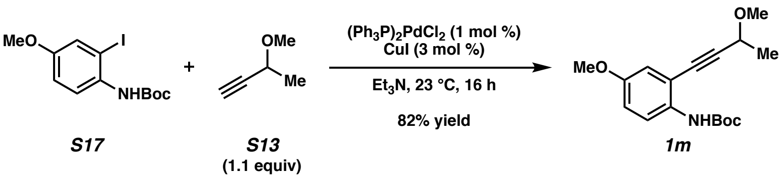


**Alkyne 1m.** To a mixture of aryl iodide **S17** (594 mg, 1.70 mmol) in $Et_3N$ (5.15 mL) at 23 °C was added propargylic ether **S13** (157 mg, 1.87 mmol), and the mixture was degassed with argon via bubbling for 15 min. CuI (9.7 mg, 0.0510 mmol) was added, followed by $PdCl_2(PPh_3)_2$ (11.9 mg, 0.0170 mmol), and the resulting reaction mixture was stirred at 23 °C for 16 h. After this time, the reaction mixture was filtered through a $SiO_2$ plug (3 x 1.5 cm), eluting with EtOAc (15 mL). The filtrate was concentrated via rotary evaporation, and the resulting residue was purified by flash chromatography (3:1 hexanes/EtOAc eluent), providing alkyne **1m** (427 mg, 82% yield, $R_f$ = 0.39 in 3:1 hexanes/EtOAc) as a colorless oil.

**Data for Alkyne 1m.**
**$^1$H NMR** (400 MHz, $CDCl_3$): δ 7.97 (br. d, *J* = 9.0 Hz, 1H), 7.01 (br. s, 1H), 6.92 – 6.85 (comp. m, 2H), 4.36 (q, *J* = 6.6 Hz, 1H), 3.76 (s, 3H), 3.49 (s, 3H), 1.56 (d, *J* = 6.6 Hz, 3H), 1.51 (s, 9H).
**$^{13}$C NMR** (100 MHz, $CDCl_3$): δ 154.6, 152.8, 133.5, 119.7, 116.3, 116.2, 112.0, 95.7, 80.8, 80.6, 67.5, 56.6, 55.7, 28.5, 22.2.
**IR** (film): 3411, 2981, 2935, 2360, 1731, 1519, 1212, 1158, 1022 $cm^{-1}$.
**HRMS** ($ESI^+$) *m/z* calc'd for $(M + Na)^+$ $[C_{17}H_{23}NO_4 + Na]^+$: 328.1519, found 328.1511.

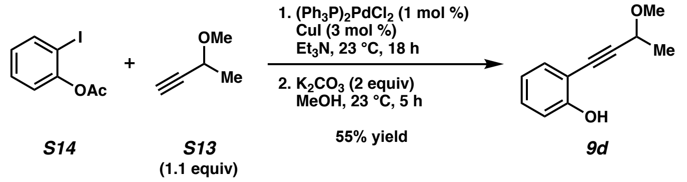


**Alkyne 9d.** To a mixture of 2-iodophenyl acetate (**S14**, 837 mg, 3.19 mmol) in $Et_3N$ (9.66 mL) at 23 °C was added propargylic ether **S13** (295 mg, 3.51 mmol), and the mixture was degassed with argon via bubbling for 15 min. CuI (18.2 mg, 0.0957 mmol) was added, followed by $PdCl_2(PPh_3)_2$ (22.4 mg, 0.0319 mmol), and the resulting reaction mixture was stirred for 18 h. After this time, the reaction mixture was filtered through a $SiO_2$ plug (3 x 1.5 cm), eluting with EtOAc (30 mL). The filtrate was concentrated via rotary evaporation, and the resulting residue was taken up in MeOH (16.0 mL). $K_2CO_3$ (882 mg, 6.38 mmol) was added at 23 °C, and the resulting mixture was stirred for 5 h. Upon completion, the mixture was diluted with $H_2O$ (20 mL) and extracted with EtOAc (3 x 20 mL). The combined organic layers were washed with brine (10 mL), dried over $MgSO_4$, and the solvent was removed by rotary evaporation. The resulting residue was purified by flash chromatography (3:1 hexanes/EtOAc eluent), providing alkyne **9d** (309 mg, 55% yield, $R_f$ = 0.55 in 3:1 hexanes/EtOAc) as a colorless oil.

**Data for Alkyne 9d.**
**$^1$H NMR** (400 MHz, $CDCl_3$): δ 7.34 (d, *J* = 7.7 Hz, 1H), 7.23 (app. t, *J* = 7.7 Hz, 1H), 6.95 (d, *J* = 7.7 Hz, 1H), 6.87 (app. t, *J* = 7.7 Hz, 1H), 5.78 (br. s, 1H), 4.35 (q, *J* = 6.6 Hz, 1H), 3.47 (s, 3H), 1.55 (d, *J* = 6.6 Hz, 3H).
**$^{13}$C NMR** (100 MHz, $CDCl_3$): δ 156.8, 132.0, 130.7, 120.5, 114.9, 109.0, 96.3, 79.4, 67.6, 56.7, 22.3.

**IR** (film): 3357, 2980, 2907, 2784, 1578, 1490, 1290, 1236, 1130, 774 $cm^{-1}$.
**HRMS** ($ESI^+$) *m/z* calc'd for $(M + Na)^+$ $[C_{11}H_{12}O_2 + Na]^+$: 199.0730, found 199.0727.

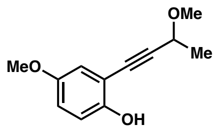


***9e***

**Alkyne 9e.** Prepared according to the procedure described by Allegretti and coworkers.[5]

# Preparation of Allylic Silane Starting Materials

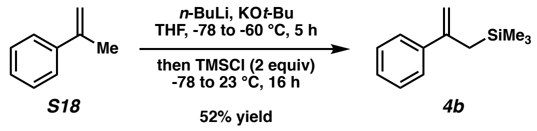


**Allylic trimethylsilane 4b.** Allylic trimethylsilane **4b** was synthesized according to the procedure described by Bauer and Maulide.[7] To a solution of KO*t*-Bu (2.60 g, 23.2 mmol) and α-methyl styrene (**S18**, 3.02 mL, 23.2 mmol) in THF (93 mL) at -78 °C was added *n*-BuLi (9.32 mL, 2.5 M in hexanes, 23.3 mmol) dropwise. The resulting mixture was stirred between -78 and -60 °C for 5 h. A solution of TMSCl (6.40 mL, 50.4 mmol) in THF (20 mL) was then added at -78 °C via syringe, and the resulting reaction mixture was allowed to warm to 23 °C and stirred for 16 h. After this time, the volatile materials were removed by rotary evaporation, and the resulting residue was vacuum filtered through a pad of silica, eluting with pentane (100 mL). The collected filtrate was concentrated under vacuo, and the residue was purified by flash chromatography (100% pentane eluent), providing allylic trimethylsilane **4b** (2.28 g, 52% yield, $R_f$ = 0.79 in 100% pentane) as a colorless oil. Observed spectroscopic data for allylic trimethylsilane **4b** were in accordance with reported data.[8]

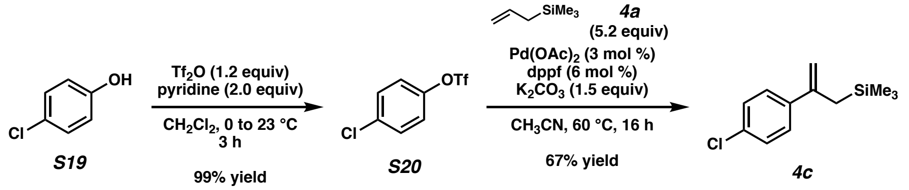


**Allylic trimethylsilane 4c.** Aryl triflate **S20** was synthesized according to the procedure described by Ishikawa and Manabe.[9] To a solution of *p*-chlorophenol (**S19**, 2.00 g, 15.6 mmol) in $CH_2Cl_2$ (31.1 mL) at 0 °C was added pyridine (2.52 mL, 31.1 mmol) and triflic anhydride (3.15 mL, 18.7 mmol) sequentially. The reaction mixture was allowed to warm to 23 °C and stirred for 3 h. After this time, the mixture was diluted with sat. aq. $NH_4Cl$ (15 mL) and extracted with $CH_2Cl_2$ (3 x 15 mL). The combined organic layers were washed with brine (10 mL), dried over $MgSO_4$, and the solvent was removed by rotary evaporation, affording aryl triflate **S17** (4.03 g, 99% yield, $R_f$ = 0.31 in 9:1 hexanes/EtOAc) as a pale yellow oil. The aryl triflate was sufficiently pure to be carried to the subsequent reaction. Observed spectroscopic data for aryl triflate **S20** were in accordance with reported data.[9]

Allylic trimethylsilane **4c** was synthesized according to a modified procedure of Hallberg and coworkers.[10] To a mixture of triflate **S20** (976 mg, 3.75 mmol), 1,1'-bis-(diphenylphosphino)ferrocene (125 mg, 0.225 mmol) and $Pd(OAc)_2$ (25.4 mg, 0.113 mmol) in $CH_3CN$ (9.36 mL) was added allyltrimethylsilane (**4a**, 3.11 mL, 19.6 mmol) and $K_2CO_3$ (800 mg, 5.79 mmol) sequentially. The resulting mixture was stirred at 60 °C for 16 h. After this time, the mixture was diluted with $H_2O$ (50 mL) and extracted with $Et_2O$ (3 x 30 mL). The combined organic layers were washed with brine (30 mL), dried over $MgSO_4$, and the solvent was removed by rotary evaporation. The resulting residue was purified by flash chromatography (25:1 hexanes/EtOAc eluent), providing allylic trimethylsilane **4c** (563 mg, 67% yield, $R_f$ = 0.78 in 9:1 hexanes/EtOAc) as colorless oil. Observed spectroscopic data for allylic trimethylsilane **4c** were in accordance with reported data.[11]

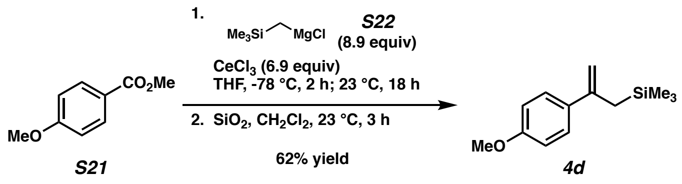

**Allylic trimethylsilane 4d.** Allylic trimethylsilane **4d** was synthesized according to a modification of the procedure described by Narayanan and Bunnelle.[12] Anhydrous $CeCl_3$ (10.3 g, 41.8 mmol) in THF (20.9 mL) was stirred at 23 °C under argon for 12 h. The resulting slurry was cooled to -78 °C, and $ClMgCH_2SiMe_3$ (**S22**, freshly generated from $ClCH_2SiMe_3$ (7.55 mL, 54.1 mmol) and magnesium turnings (1.31 g, 53.9 mmol)) in $Et_2O$ (27.0 mL) was added dropwise via addition funnel. The suspension was stirred at -78 °C for 2 h, then methyl 4-methoxybenzoate (**S21**, 1.01 g, 6.08 mmol) in THF (30.4 mL) was added dropwise over 5 min via syringe. The mixture was stirred at -78 °C for 2 h, then allowed to warm to 23 °C and stirred for 18 h. After this time, sat. aq. $NH_4Cl$ (180 mL) was added at 0 °C, and the mixture was extracted with $Et_2O$ (3 x 100 mL). The combined organic layers were washed with brine (10 mL), dried over $MgSO_4$, and the solvent was removed by rotary evaporation. The resulting residue was diluted with $CH_2Cl_2$ (20 mL) and stirred in silica gel (3 g) for 3 h. The mixture was filtered through a celite plug, and the filtrate was concentrated in vacuo. The resulting residue was purified by flash chromatography (19:1 hexanes/EtOAc eluent), providing allylic trimethylsilane **4d** (832 mg, 62% yield, $R_f$ = 0.41 in 19:1 hexanes/EtOAc) as a colorless oil. Observed spectroscopic data for allylic trimethylsilane **4d** were in accordance with reported data.[13]

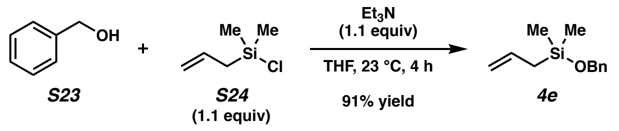

**Allylic silane 4e.** Allylic silane **4e** was synthesized according to a modification of the procedure reported by Wei and coworkers.[14] To a mixture of allyl(chloro)dimethylsilane (**S24**, 751 μL, 4.97 mmol) in THF (2.0 mL) at 23 °C was added a solution of benzyl alcohol (**S23**, 468 μL, 4.52 mmol) and $Et_3N$ (693 μL, 4.97 mmol) in THF (2.26 mL). The resulting reaction mixture was stirred at 23 °C for 4 h. After this time, the reaction mixture was filtered through a $SiO_2$ plug (3 x 1.5 cm), eluting with $Et_2O$ (30 mL), and the volatile materials from the filtrate were removed by rotary evaporation to afford allylic silane **4e** (849 mg, 91% yield, $R_f$ = 0.76 in 9:1 hexanes/EtOAc) as a colorless oil. The silane was sufficiently pure to be used without further purification. Observed spectroscopic data for allylic silane **4e** were in accordance with reported data.[14]

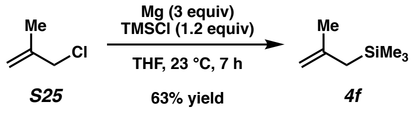

**Allylic trimethylsilane 4f**. Allylic trimethylsilane **4f** was synthesized according to a modification of the procedure described by Marković and coworkers.[15] To a mixture of Mg turnings (3.70 g, 152 mmol) in THF (50 mL) at 70 °C was added β-methallyl chloride (**S25**, 4.96 mL, 50.7 mmol) dropwise. A solution of TMSCl (7.72 mL, 60.8 mmol) in THF (122 mL) was next added dropwise via addition funnel while the reaction mixture was maintained at 70 °C, and the resulting mixture was stirred at 70 °C for 7 h. After this time, the reaction mixture was cooled to -20 °C, diluted with sat. aq. $NH_4Cl$ (100 mL), and extracted with $Et_2O$ (3 x 100 mL). The combined organic layers were washed with brine, dried over $MgSO_4$, and the

solvent was removed by rotary evaporation. The desired product was purified by distillation, providing allylic trimethylsilane **4f** (4.07 g, 63% yield; b.p. 110 °C at 760 torr) as a colorless oil. Observed spectroscopic data for allylic trimethylsilane **4f** were in accordance with reported data values.[16] (*Note*: a minor amount (<10%) of 2,5-dimethyl-1,5-hexadiene[17] was present in the isolated material.)

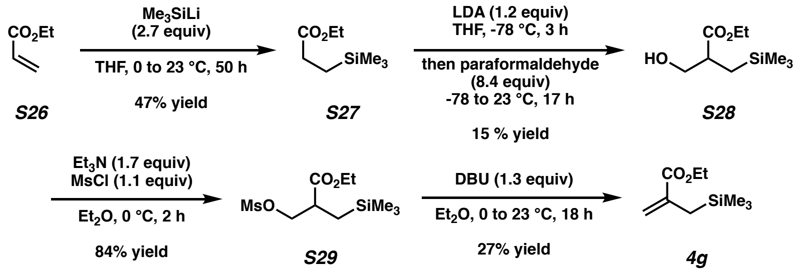


**Allylic trimethylsilane 4g.** Allylic trimethylsilane **4g** was synthesized according to a modification of the procedure described by Trost and Chan.[18] To a mixture of Li metal (1.08 g, 156 mmol) in THF (40 mL) at 0 °C was added TMSCl (19.0 mL, 150 mmol) dropwise, and the resulting mixture was stirred for 1 h at 0 °C. A solution of ethyl acrylate (**S26**, 6.00 mL, 55.0 mmol) in THF (40 mL) was then added dropwise over 2 h via additional funnel. The resulting reaction mixture was allowed to warm to 23 °C and stirred for 48 h. After this time, the mixture was filtered over celite by vacuum filtration, rinsing with $Et_2O$ (100 mL). The filtrate was washed sequentially with 1 M HCl (20 mL) and brine (10 mL), and then it was dried over $MgSO_4$. The solvent was removed by rotary evaporation, and the resulting residue was purified via distillation to afford trimethylsilane **S27** (4.49 g, 47% yield, b.p. 93 °C at 40 Torr) as a colorless oil. Observed spectroscopic data for trimethylsilane **S27** were in accordance with reported data.[19]

To a solution of *i*-$Pr_2NH$ (4.34 mL, 31.0 mmol) in THF (45 mL) at 0 °C was added *n*-BuLi (14.0 mL, 2.5 M in hexanes, 35.0 mmol) dropwise. The resulting mixture was stirred at 0 °C for 30 min, then cooled to -78 °C. Ester **S27** (4.49 g, 25.8 mmol) in THF (53 mL) was then added dropwise over 45 min via addition funnel, and the reaction mixture was stirred at -78 °C for 2 h. Paraformaldehyde (6.52 g, 217 mmol) was then added in one portion at -78 °C, and the resulting reaction mixture was allowed to warm to 23 °C and stirred for 17 h. After this time, the mixture was diluted with $H_2O$ (100 mL) and $Et_2O$ (100 mL). The layers were separated, and the aqueous layer was extracted with $Et_2O$ (2 x 100 mL). The combined organic layers were washed with brine (50 mL), dried over $MgSO_4$, and concentrated in vacuo to afford alcohol **S28** (797 mg, 15% yield, $R_f$ = 0.28 in 3:1 hexanes/EtOAc) as a yellow oil. The crude alcohol[18] was sufficiently pure to be used in the subsequent transformation.

To a mixture of alcohol **S28** (797 mg, 3.90 mmol) in $Et_2O$ (19.5 mL) at 0 °C was added $Et_3N$ (930 μL, 6.67 mmol), followed by MsCl (330 μL, 4.26 mmol) dropwise. The resulting mixture was stirred at 0 °C for 2 h. After this time, the reaction mixture was diluted with $Et_2O$ (20 mL) and washed sequentially with sat. aq. $CuSO_4$ (10 mL), $H_2O$ (10 mL), and brine (10 mL). The organic layer was dried over $MgSO_4$, and the solvent was removed by rotary evaporation to afford mesylate **S29** (922 mg, 84% yield, $R_f$ = 0.35 in 3:1 hexanes/EtOAc) as a yellow oil. The crude mesylate[18] was sufficiently pure to be used in the subsequent transformation.

To a mixture of mesylate **S29** (922 mg, 3.26 mmol) in $Et_2O$ (8.15 mL) at 0 °C was added DBU (633 μL, 4.23 mmol). The resulting mixture was allowed to warm to 23 °C and stirred for 18 h. After this time, the reaction mixture was diluted with $Et_2O$ (10 mL) and $H_2O$ (10 mL). The layers were separated, and the organic layer was washed with brine (10 mL), dried over $MgSO_4$, and concentrated in vacuo. The resulting residue was purified by flash chromatography (9:1 hexanes/EtOAc eluent) to afford allylic trimethylsilane **4g** (165 mg, 27% yield, $R_f$ = 0.57 in 9:1 hexanes/EtOAc) as a colorless oil. Observed spectroscopic data for allylic trimethylsilane **4g** were in accordance with reported data.[18]

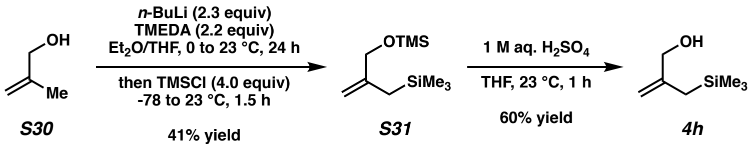

**Allylic trimethylsilane 4h**. Allylic silane **4h** was synthesized according to a modification of the procedure described by Migaud and coworkers.[20] To a solution of *n*-BuLi (65.2 mL, 2.5 M in hexanes, 163 mmol) in $Et_2O$ (54 mL) at 0 °C was added TMEDA (23.5 mL, 157 mmol). β-Methallyl alcohol (**S30**, 6.02 mL, 71.6 mmol) in THF (36 mL) was then added dropwise at 0 °C via syringe. The resulting mixture was allowed to warm to 23 °C and stirred for 24 h. The reaction mixture was then cooled to -78 °C, and TMSCl (36.2 mL, 285 mmol) was added rapidly. The resulting mixture was stirred for 30 min at -78 °C, then allowed to warm to 23 °C and stirred for 1 h. After this time, the reaction mixture was diluted with $Et_2O$ (400 mL) and sat. aq. $NaHCO_3$ (200 mL). The layers were separated, and the organic layer was washed sequentially with $H_2O$ (100 mL), sat. aq. $CuSO_4$ (2 x 100 mL), $H_2O$ (100 mL), and brine (100 mL). The organic layer was dried over $MgSO_4$, and the solvent was removed by rotary evaporation to afford allylic silane **S31** (6.39 g, 41% yield) as a yellow oil. The allylic silane was sufficiently pure to be carried on to the subsequent transformation. Observed spectroscopic data for allylic silane **S31** were in accordance with reported data.[20]

To a solution of allylic silane **S31** (6.39 g, 29.5 mmol) in THF (58 mL) at 23 °C was added 1 M aq. $H_2SO_4$ (11.8 mL). The resulting mixture was stirred vigorously for 1 h. Upon completion, as determined by TLC, the reaction mixture was neutralized to pH=6 with portionwise additions of $K_2CO_3$. The mixture was then diluted with $H_2O$ (50 mL) and $Et_2O$ (50 mL), and the layers were separated. The aqueous phase was extracted with $Et_2O$ (2 x 50 mL), and the combined organic layers were washed with brine (50 mL), dried over $MgSO_4$, and concentrated in vacuo. The crude product was purified by flash chromatography (4:1 hexanes/EtOAc eluent), providing allylic trimethylsilane **4h** (2.56 g, 60% yield, $R_f$ = 0.55 in 4:1 hexanes/EtOAc) as a colorless oil. Observed spectroscopic data for allylic trimethylsilane **4h** were in accordance with reported data.[20]

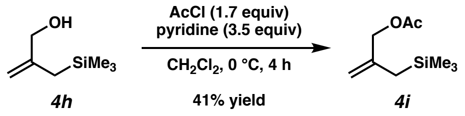

**Allylic trimethylsilane 4i.** Allylic acetate **4i** was synthesized according to the procedure described by Trost and Chan.[21] To a mixture of alcohol **4h** (750 mg, 5.20 mmol) in $CH_2Cl_2$ (6.0 mL) at 0 °C was added pyridine (1.47 mL, 18.2 mmol), followed by acetyl chloride (629 μL, 8.84 mmol) dropwise. The resulting mixture was stirred at 0 °C for 4 h. After this time, the reaction mixture was diluted with $Et_2O$ (25 mL) and washed with sat. aq. $NaHCO_3$ (5.0 mL). The organic layer was washed with brine (5.0 mL), dried over $MgSO_4$, and concentrated in vacuo. The crude product was purified by flash chromatography (9:1 hexanes/EtOAc eluent), providing allylic trimethylsilane **4i** (398 mg, 41% yield, $R_f$ = 0.54 in 9:1 hexanes/EtOAc) as a colorless oil. Observed spectroscopic data for allylic trimethylsilane **4i** were in accordance with reported data.[21]

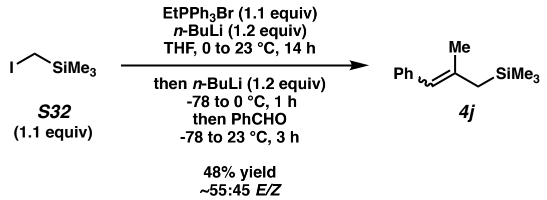


**Allylic trimethylsilane 4j.** Allylic trimethylsilane **4j** was synthesized according to a modified procedure of Seyferth and coworkers.[22] To a solution of ethyltriphenylphoshonium bromide (6.01 g, 16.2 mmol) in THF (30 mL) at 0 °C was added *n*-BuLi (7.00 mL, 2.5 M in hexanes, 17.5 mmol) dropwise. The reaction mixture was allowed to warm to 23 °C and stirred for 1 h. The resulting mixture was cooled back to 0 °C, and (trimethysilyl)methyl iodide (**S32**, 2.40 mL, 16.2 mmol) was added. The reaction mixture was allowed to warm to 23 °C and stirred for 14 h. The resulting mixture was cooled to -78 °C, and *n*-BuLi (7.00 mL, 2.5 M in hexanes, 17.5 mmol) was added. The mixture was warmed to 0 °C and stirred for 1 h. The resulting mixture was then recooled to -78 °C, and benzaldehyde (1.50 mL, 14.8 mmol) was added dropwise. The reaction mixture was allowed to warm to 23 °C and stirred for 3 h. After this time, the mixture was diluted with sat. aq. $NH_4Cl$ (150 mL) and extracted with $Et_2O$ (3 x 100 mL). The combined organic layers were washed with brine (10 mL), dried over $MgSO_4$, and the solvent was removed by rotary evaporation. The resulting residue was purified by flash chromatography (99:1 hexanes/EtOAc eluent), providing allylic trimethylsilane **4j** (1.46 g, 48% yield, ~55:45 *E/Z*, $R_f$ = 0.77 in 99:1 hexanes/EtOAc) as a colorless oil.

**Data for allylic trimethylsilane 4j.**
**$^1$H NMR** (400 MHz, $CDCl_3$) (*E*)-*Isomer*: δ 7.34 – 7.27 (comp. m, 2H), 7.21 (app. d, *J* = 7.3 Hz, 2H), 7.15 (app. t, *J* = 7.3 Hz, 1H), 6.11 (s, 1H), 1.89 – 1.87 (comp. m, 3H), 1.86 (s, 2H), 0.08 (s, 9H); (*Z*)-*Isomer*: δ 7.34 – 7.27 (comp. m, 2H), 7.21 (app. d, *J* = 7.3 Hz, 2H), 7.15 (app. t, *J* = 7.3 Hz, 1H), 6.16 (s, 1H), 1.89 – 1.87 (comp. m, 3H), 1.70 (s, 2H), 0.03 (s, 9H).
**$^{13}$C NMR** (100 MHz, $CDCl_3$, mixture of isomers): δ 139.4, 139.3, 137.9, 137.7, 128.8, 128.6, 128.2, 128.1, 125.6, 125.5, 123.1, 122.8, 31.8, 27.6, 23.9, 20.7, -0.4, -1.0.
**IR** (film): 3022, 2954, 1755, 1640, 1492, 1249, 843 $cm^{-1}$.
**LRMS** (EI) *m/z* calc'd for (M) [$C_{13}H_{20}Si$]: 204, found 204. (*Note*: This compound failed to be detected successfully by HRMS techniques.)

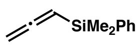


***14a***

**Allenyldimethylphenylsilane (14a).** Prepared according to the procedure described by Li and coworkers.[23] Spectroscopic data were in accordance with reported data.[24]

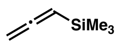


***14b***

**Allenyltrimethylsilane (14b).** Prepared according to the procedure described by Li and coworkers.[23] Spectroscopic data were in accordance with reported data.[24]

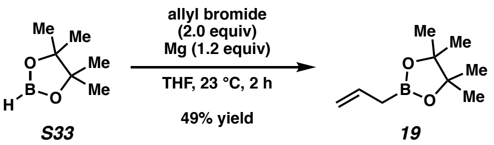


**Allyl boronate 19.** Allyl boronate **19** was synthesized according to the procedure described by Wang and Hadjichristidis.[25] To a mixture of magnesium turnings (1.01 g, 41.5 mmol) and pinacolborane (**S33**, 5.02 mL, 34.6 mmol) in THF (58.0 mL) at 23 °C was added allyl bromide (2.99 mL, 34.6 mmol) dropwise. The resulting mixture was stirred at 23 °C for 15 min. Another amount of allyl bromide (2.99 mL, 34.6 mmol, 5.98 mL added total) was added dropwise, and the resulting mixture was stirred at 23 °C for 2 h. The reaction mixture was then diluted with hexanes (60 mL) and 1 M aq. HCl (60 mL), and the resulting mixture was stirred for 10 min. The layers were separated, and the aqueous layer was extracted with hexanes (2 x 60 mL). The combined organic layers were washed sequentially with $H_2O$ (25 mL) and brine (25 mL), dried over $MgSO_4$, and concentrated in vacuo. The resulting residue was purified via distillation to afford allyl boronate **19** (2.87 g, 49% yield, b.p. 25 °C at 1 torr) as a colorless oil. Observed spectroscopic data for allyl boronate **19** were in accordance with reported data.[26]

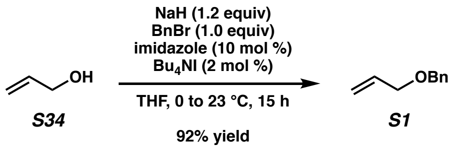


**Benzyl allyl ether (S1).** Benzyl allyl ether (**S1**) was synthesized according to a modified procedure of Nicolaou and coworkers.[27] To a mixture of allyl alcohol (**S34**, 3.00 mL, 44.1 mmol) in THF (44.0 mL) at 0 °C was added NaH (2.12 g, 60% dispersion in mineral oil, 52.9 mmol). The resulting mixture was stirred at 0 °C for 1 h. Imidazole (300 mg, 4.41 mmol) and tetrabutylammonium iodide (326 mg, 0.882 mmol) were then added, followed by benzyl bromide (5.25 mL, 44.1 mmol) dropwise. The reaction mixture was allowed to warm to 23 °C and stirred for 15 h. After this time, the mixture was diluted with $H_2O$ (50 mL) and extracted with $Et_2O$ (3 x 50 mL). The combined organic layers were washed with brine (50 mL), dried over $MgSO_4$, and concentrated in vacuo. The resulting residue was purified by flash chromatography (9:1 hexanes/EtOAc eluent) to afford benzyl ether **S1** (6.01 g, 92% yield, $R_f = 0.57$ in 9:1 hexanes/EtOAc) as a colorless oil. Observed spectroscopic data for benzyl ether **S1** were in accordance with reported data.[27]

# Additional Starting Material Syntheses

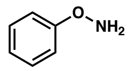

*S3*

**Phenoxyamine (S3).** Prepared according to the procedure described by Petrassi and coworkers.[28]

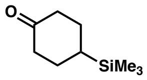

*S4*

**Cyclohexanone S4.** Prepared according to the procedure described by Cren and coworkers.[29]

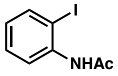

*S6*

**2-Iodoacetanilide (S6).** Prepared according to the procedure described by Pialat and coworkers.[30]

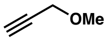

*S7*

**Propargylic ether S7.** Prepared according to the procedure described by Mames and coworkers.[31]

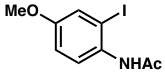

*S9*

**2-Iodo-4-methoxyacetanilide (S9).** Prepared according to the procedure described by Wang and Frankowski.[32]

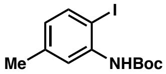

*S10*

***N*-Boc aniline S10.** Prepared according to the procedure described by Zehr and coworkers.[33]

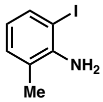

*S11*

**2-iodo-6-methylaniline (S11).** Prepared according to the procedure described by Shen and Vollhardt.[34]

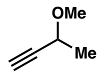


***S13***

**Propargylic ether S13.** Prepared according to the procedure described by Bell and coworkers.[35]

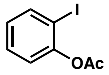


***S14***

**2-Iodophenyl acetate (S14).** Prepared according to the procedure described by Liu and Ma.[36]

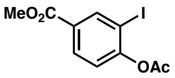


***S15***

**Methyl 4-acetoxy-3-iodobenzoate (S15).** Prepared according to the procedure described by Hirner and coworkers.[37]

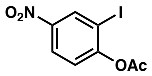


***S16***

**2-Iodo-4-nitrophenyl acetate (S16).** Prepared according to the procedure described by Vojtičková and coworkers.[38]

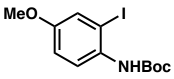


***S17***

**Aryl iodide S17.** Prepared according to the procedure described by Kondo and coworkers.[39]

# References

[1] Contiero, F.; Jones, K. M.; Matts, E. A.; Porzelle, A.; Tomkinson, N. C. O. *Synlett* **2009**, *18*, 3003-3006.
[2] Saito, K.; Sogou, H.; Suga, T.; Kusama, H.; Iwasawa, N. *J. Am. Chem. Soc.* **2011**, *133*, 689-691.
[3] Rudisill, D. E.; Stille, J. K. *J. Org. Chem.* **1989**, *54,* 5856–5866.
[4] Shen, Z.; Lu, X. *Adv. Synth. Catal*. **2009**, *351*, 3107-3112.
[5] Allegretti, P. A.; Huynh, K.; Ozumerzifon, T. J.; Ferreira. E. M. *Org. Lett*. **2016**, 18, 64-67.
[6] Nan, Y.; Miao, H; Yang, Z. *Org. Lett.* **2000**, *2,* 297–299.
[7] Bauer, A.; Maulide, N. *Org. Lett*. **2018**, *20*, 1461–1464.
[8] Mo, J.; Xu, L.; Xiao, J. *J. Am. Chem. Soc*. **2005**, *127*, 751–760.
[9] Ishikawa, S.; Manabe, K. *Tetrahedron* **2010**, *66,* 297–303.
[10] Olofsson, K.; Larhed, M.; Hallberg, A. *J. Org. Chem*. **1998**, *63*, 5076–5079.
[11] Hou, Z.-L.; Yang, F.; Zhou, Z.; Ao, Y.-F.; Yao, B. *Tetrahedron Lett*. **2018**, *59*, 4557–4561.
[12] Narayanan, B. A.; Bunnelle, W. H. *Tetrahedron Lett*. **1987**, *28*, 6261–6264.
[13] Shimizu, R.; Egami, H.; Hamashima, Y.; Sodeoka, M. *Angew. Chem. Int. Ed.* **2012**, *51*, 4577–4580.
[14] Wei, Z. Y.; Wang, D.; Li, J. S.; Chan, T. H. *J. Org. Chem*. **1989**, *54*, 5768–5774.
[15] Marković, D.; Tchawou, W. A.; Novosjolova, I.; Laclef, S.; Stepanovs, D.; Turks, M.; Vogel, P. *Chem. Eur. J.* **2016**, *22*, 4196–4205.
[16] (a) Ito, S.; Hayashi, A.; Komai, H.; Yamaguchi, H.; Kubota, Y.; Asami, M. *Tetrahedron* **2011**, *67,* 2081–2089; (b) Carr, S. A.; Weber, W. P. *J. Org. Chem*. **1985**, *50*, 2782–2785.
[17] Semmelhack, M. F.; Helquist, P. M. *Org. Synth*. **1972**, *52*, 115–121.
[18] Trost, B. M.; Chan, D. M. T. *J. Am. Chem. Soc*. **1983**, *105*, 2326–2335.
[19] Takeuchi, R.; Ishii, N.; Sugiura, N.; Sato, N. *J. Org. Chem.* **1992**, *57,* 4189–4194.
[20] Redpath, P.; Macdonald, S.; Migaud, M. E. *Org. Lett*. **2008**, *10*, 3323–3326.
[21] Trost, B. M.; Chan, D. M. T. *J. Am. Chem. Soc*. **1983**, *105*, 2315–2325.
[22] Seyferth, D.; Wursthorn, K. R.; Lim, T. F. O.; Sepelak, D. J. *J. Organomet. Chem*. **1979**, *181*, 293–304.
[23] Li, Z.; Yang, C.; Zheng, H.; Qiu, H.; Lai, G. *J. Organomet. Chem*. **2008**, *693*, 3771–3779.
[24] Fandrick, D. R.; Reeves, J. T.; Tan, Z.; Lee, H.; Song, J. J.; Yee, N. K.; Senanayake, C. H. *Org. Lett.* **2009**, *11*, 5458–5461.
[25] Wang, D.; Hadjichristidis, N. *Chem. Commun.* **2017**, *53*, 1196–1199.
[26] Mao, L.; Szabó, K. J.; Marder, T. B. *Org. Lett*. **2017**, *19*, 1204–1207.
[27] Nicolaou, K. C.; Patron, A. P.; Ajito, K.; Richter, P. K.; Khatuya, H.; Bertinato, P.; Miller, R. A.; Tomaszewski, M. J. *Chem. Eur. J.* **1996**, *2*, 847–868.
[28] Petrassi, H. M.; Sharpless, K. B.; Kelly, J. W. *Org. Lett.* **2001**, *3*, 139-142.
[29] Cren, S.; Schär, P.; Renaud, P.; Schenk, K. *J. Org. Chem.* **2009**, *74*, 2942-2946.
[30] Pialat, A.; Liégault, B.; Taillefer, M. *Org. Lett.* **2013**, *15,* 1764–1767.
[31] Mames, A.; Stecko, S.; Mikołajczyk, P.; Soluch, M.; Furman, B.; Chmielewski, M. *J. Org. Chem.* **2010**, *75,* 7580–7587.
[32] Wang, F.; Frankowski, K. J. *Eur. J. Org. Chem.* **2022**, e202201153.
[33] Zehr, P. S.; Kayali, R.; Peña-Cabrera, E.; Robles-Resendiz, O.; Villanueva-Rendon, A. D.; Söderberg, B. C. G. *Tetrahedron* **2008**, *64*, 5336–5344.
[34] Shen, H.; Vollhardt, K. *Synlett* **2012**, *23*, 208–214.
[35] Bell, A.; Davidson, A. H.; Earnshaw, C.; Norrish, H. K.; Torr, R. S.; Trowbridge, D. B.; Warren, S. *J. Chem. Soc., Perkin Trans. 1* **1983**, 2879–2891.
[36] Liu, Y.; Ma, S. *Org. Lett*. **2012**, *14*, 720–723.
[37] Hirner, J. J.; Faizi, D. J.; Blum, S. A. *J. Am. Chem. Soc.* **2014**, *136,* 4740−4745.
[38] Vojtičková, M.; Dobiaš, J.; Hanquet, G.; Addová, G.; Cetin-Atalay, R.; Yildirim, D. C.; Boháč, A. *Eur. J. Med. Chem*. **2015**, *103,* 105–122.
[39] Kondo, Y.; Kojima, S.; Sakamoto, T. *J. Org. Chem*. **1997**, *62*, 6507–6511.

## The Dichotomous Behavior of Allylsilanes in the Additions to Platinum α,β-Unsaturated Carbenes

# $^{1}$H and $^{13}$C NMR Spectra Compilation

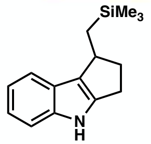

***5aa´***
**(from Boc removal)**
**$^1$H NMR**
**(400 MHz, $CDCl_3$)**

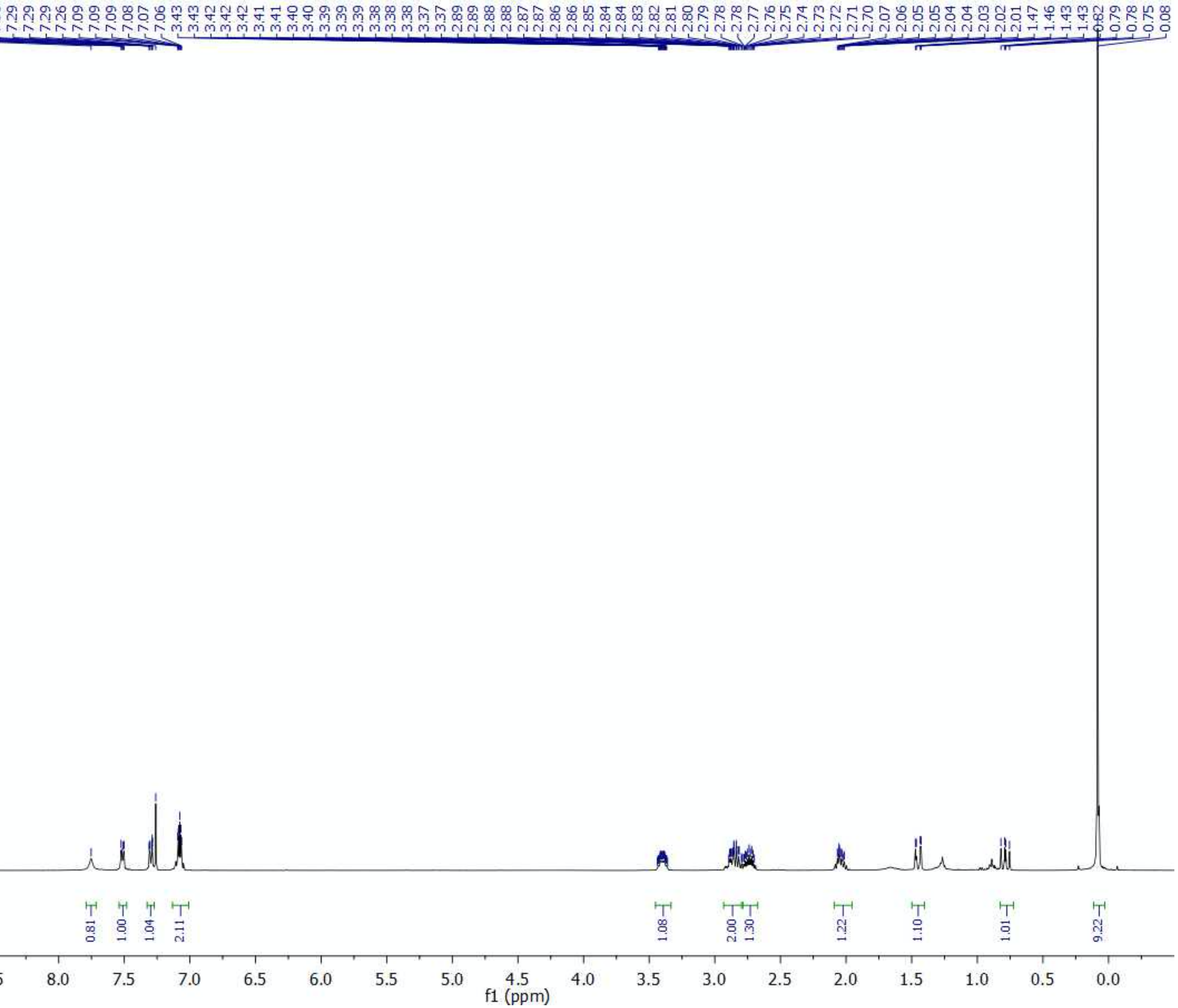

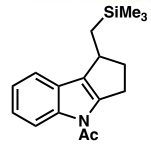


***5ba***
$^{1}$H NMR
(400 MHz, $CDCl_3$)

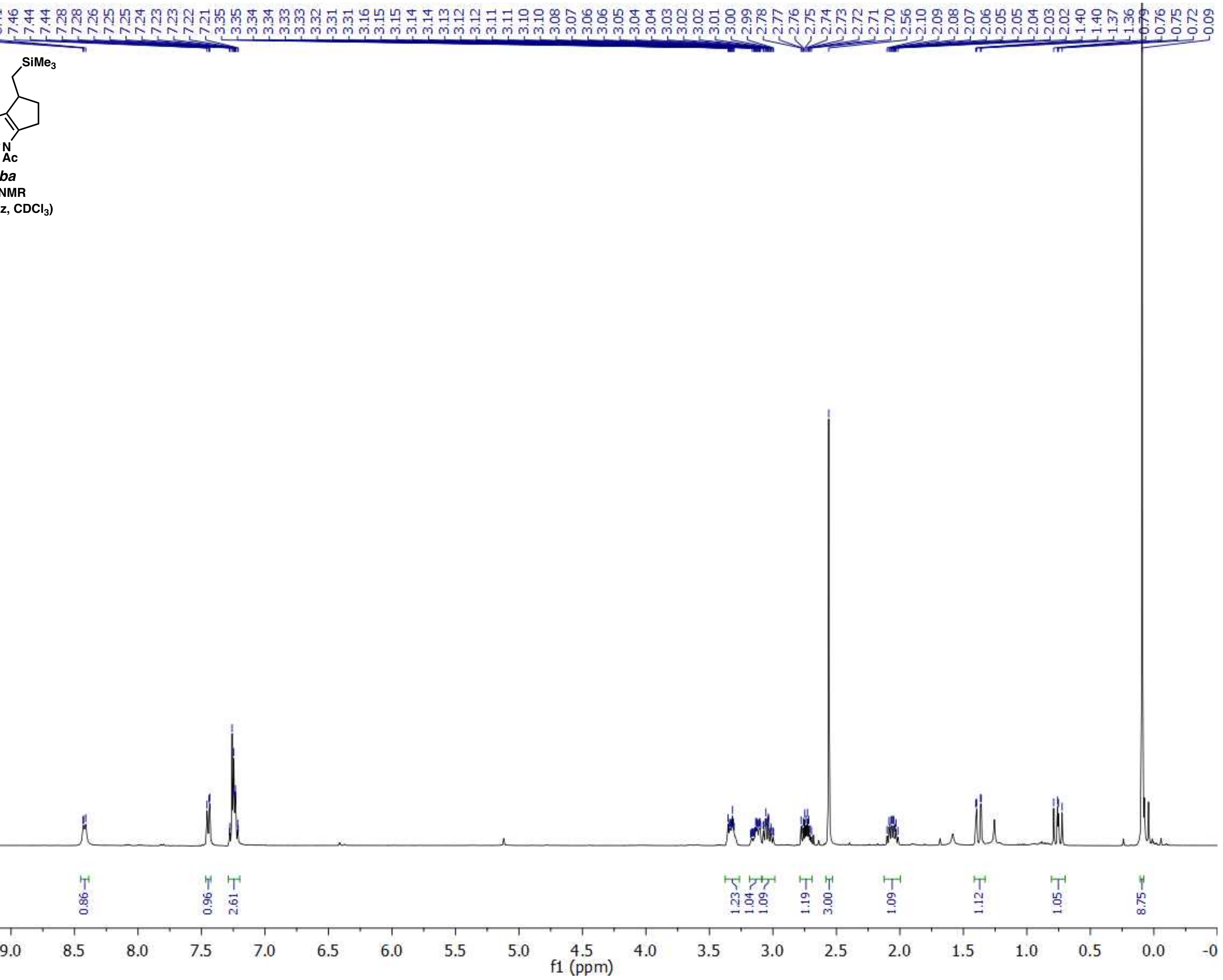

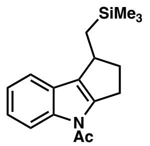


***5ba***
$^{13}$C NMR
(100 MHz, $CDCl_3$)

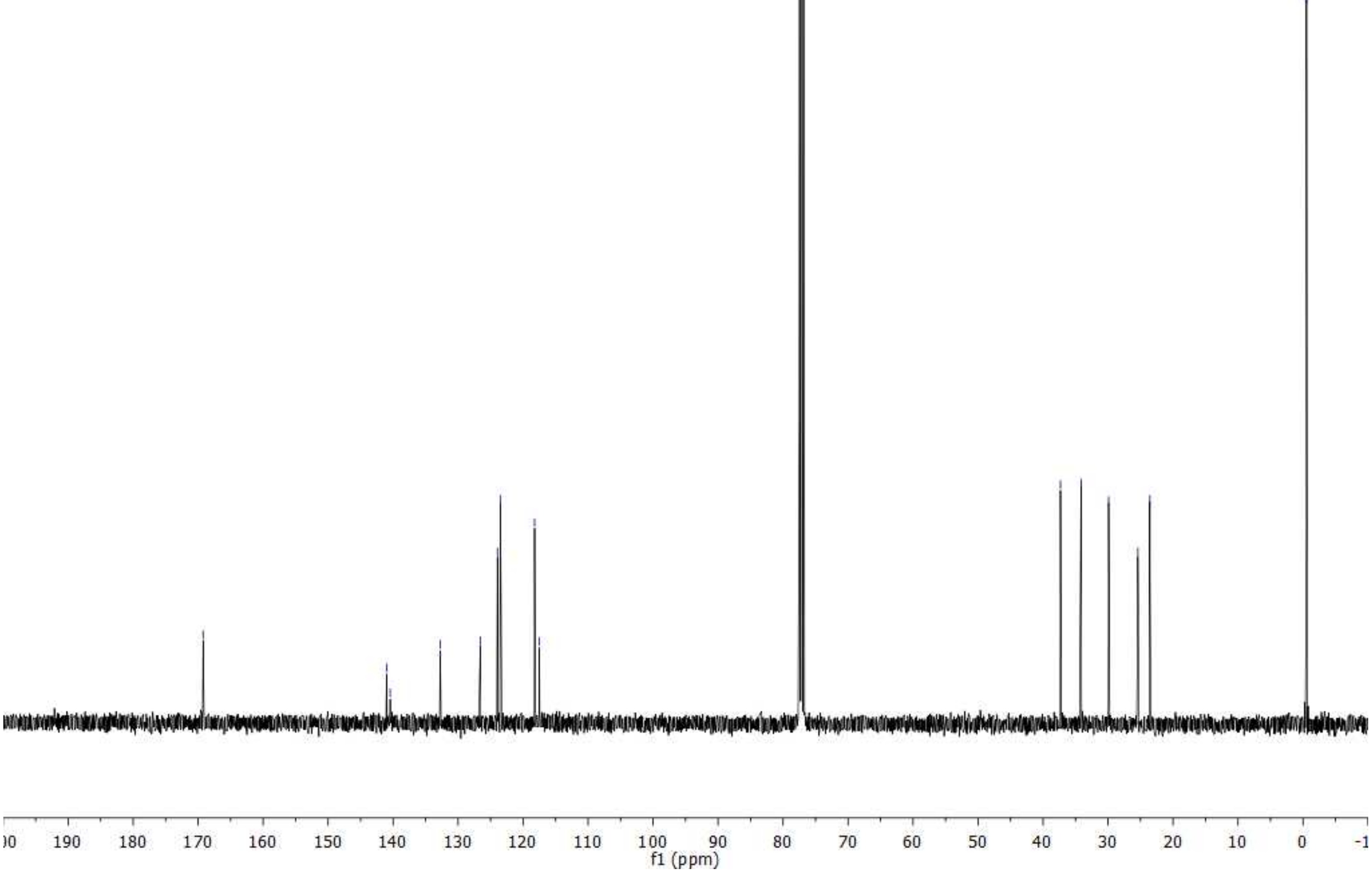

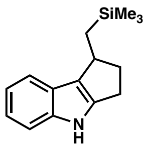


***5ca´***
**(from detosylation)**
**$^{1}$H NMR**
**(400 MHz, $CDCl_3$)**

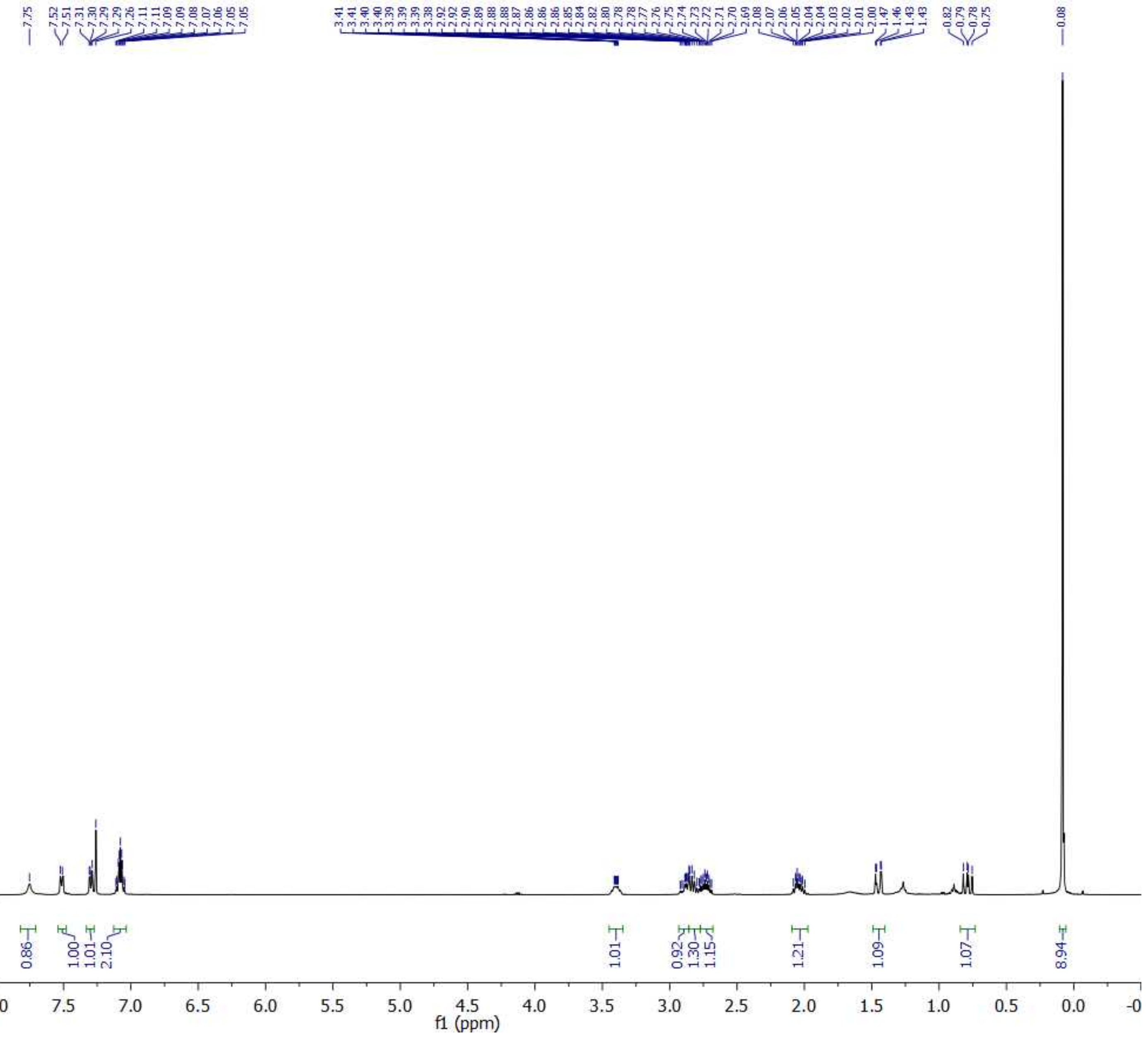

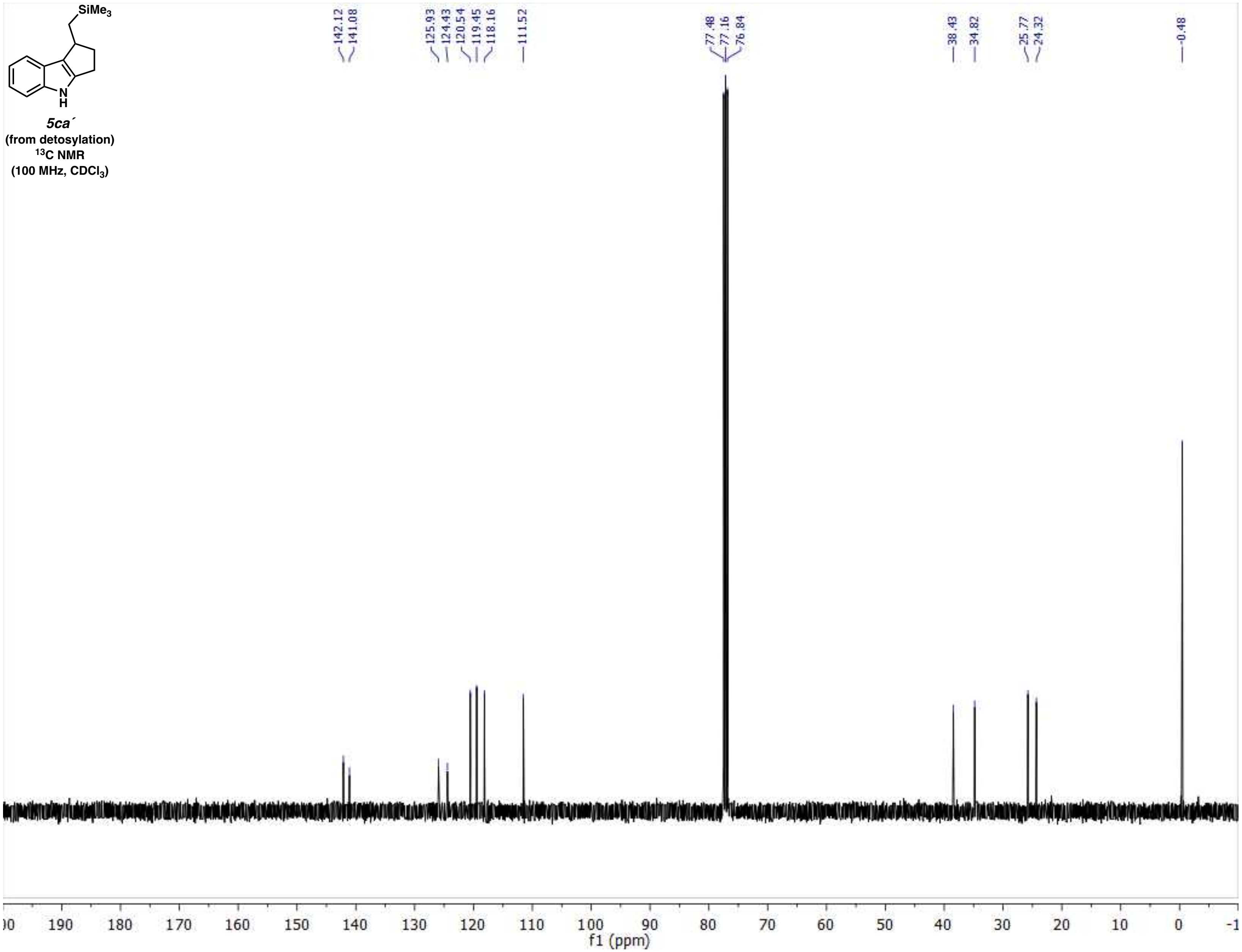
SiMe3
N
H
5ca´
(from detosylation)
13C NMR
(100 MHz, CDCl3)
142.12
141.08
125.93
124.43
120.54
119.45
118.16
111.52
77.48
77.16
76.84
38.43
34.82
25.77
24.32
-0.48
f1 (ppm)

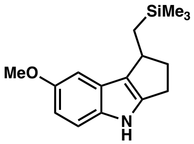

**5ea´**
**(from deacetylation)**
**$^{1}$H NMR**
**(400 MHz, $CDCl_3$)**

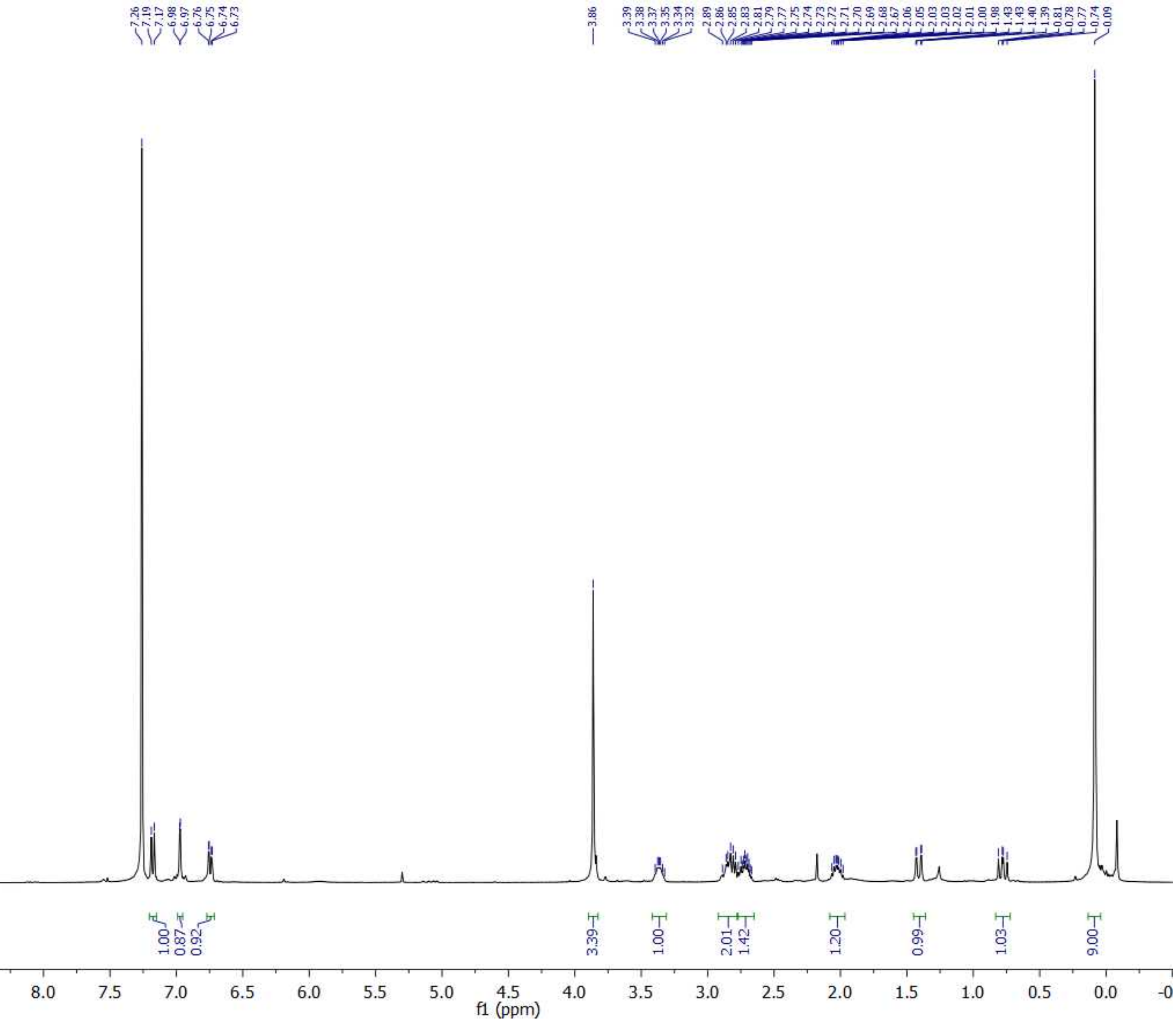

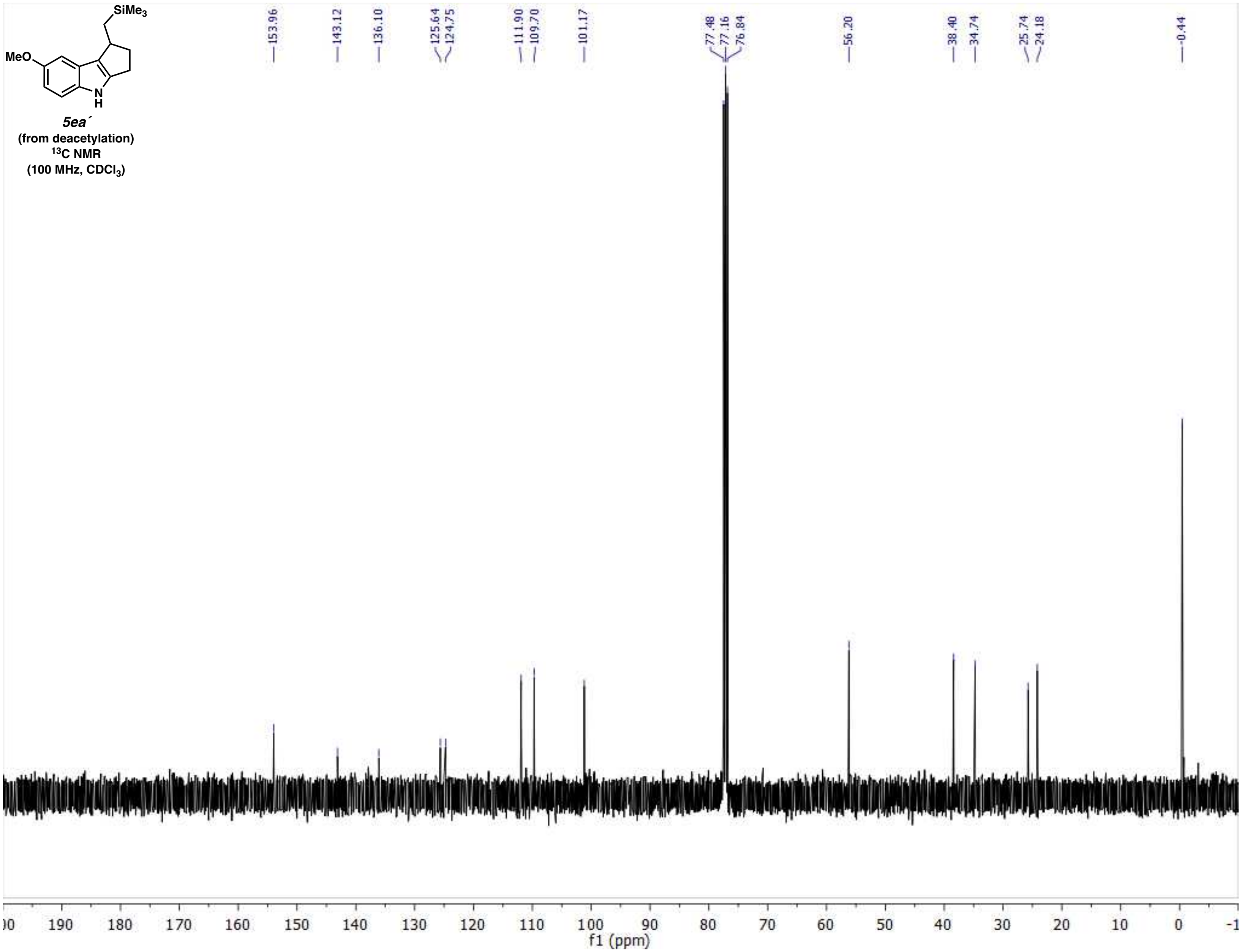
SiMe3
MeO
N
H
5ea´
(from deacetylation)
13C NMR
(100 MHz, CDCl3)
153.96
143.12
136.10
125.64
124.75
111.90
109.70
101.17
77.48
77.16
76.84
56.20
38.40
34.74
25.74
24.18
-0.44
f1 (ppm)

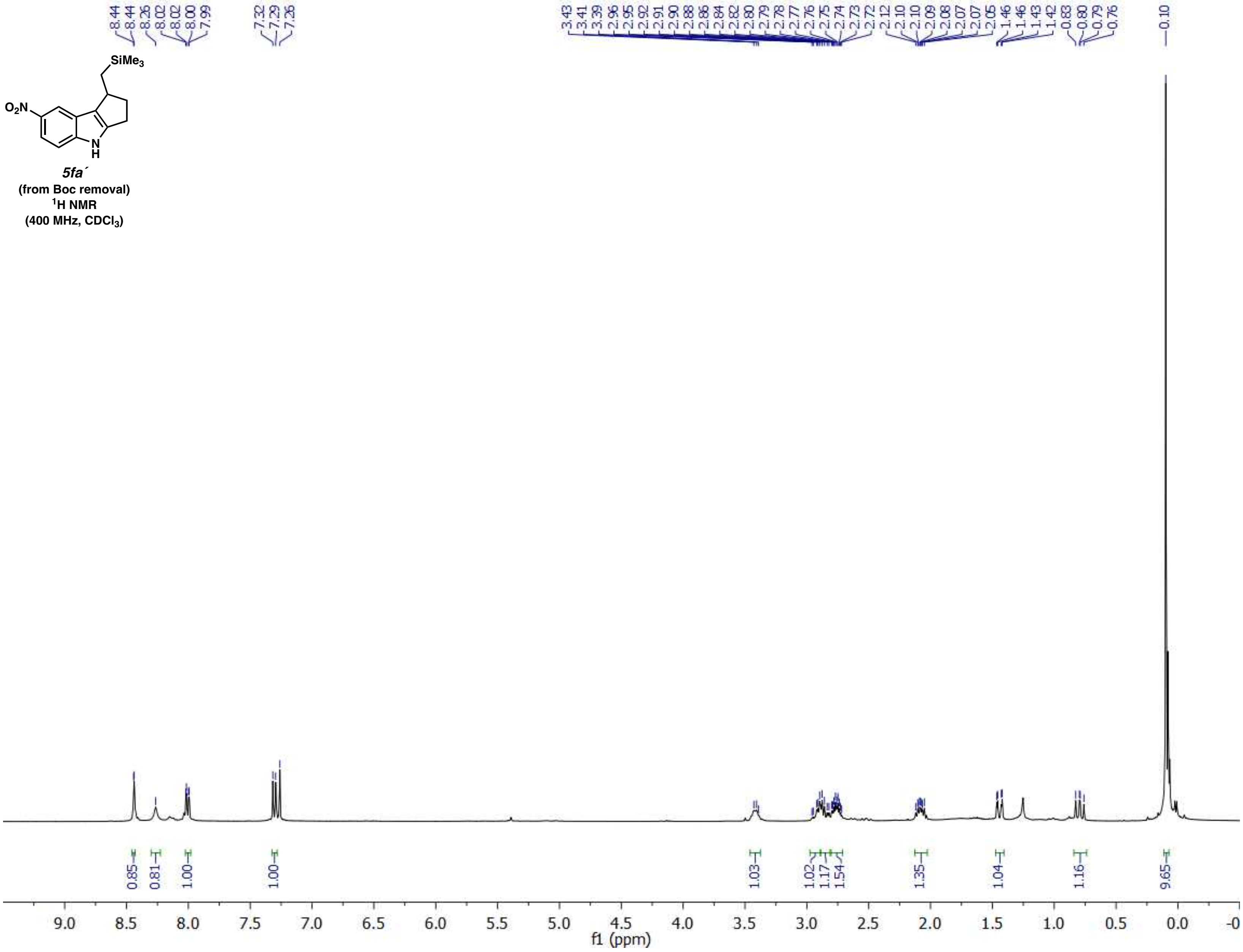

SiMe3
O2N
N
H
5fa´
(from Boc removal)
1H NMR
(400 MHz, CDCl3)
f1 (ppm)

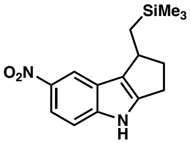


***5fa´***
**(from Boc removal)**
**$^{13}$C NMR**
**(100 MHz, $CDCl_3$)**

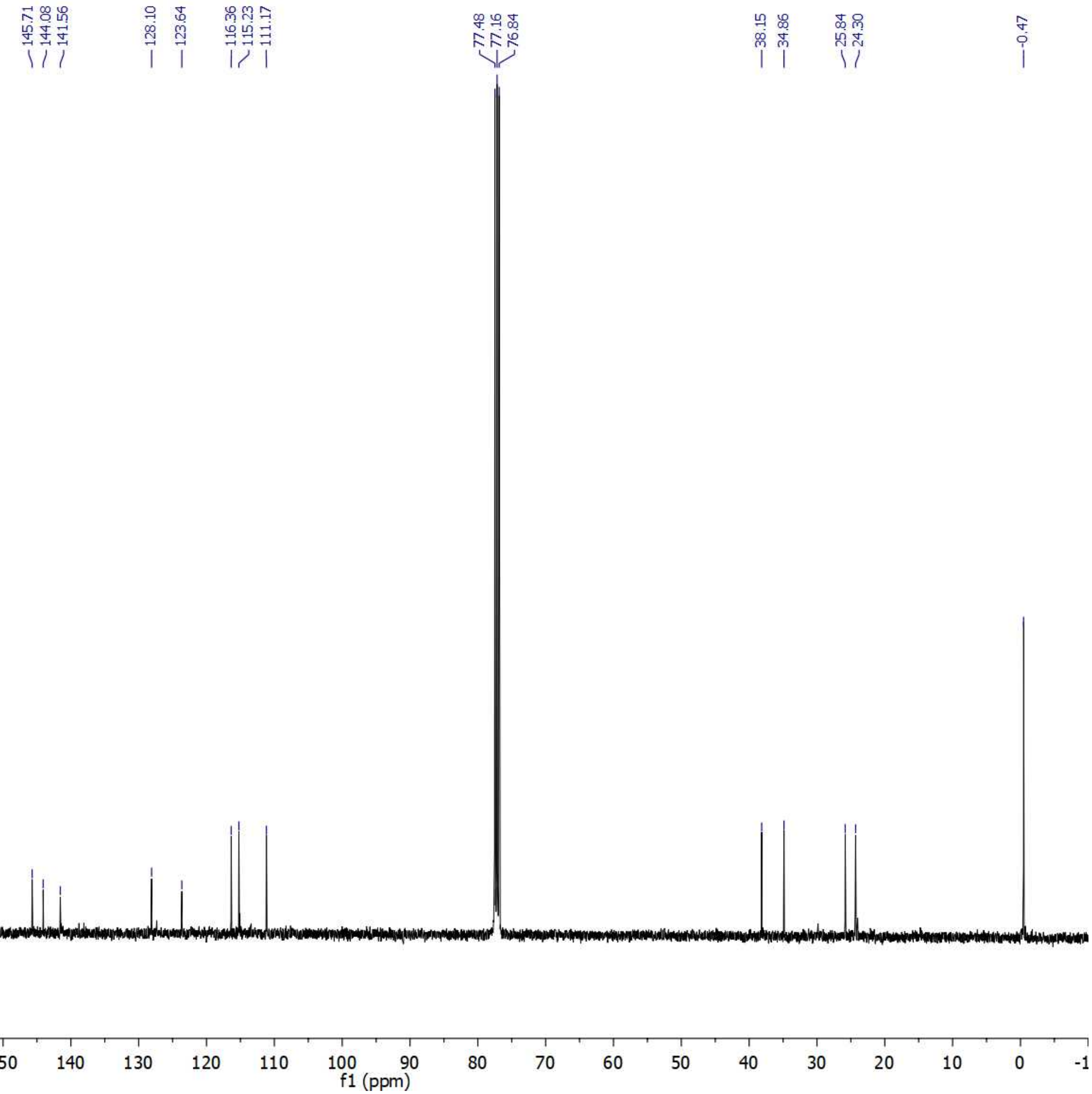

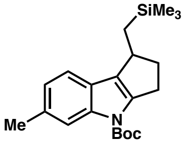


***5ga***
$^1$H NMR
(400 MHz, $CDCl_3$)

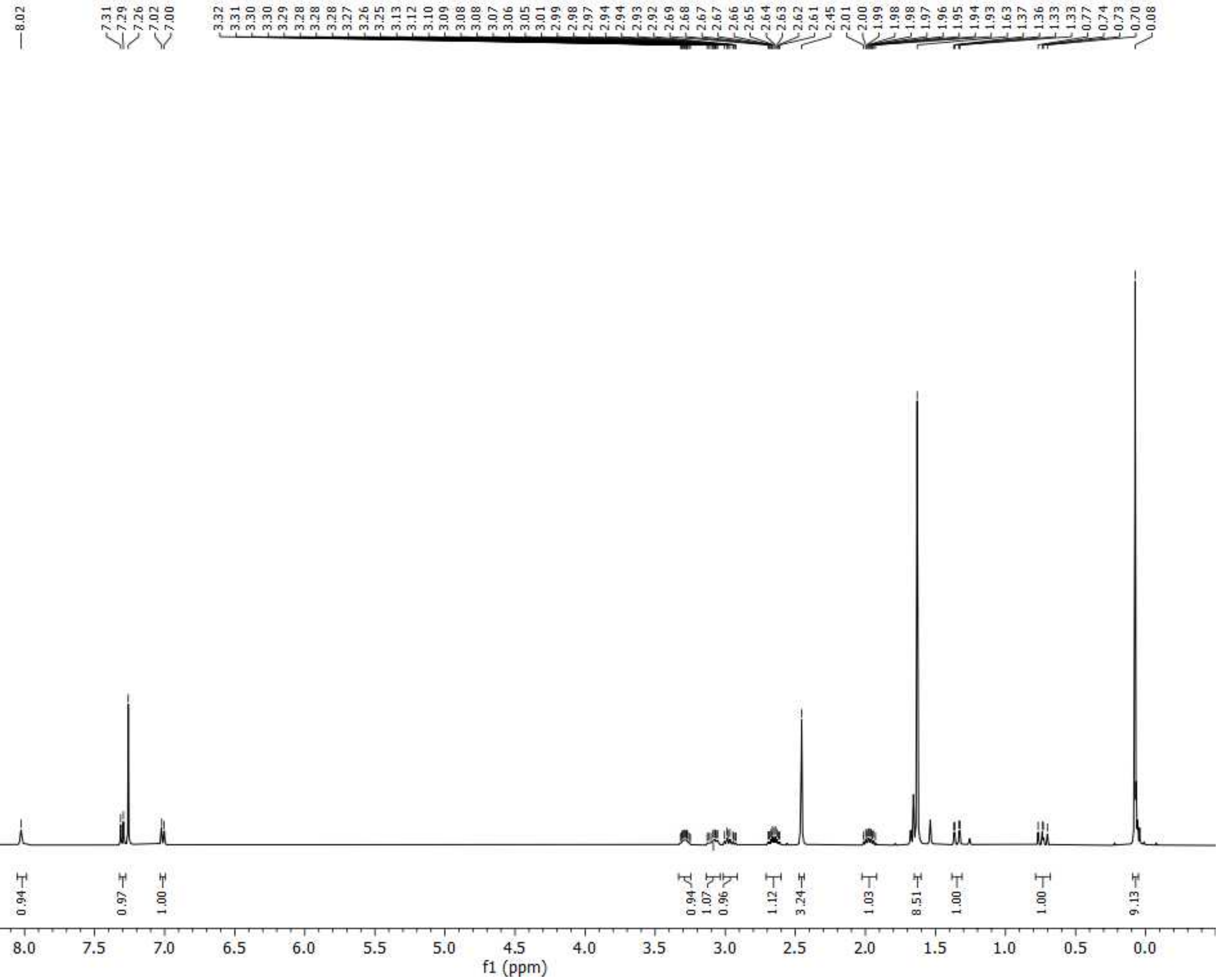

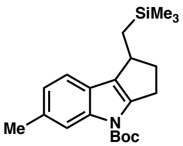


***5ga***
$^{13}$C NMR
(100 MHz, $CDCl_3$)

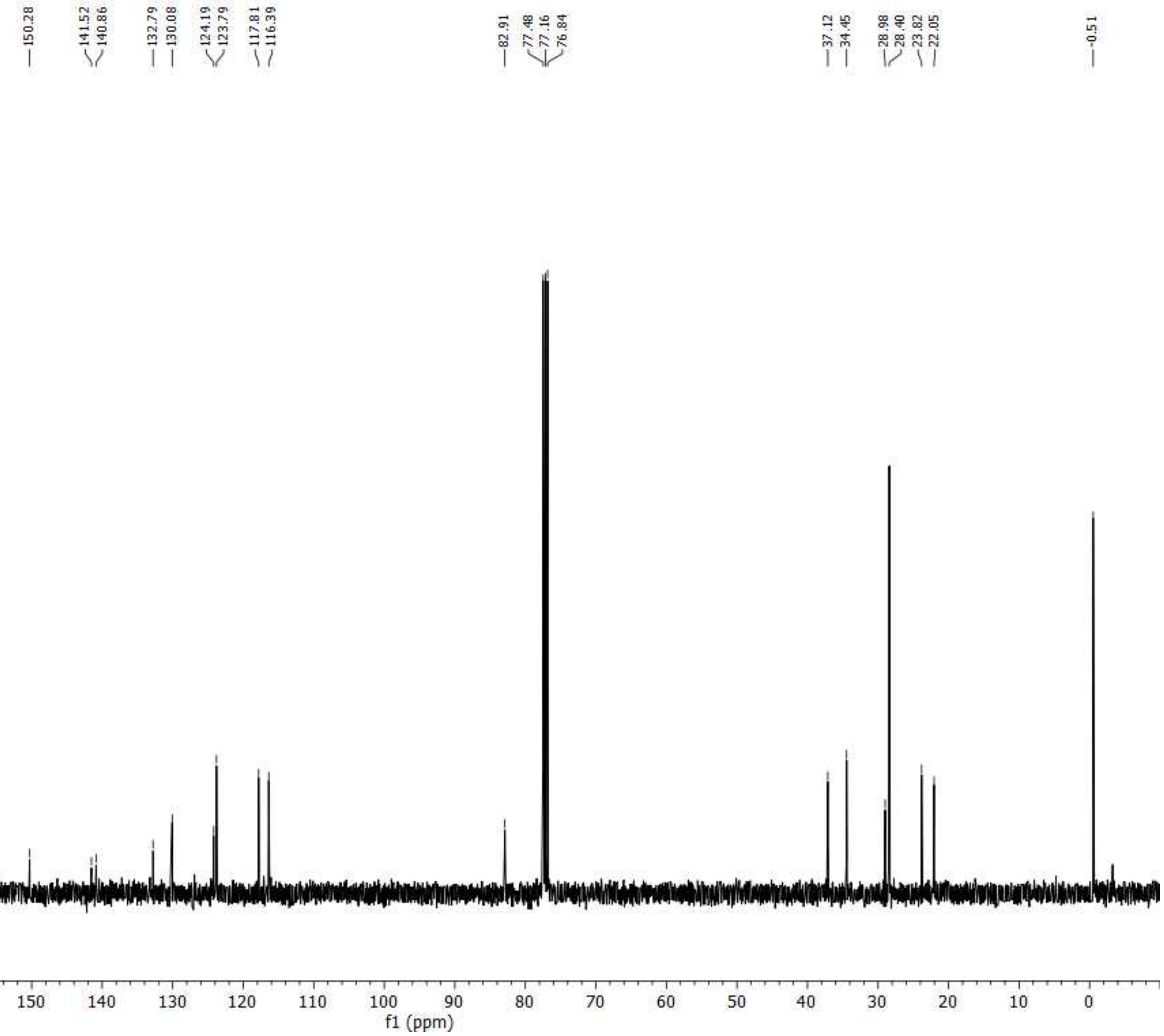

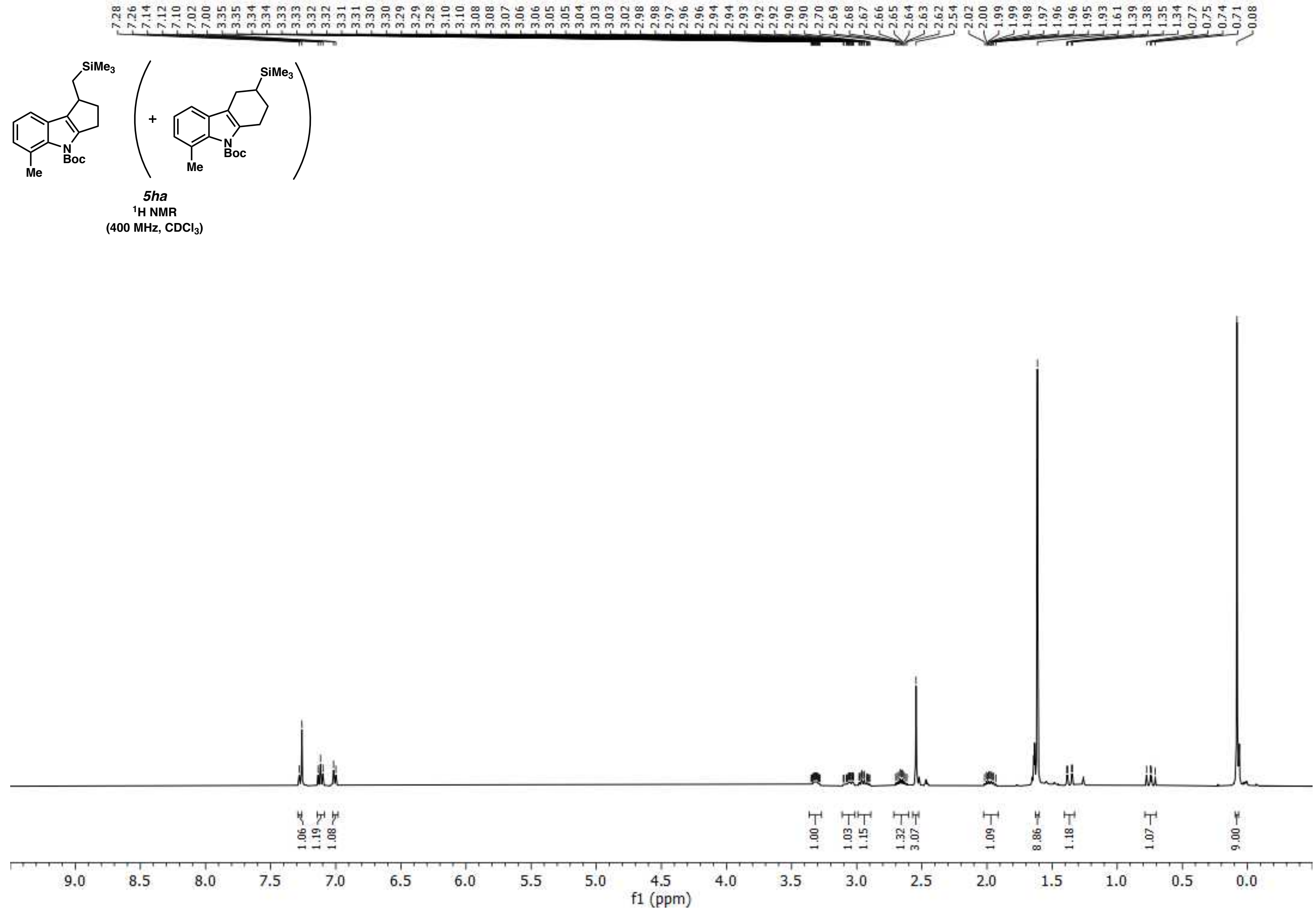


***5ha***
**$^1$H NMR**
**(400 MHz, $CDCl_3$)**

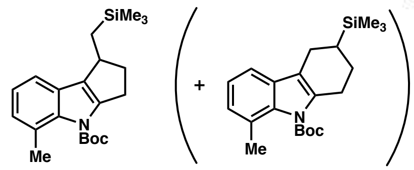


***5ha***
$^{13}$C NMR
(100 MHz, $CDCl_3$)

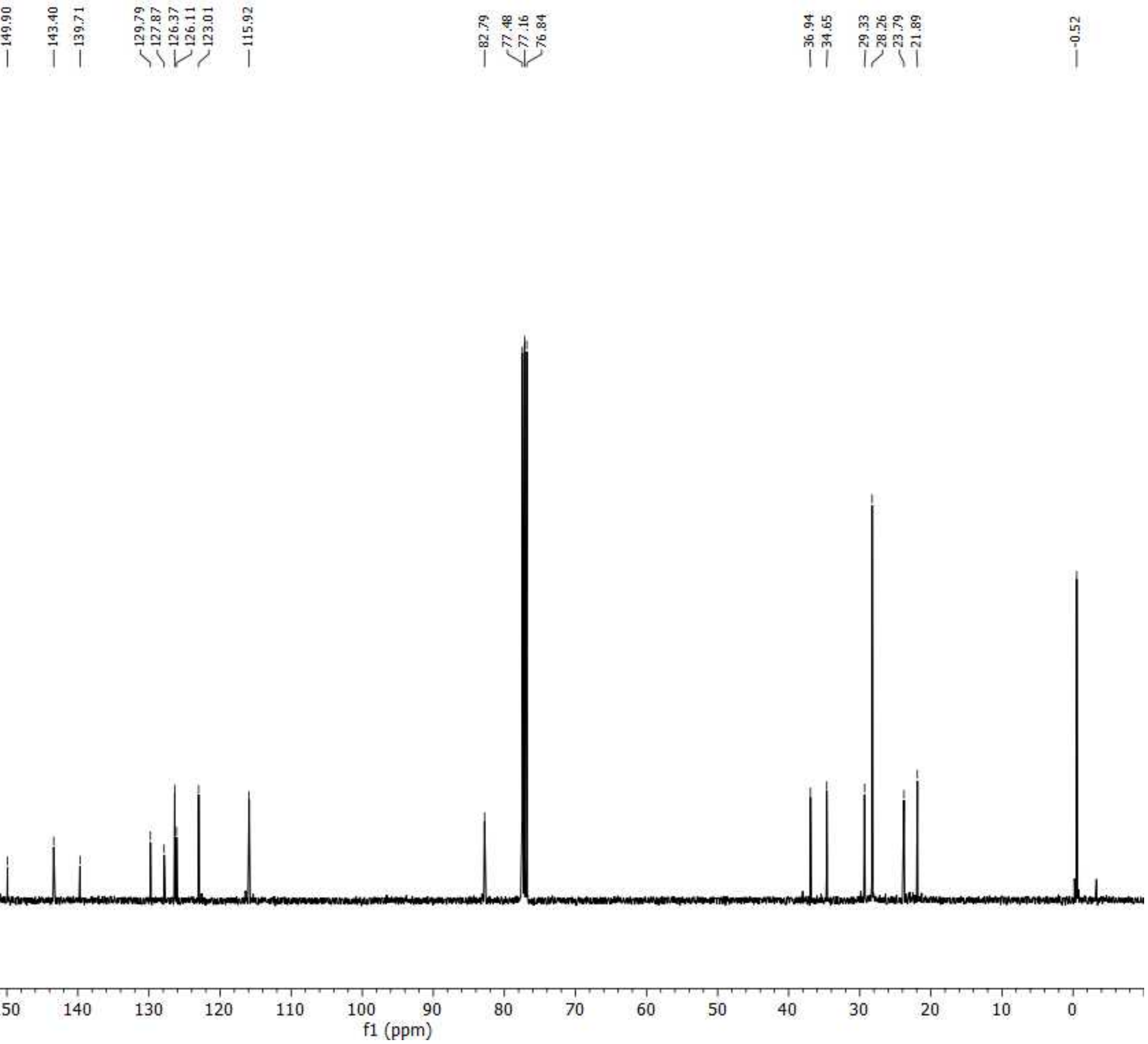

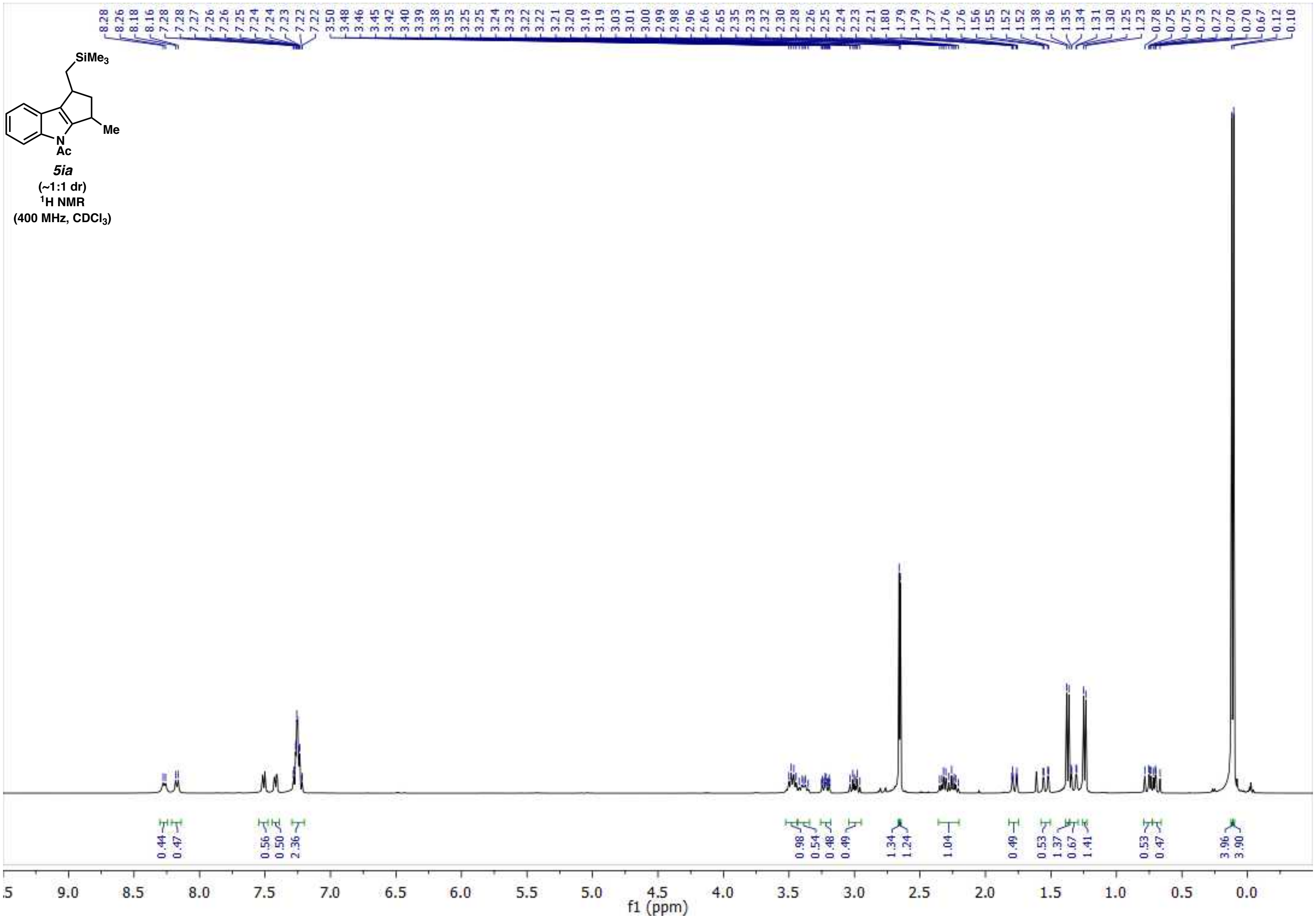
SiMe3
Me
N
Ac
5ia
(~1:1 dr)
1H NMR
(400 MHz, CDCl3)
8.28 8.26 8.18 8.16 7.28 7.28 7.27 7.26 7.26 7.25 7.24 7.24 7.23 7.22 7.22 3.50 3.48 3.46 3.45 3.42 3.40 3.39 3.38 3.35 3.25 3.25 3.24 3.23 3.22 3.22 3.21 3.20 3.19 3.19 3.03 3.01 3.00 2.99 2.98 2.96 2.66 2.65 2.35 2.33 2.32 2.30 2.28 2.26 2.25 2.24 2.23 2.21 1.80 1.79 1.79 1.77 1.76 1.76 1.56 1.55 1.52 1.52 1.38 1.36 1.35 1.34 1.31 1.30 1.25 1.23 0.78 0.75 0.75 0.73 0.72 0.70 0.70 0.67 0.12 0.10
0.44 0.47 0.56 0.50 2.36 0.98 0.54 0.48 0.49 1.34 1.24 1.04 0.49 0.53 1.37 0.67 1.41 0.53 0.47 3.96 3.90
.5 9.0 8.5 8.0 7.5 7.0 6.5 6.0 5.5 5.0 4.5 4.0 3.5 3.0 2.5 2.0 1.5 1.0 0.5 0.0
f1 (ppm)

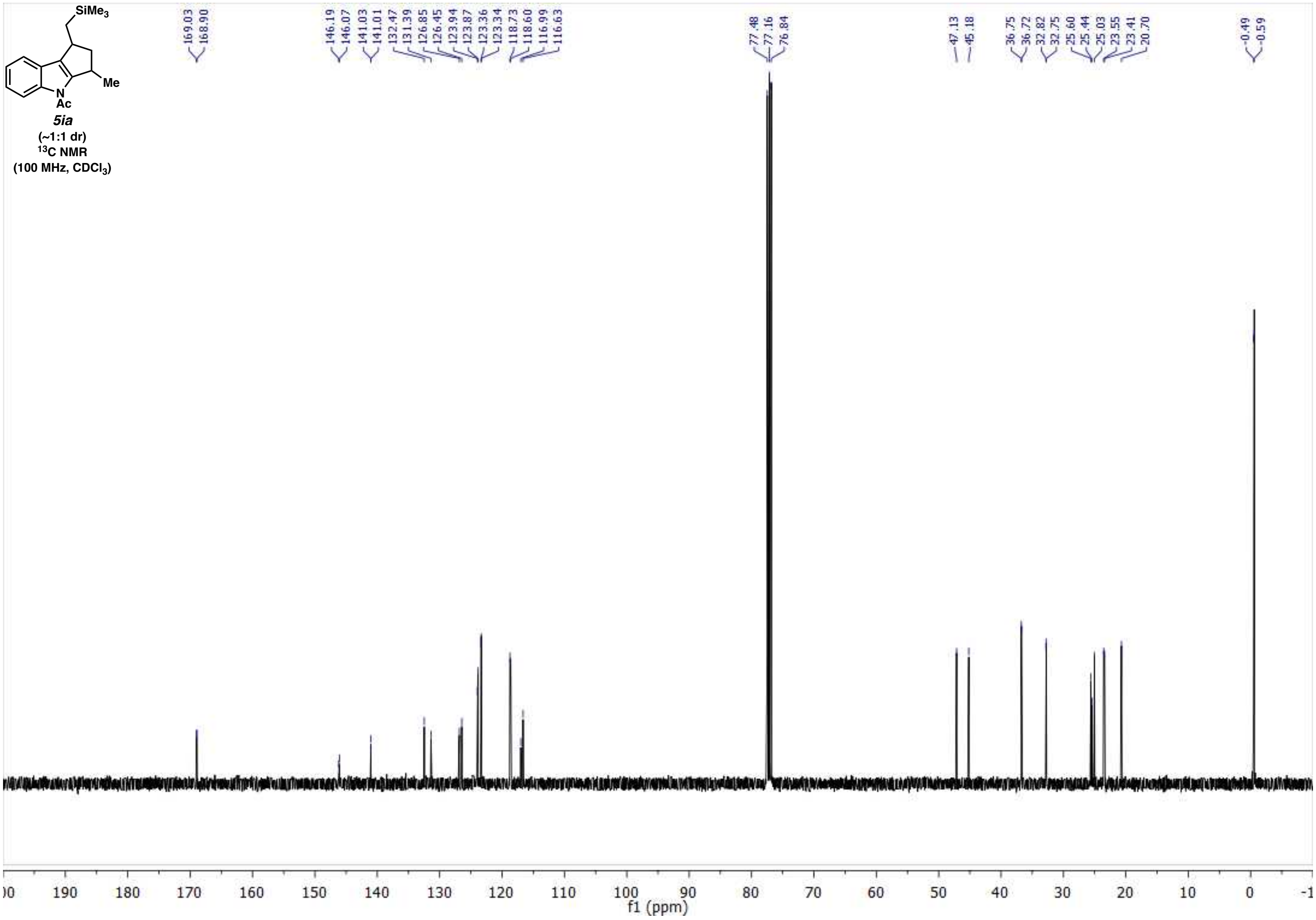
SiMe3
Me
N
Ac
5ia
(~1:1 dr)
13C NMR
(100 MHz, CDCl3)
169.03
168.90
146.19
146.07
141.03
141.01
132.47
131.39
126.85
126.45
123.94
123.87
123.36
123.34
118.73
118.60
116.99
116.63
77.48
77.16
76.84
47.13
45.18
36.75
36.72
32.82
32.75
25.60
25.44
25.03
23.55
23.41
20.70
-0.49
-0.59
f1 (ppm)

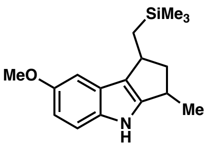

**5ja´**
**(~1:1 dr)**
**$^1$H NMR**
**(400 MHz, $CDCl_3$)**

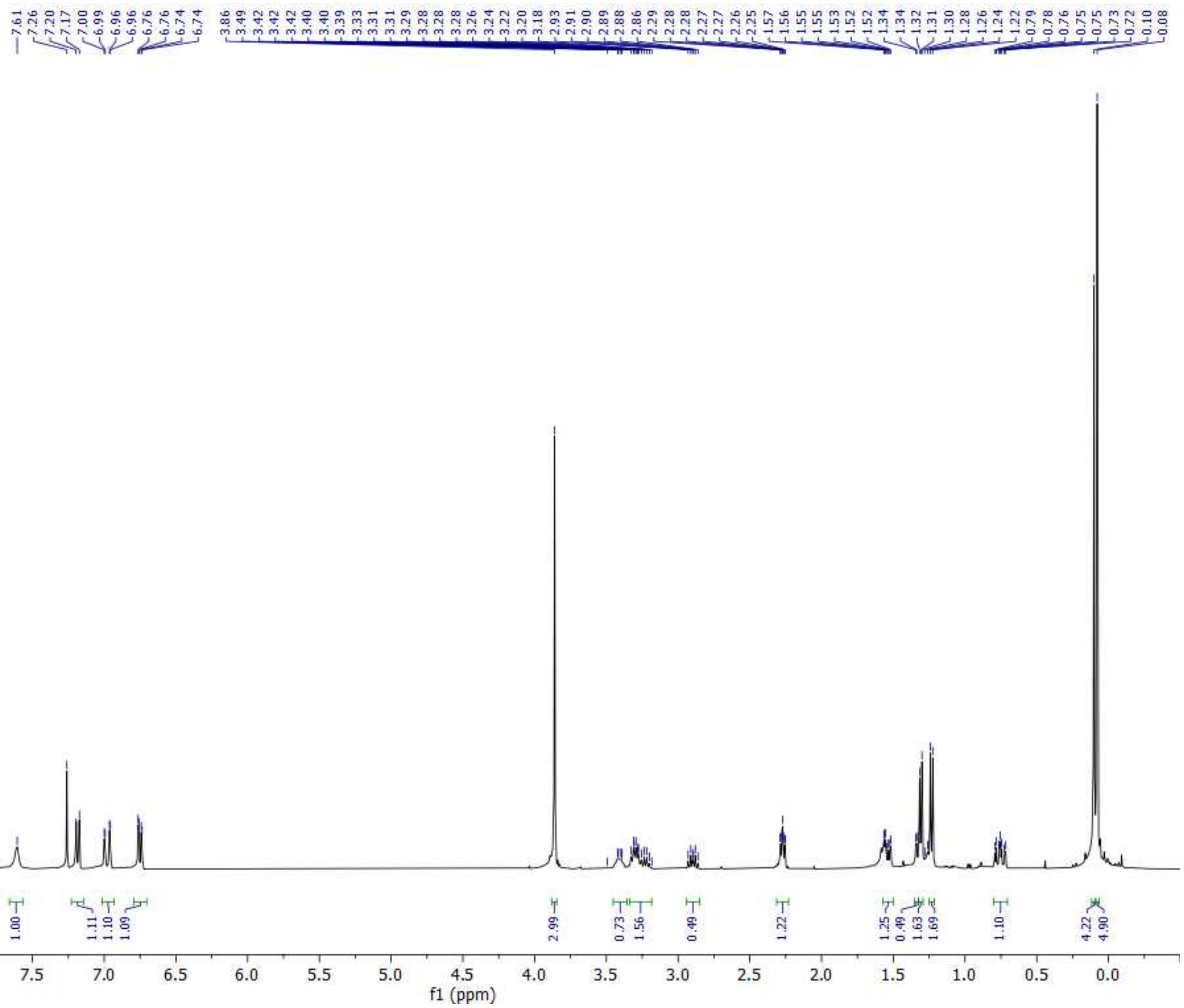

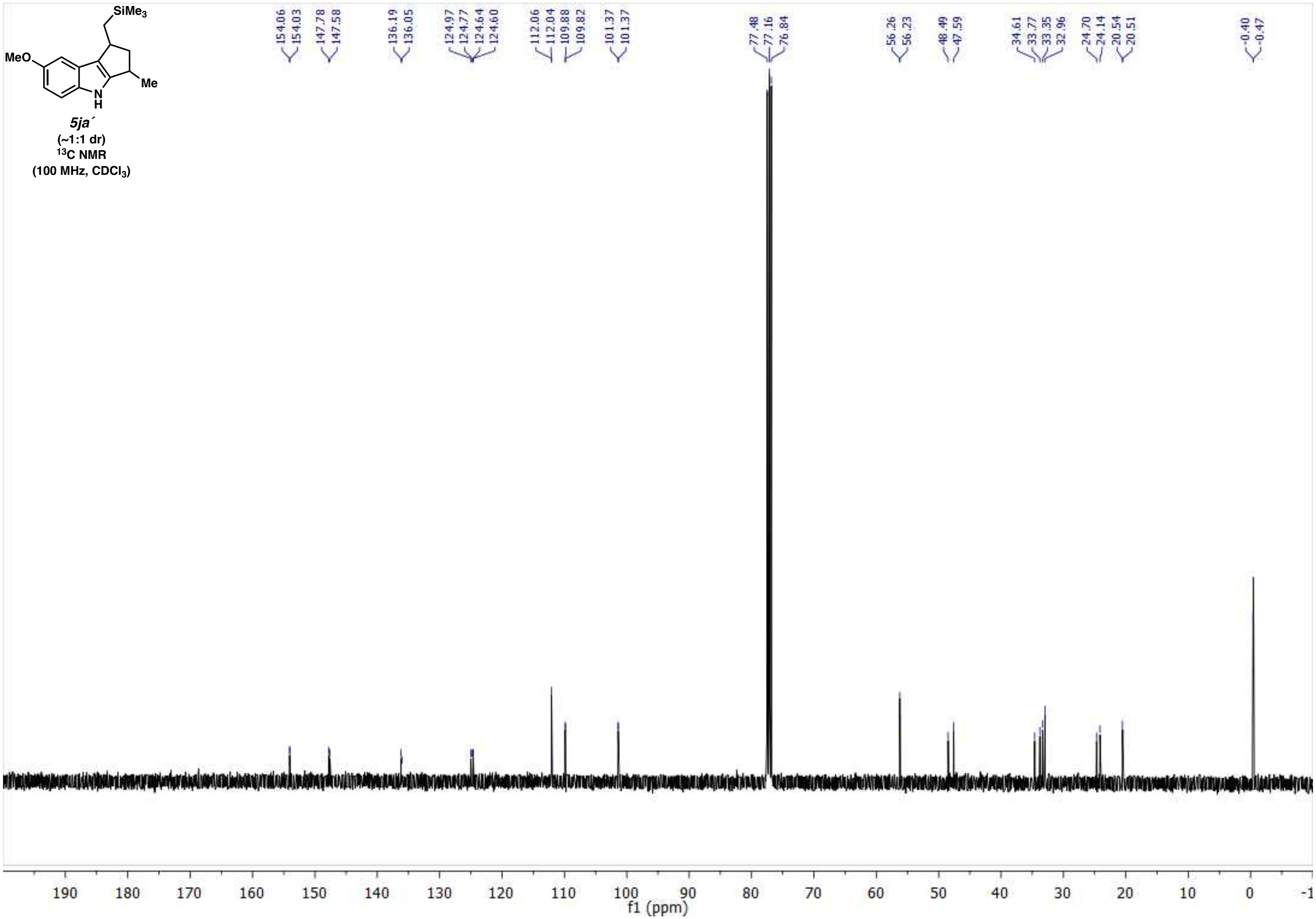
SiMe3
MeO
Me
N
H
5ja´
(~1:1 dr)
13C NMR
(100 MHz, CDCl3)
154.06
154.03
147.78
147.58
136.19
136.05
124.97
124.77
124.64
124.60
112.06
112.04
109.88
109.82
101.37
101.37
77.48
77.16
76.84
56.26
56.23
48.49
47.59
34.61
33.77
33.35
32.96
24.70
24.14
20.54
20.51
-0.40
-0.47
190
180
170
160
150
140
130
120
110
100
90
80
70
60
50
40
30
20
10
0
-1
f1 (ppm)

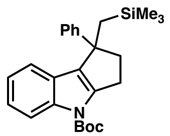


***5ab***
**$^1$H NMR**
**(400 MHz, $CDCl_3$)**

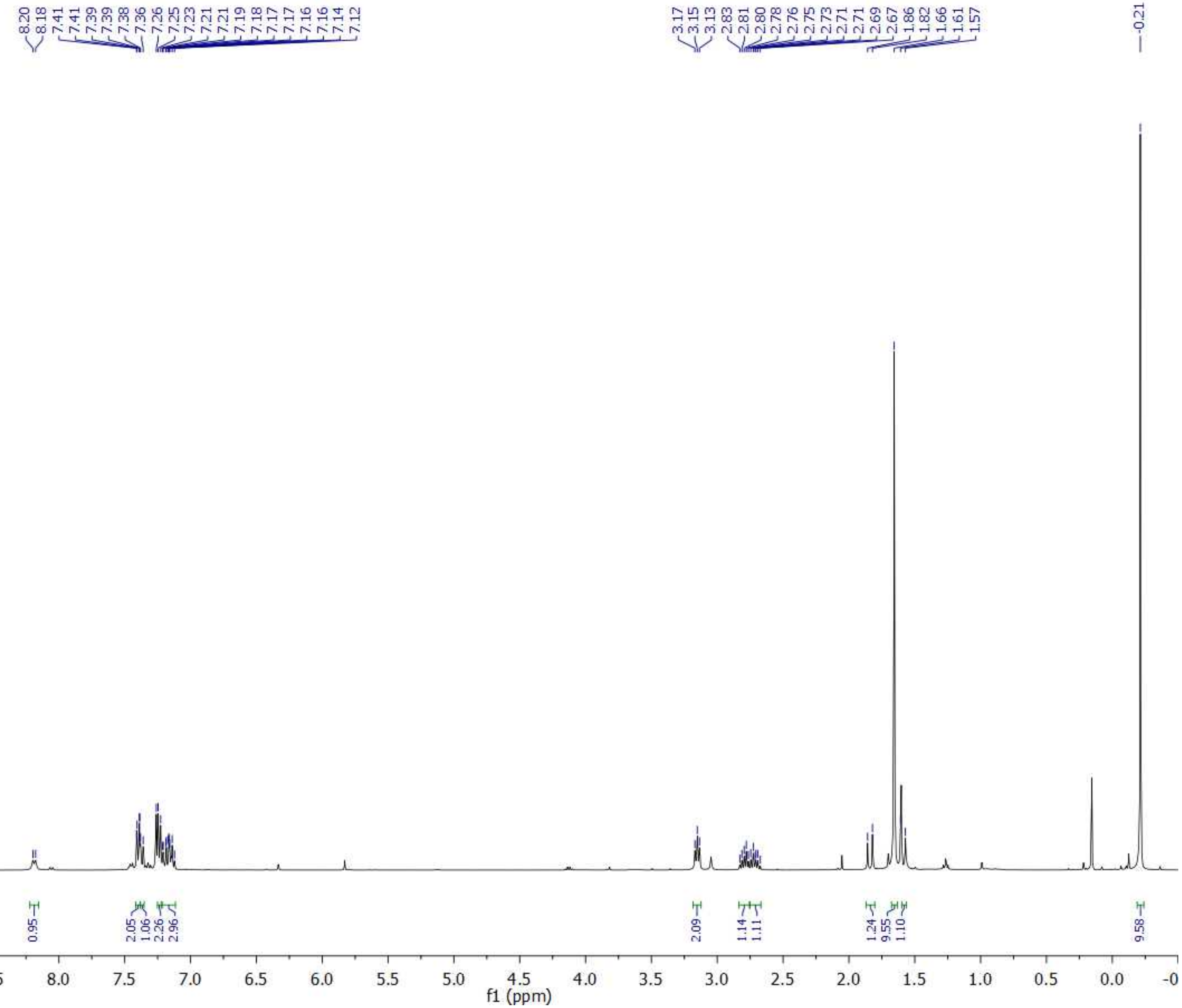

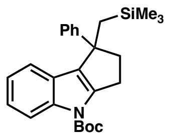

***5ab***
$^{13}C$ NMR
(100 MHz, $CDCl_3$)

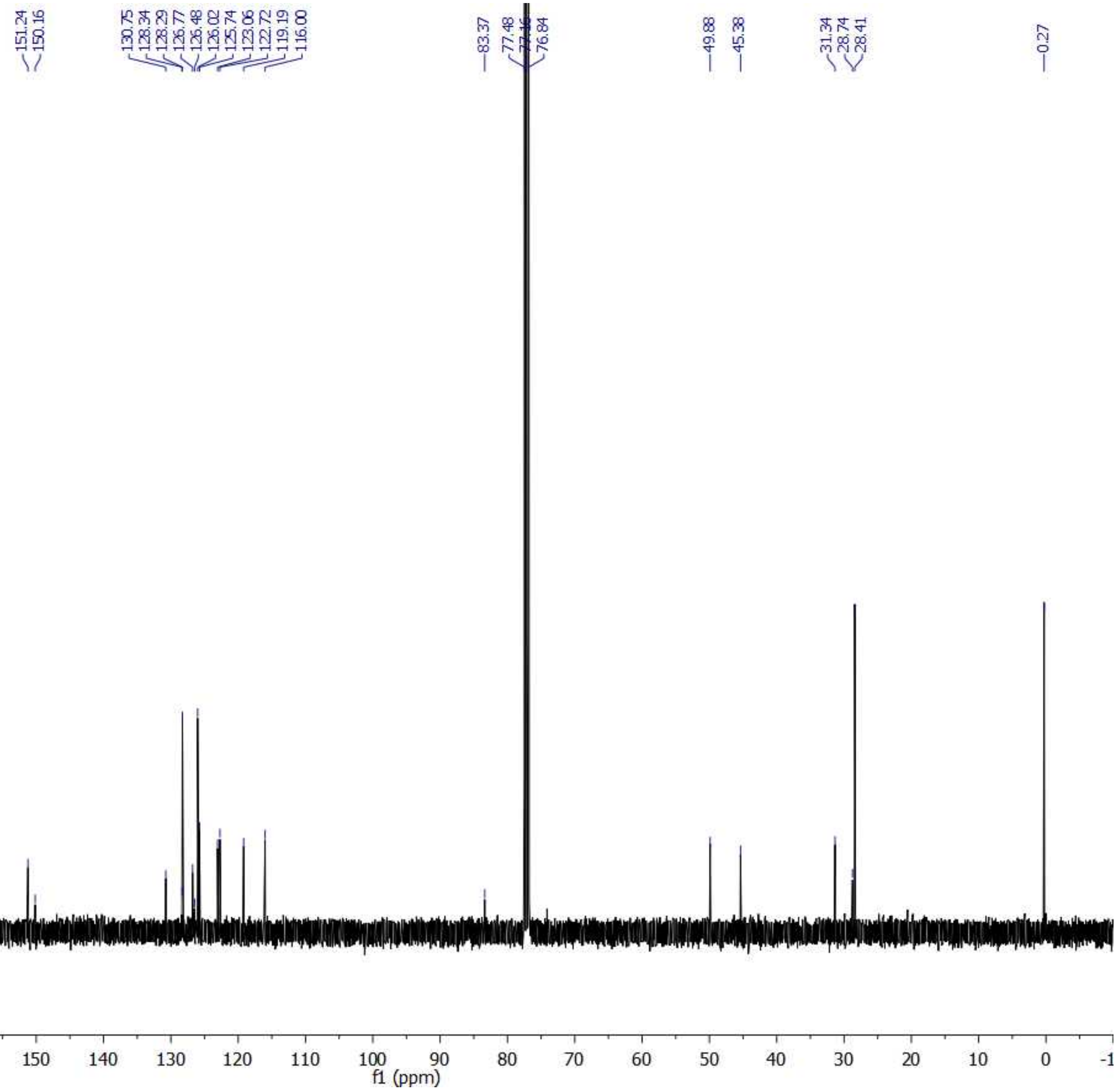

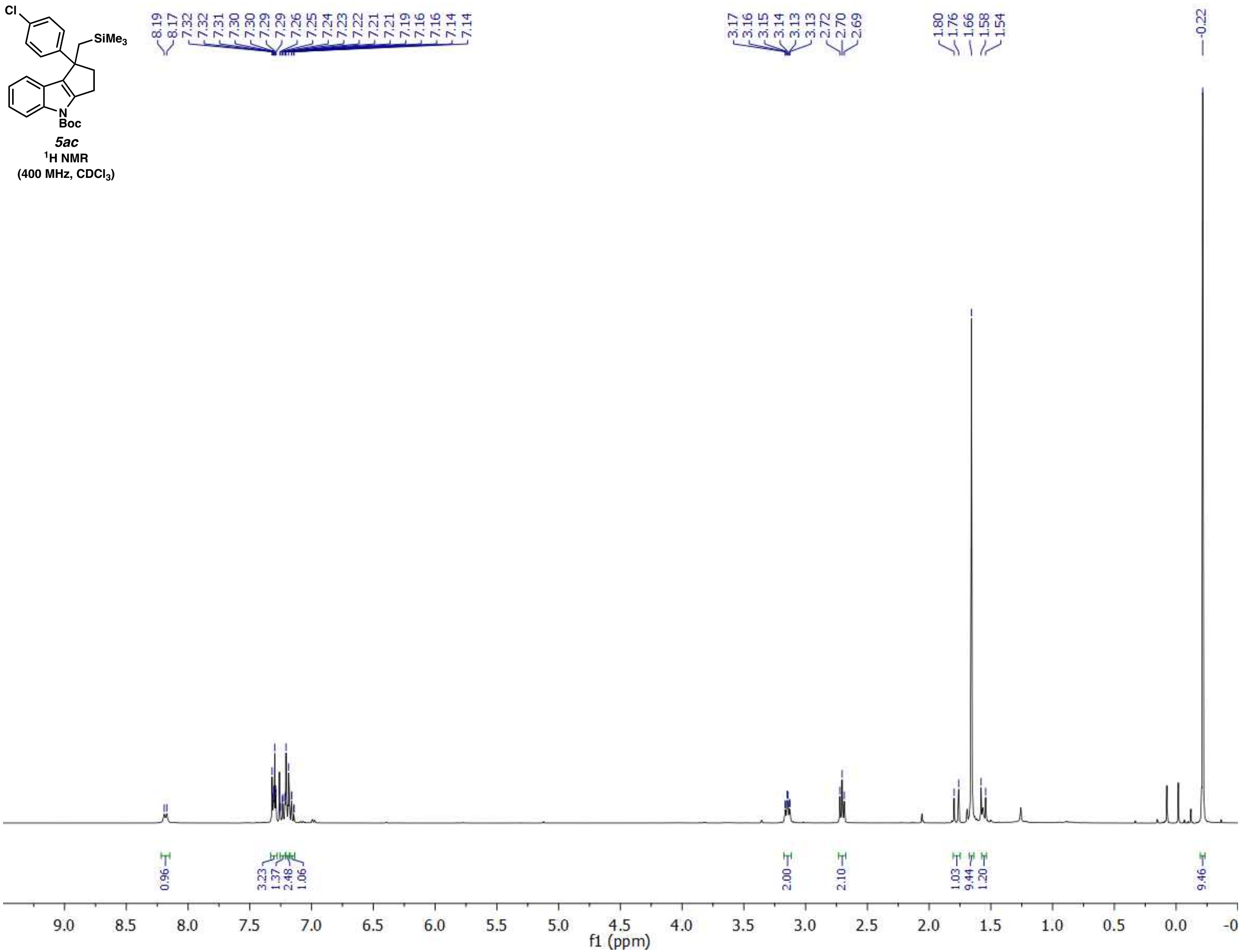
Cl
SiMe3
N
Boc
5ac
1H NMR
(400 MHz, CDCl3)
8.19
8.17
7.32
7.32
7.31
7.30
7.30
7.29
7.29
7.26
7.25
7.24
7.23
7.22
7.21
7.21
7.19
7.16
7.16
7.14
7.14
3.17
3.16
3.15
3.14
3.13
3.13
2.72
2.70
2.69
1.80
1.76
1.66
1.58
1.54
-0.22
0.96
3.23
1.37
2.48
1.06
2.00
2.10
1.03
9.44
1.20
9.46
9.0
8.5
8.0
7.5
7.0
6.5
6.0
5.5
5.0
4.5
4.0
3.5
3.0
2.5
2.0
1.5
1.0
0.5
0.0
-0
f1 (ppm)

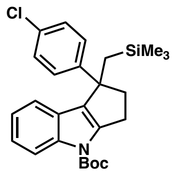

***5ac***
$^{13}C$ NMR
(100 MHz, $CDCl_3$)

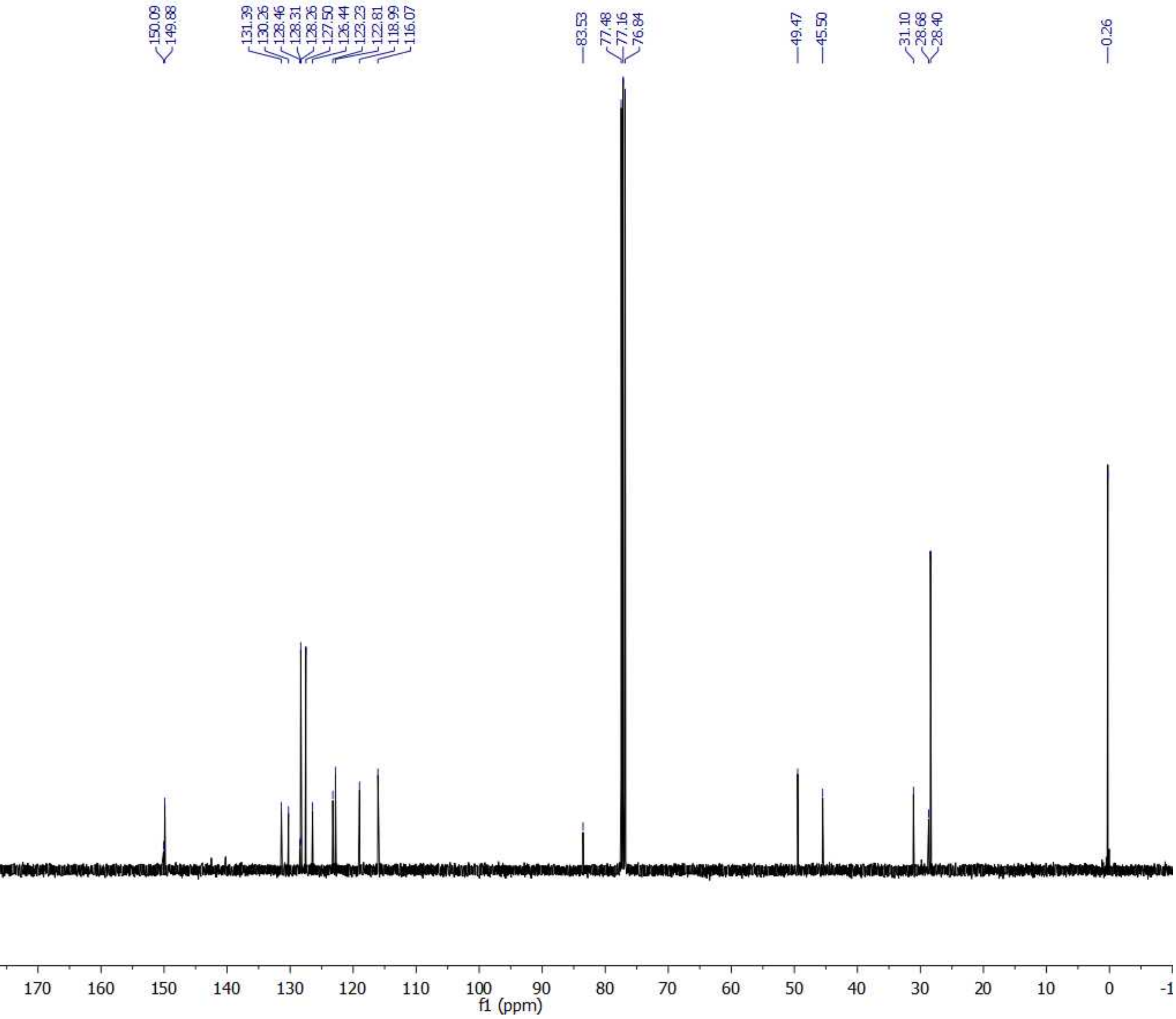

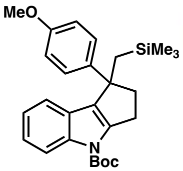


***5ad***
$^1$H NMR
(400 MHz, $CDCl_3$)

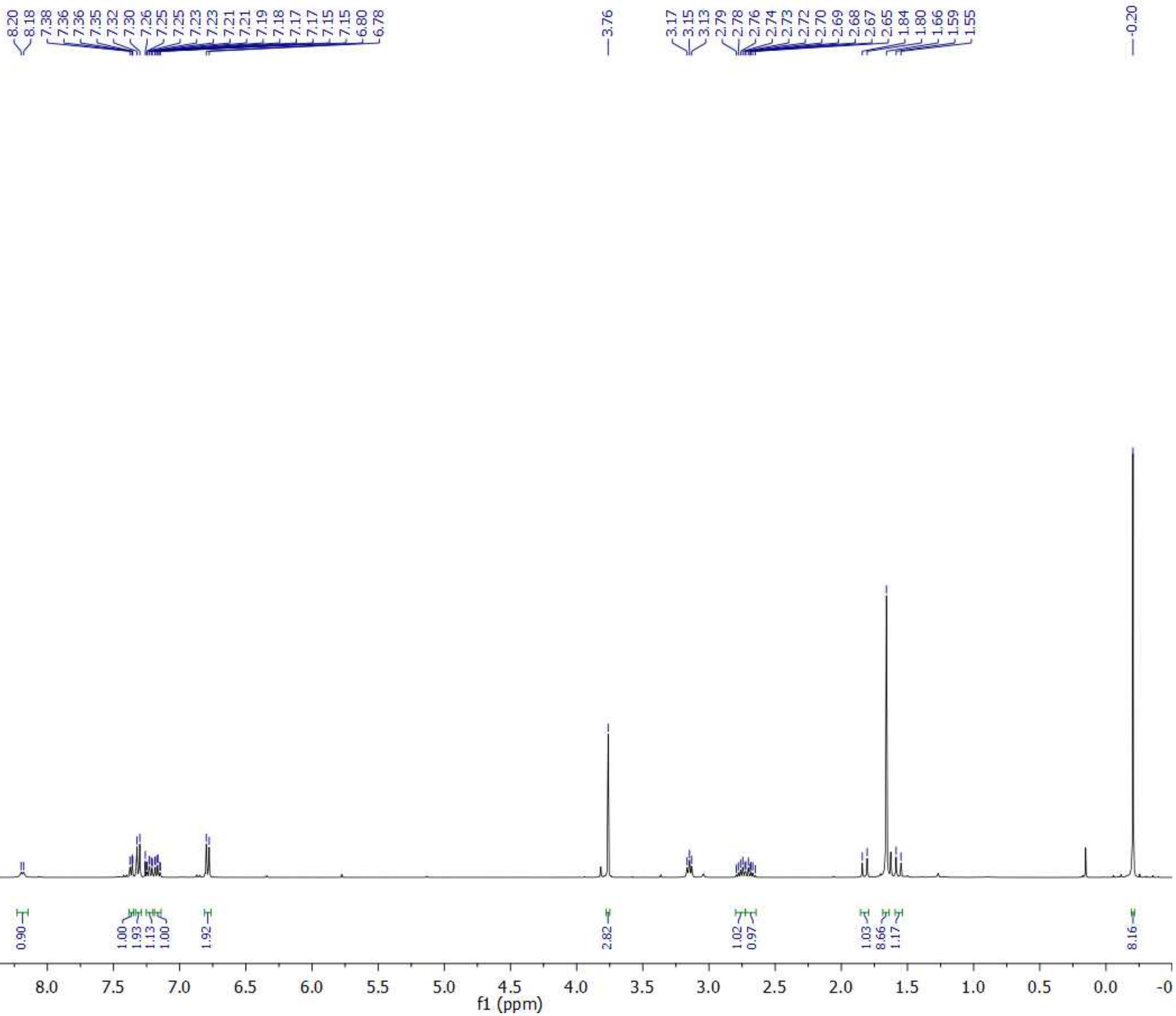

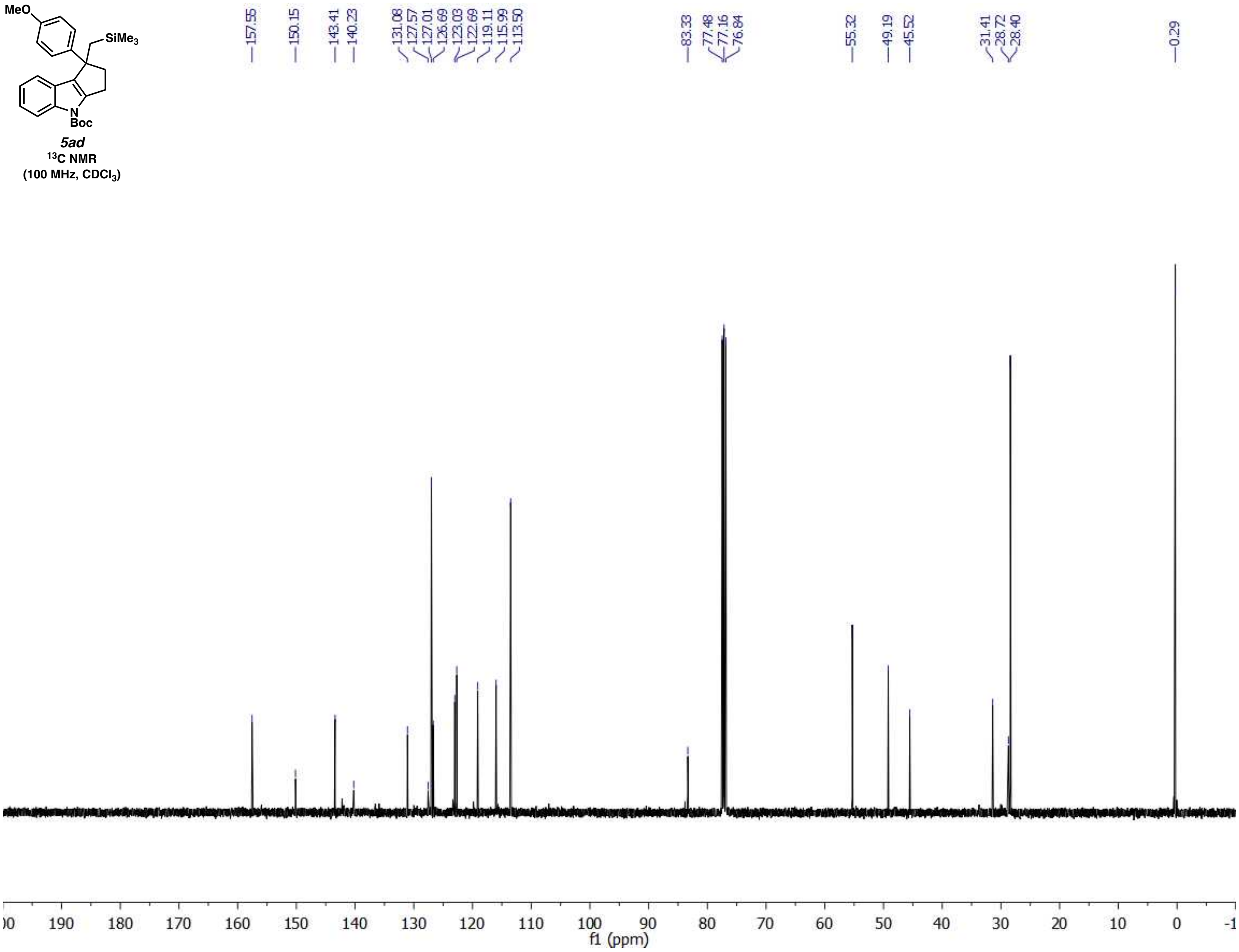


***5ad***
$^{13}$C NMR
(100 MHz, $CDCl_3$)

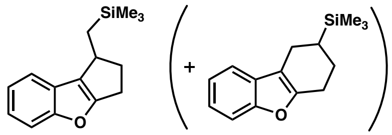


***10aa***
$^1$H NMR
(400 MHz, $CDCl_3$)

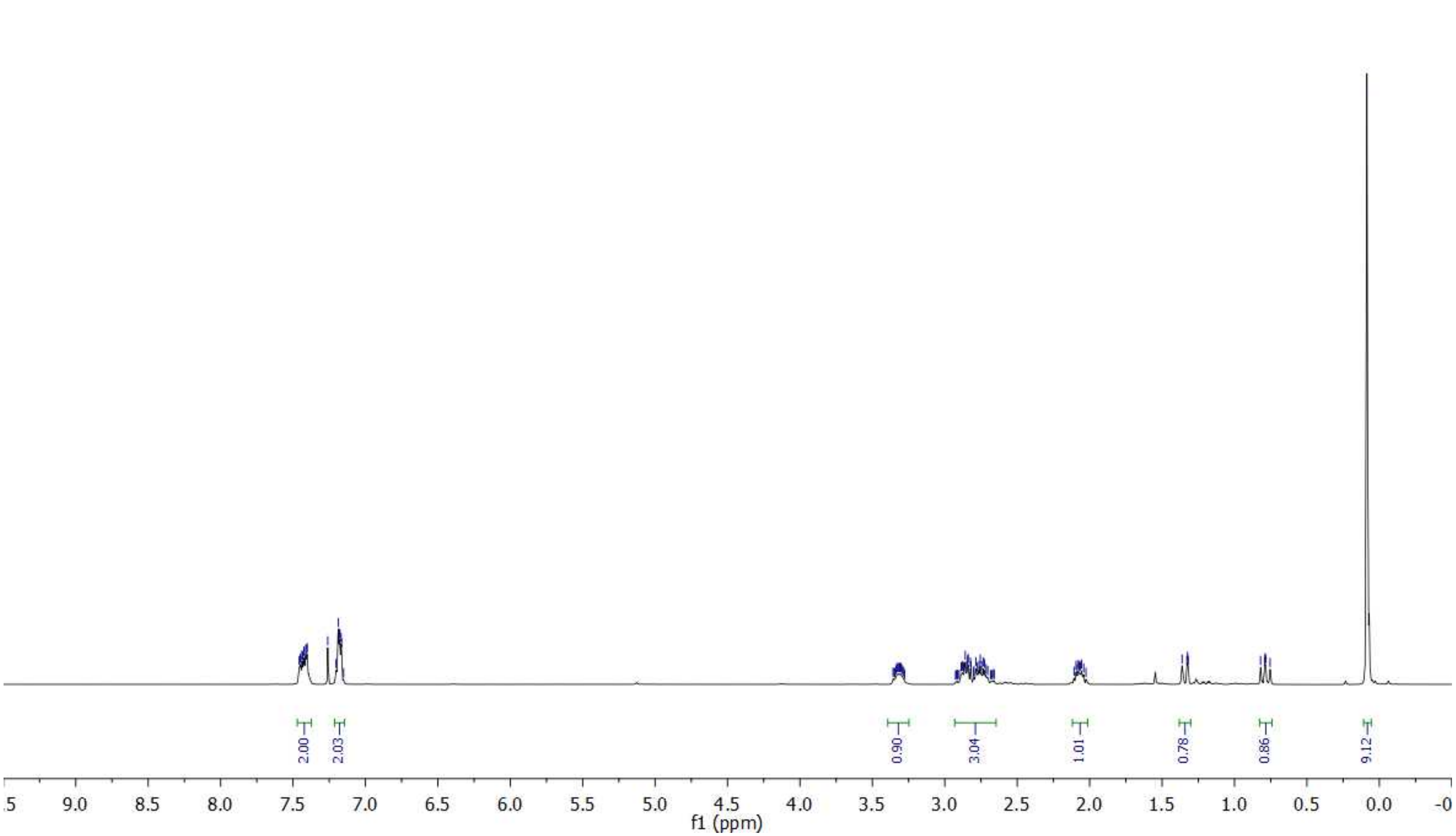

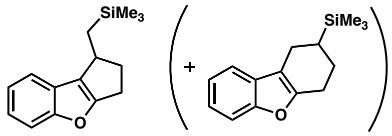

**10aa**
$^{13}C$ NMR
(100 MHz, $CDCl_3$)

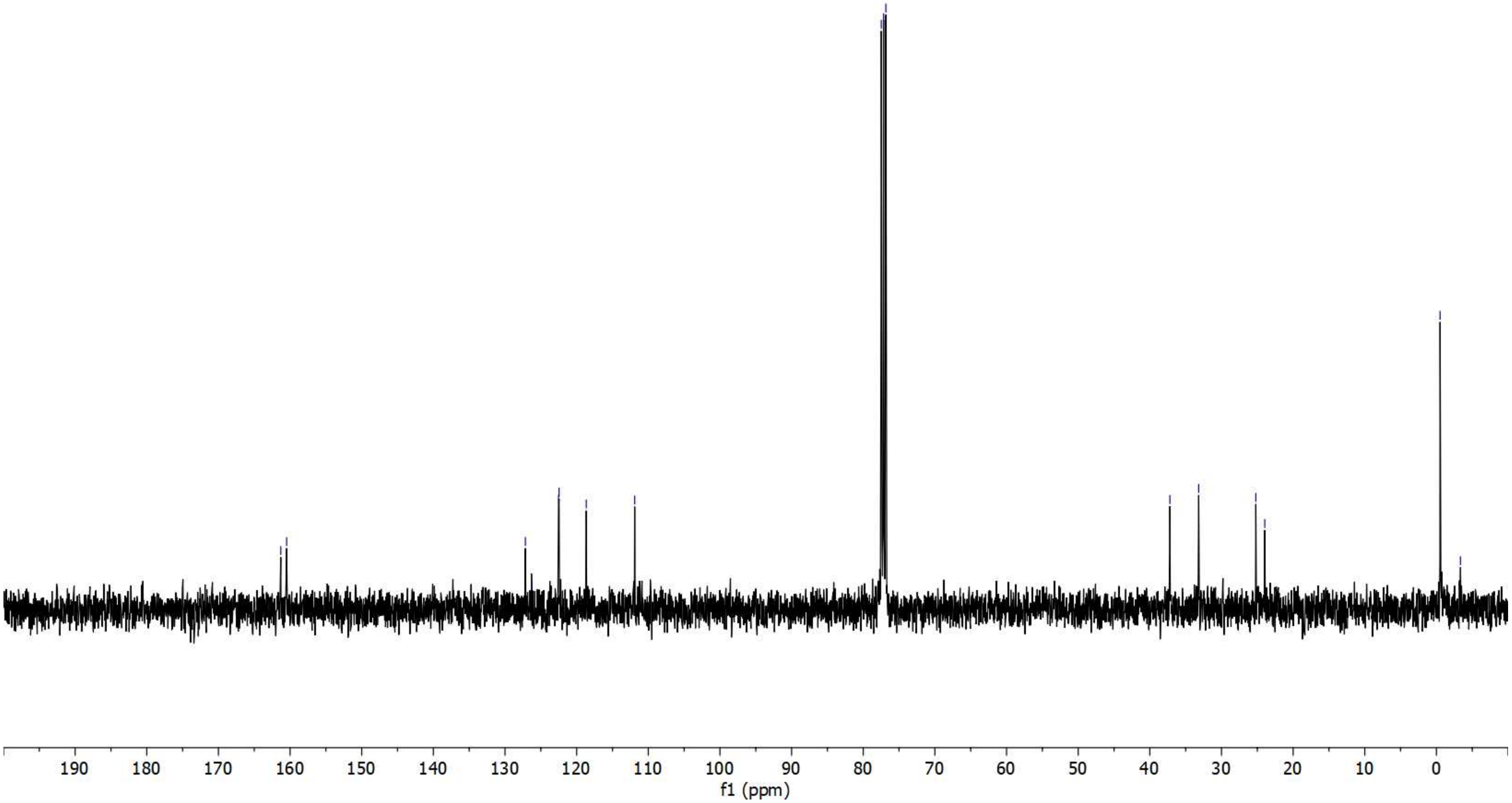

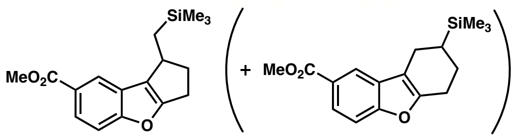


***10ba***
$^1$H NMR
(400 MHz, $CDCl_3$)

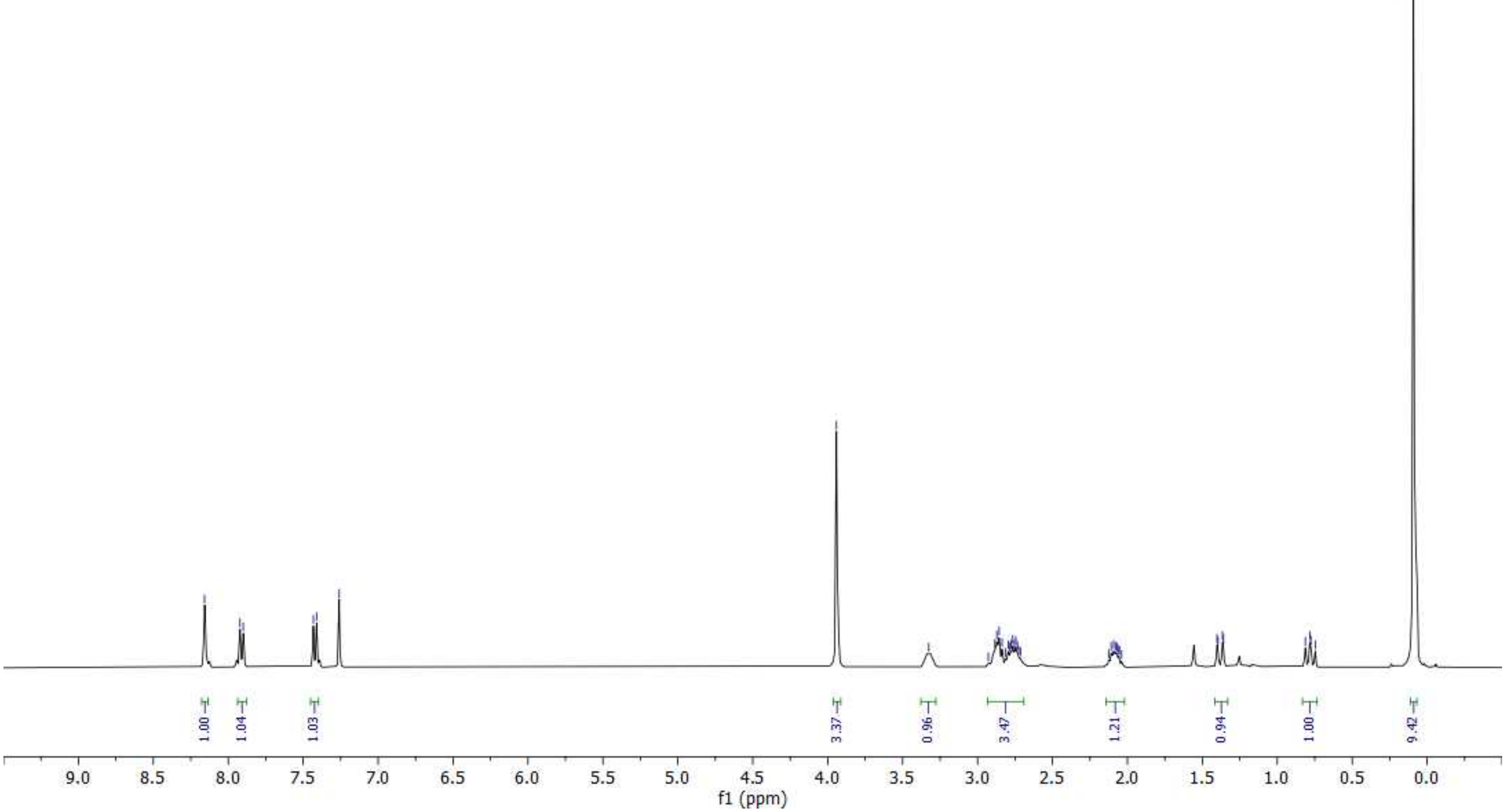

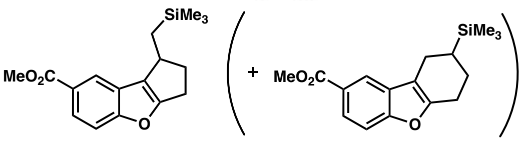


***10ba***
$^{13}C$ NMR
(100 MHz, $CDCl_3$)

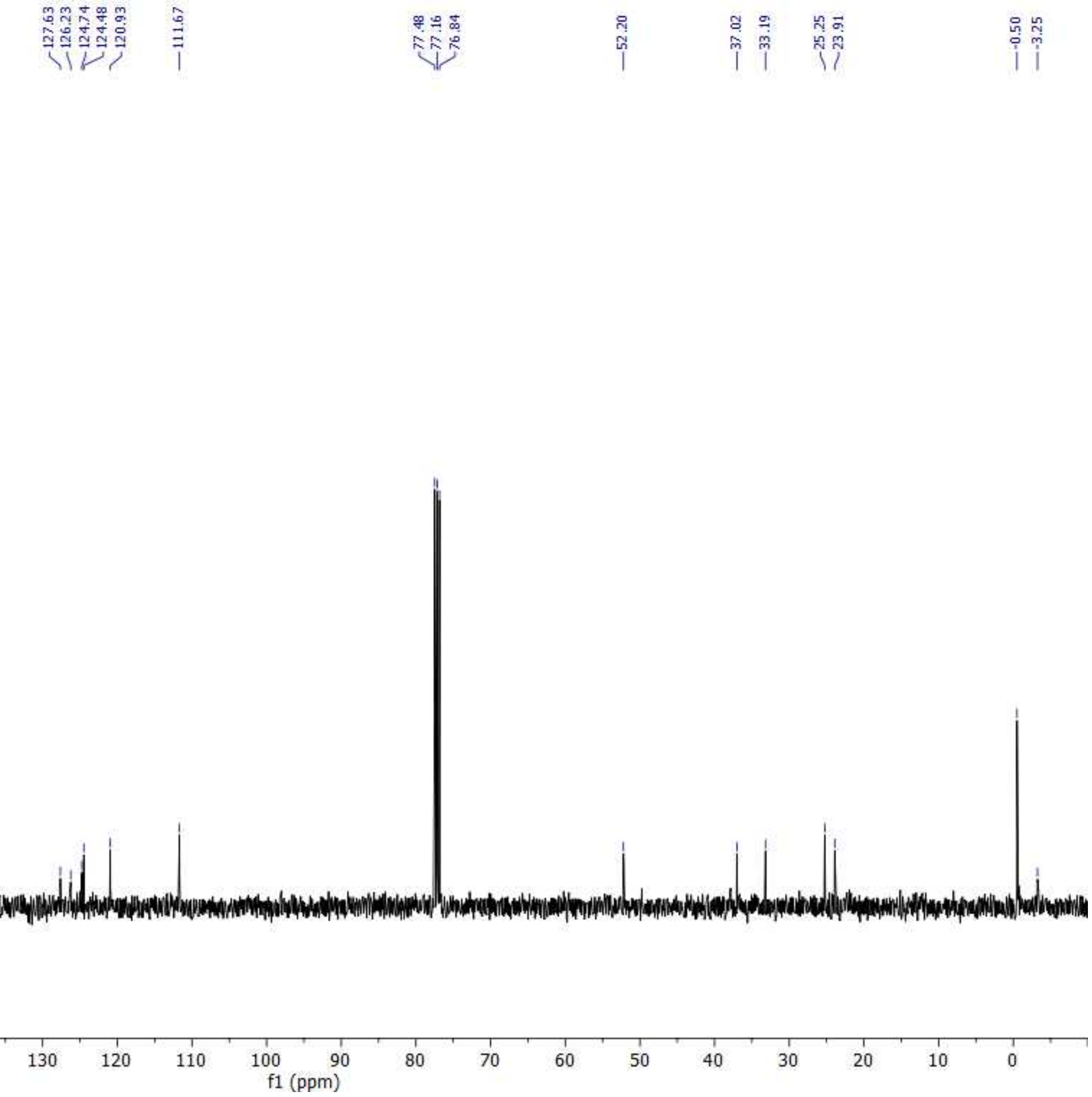

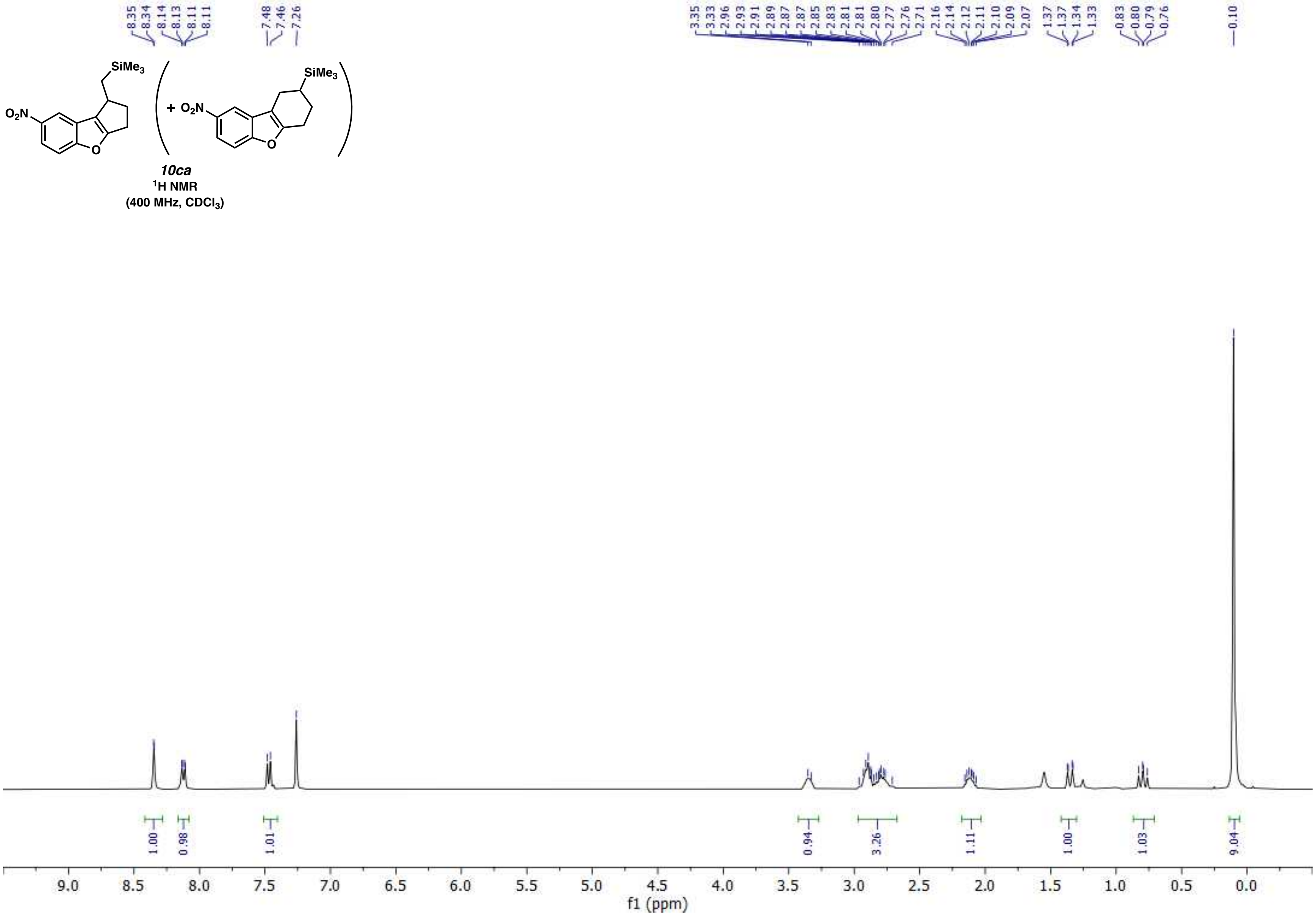


***10ca***
$^1$H NMR
(400 MHz, $CDCl_3$)

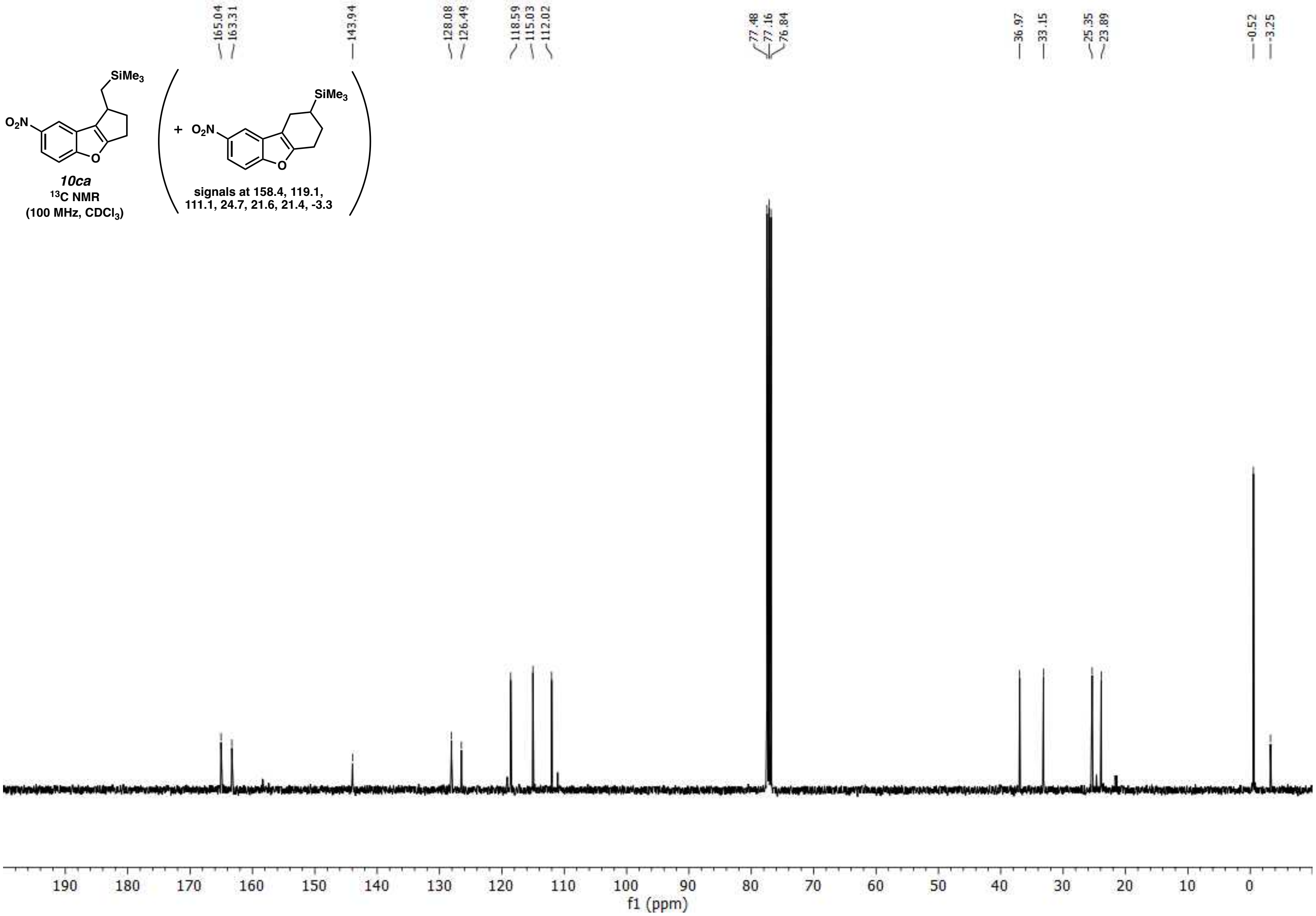

165.04
163.31
143.94
128.08
126.49
118.59
115.03
112.02
77.48
77.16
76.84
36.97
33.15
25.35
23.89
-0.52
-3.25
SiMe3
O2N
O
10ca
13C NMR
(100 MHz, CDCl3)
+ O2N
SiMe3
O
signals at 158.4, 119.1,
111.1, 24.7, 21.6, 21.4, -3.3
190 180 170 160 150 140 130 120 110 100 90 80 70 60 50 40 30 20 10 0
f1 (ppm)

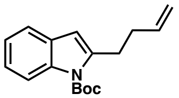

**6aa**
**$^1$H NMR**
**(400 MHz, $CDCl_3$)**

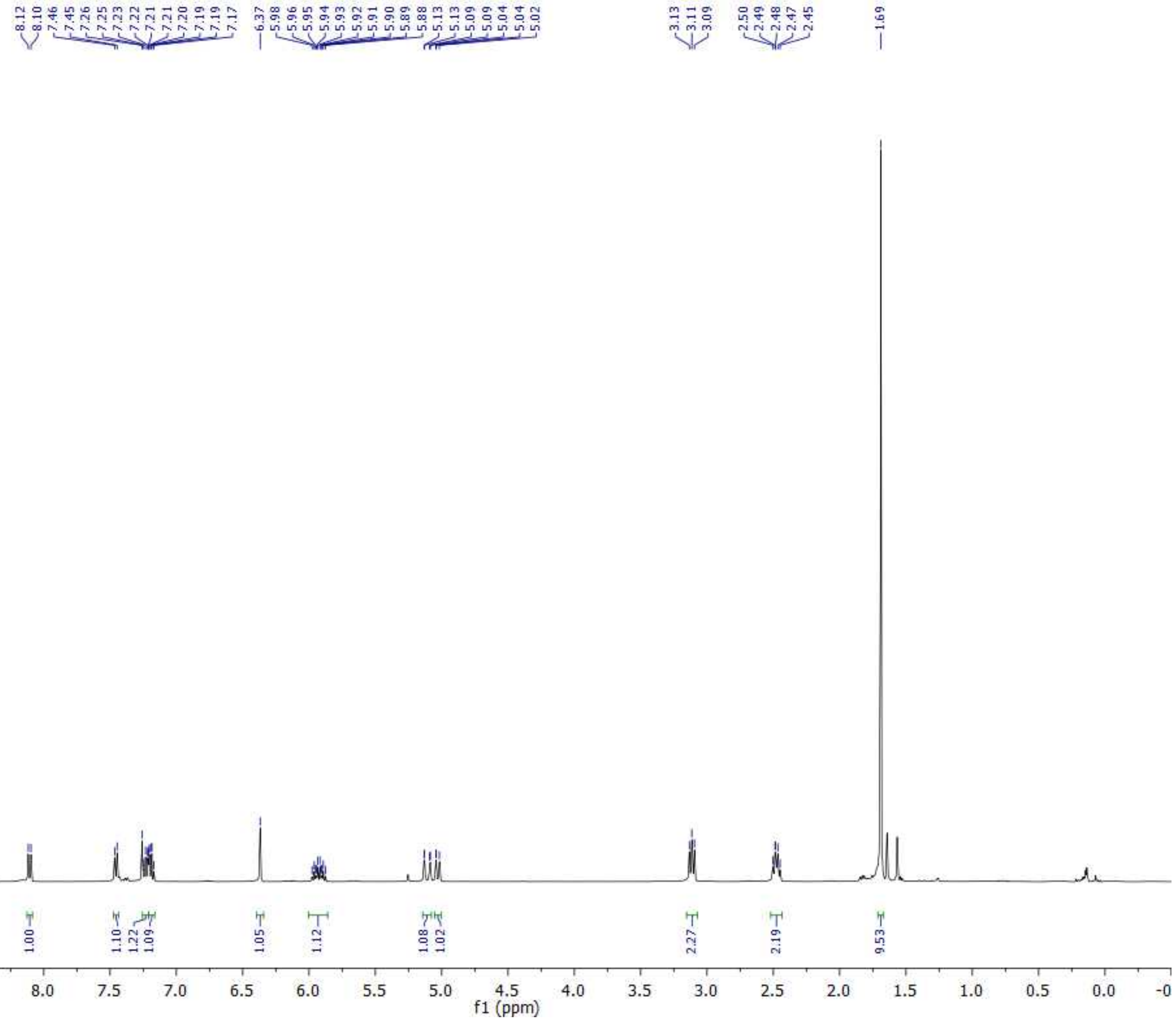

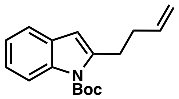


***6aa***
**$^{13}$C NMR**
**(100 MHz, $CDCl_3$)**

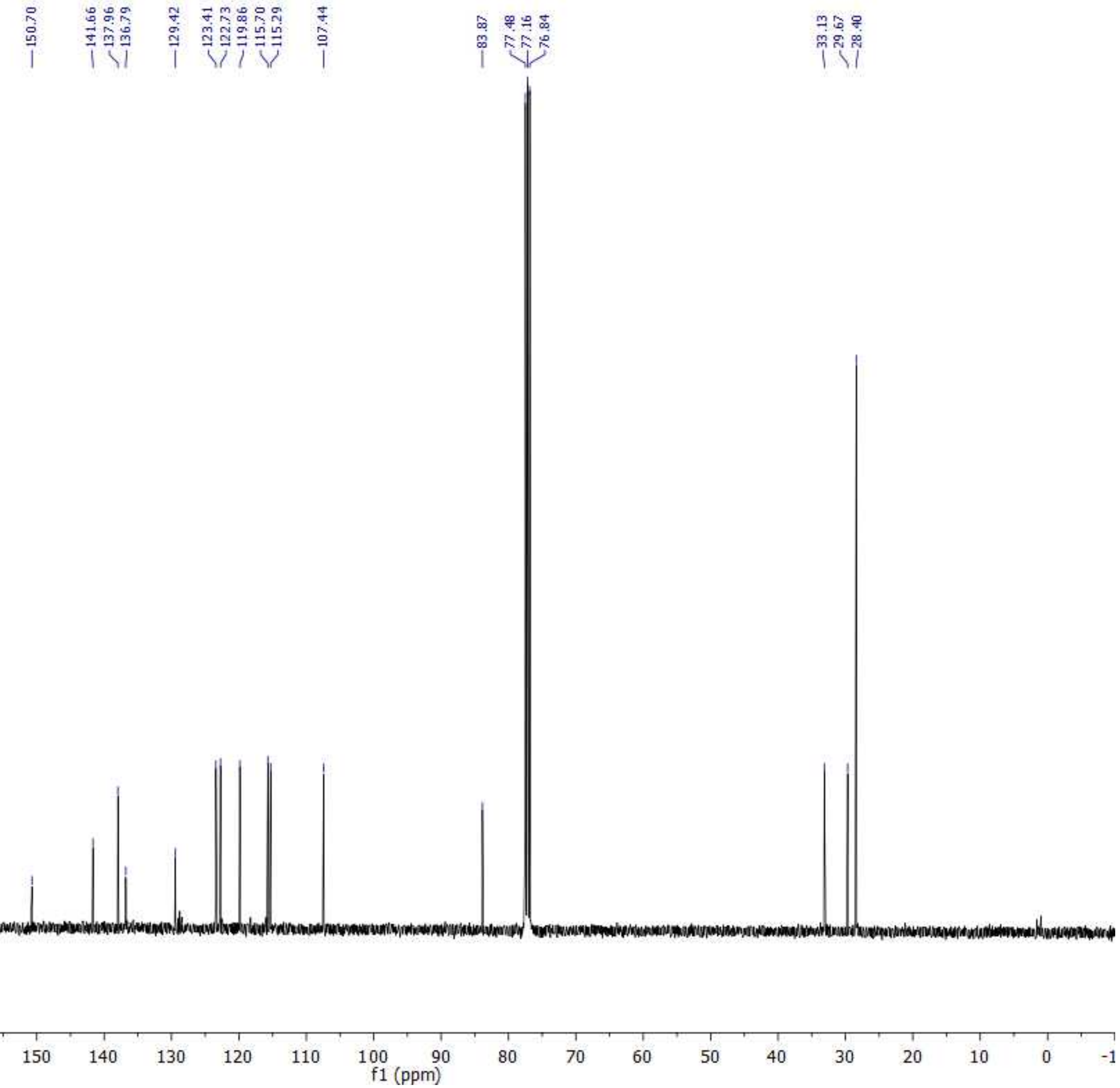

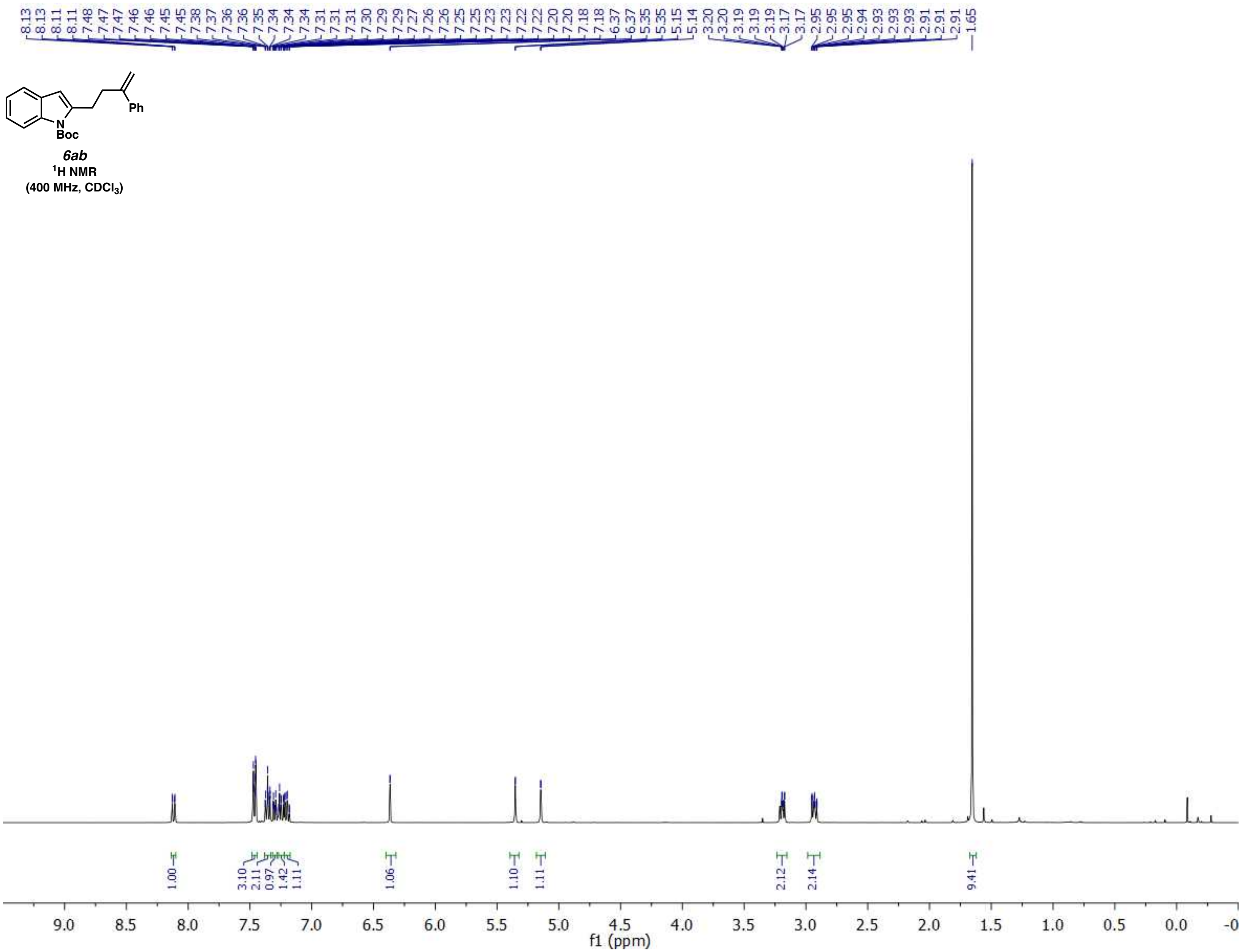
Ph
N
Boc
6ab
1H NMR
(400 MHz, CDCl3)
f1 (ppm)

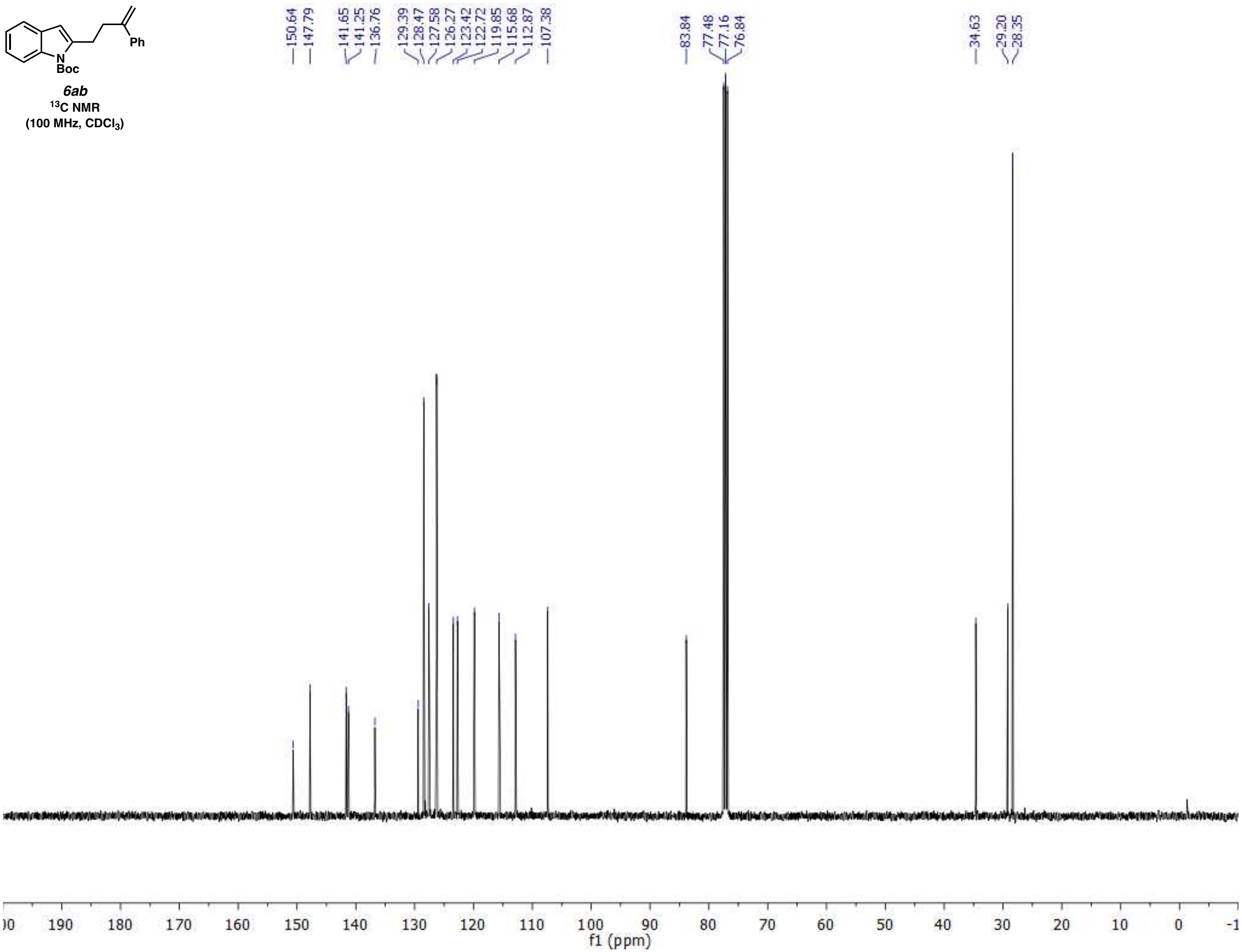
Ph
N
Boc
6ab
$^{13}$C NMR
(100 MHz, CDCl$_3$)
150.64
147.79
141.65
141.25
136.76
129.39
128.47
127.58
126.27
123.42
122.72
119.85
115.68
112.87
107.38
83.84
77.48
77.16
76.84
34.63
29.20
28.35
f1 (ppm)

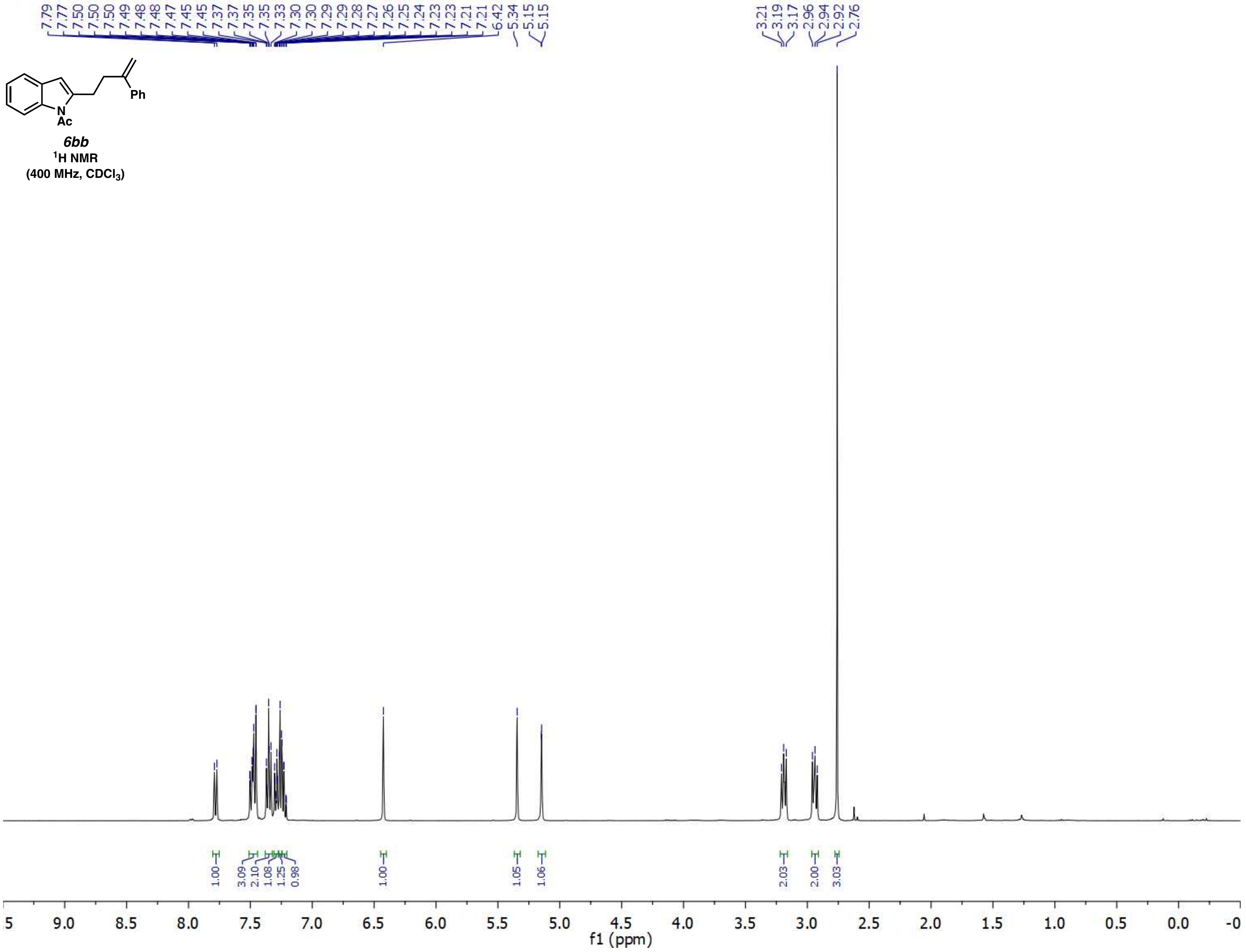

Ph
N
Ac
***6bb***
¹H NMR
(400 MHz, CDCl₃)
7.79
7.77
7.50
7.50
7.49
7.48
7.48
7.47
7.45
7.45
7.37
7.37
7.35
7.35
7.33
7.30
7.30
7.29
7.29
7.28
7.27
7.26
7.25
7.24
7.23
7.23
7.21
7.21
6.42
5.34
5.15
5.15
3.21
3.19
3.17
2.96
2.94
2.92
2.76
1.00
3.09
2.10
1.08
1.25
0.98
1.00
1.05
1.06
2.03
2.00
3.03
f1 (ppm)

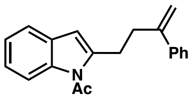

***6bb***
$^{13}$C NMR
(100 MHz, $CDCl_3$)

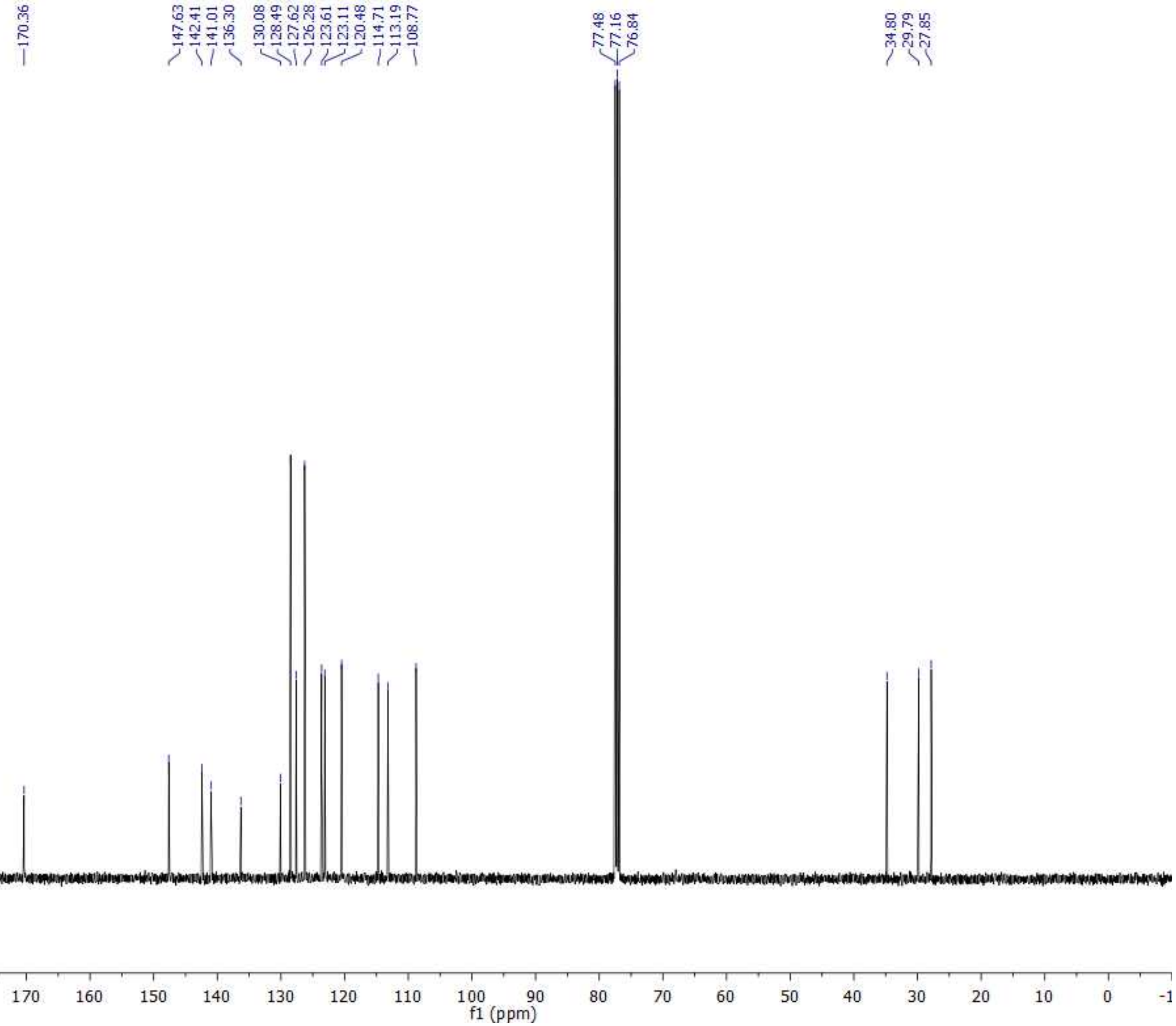

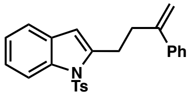

***6cb***
$^1$H NMR
(400 MHz, $CDCl_3$)

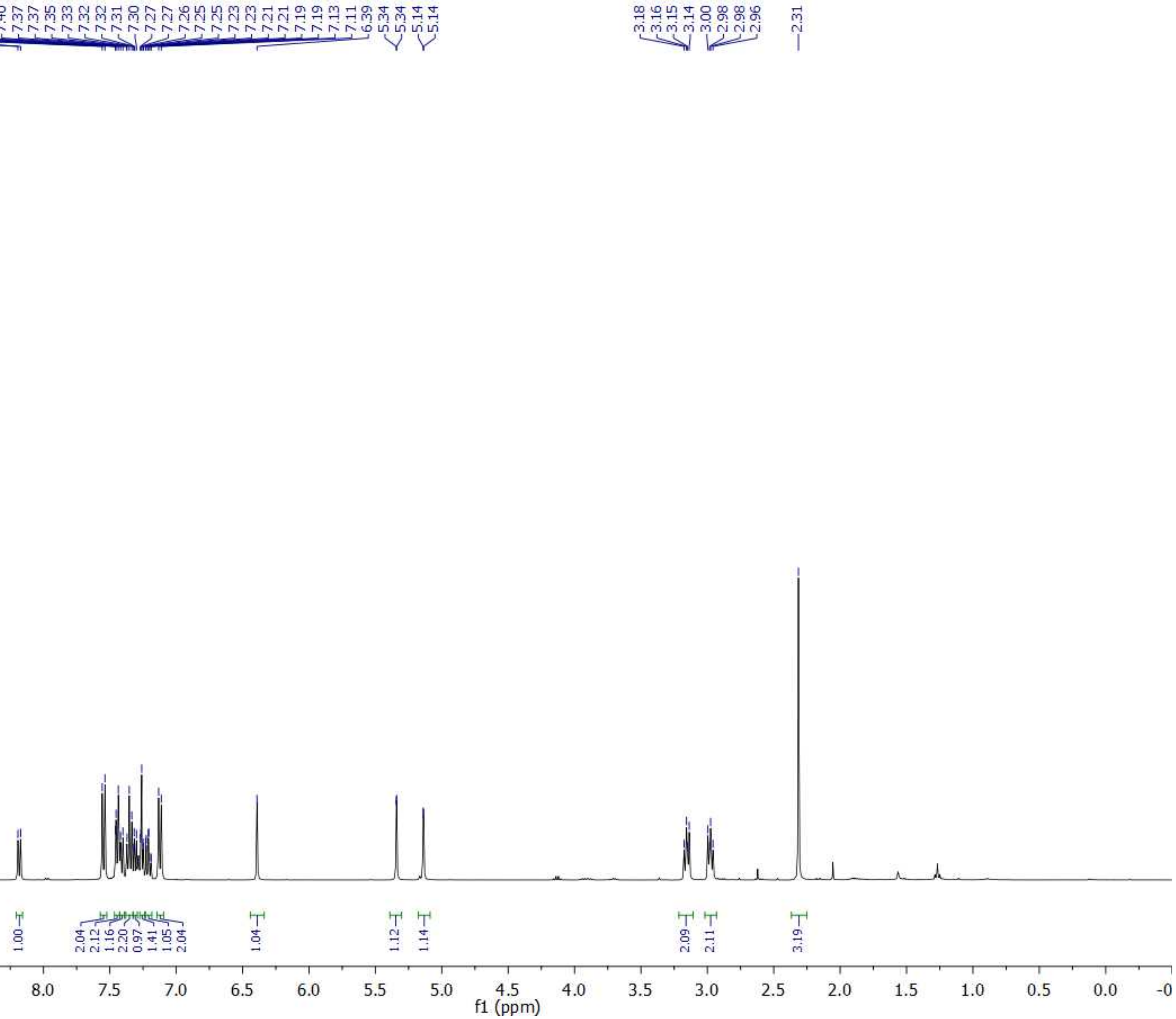

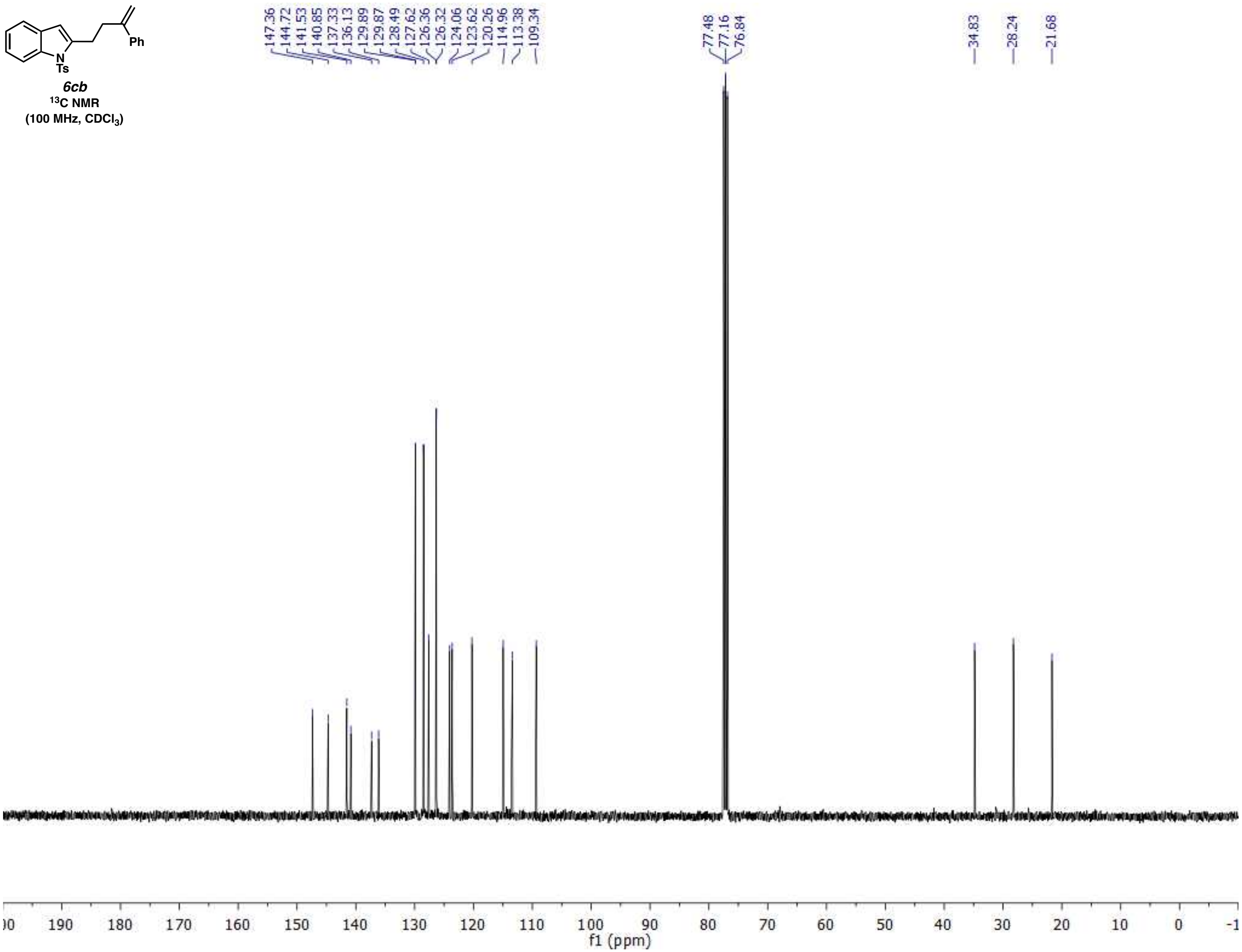

Ph
N
Ts
6cb
13C NMR
(100 MHz, CDCl3)
147.36
144.72
141.53
140.85
137.33
136.13
129.89
129.87
128.49
127.62
126.36
126.32
124.06
123.62
120.26
114.96
113.38
109.34
77.48
77.16
76.84
34.83
28.24
21.68
f1 (ppm)

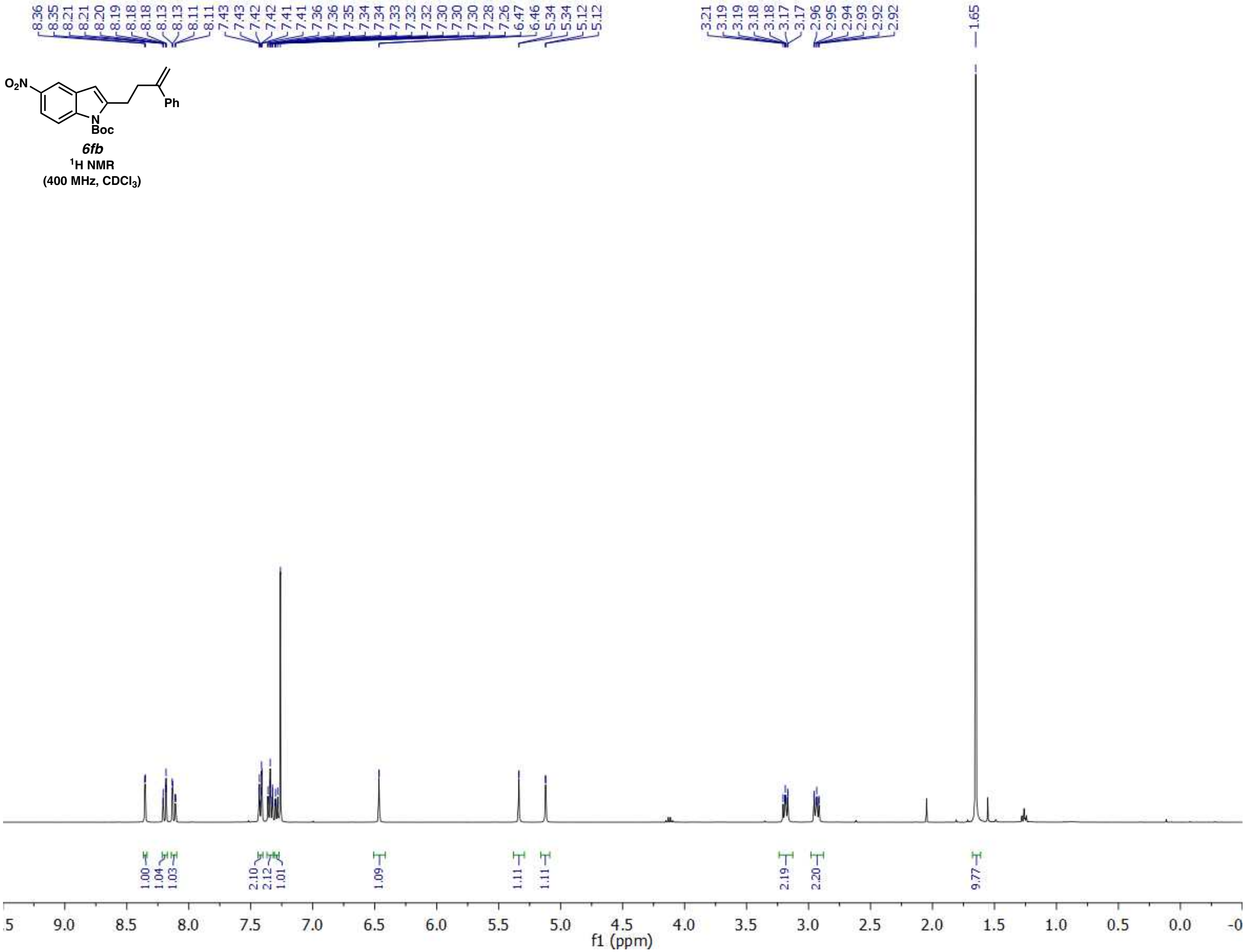

***6fb***
$^1H$ NMR
(400 MHz, $CDCl_3$)

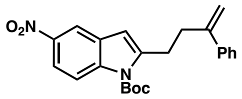

***6fb***
$^{13}C$ NMR
(100 MHz, $CDCl_3$)

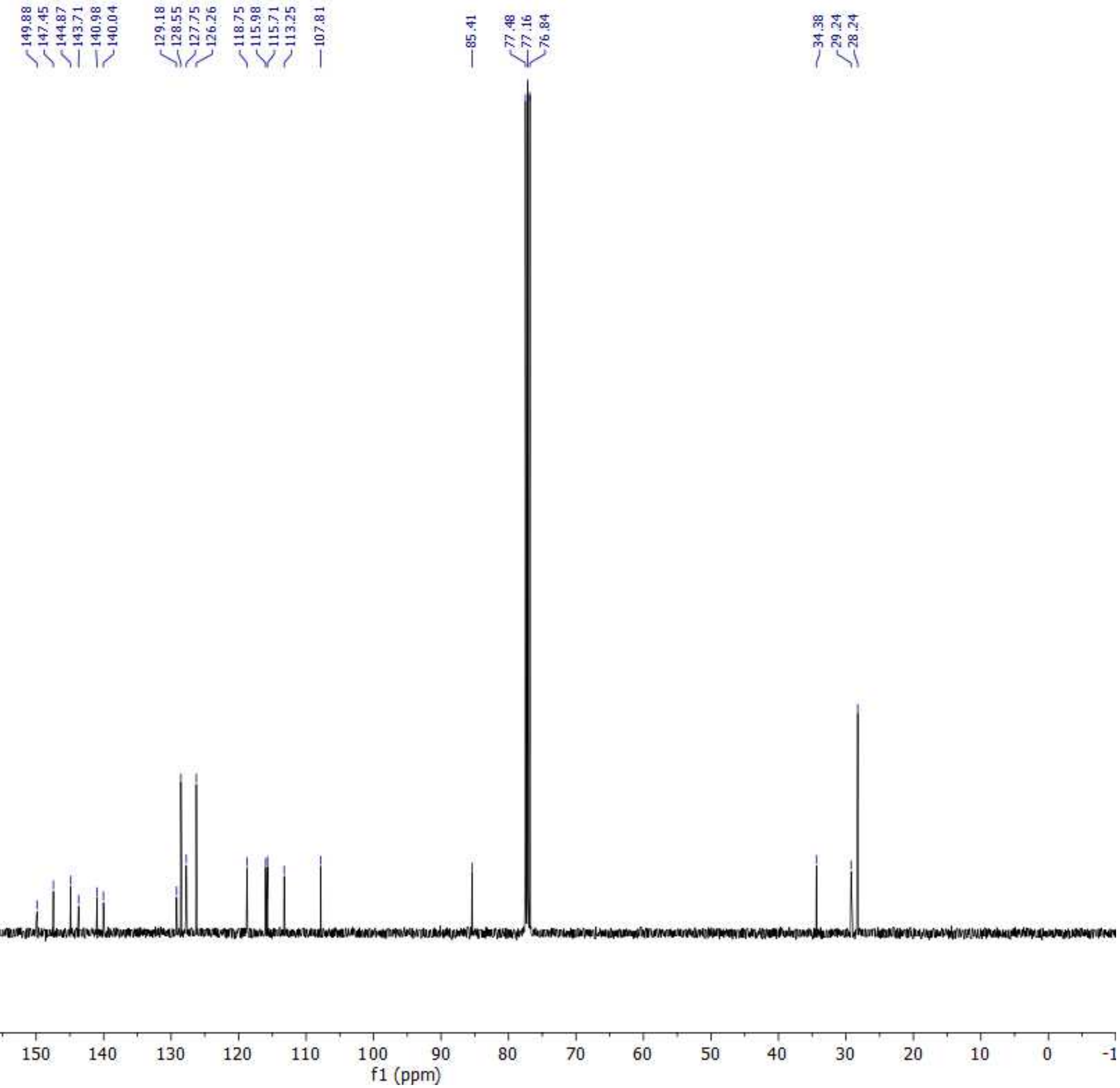

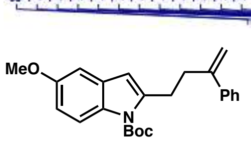

***6kb***
$^{1}$H NMR
(400 MHz, $CDCl_3$)

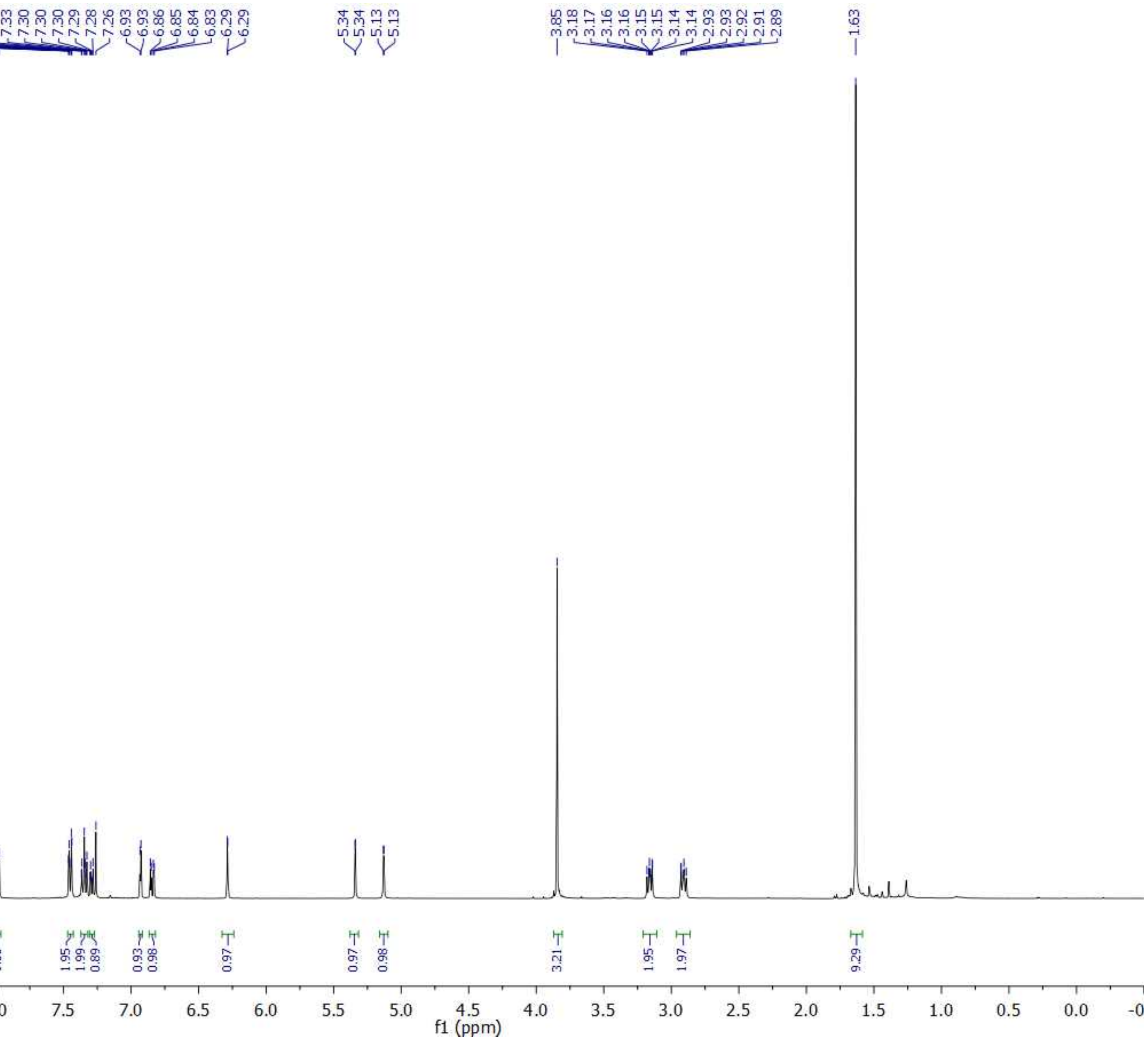

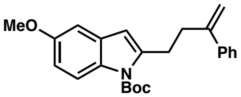


***6kb***
$^{13}$C NMR
(100 MHz, $CDCl_3$)

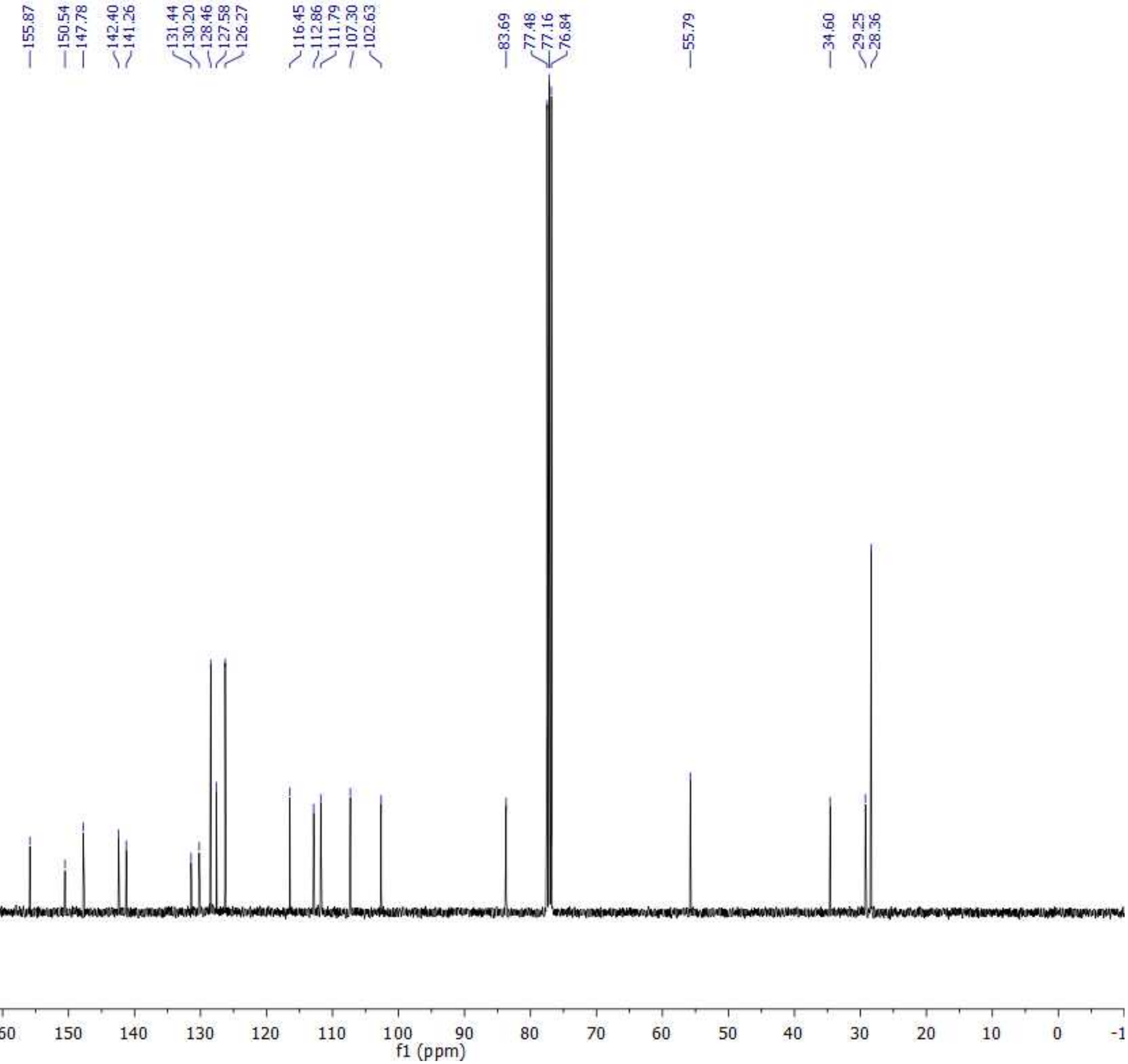

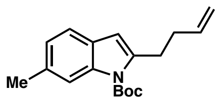


***6ga***
**$^1$H NMR**
**(600 MHz, $CDCl_3$)**

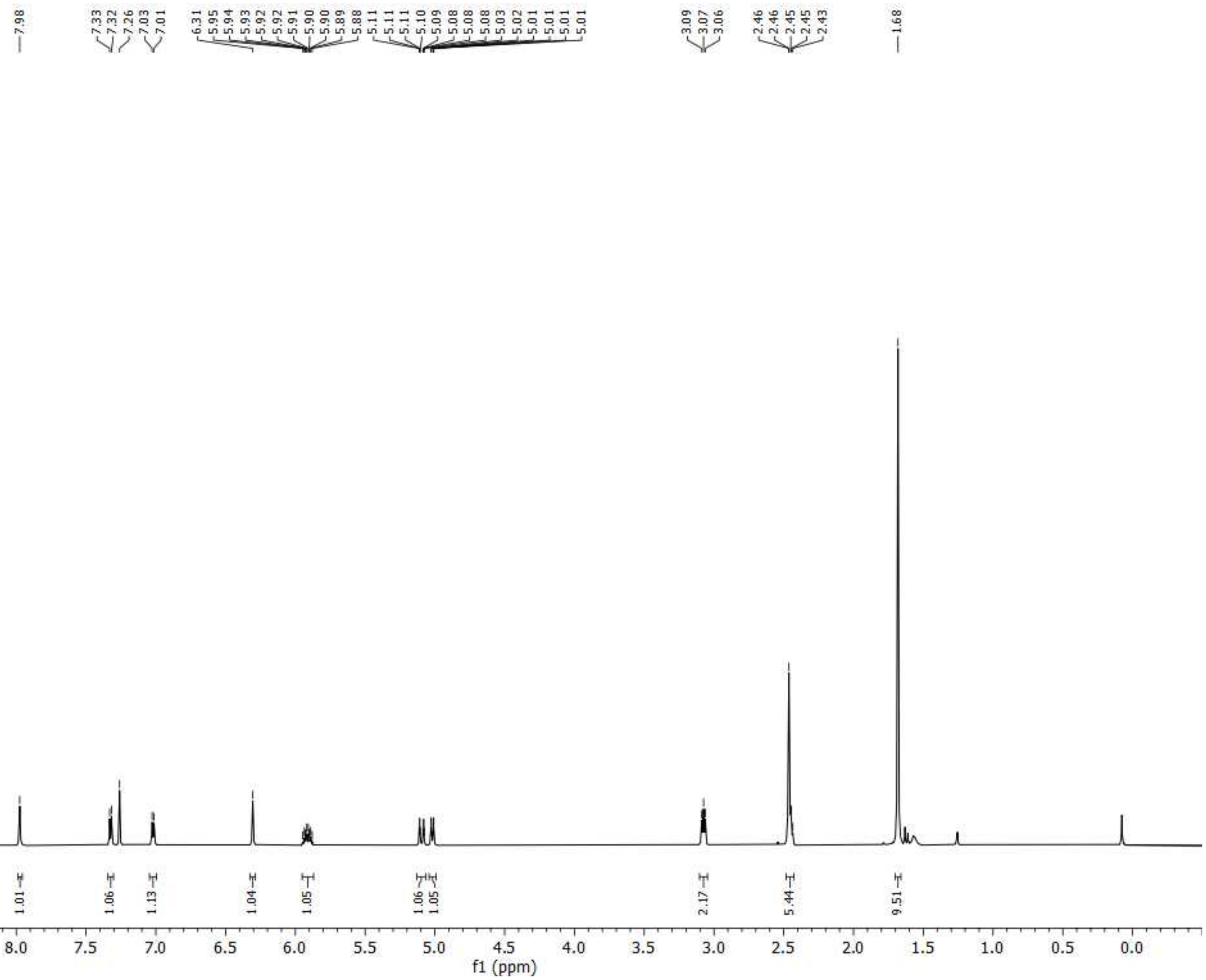

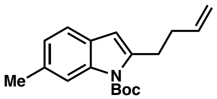

**6ga**
$^{13}$C NMR
(100 MHz, $CDCl_3$)

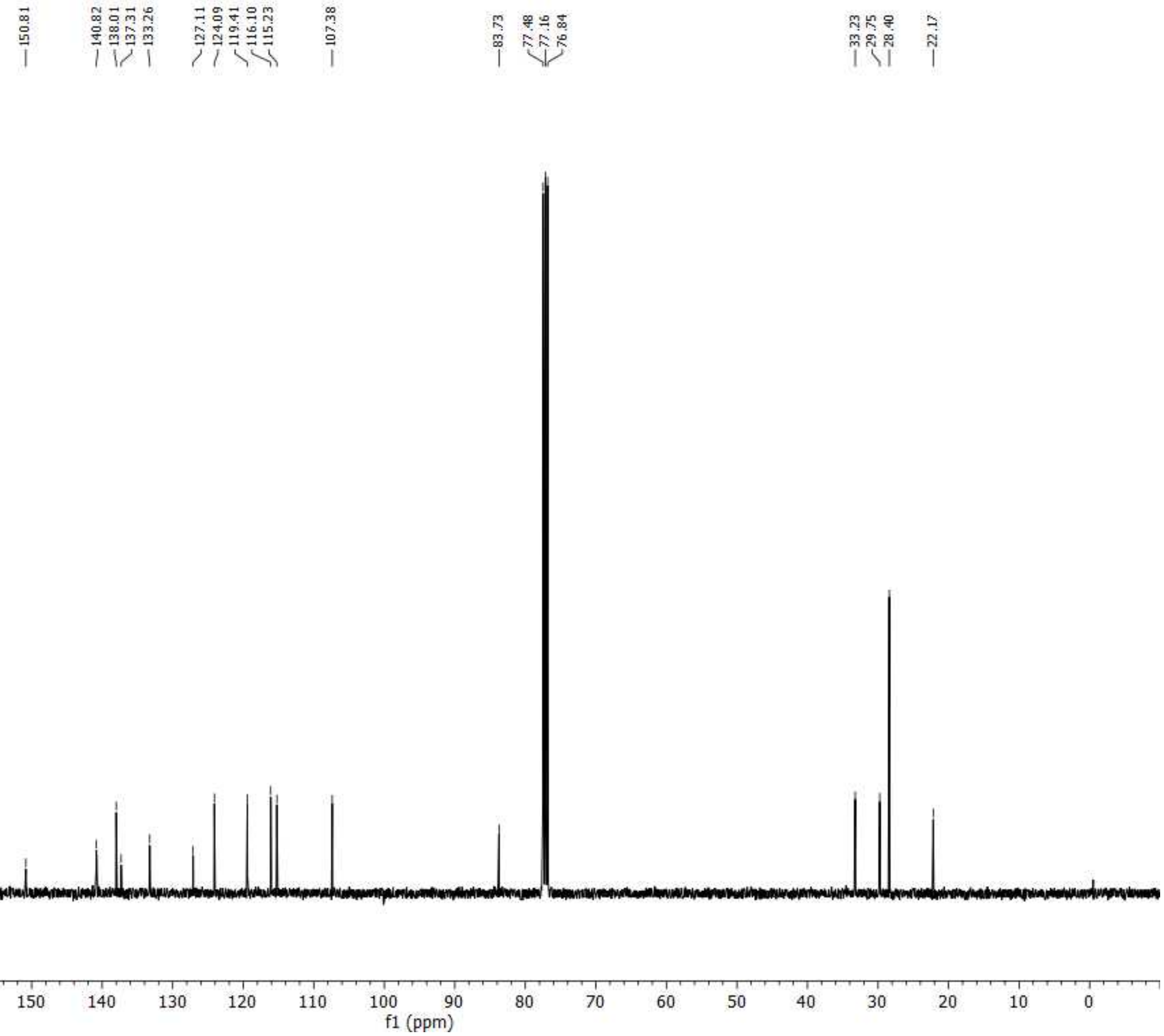

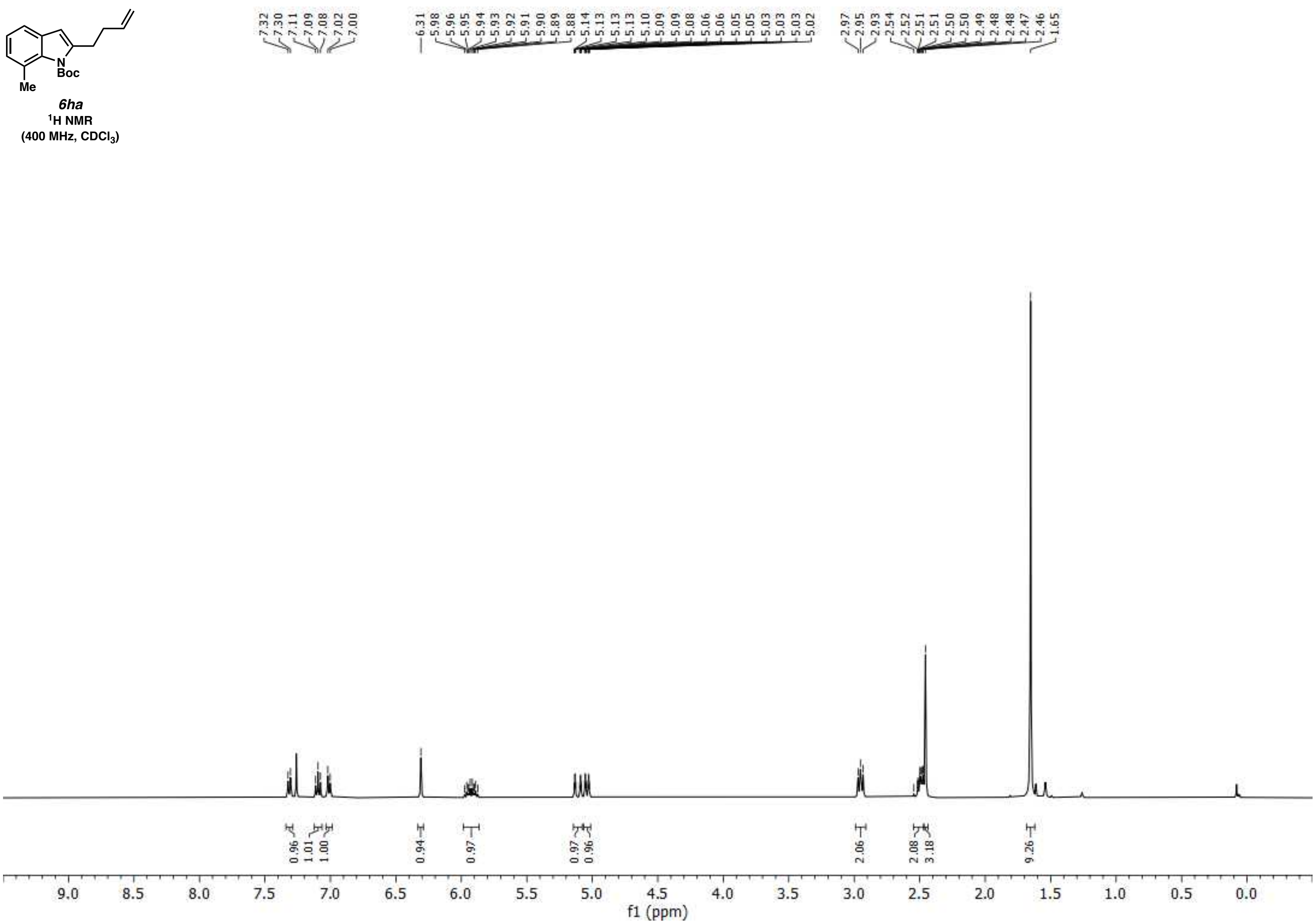
N
Boc
Me
6ha
¹H NMR
(400 MHz, CDCl3)
f1 (ppm)

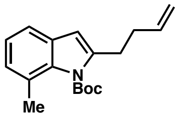

*6ha*
$^{13}C$ NMR
(100 MHz, $CDCl_3$)

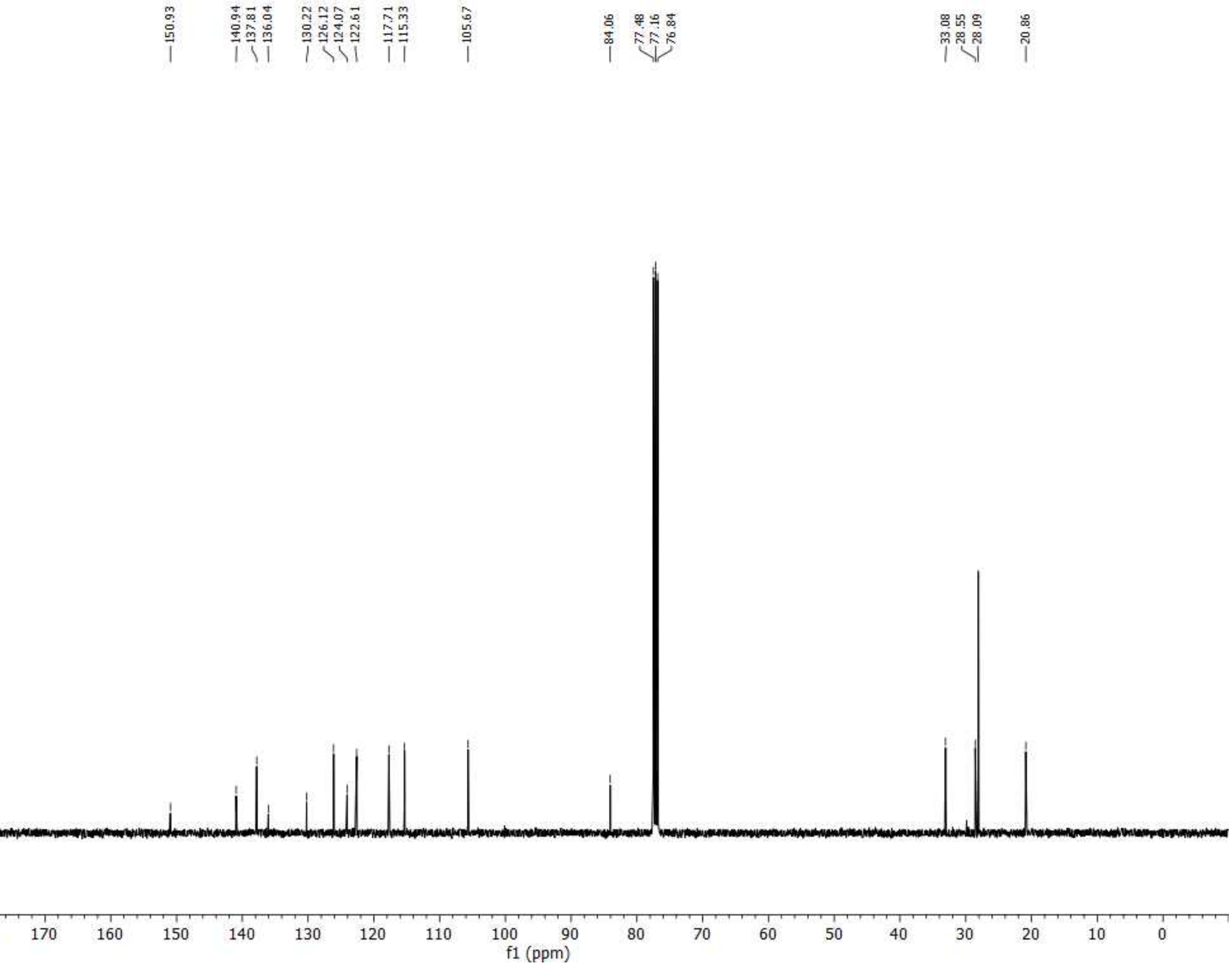

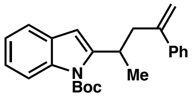

**6lb**
$^1$H NMR
(400 MHz, $CDCl_3$)

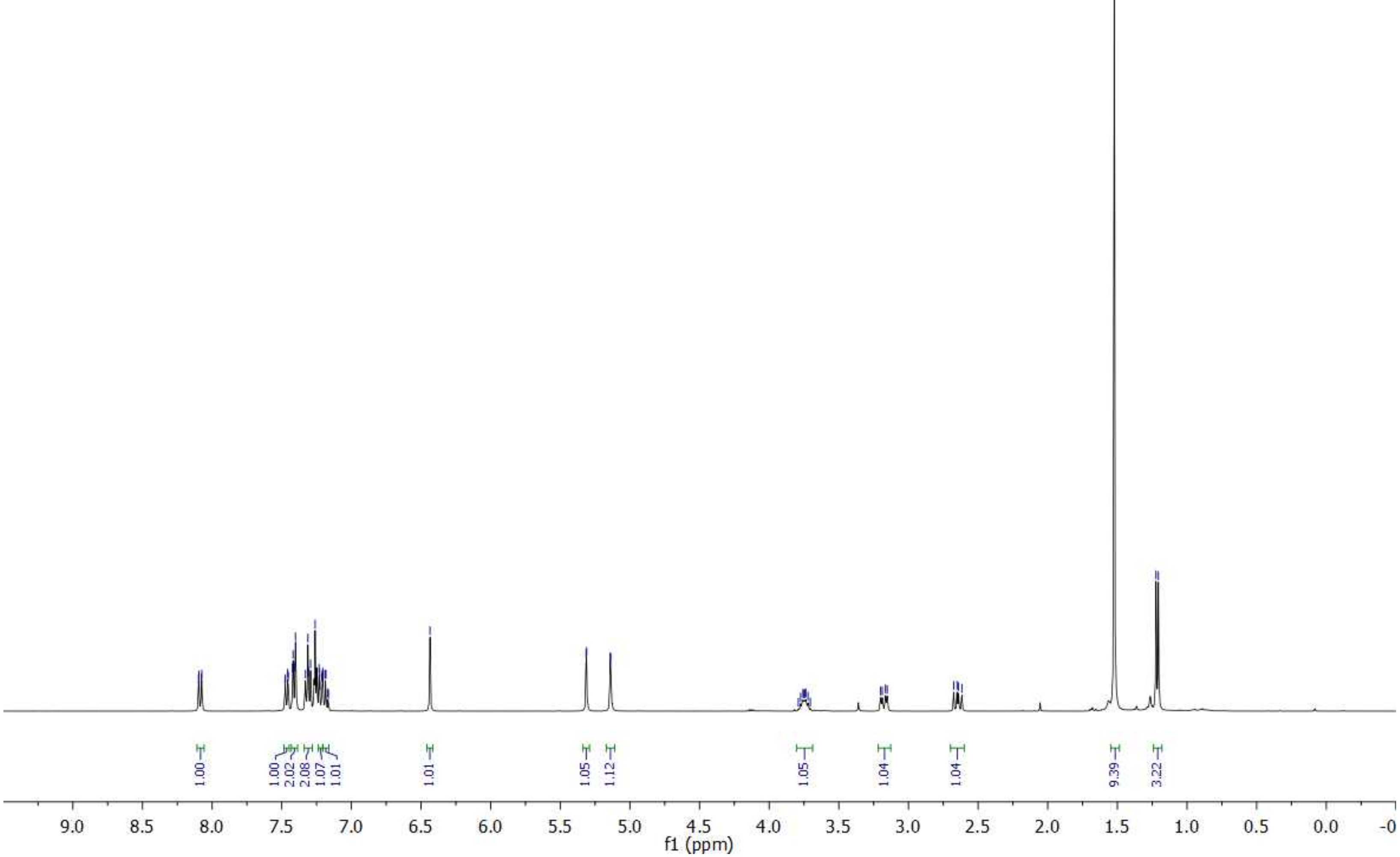

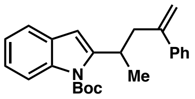


***6lb***
$^{13}$C NMR
(100 MHz, $CDCl_3$)

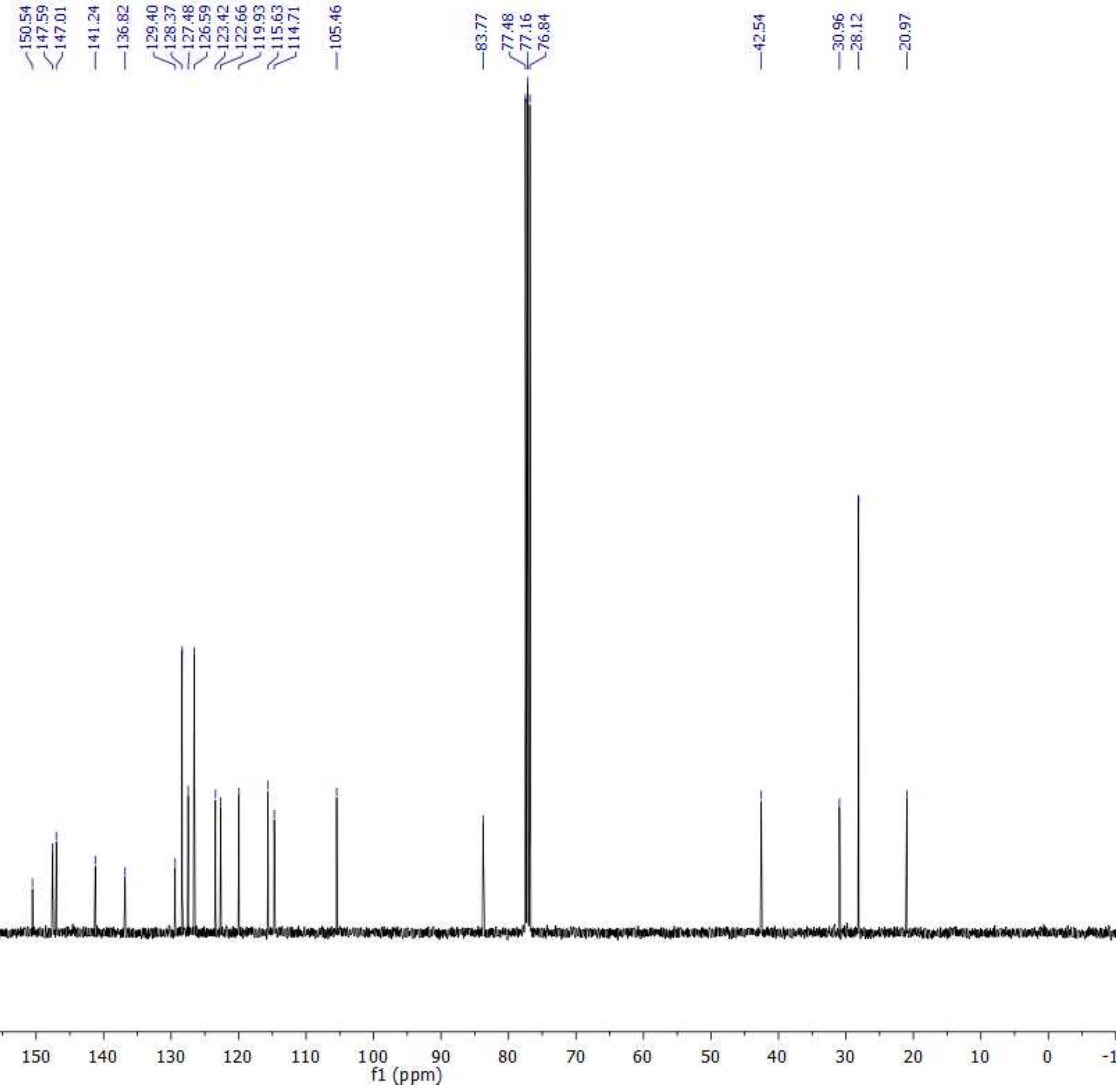

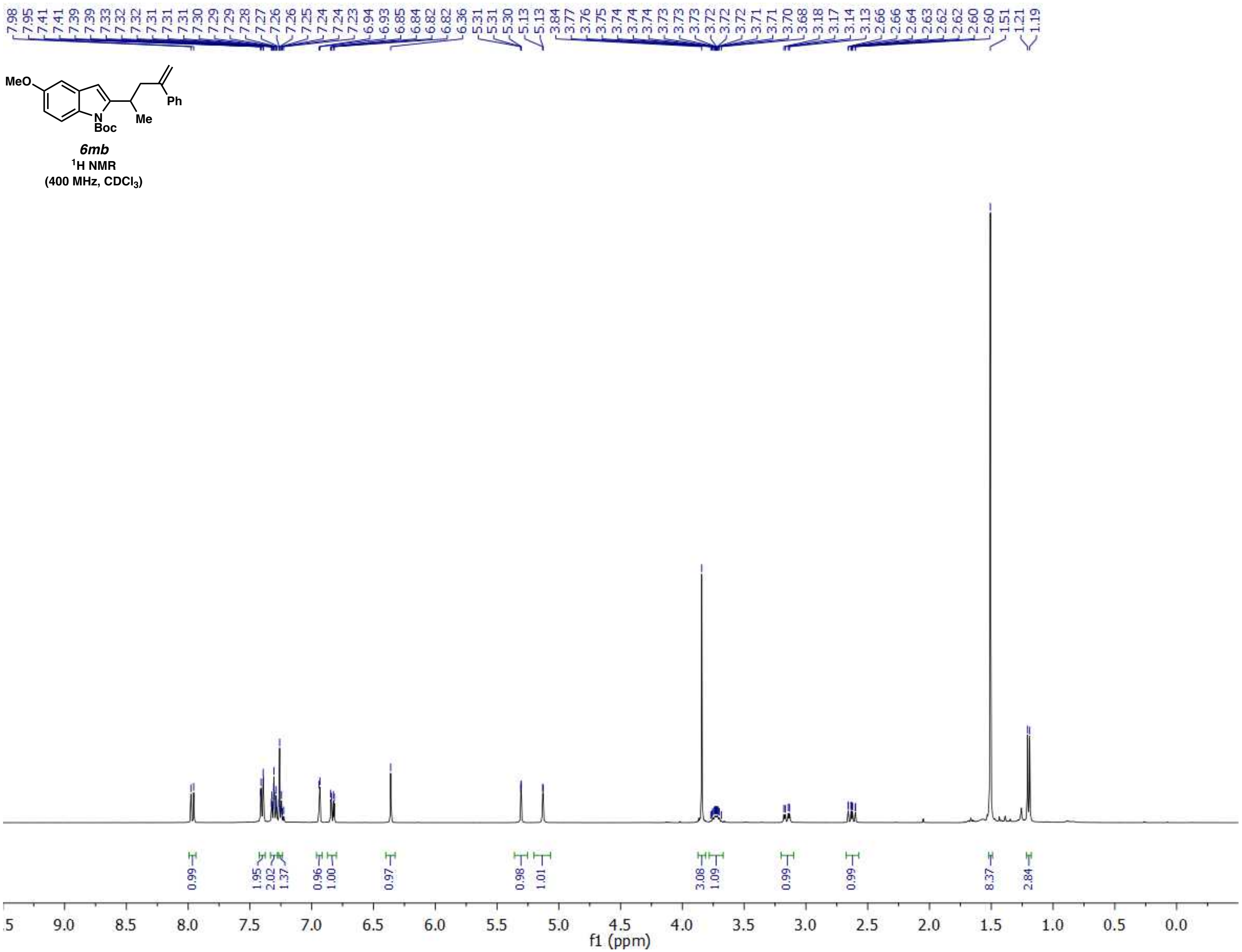
MeO
N
Boc
Me
Ph
**6mb**
$^1$H NMR
(400 MHz, $CDCl_3$)
f1 (ppm)

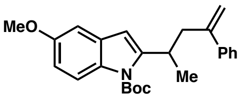


***6mb***
$^{13}$C NMR
(100 MHz, $CDCl_3$)

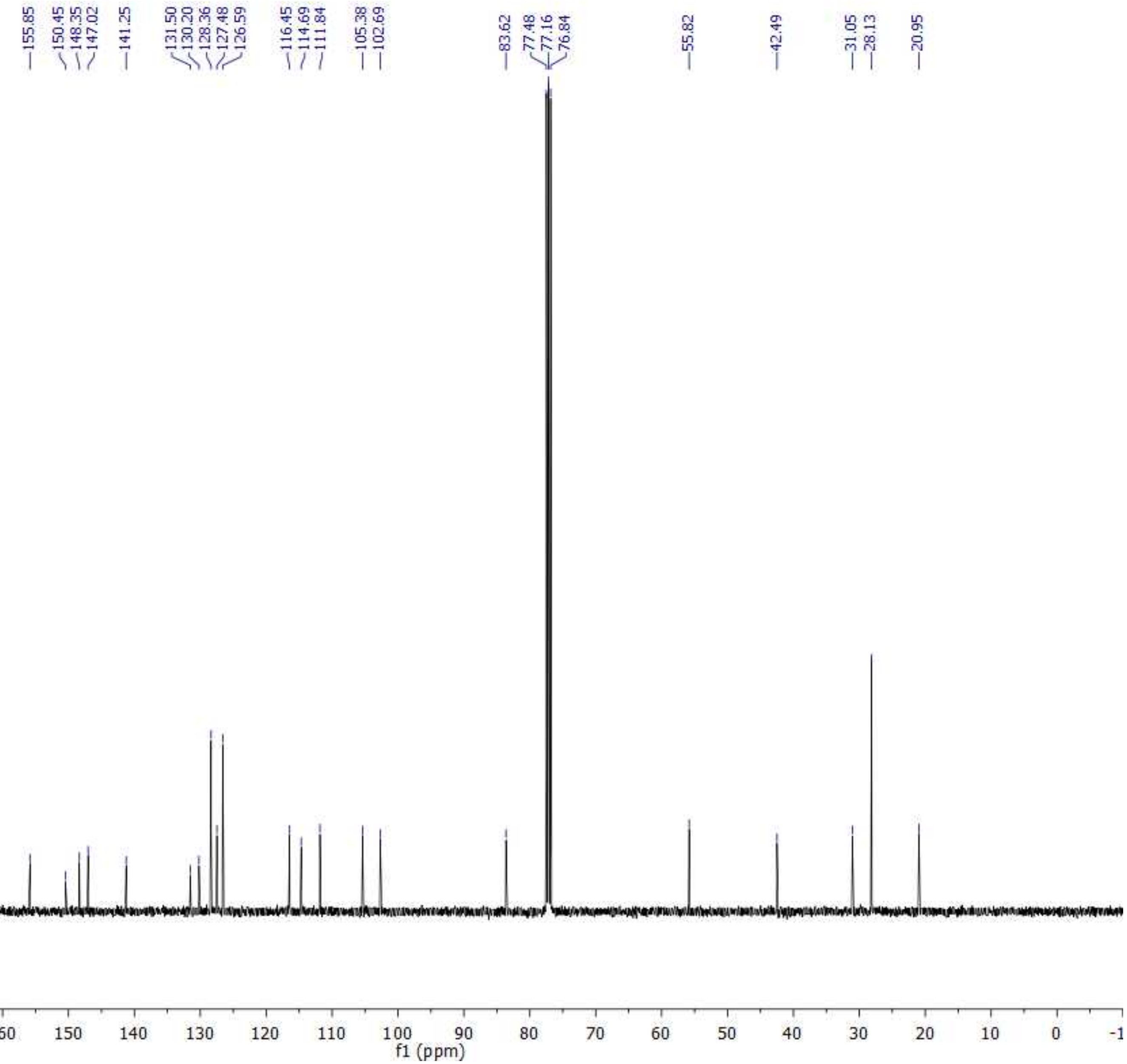

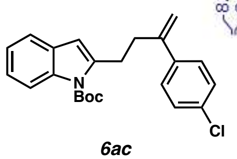


***6ac***
**$^{1}$H NMR**
**(400 MHz, $CDCl_3$)**

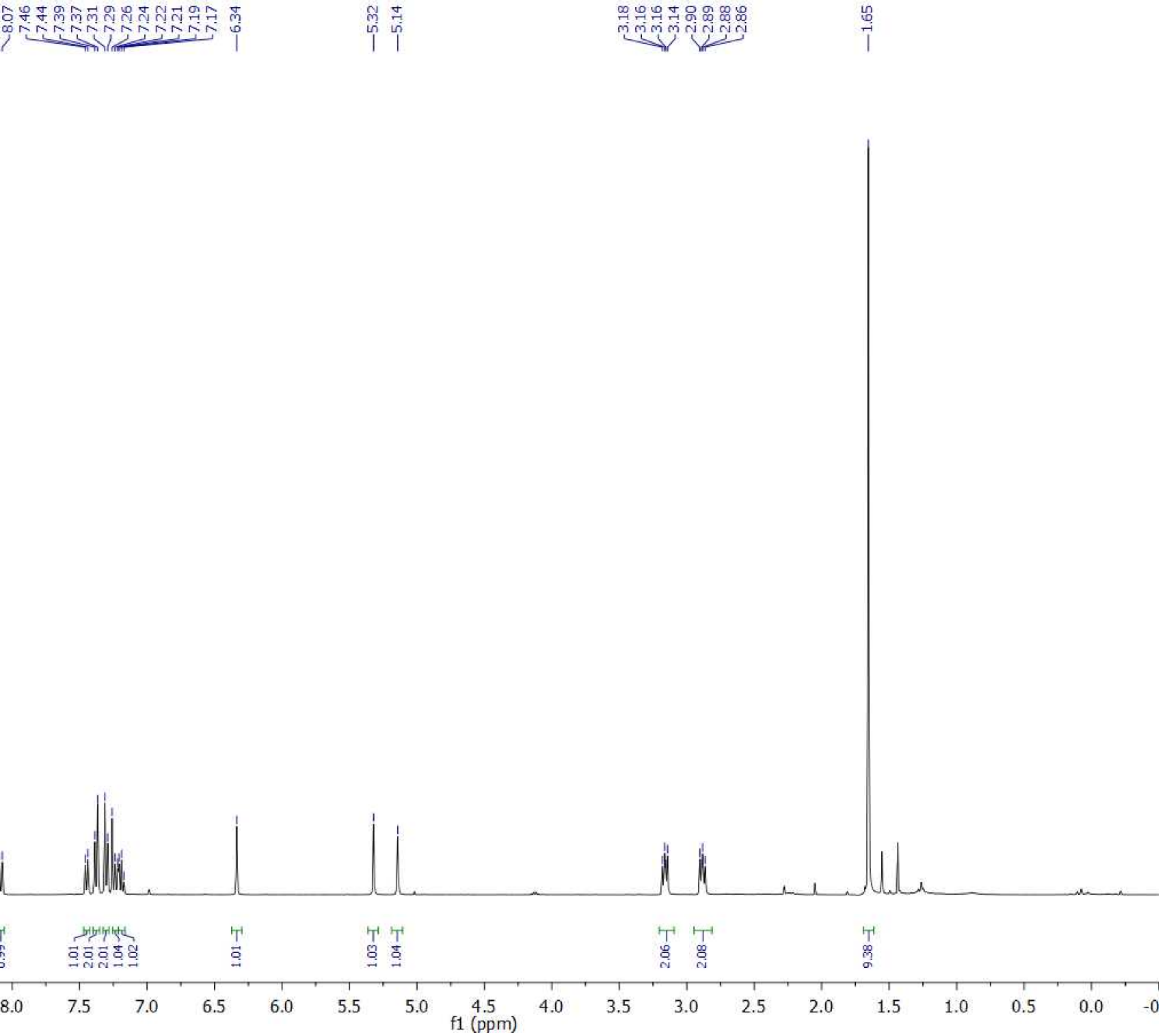

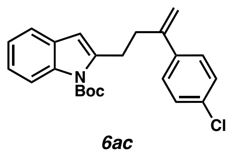


***6ac***
**$^{13}C$ NMR**
**(100 MHz, $CDCl_3$)**

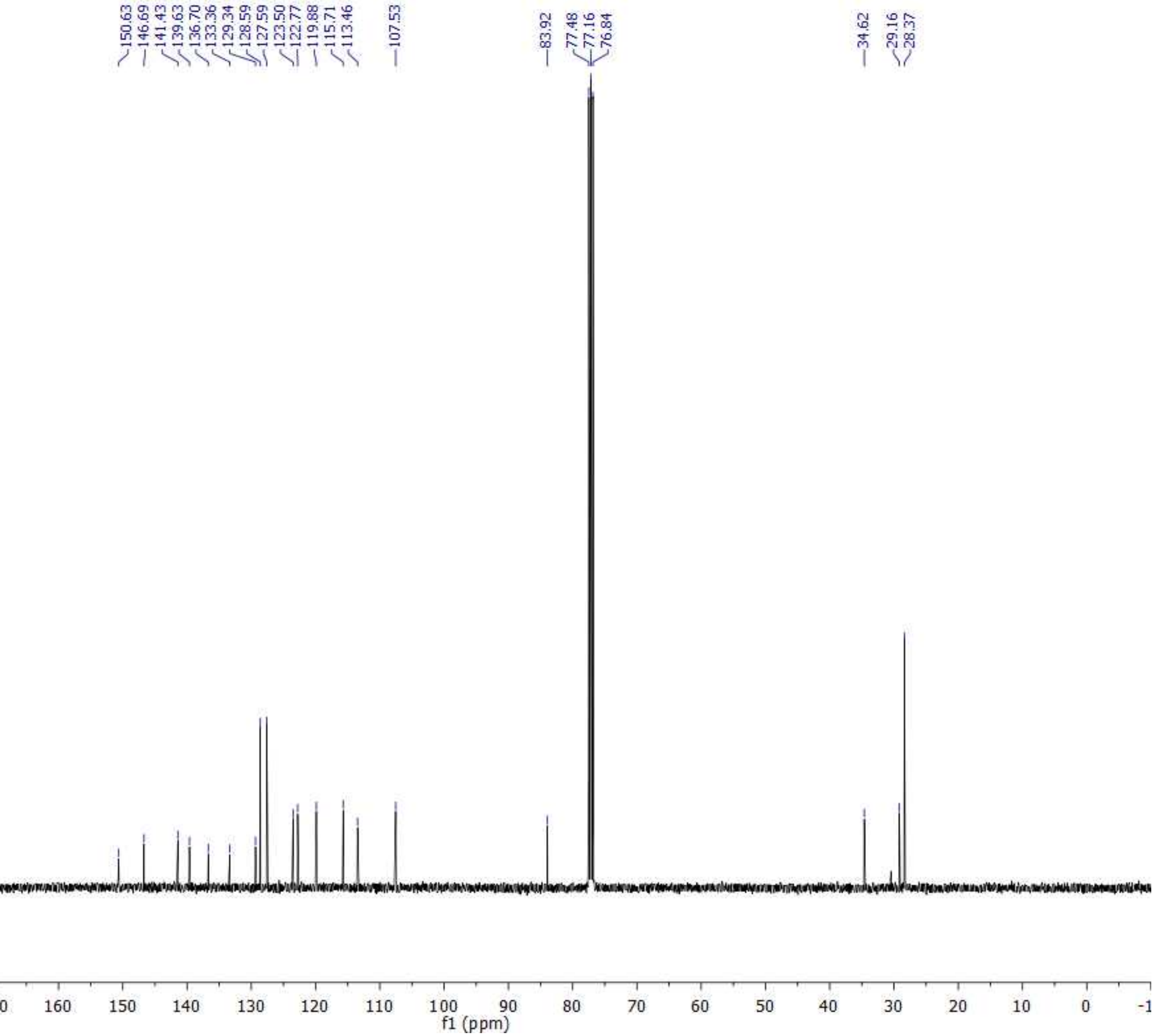

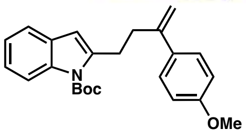

***6ad***
$^1H$ NMR
(400 MHz, $CDCl_3$)

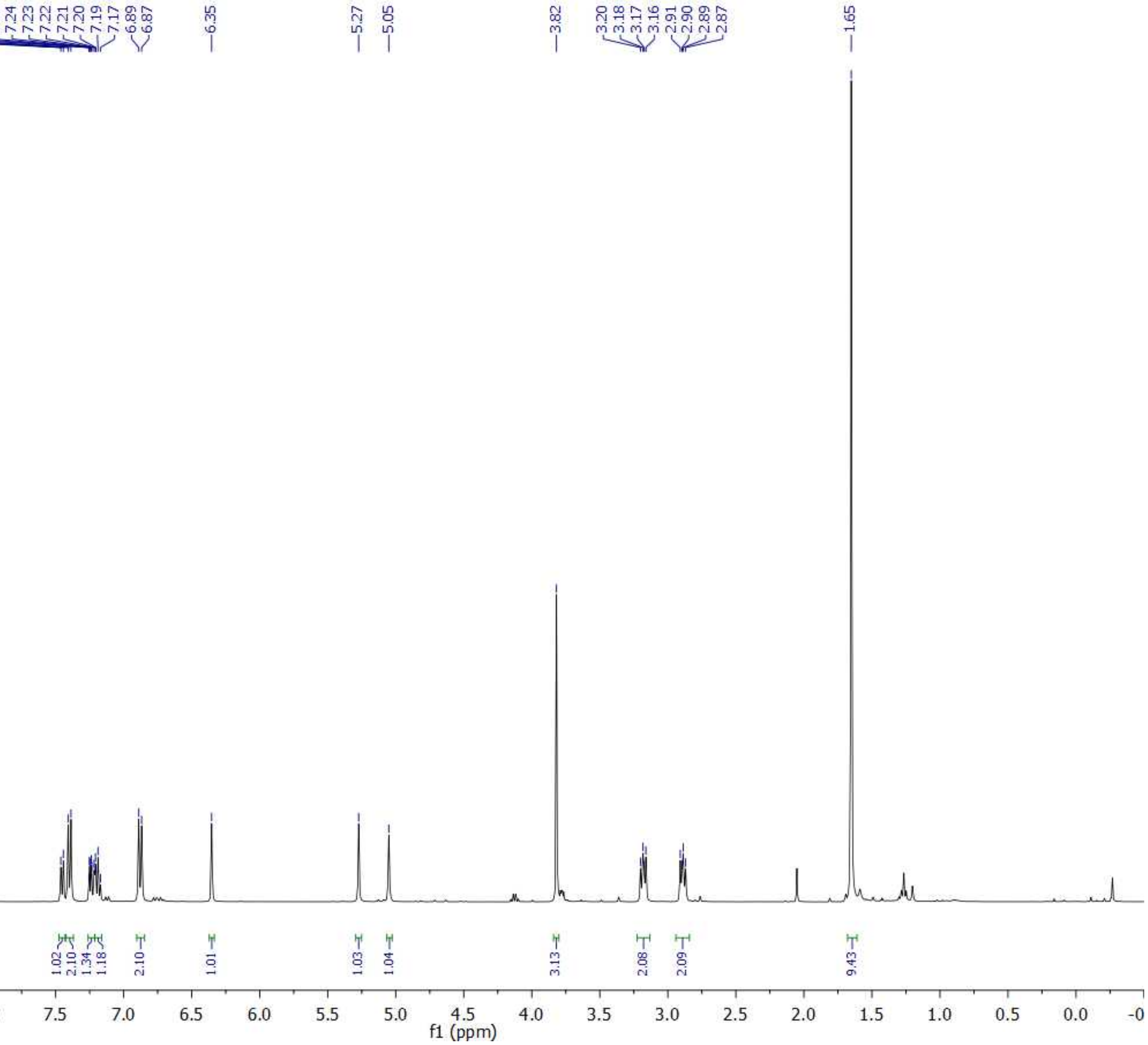

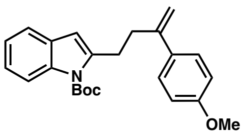

***6ad***
$^{13}C$ NMR
(100 MHz, $CDCl_3$)

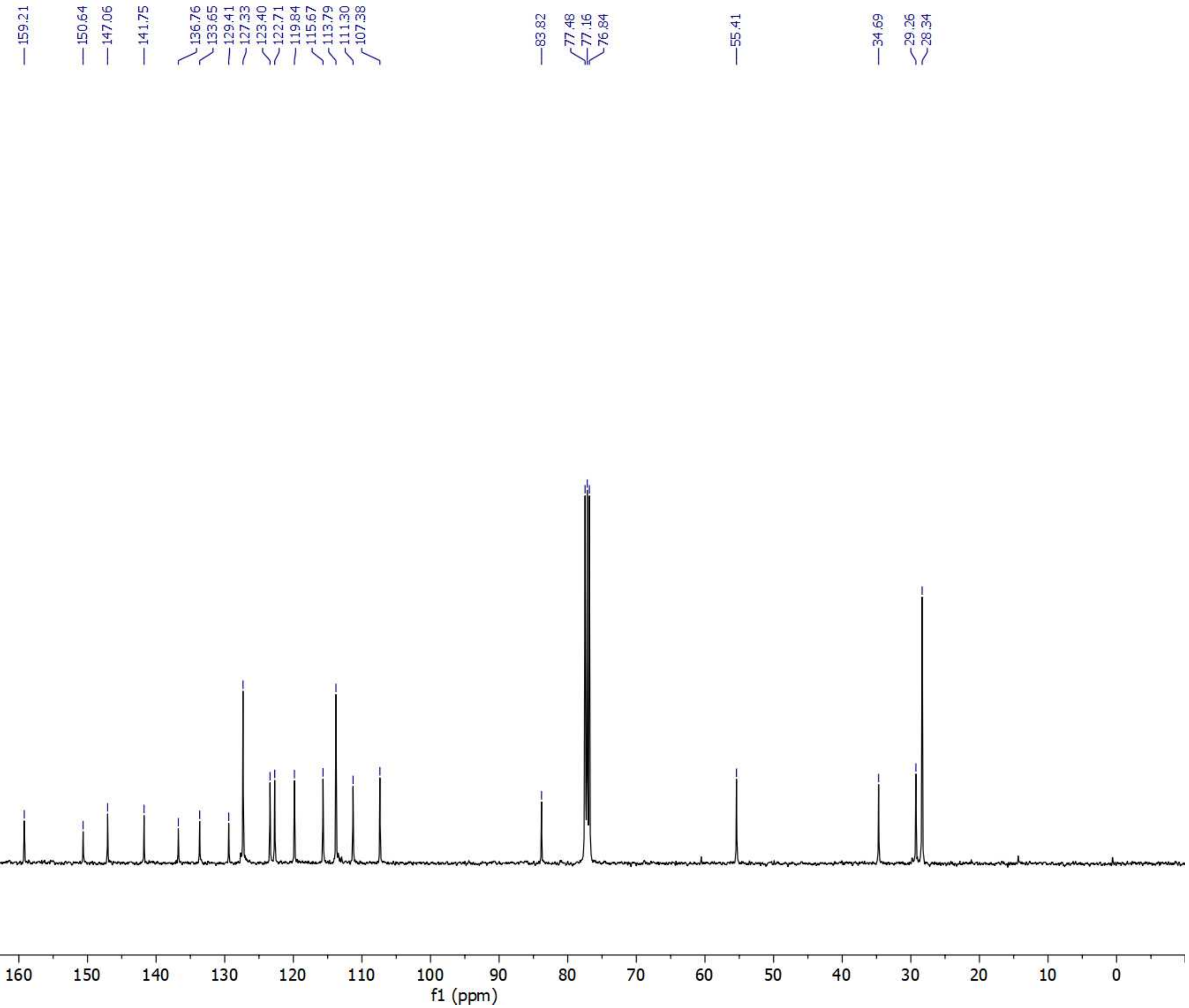

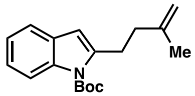


*6af*
$^1$H NMR
(400 MHz, $CDCl_3$)

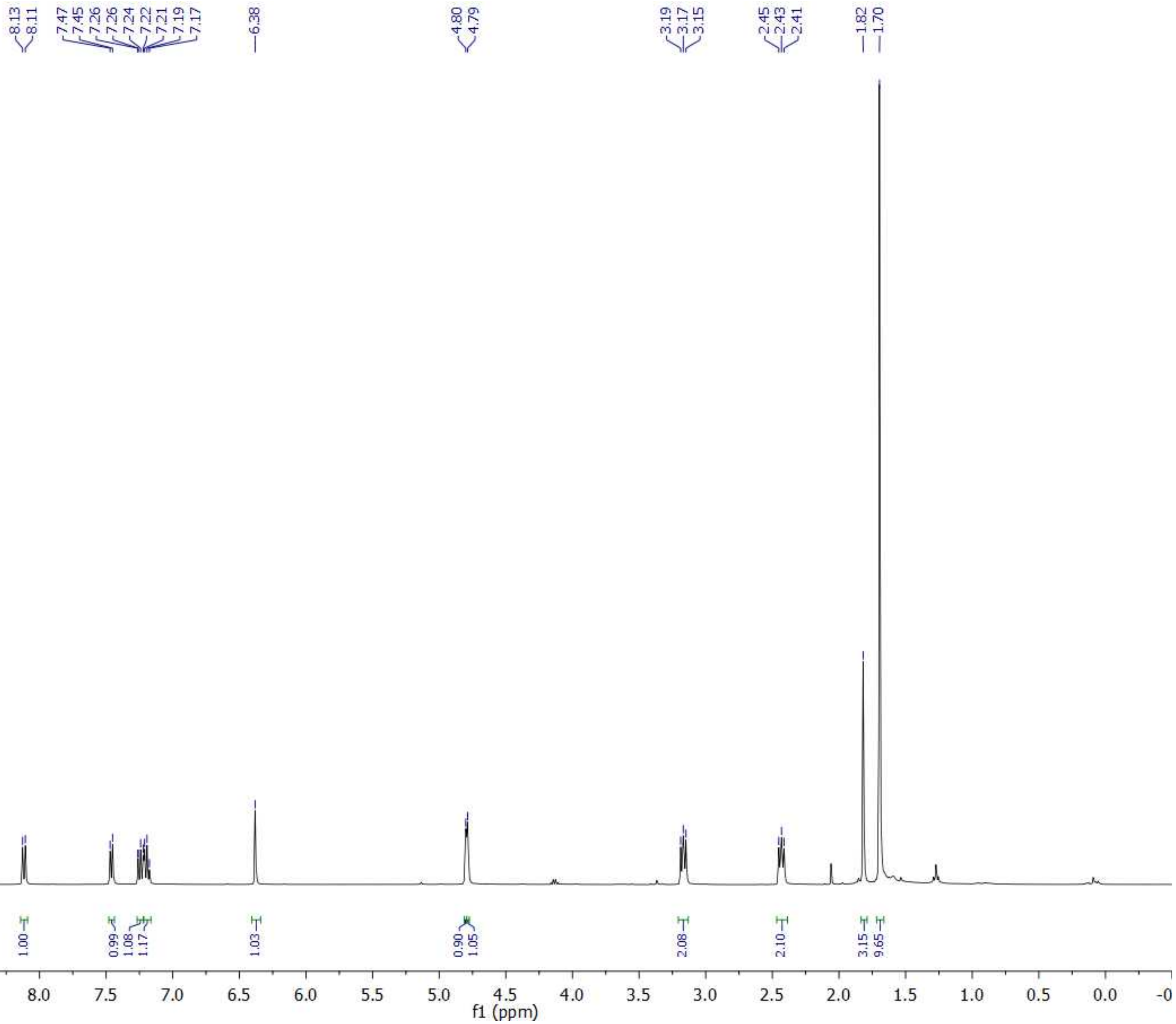

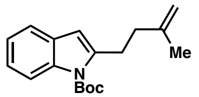


***6af***
$^{13}C$ NMR
(100 MHz, $CDCl_3$)

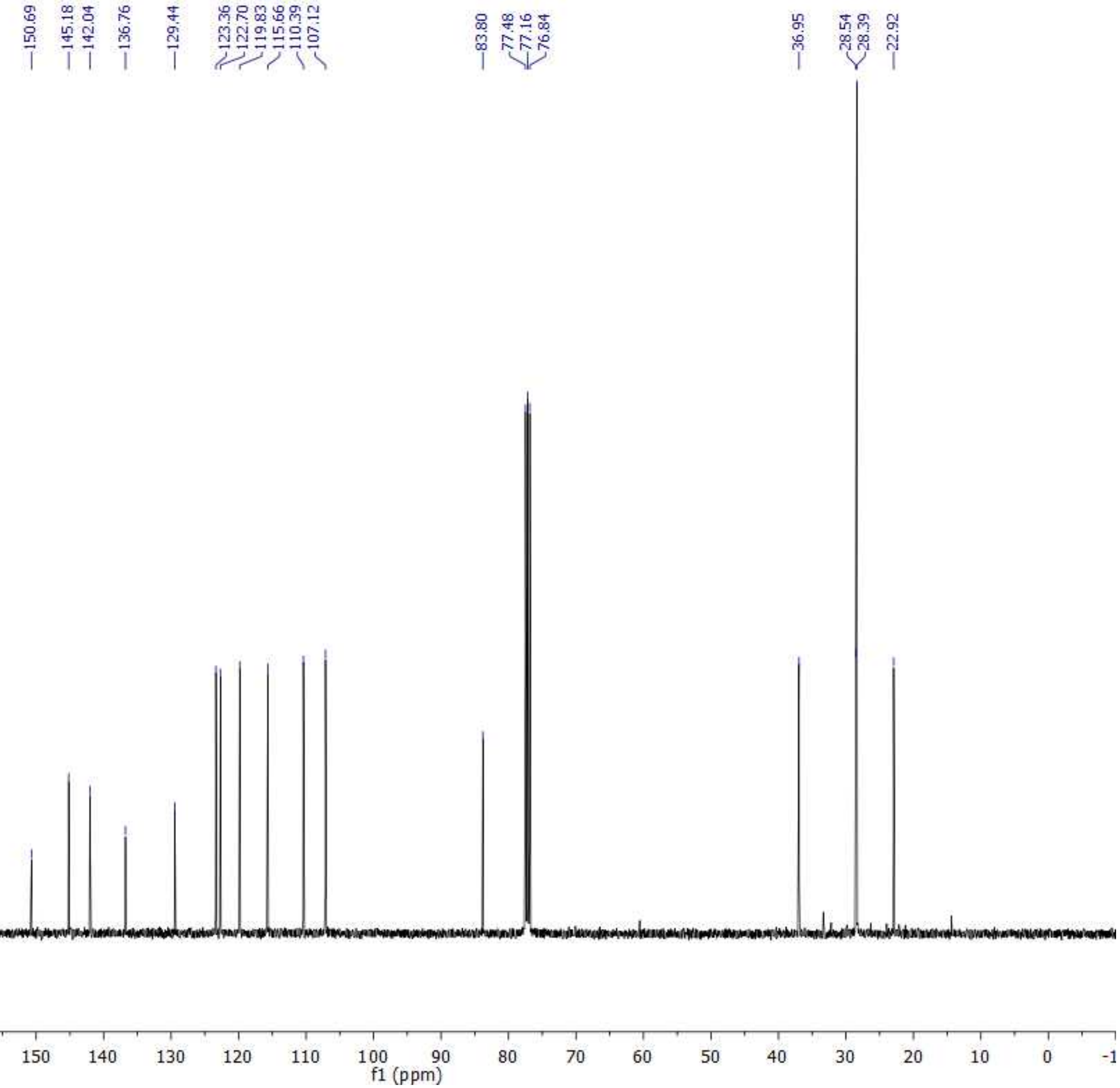

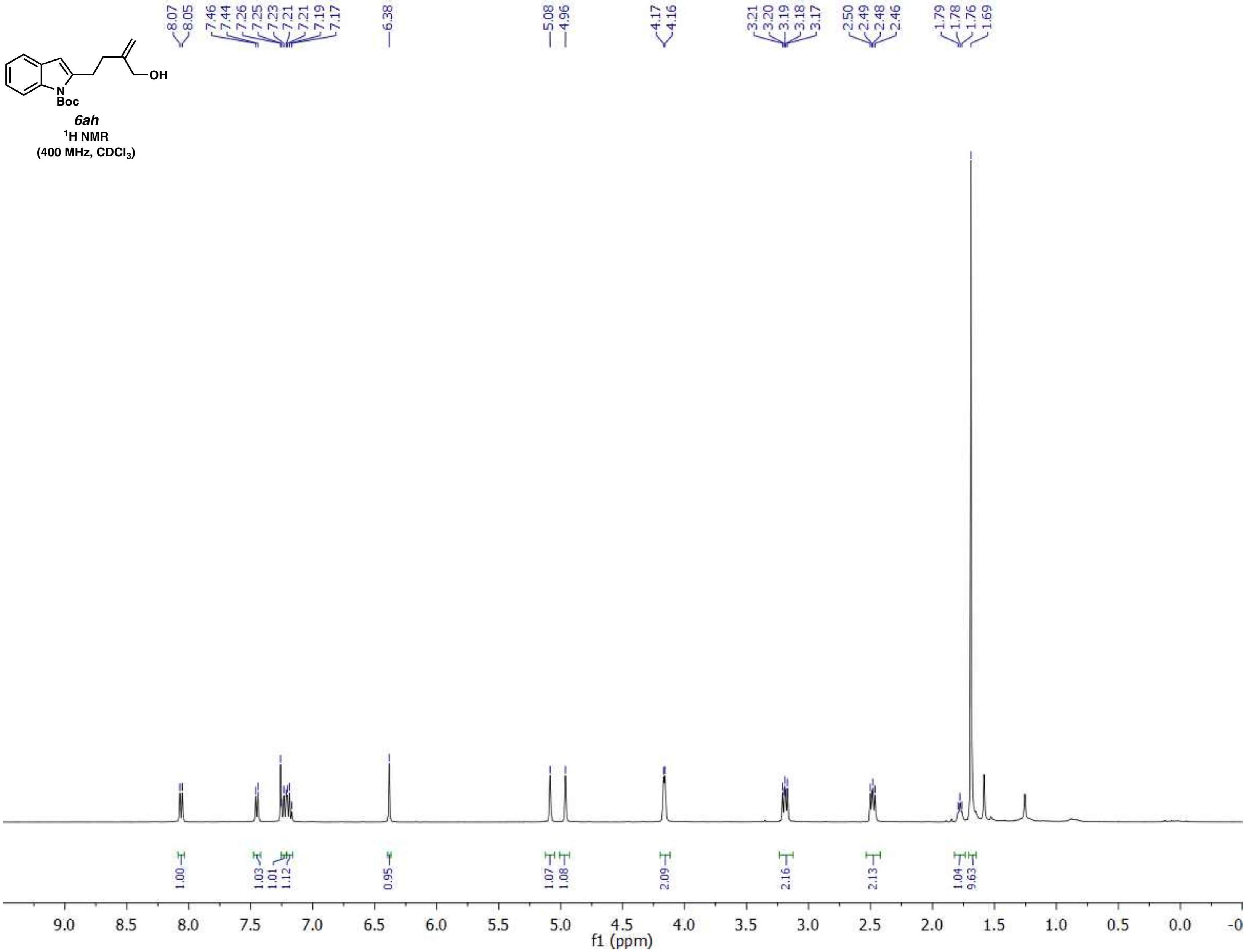


***6ah***
**$^1$H NMR**
**(400 MHz, $CDCl_3$)**

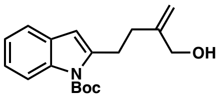

***6ah***
$^{13}$C NMR
(100 MHz, $CDCl_3$)

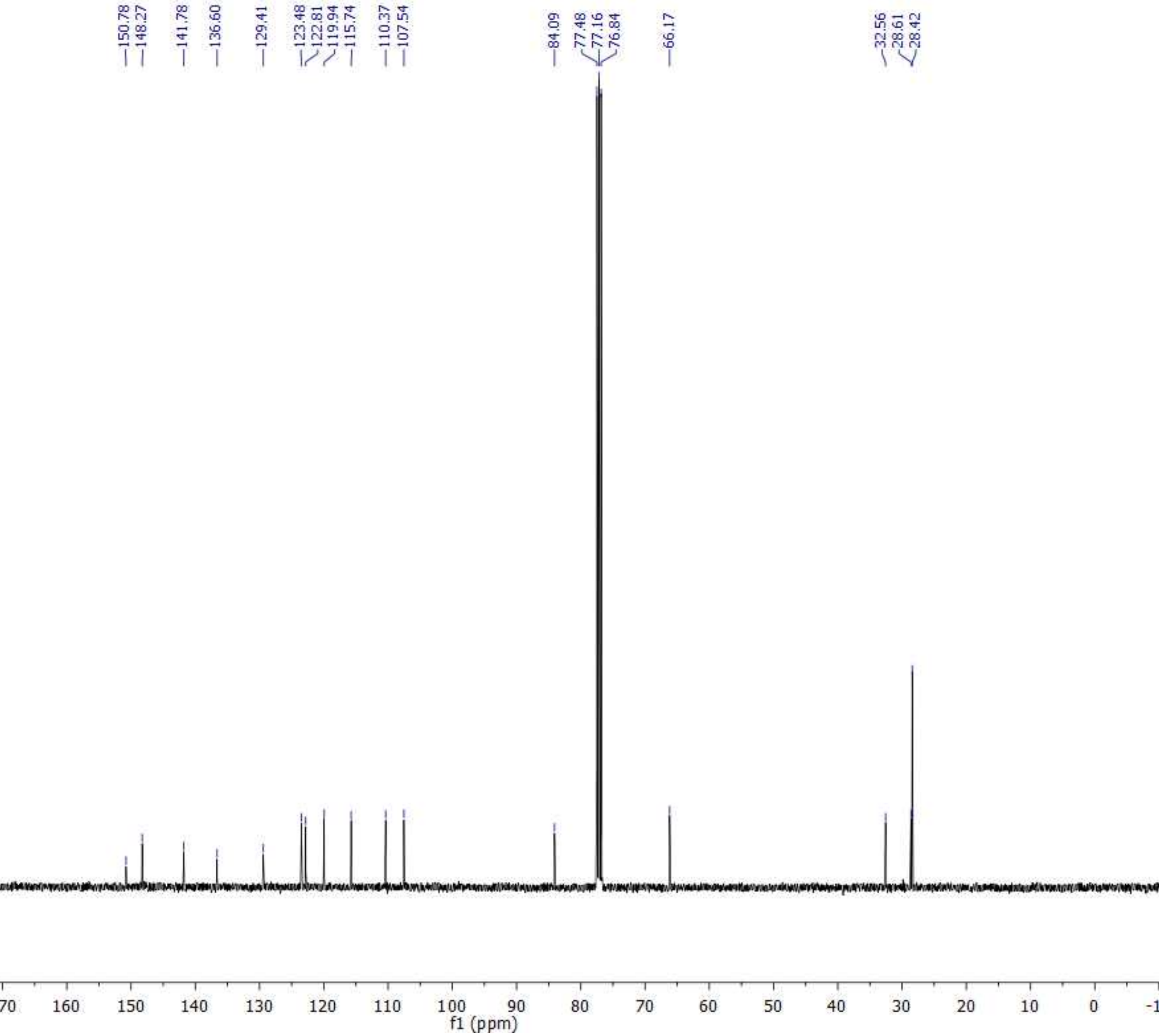

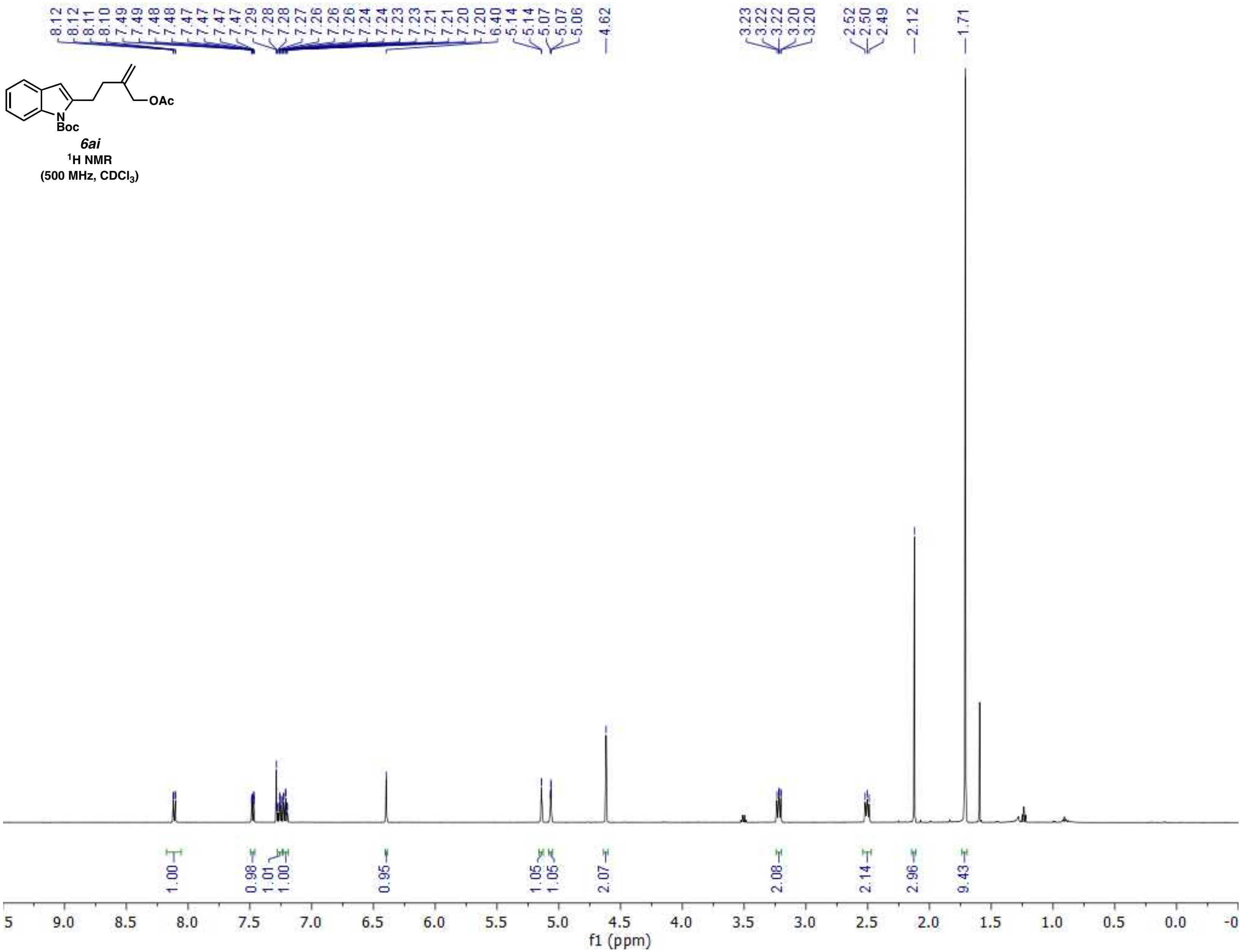
OAc
N
Boc
6ai
¹H NMR
(500 MHz, CDCl₃)
8.12
8.12
8.11
8.10
7.49
7.49
7.48
7.48
7.47
7.47
7.47
7.47
7.29
7.28
7.28
7.27
7.26
7.26
7.26
7.24
7.24
7.23
7.23
7.21
7.21
7.20
7.20
6.40
5.14
5.14
5.07
5.07
5.06
4.62
3.23
3.22
3.22
3.20
3.20
2.52
2.50
2.49
2.12
1.71
1.00
0.98
1.01
1.00
0.95
1.05
1.05
2.07
2.08
2.14
2.96
9.43
f1 (ppm)

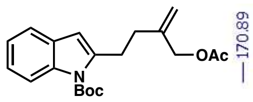


***6ai***
**$^{13}$C NMR**
**(125 MHz, $CDCl_3$)**

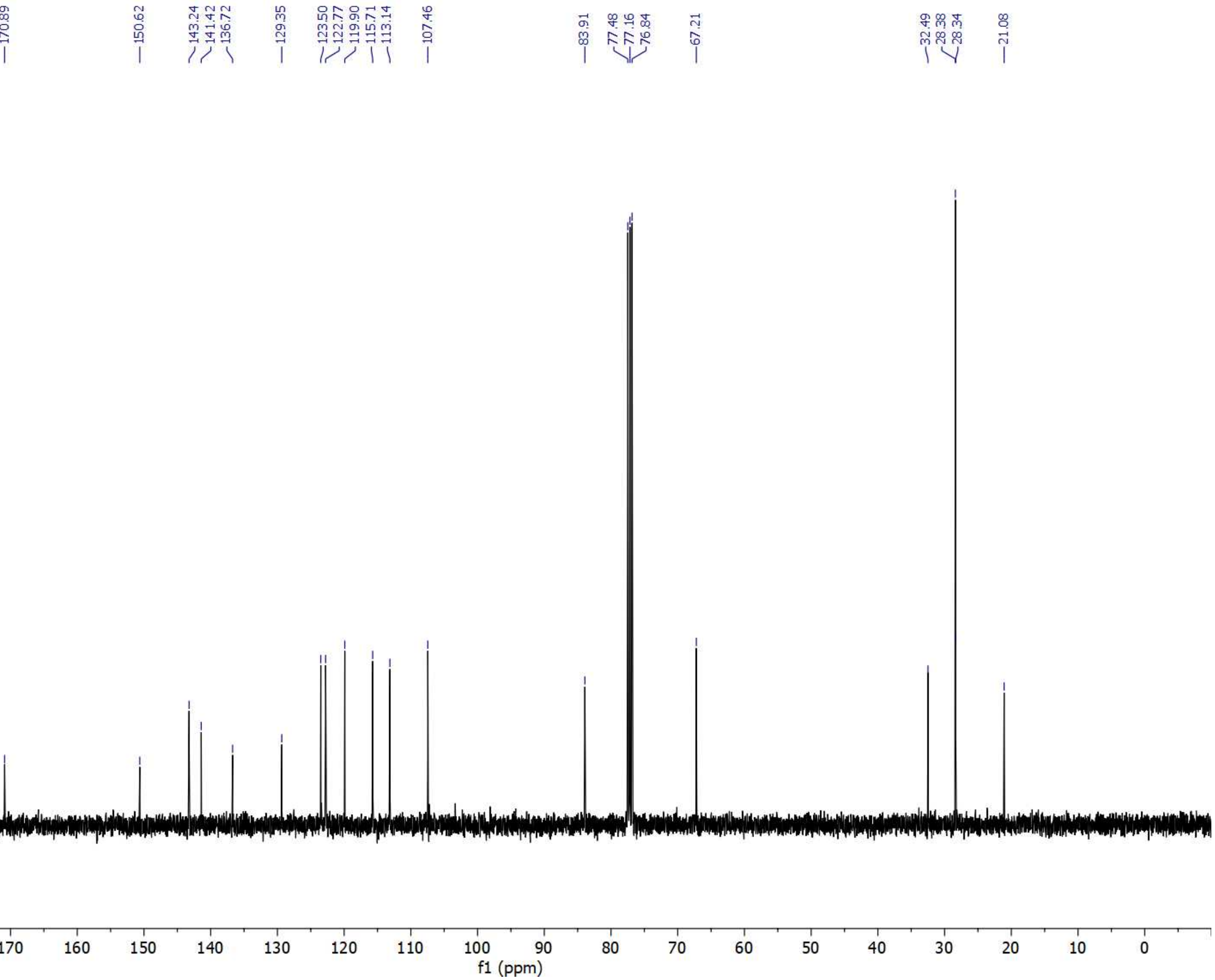

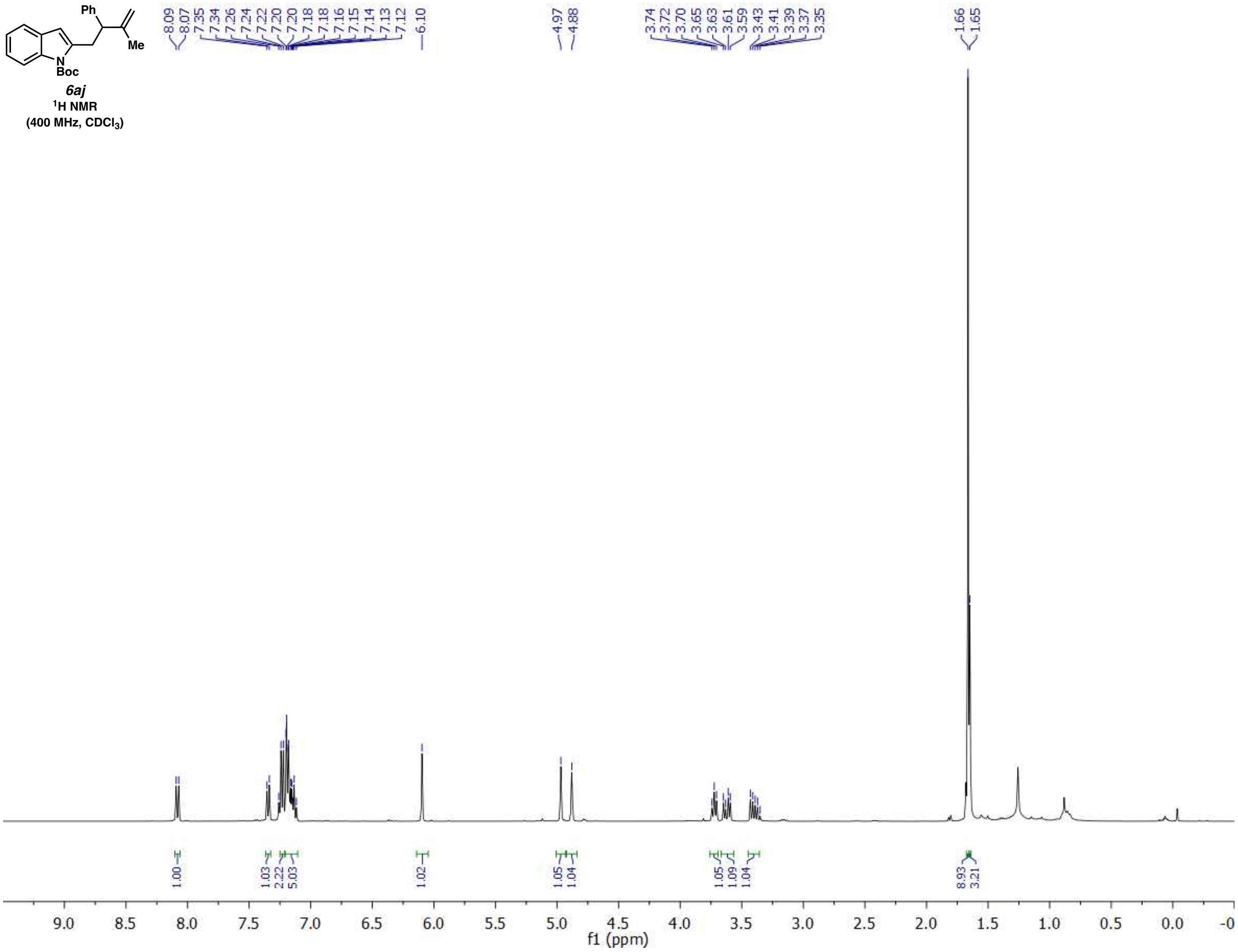
Ph
Me
N
Boc
*6aj*
$^1$H NMR
(400 MHz, CDCl$_3$)
8.09 8.07 7.35 7.34 7.26 7.24 7.22 7.20 7.20 7.18 7.18 7.16 7.15 7.14 7.13 7.12 6.10
4.97 4.88
3.74 3.72 3.70 3.65 3.63 3.61 3.59 3.43 3.41 3.39 3.37 3.35
1.66 1.65
1.00 1.03 2.22 5.03 1.02 1.05 1.04 1.05 1.09 1.04 8.93 3.21
9.0 8.5 8.0 7.5 7.0 6.5 6.0 5.5 5.0 4.5 4.0 3.5 3.0 2.5 2.0 1.5 1.0 0.5 0.0 -0
f1 (ppm)

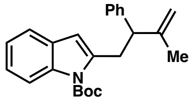


***6aj***
$^{13}$C NMR
(100 MHz, $CDCl_3$)

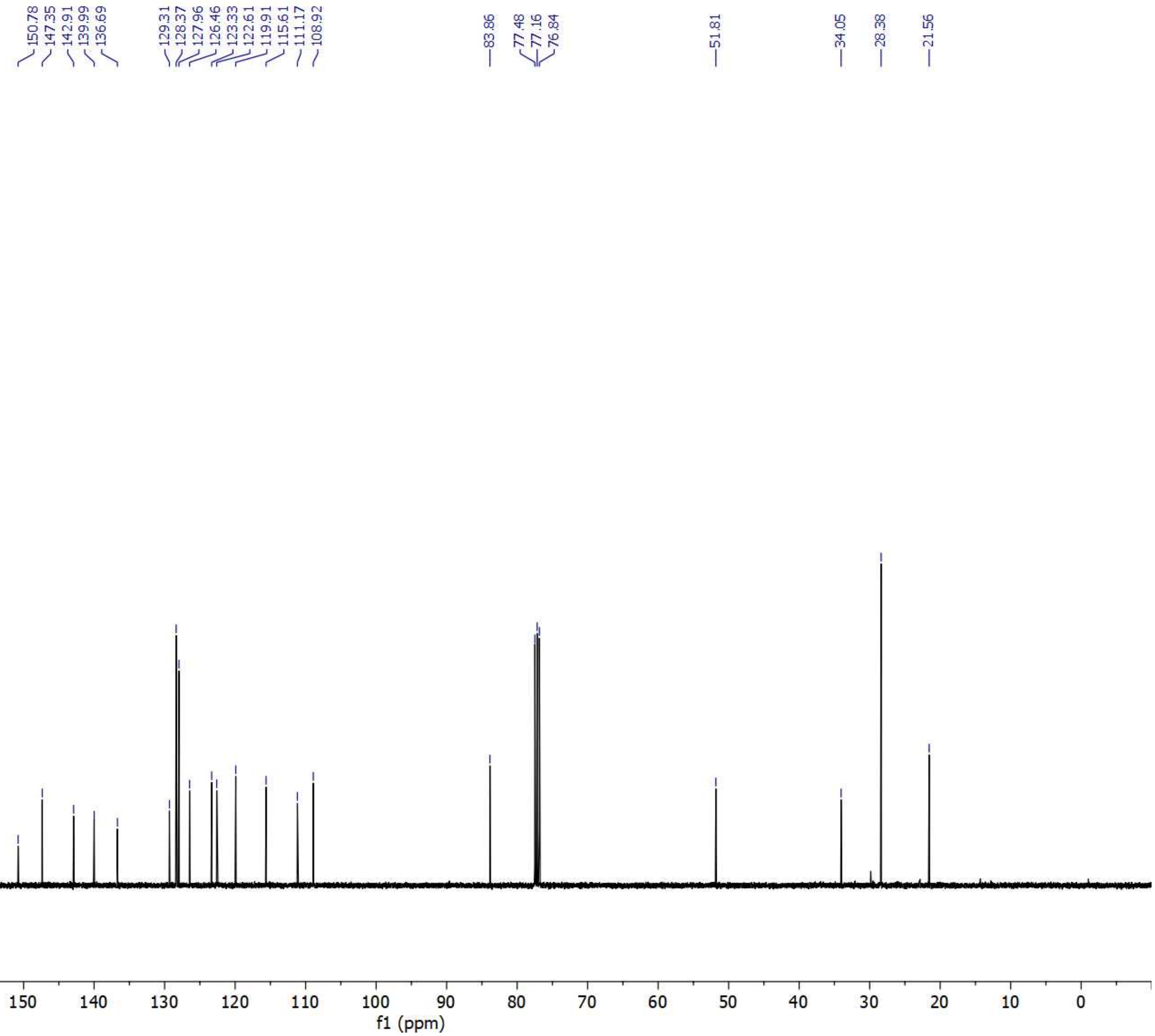

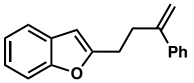


***12ab***
$^1$H NMR
(400 MHz, $CDCl_3$)

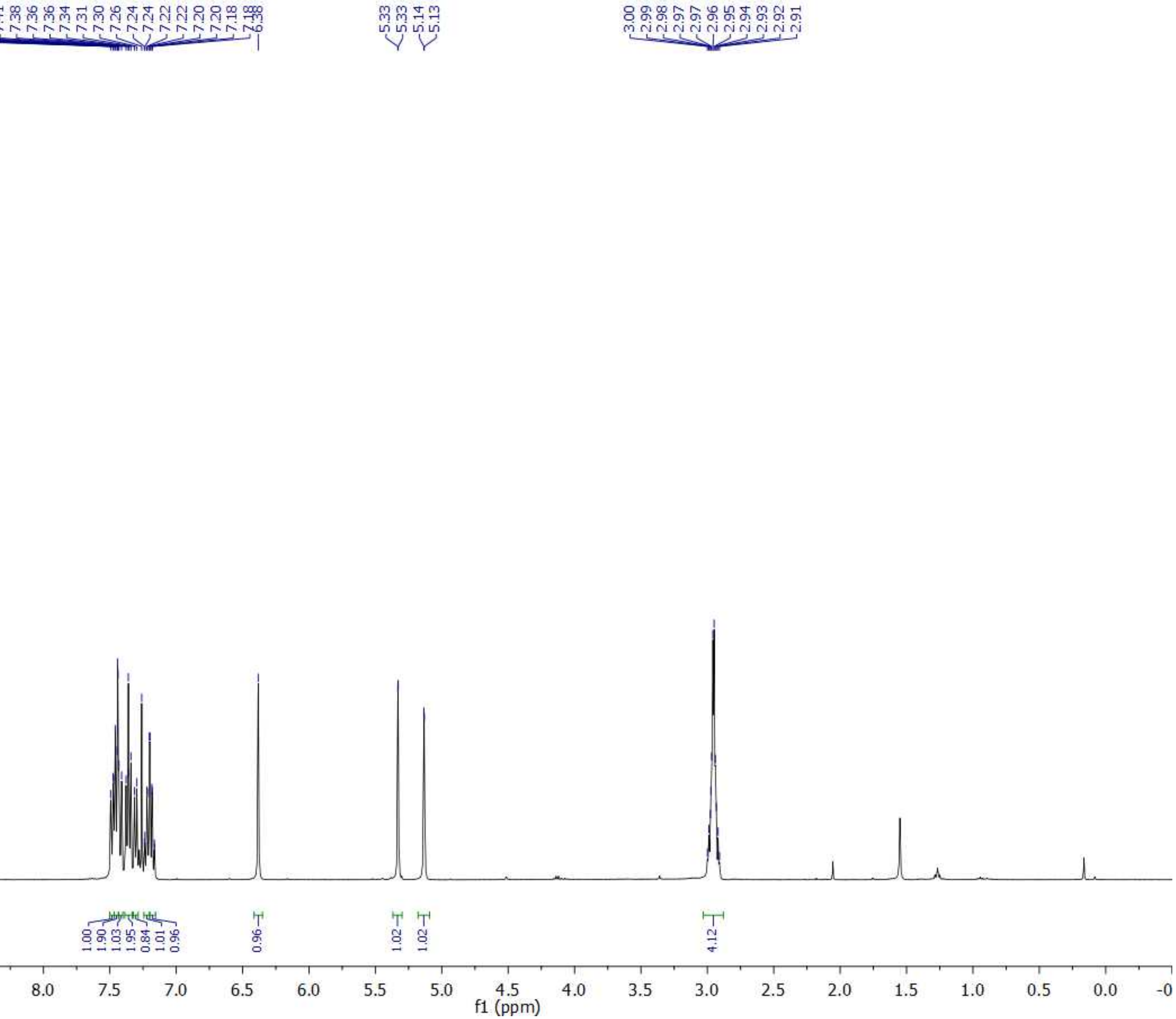

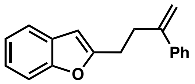

**12ab**
$^{13}C$ NMR
(100 MHz, $CDCl_3$)

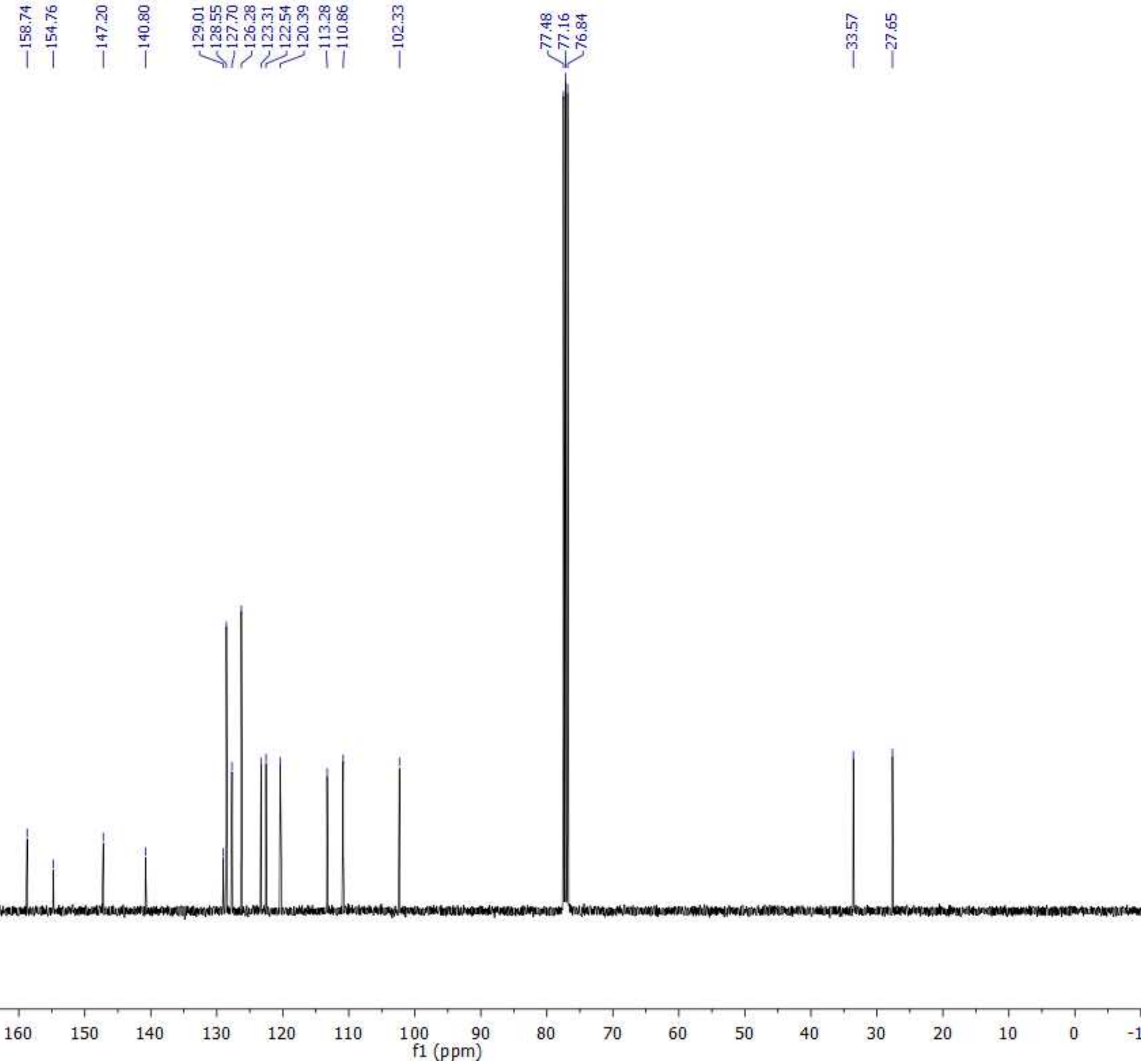

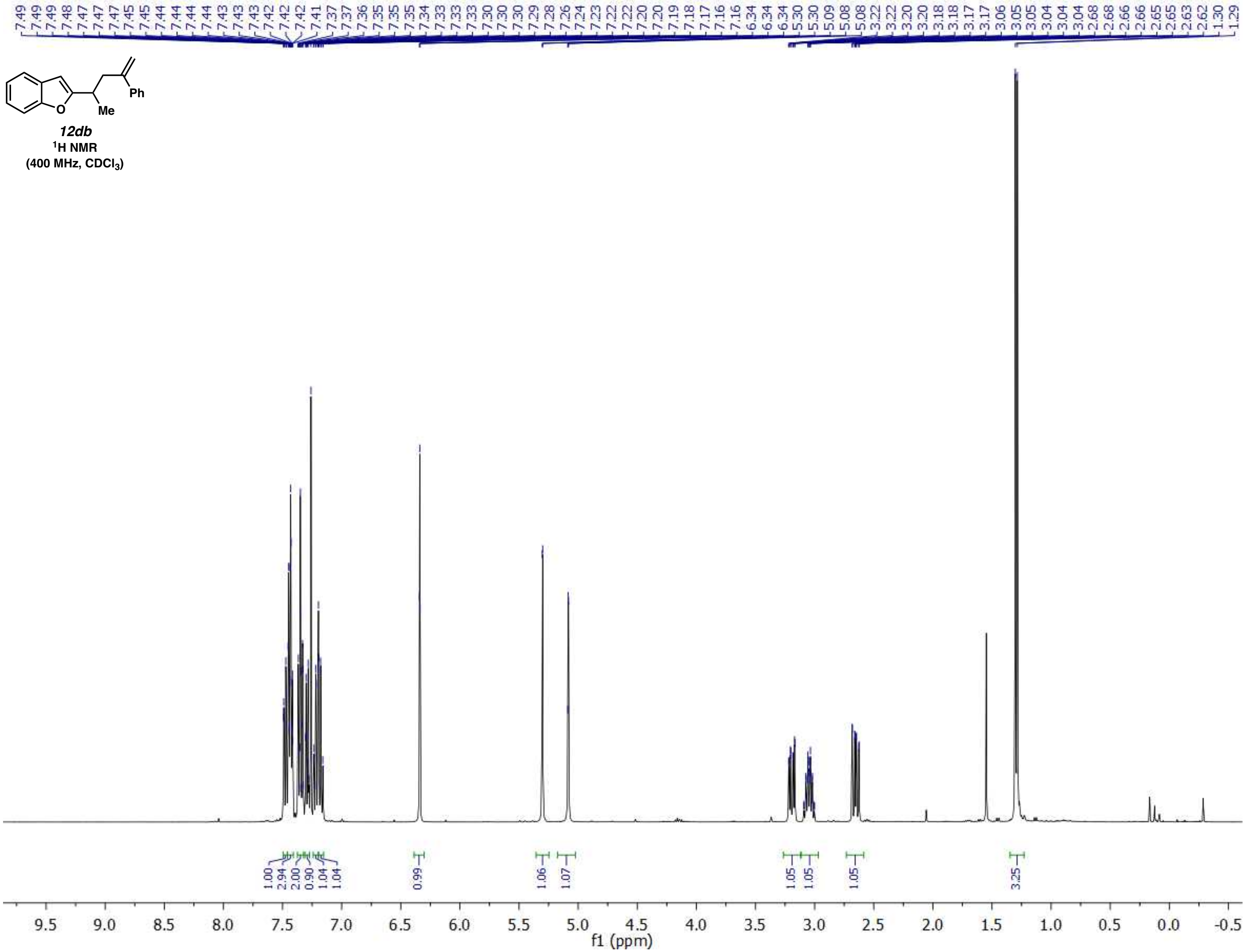
12db
1H NMR
(400 MHz, CDCl3)
f1 (ppm)

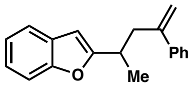

***12db***
$^{13}C$ NMR
(100 MHz, $CDCl_3$)

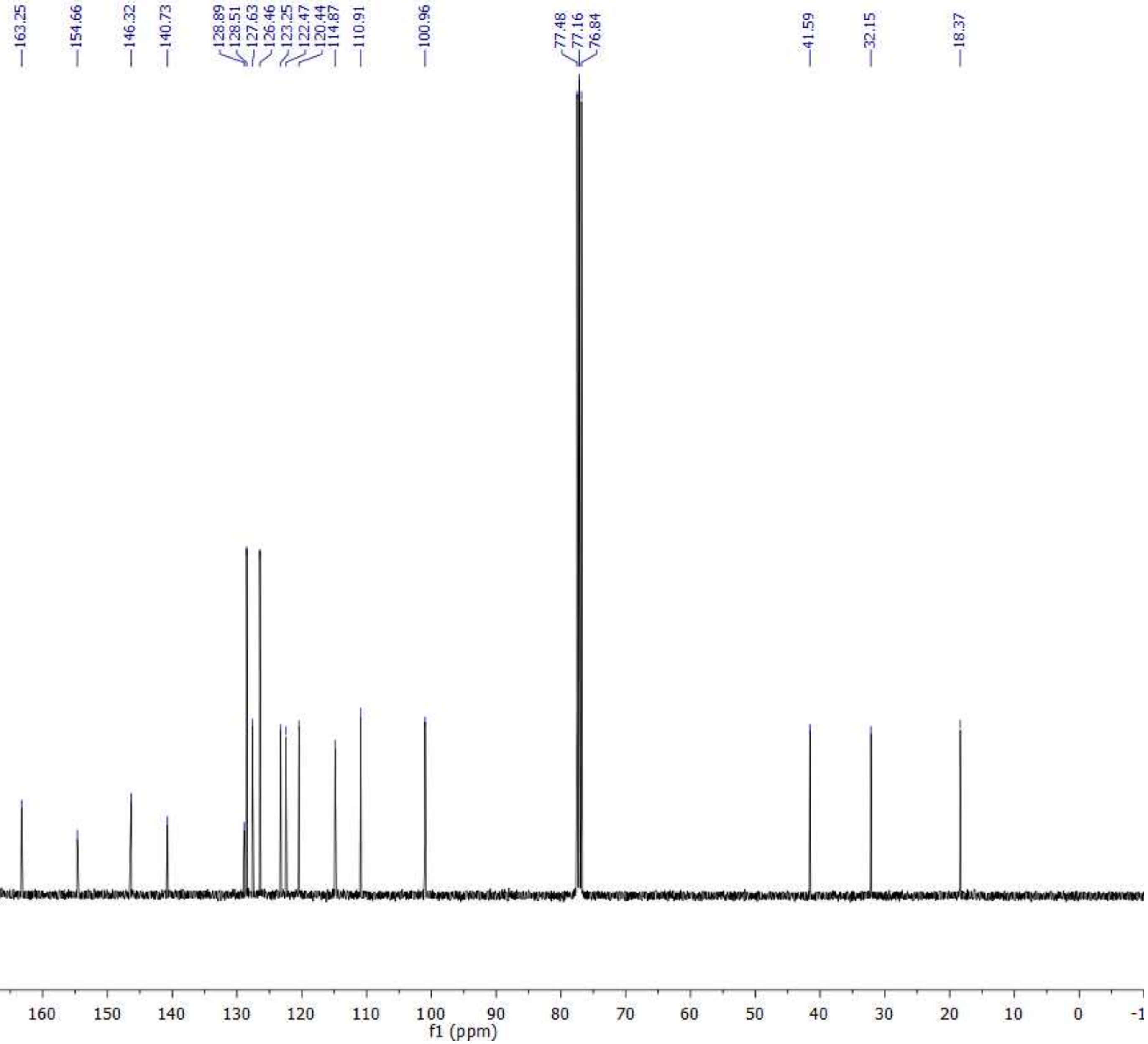

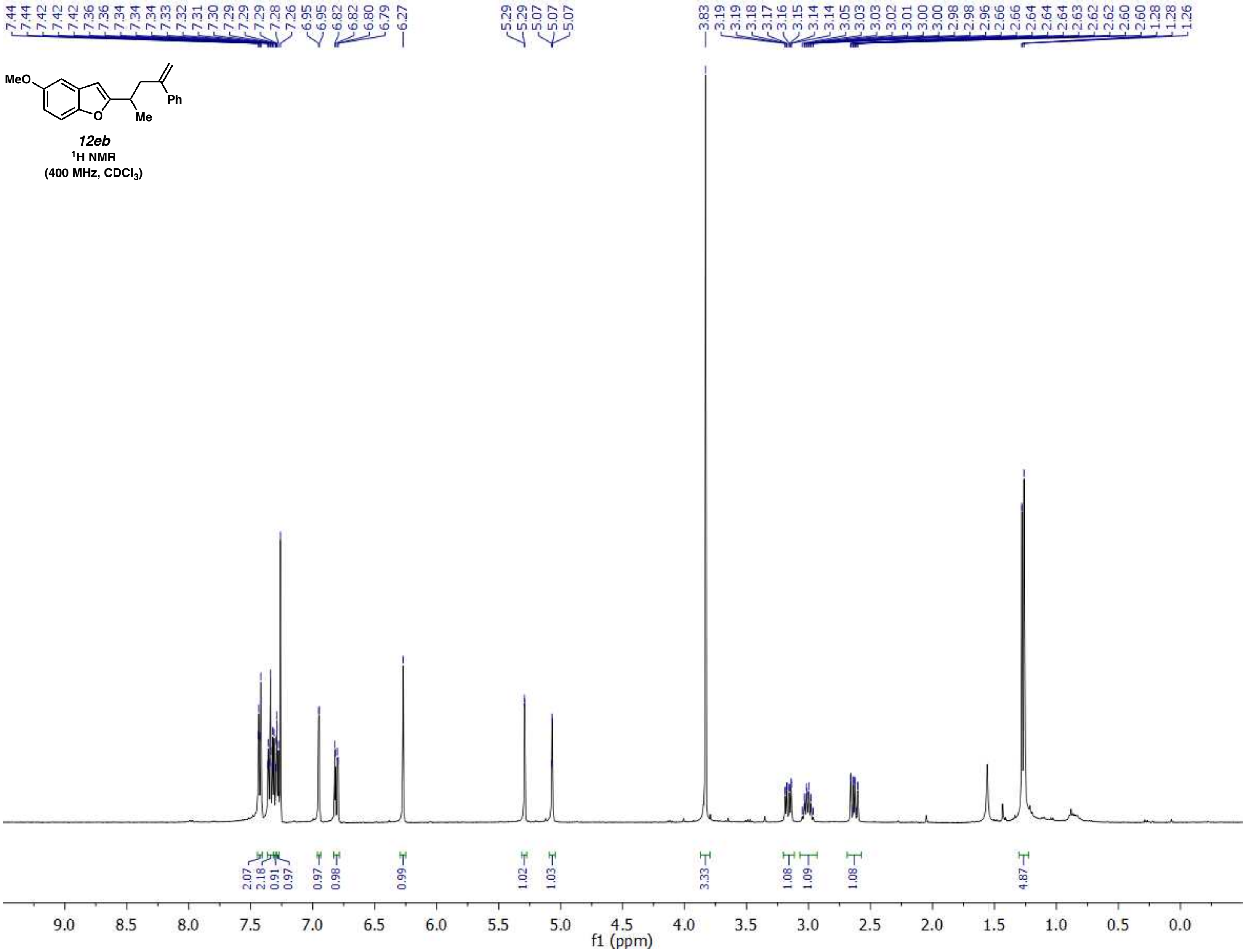

MeO
O
Me
Ph
12eb
$^1$H NMR
(400 MHz, $CDCl_3$)
f1 (ppm)

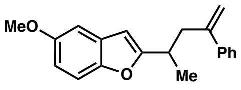

***12eb***
$^{13}C$ NMR
(100 MHz, $CDCl_3$)

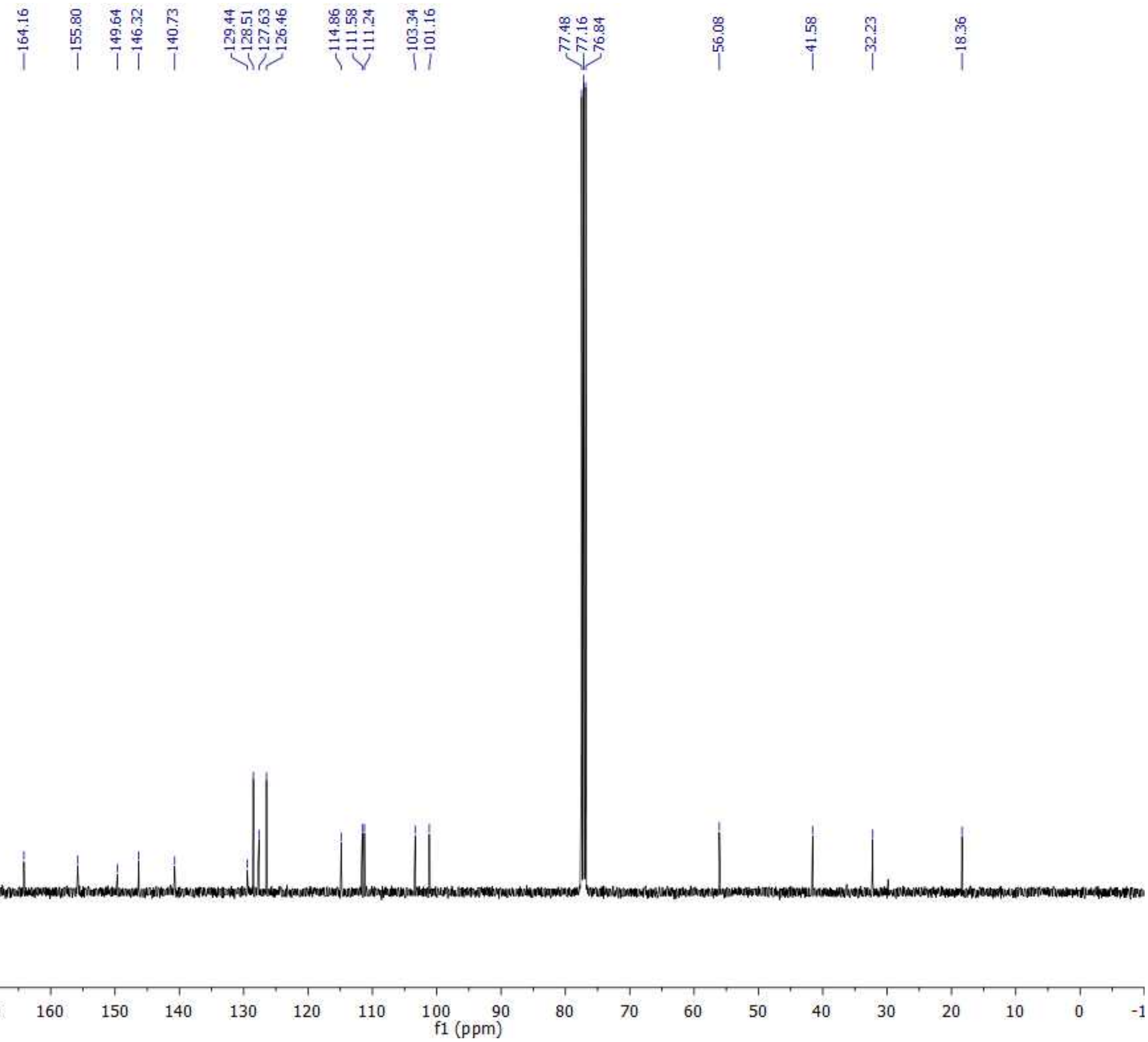

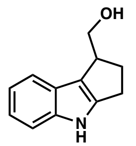

*11ae*
$^1$H NMR
(400 MHz, $CDCl_3$)

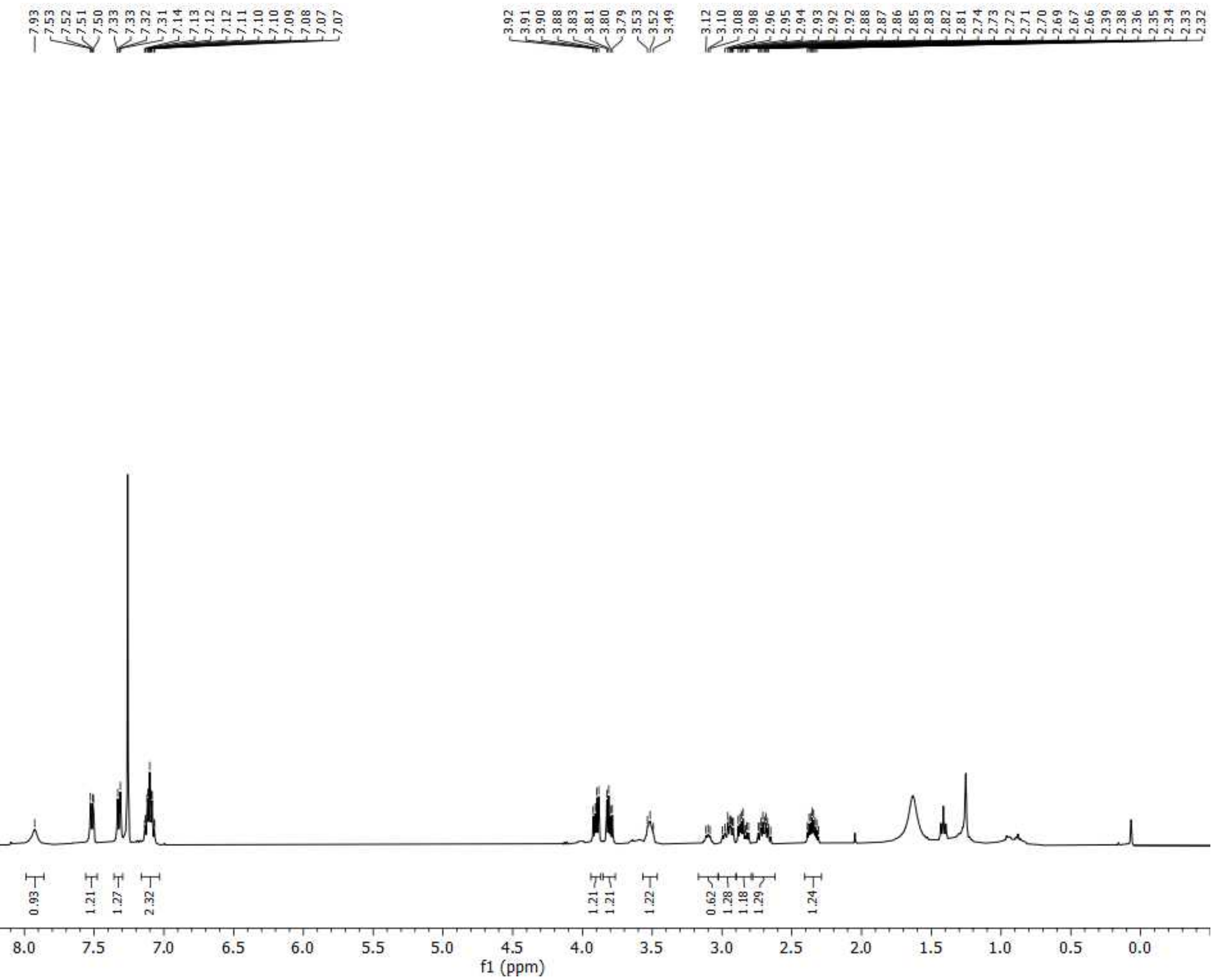

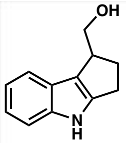

*11ae*
$^{13}C$ NMR
(100 MHz, $C_6D_6$)

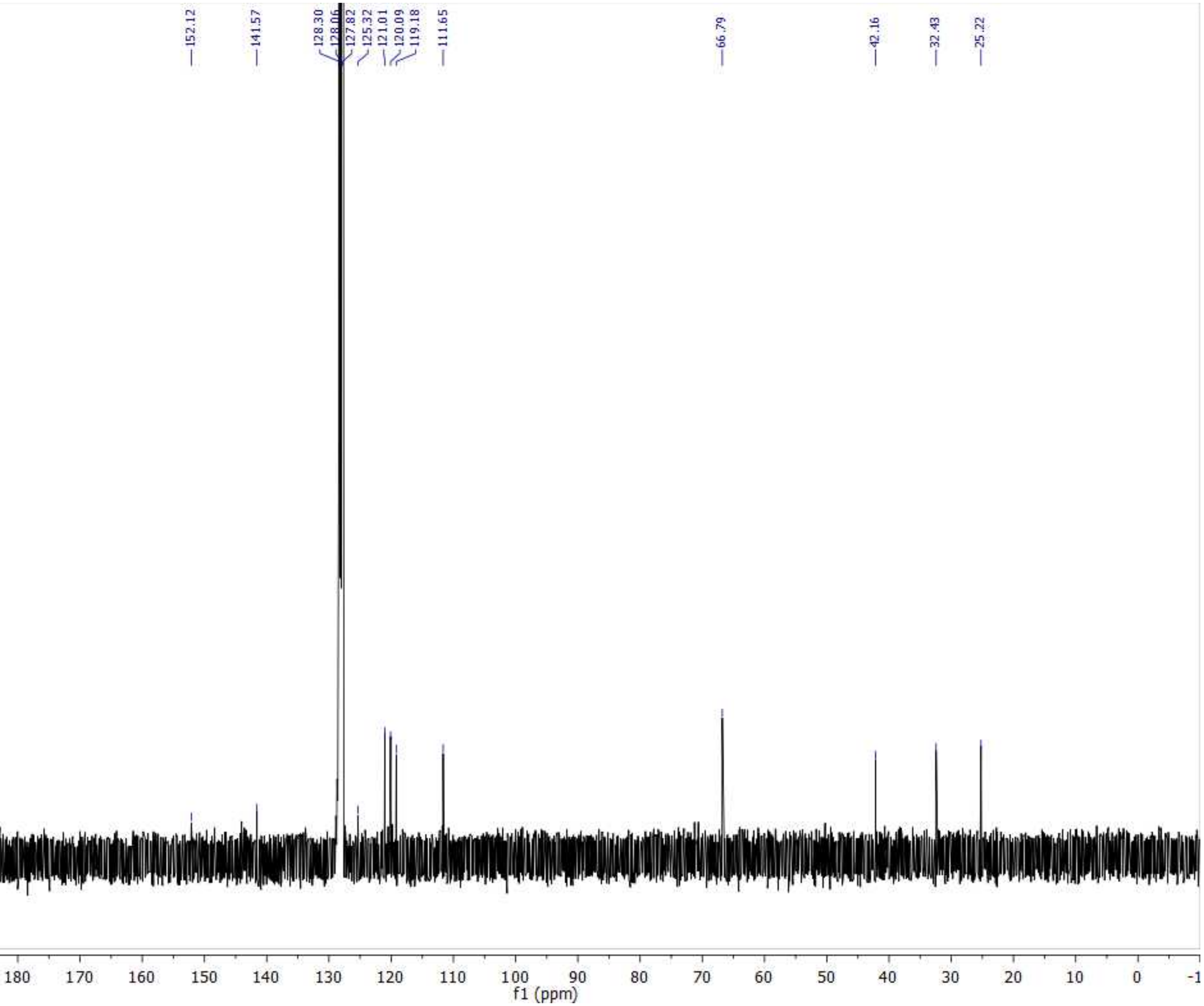

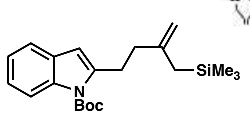


***13af***
$^1$H NMR
(400 MHz, CDCl$_3$)

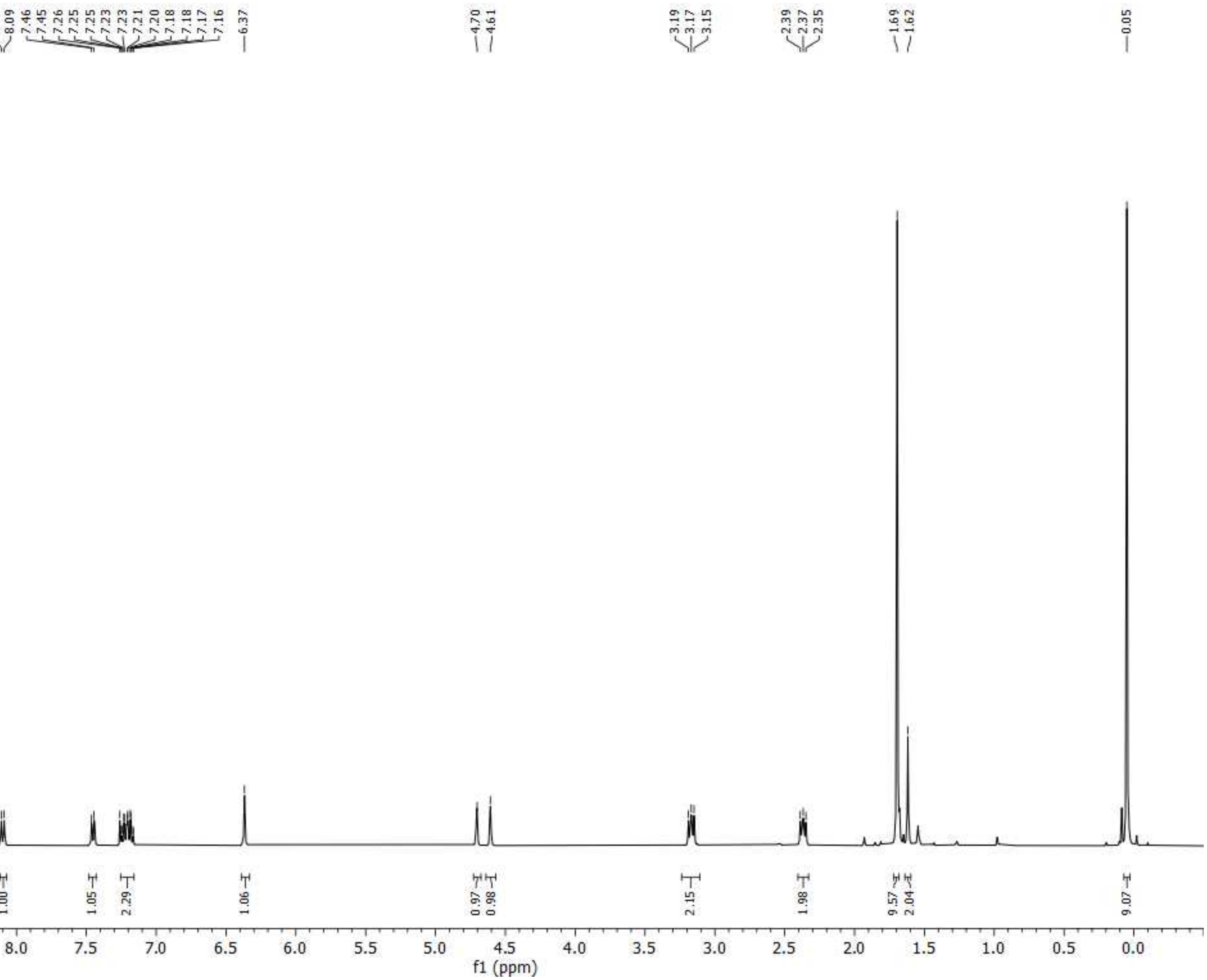

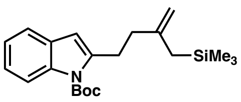

***13af***
$^{13}C$ NMR
(100 MHz, $CDCl_3$)

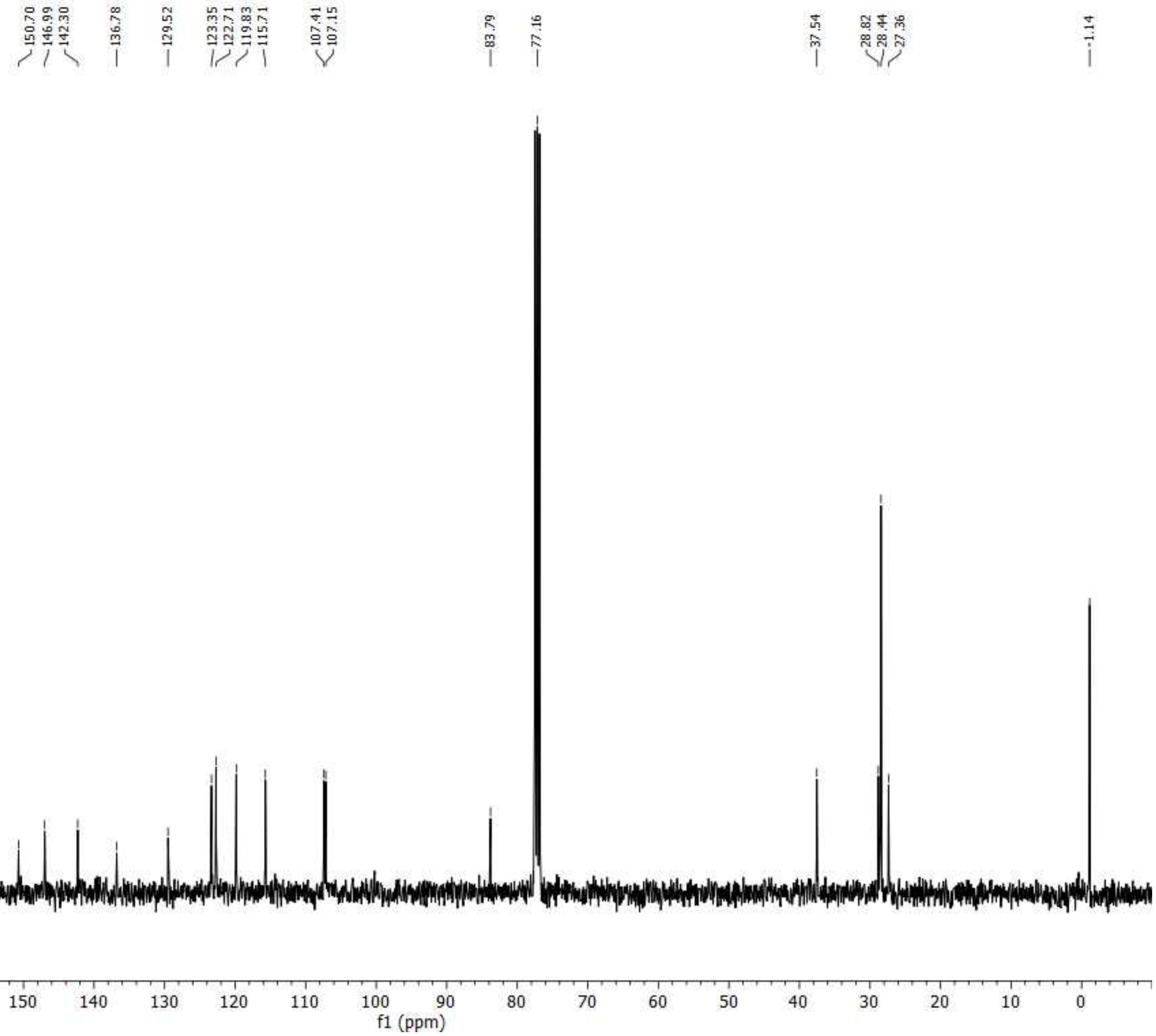

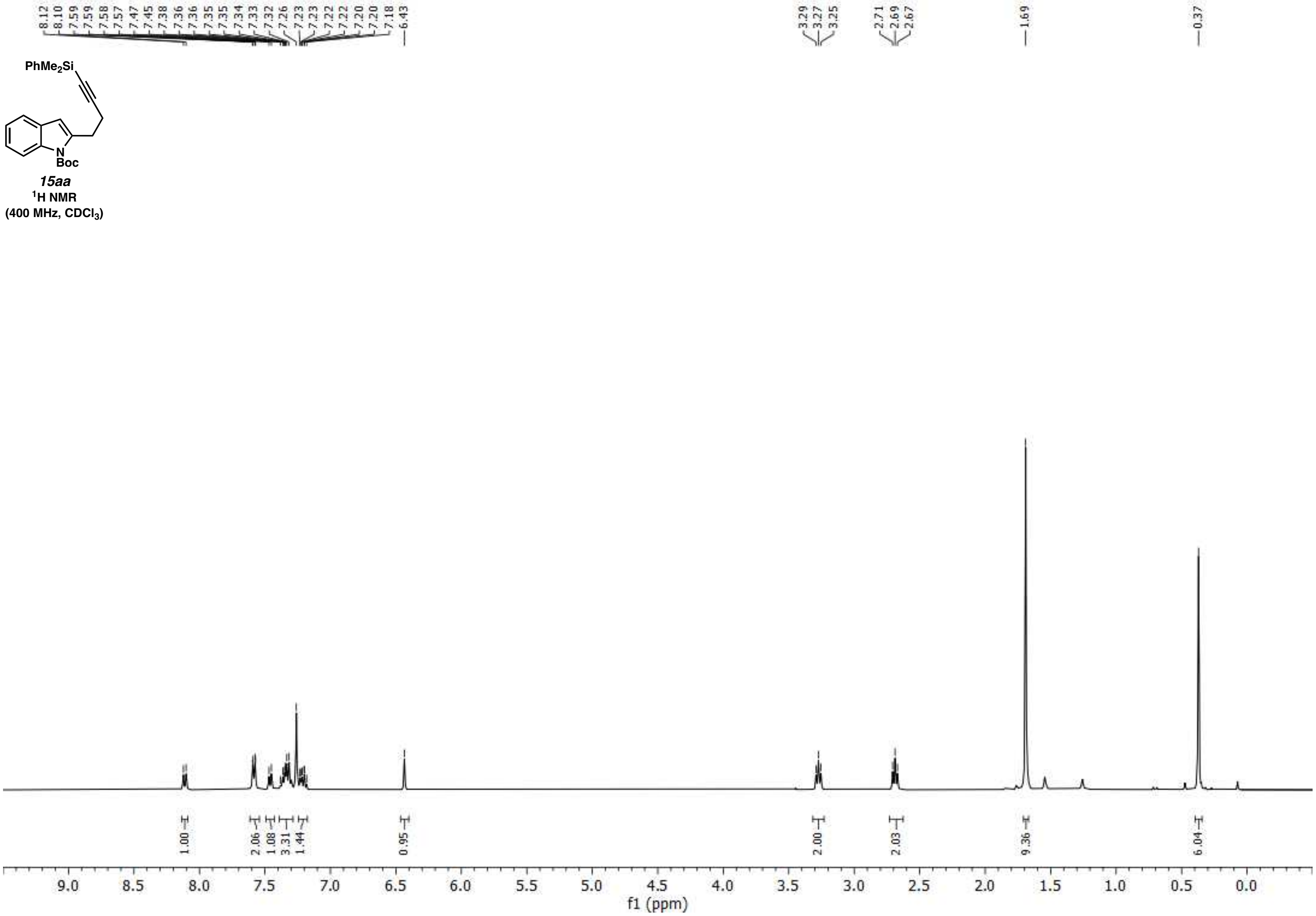


***15aa***
**$^1$H NMR**
**(400 MHz, $CDCl_3$)**

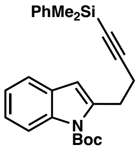


***15aa***
**$^{13}$C NMR**
**(100 MHz, $CDCl_3$)**

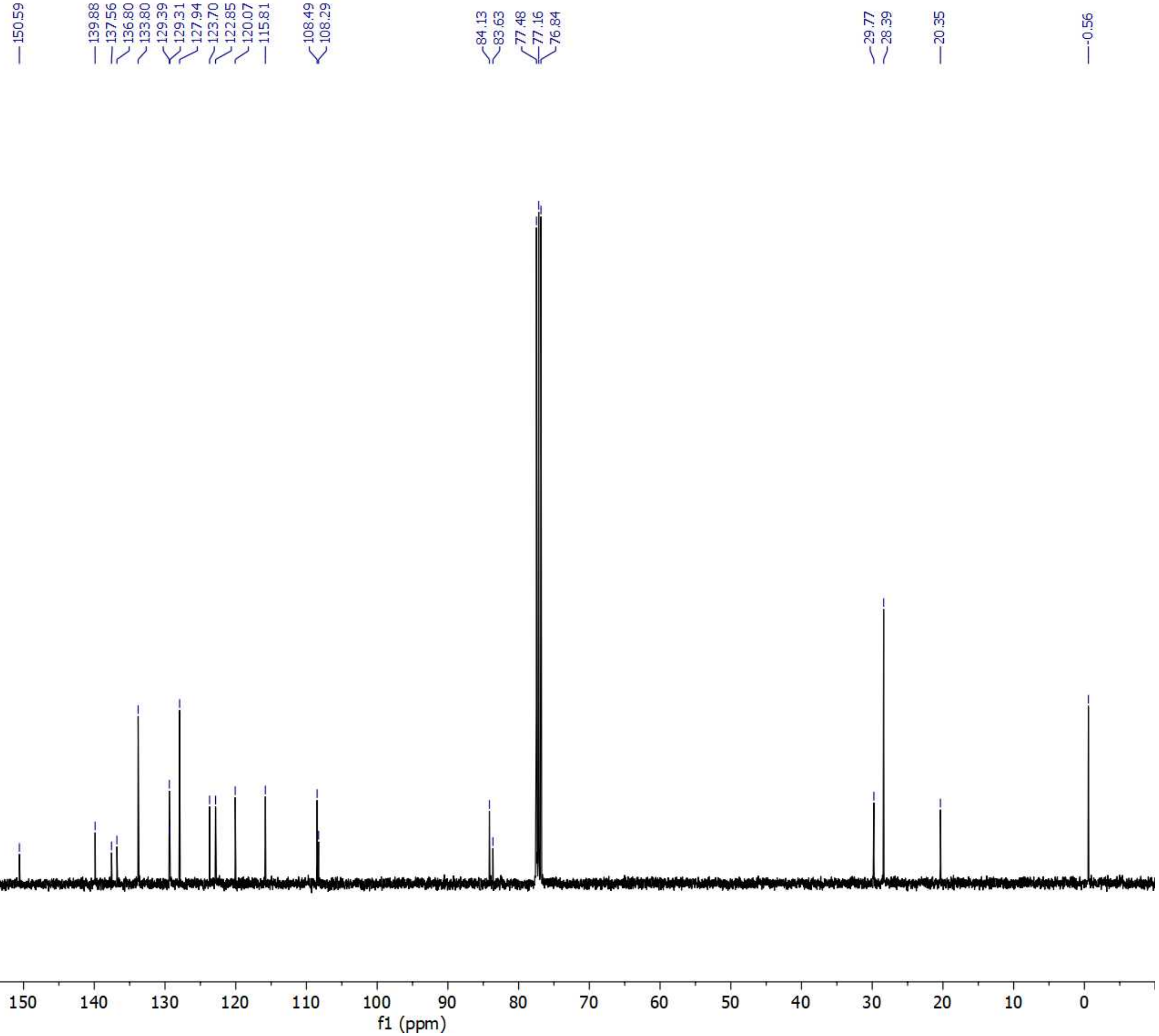

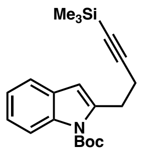

***15ab***
**$^1$H NMR**
**(400 MHz, $CDCl_3$)**

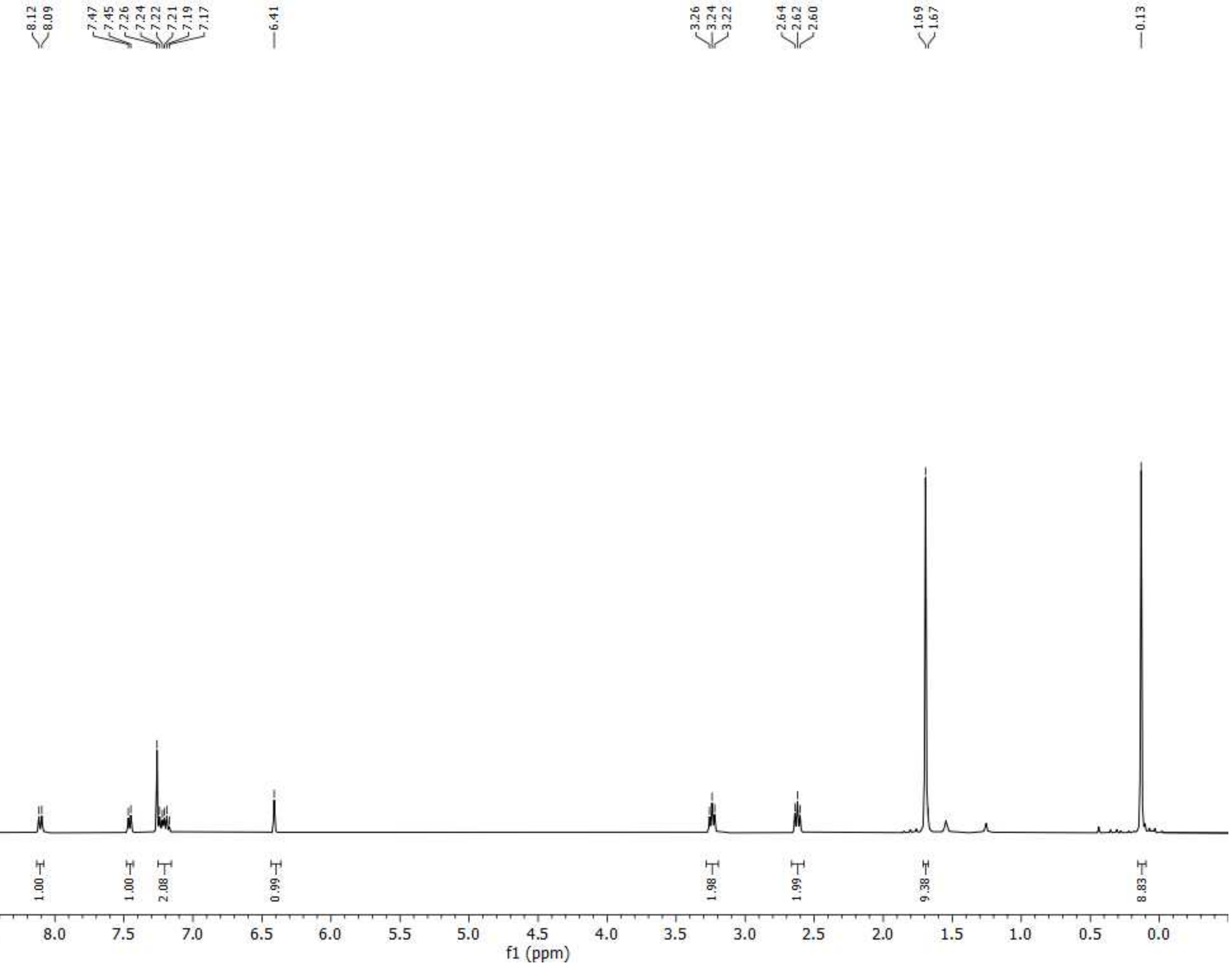

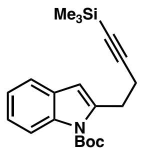

**15ab**
$^{13}$C NMR
(100 MHz, $CDCl_3$)

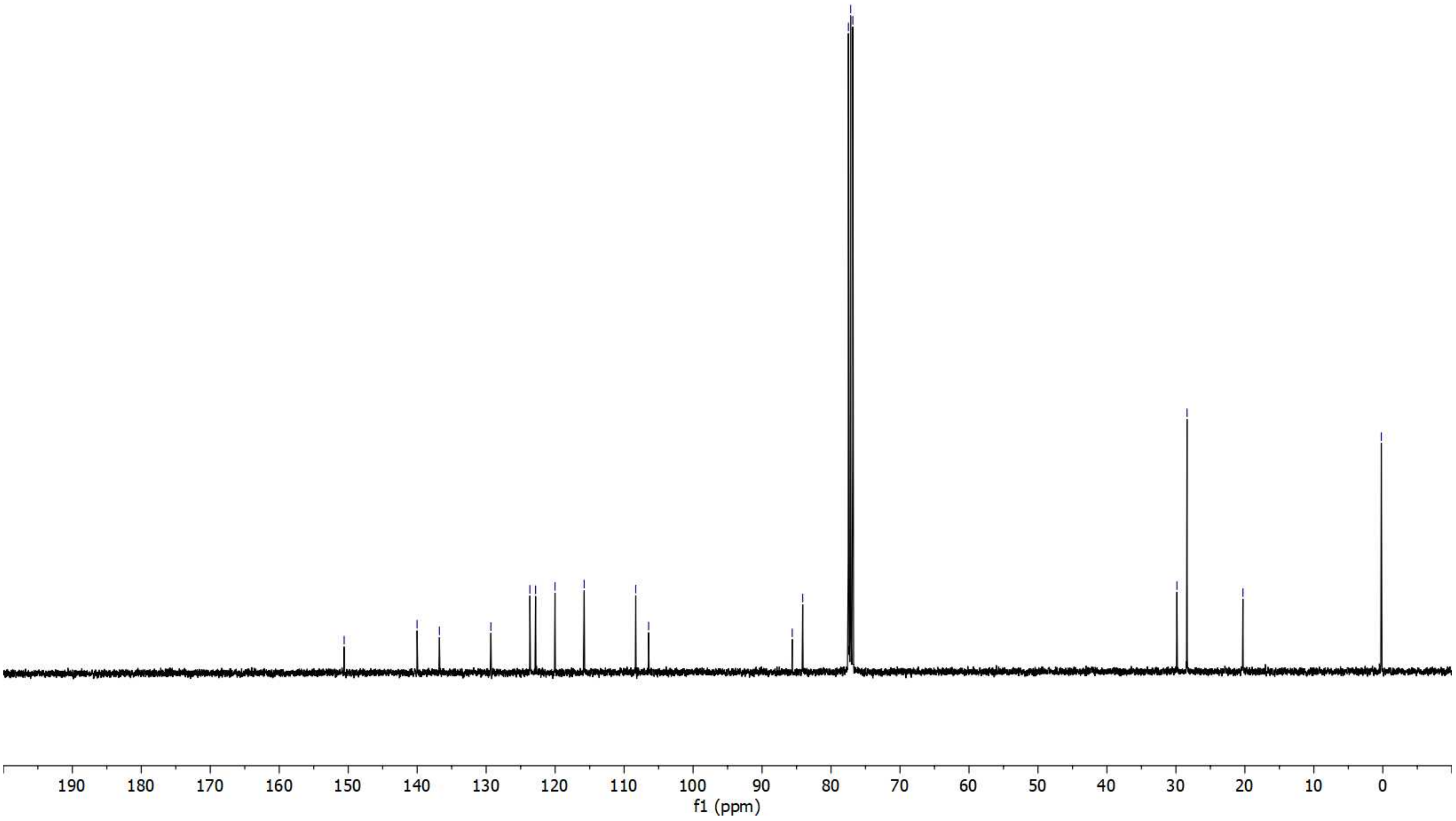

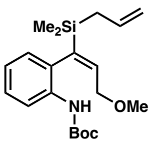

***16ak***
$^1$H NMR
(400 MHz, $CDCl_3$)

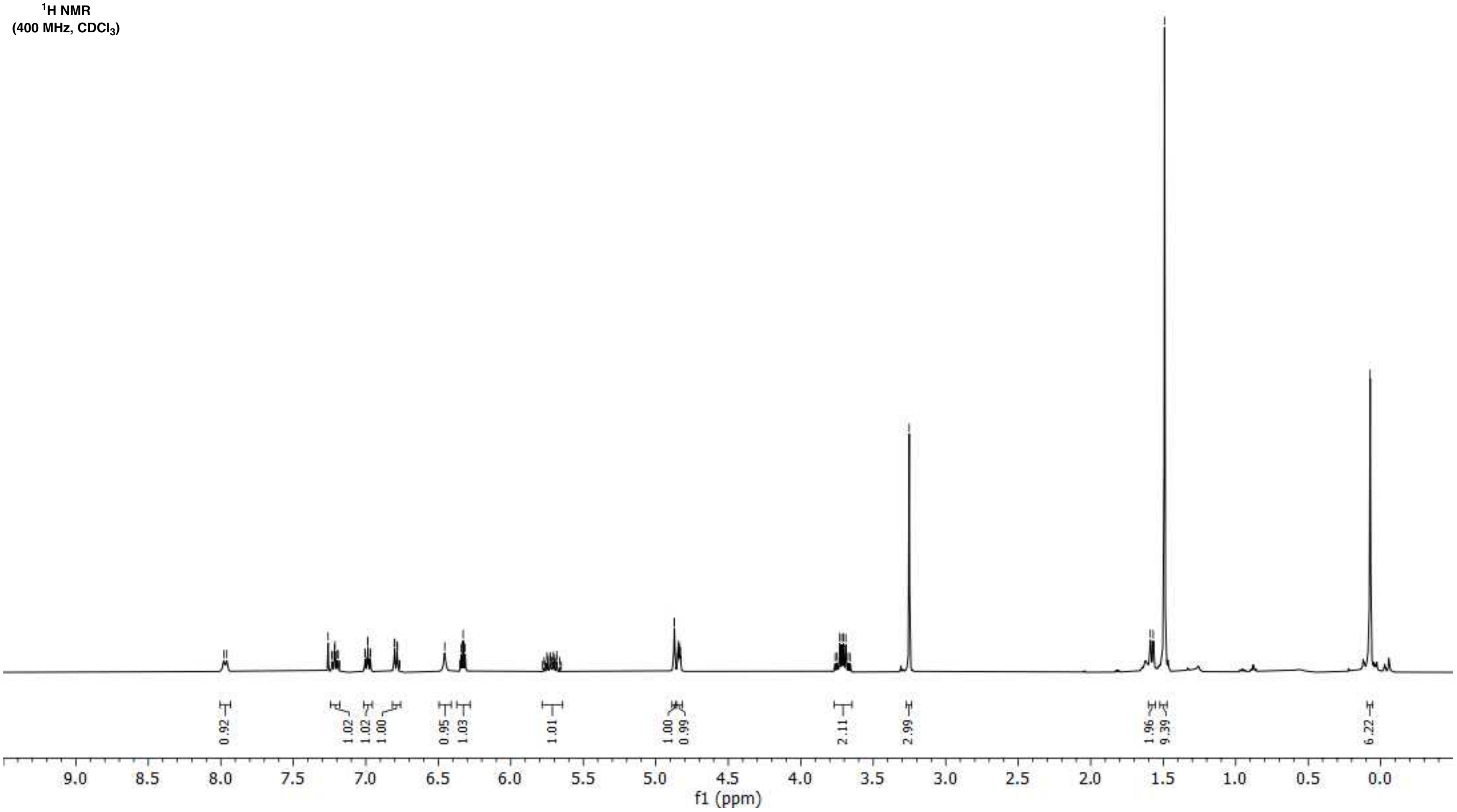

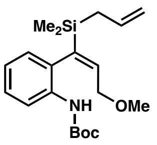

*16ak*
$^{13}C$ NMR
(100 MHz, $CDCl_3$)

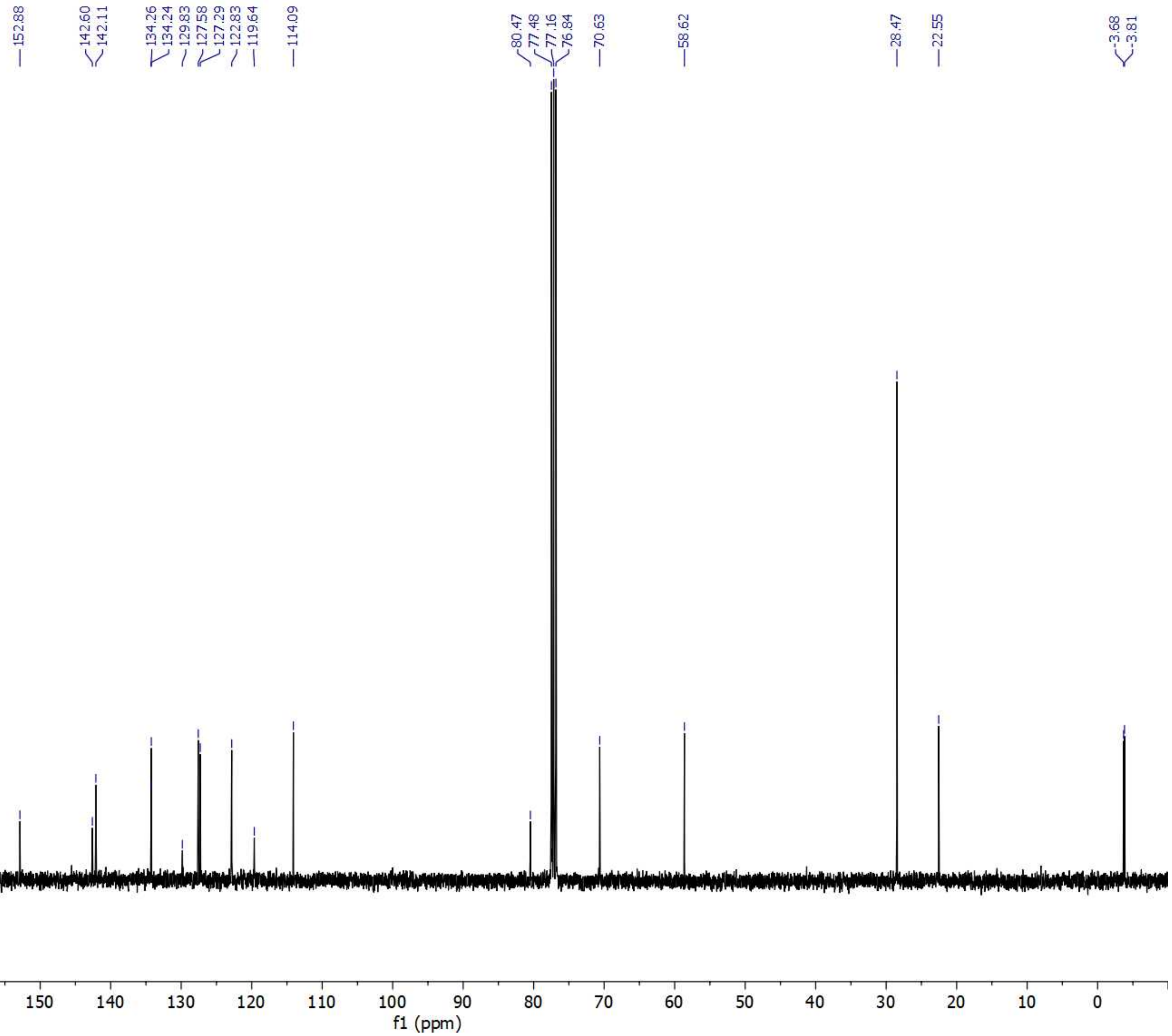

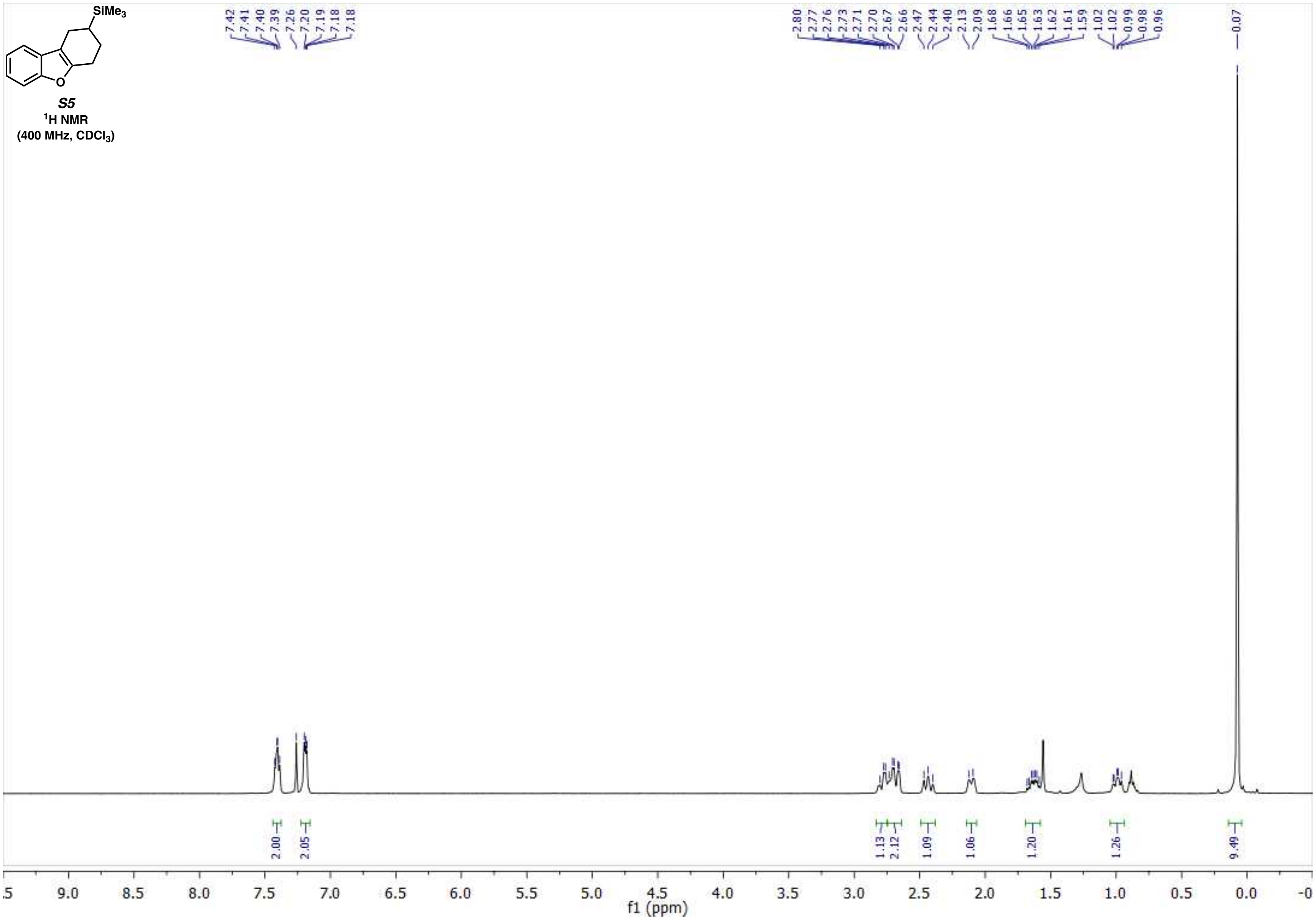

SiMe3
O
S5
1H NMR
(400 MHz, CDCl3)
7.42 7.41 7.40 7.39 7.26 7.20 7.19 7.18 7.18
2.80 2.77 2.76 2.73 2.71 2.70 2.67 2.66 2.47 2.44 2.40 2.13 2.09 1.68 1.66 1.65 1.63 1.62 1.61 1.59 1.02 1.02 0.99 0.98 0.96
0.07
2.00 2.05 1.13 2.12 1.09 1.06 1.20 1.26 9.49
9.0 8.5 8.0 7.5 7.0 6.5 6.0 5.5 5.0 4.5 4.0 3.5 3.0 2.5 2.0 1.5 1.0 0.5 0.0
f1 (ppm)

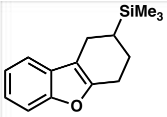

***S5***
$^{13}C$ NMR
(100 MHz, $CDCl_3$)

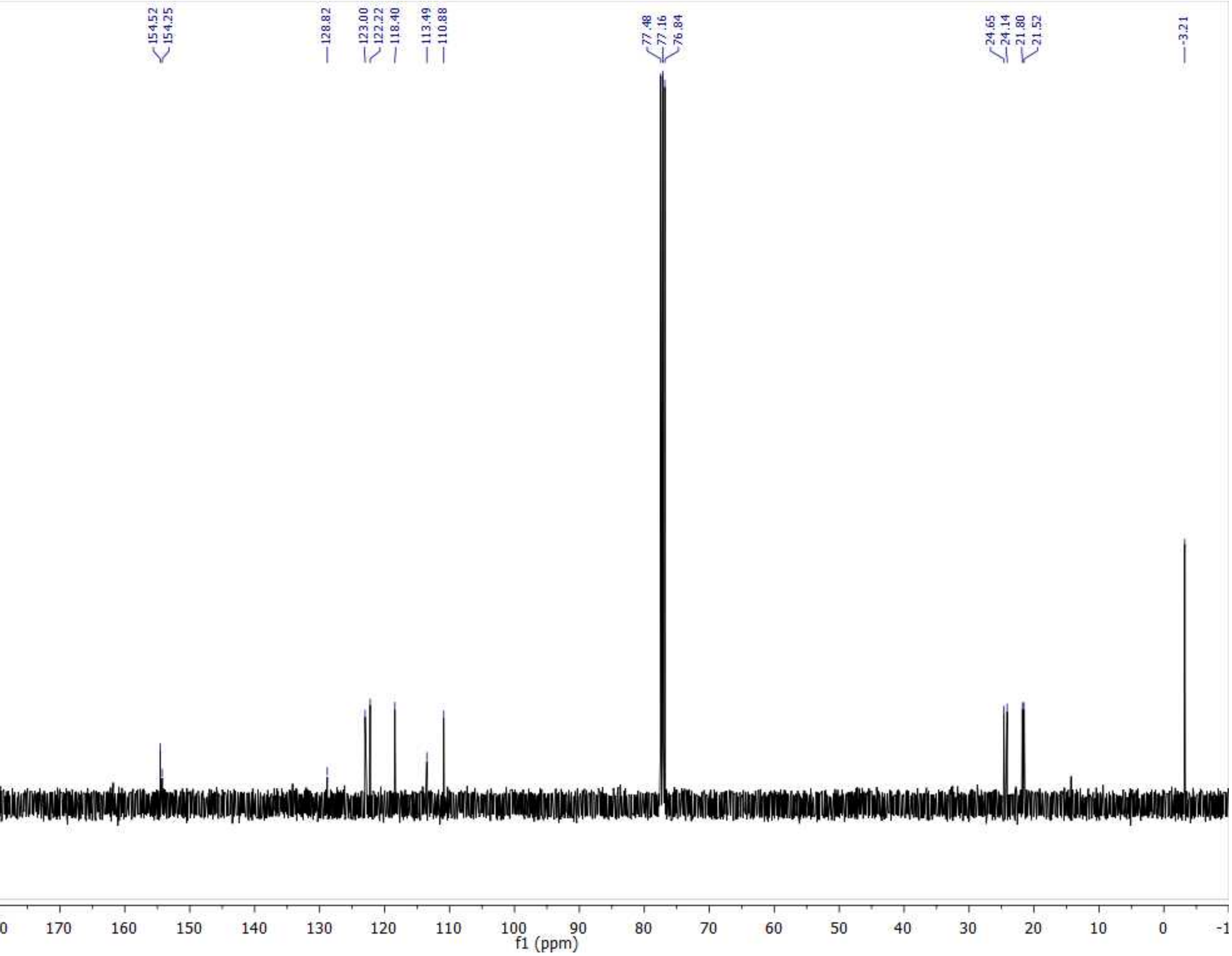

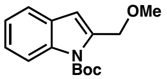


*8*
$^1$H NMR
(400 MHz, $CDCl_3$)

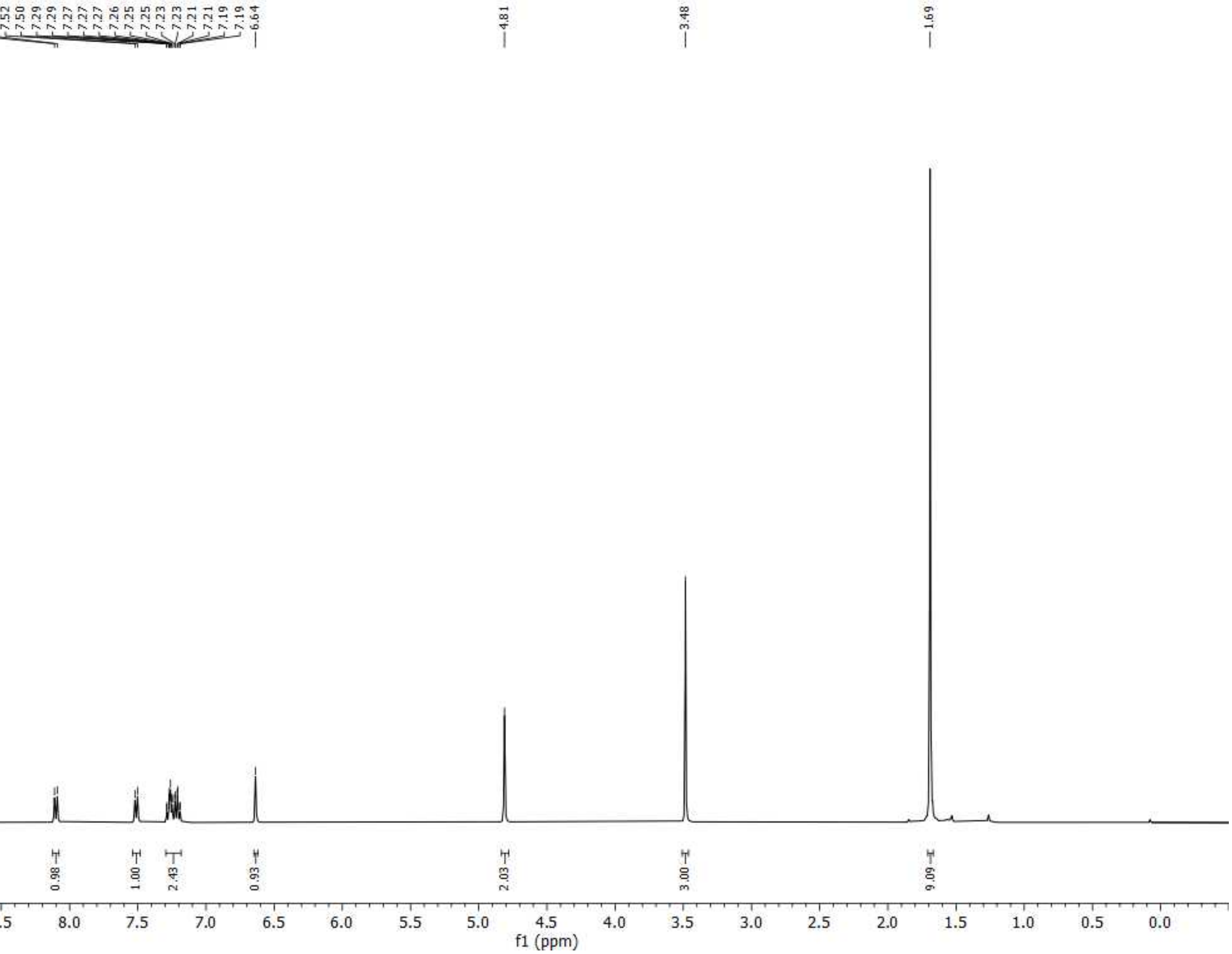

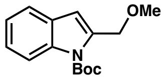


***8***
**$^{13}$C NMR**
**(100 MHz, $CDCl_3$)**

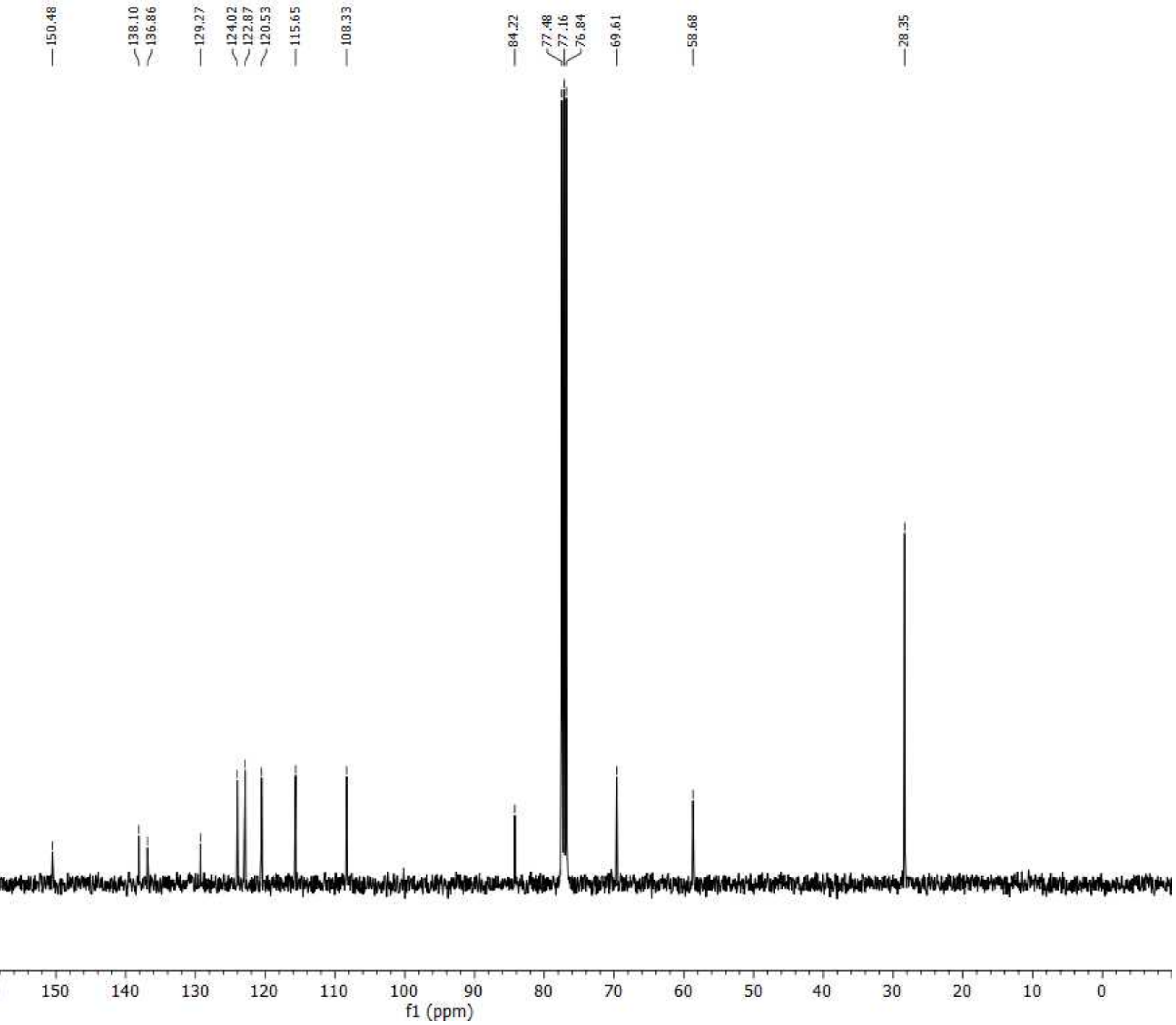

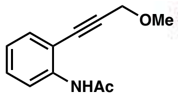


***1b***
$^1$H NMR
(400 MHz, $CDCl_3$)

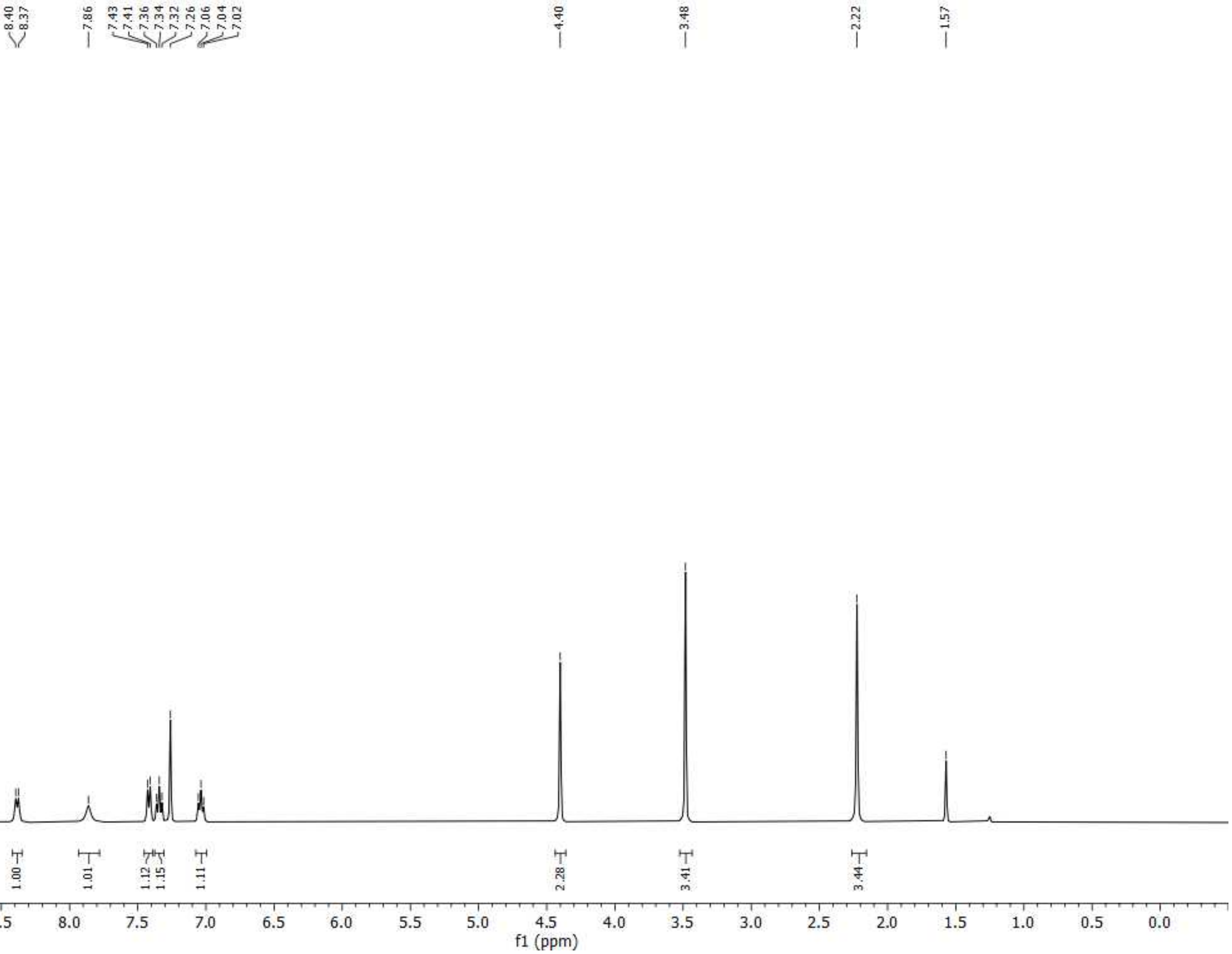

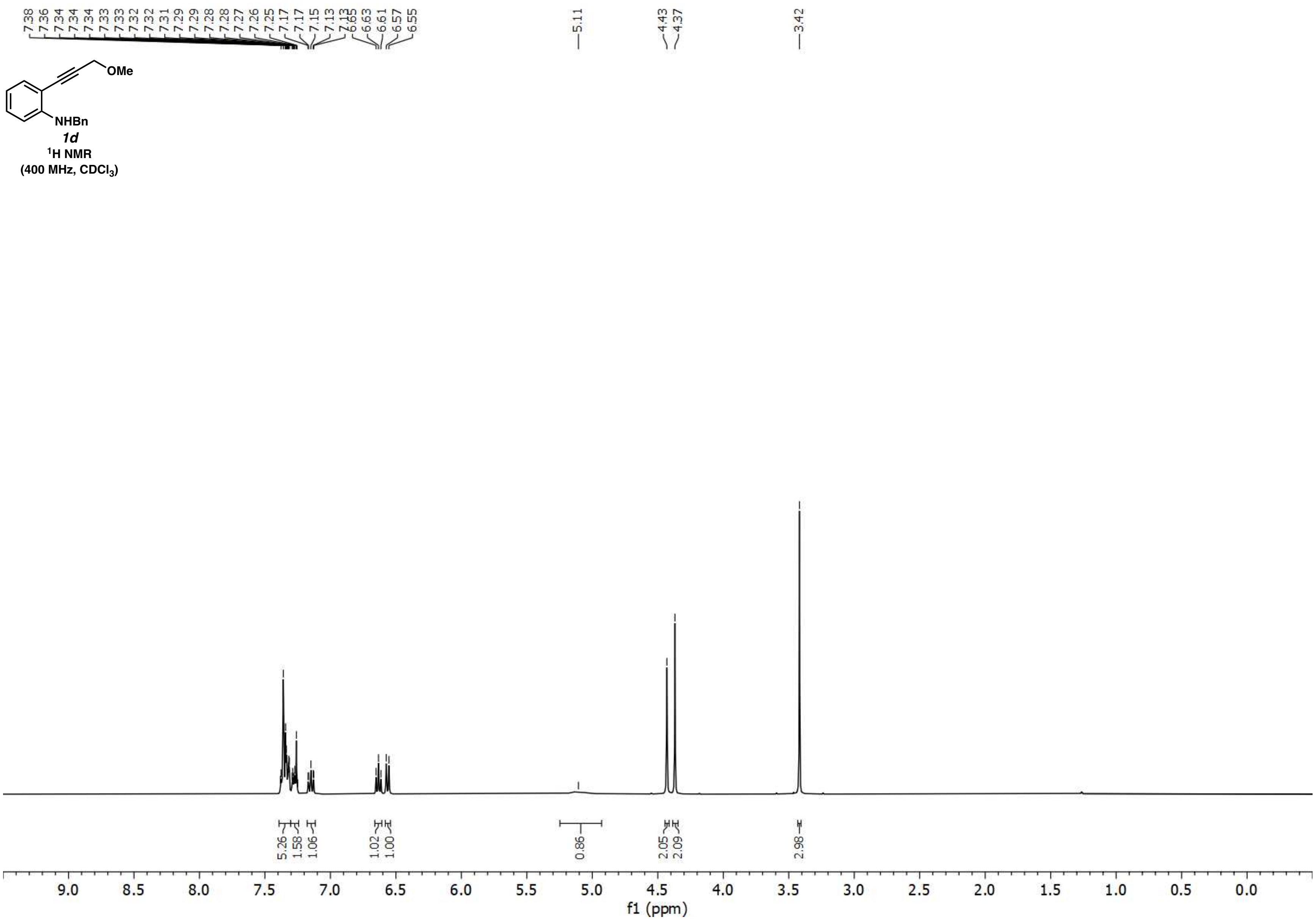
OMe
NHBn
1d
1H NMR
(400 MHz, CDCl3)
7.38
7.36
7.34
7.34
7.34
7.33
7.33
7.32
7.32
7.31
7.29
7.29
7.28
7.28
7.27
7.26
7.25
7.17
7.17
7.15
7.13
7.13
6.65
6.63
6.61
6.57
6.55
5.11
4.43
4.37
3.42
5.26
1.58
1.06
1.02
1.00
0.86
2.05
2.09
2.98
9.0
8.5
8.0
7.5
7.0
6.5
6.0
5.5
5.0
4.5
4.0
3.5
3.0
2.5
2.0
1.5
1.0
0.5
0.0
f1 (ppm)

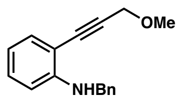


***1d***
$^{13}C$ NMR
(100 MHz, $CDCl_3$)

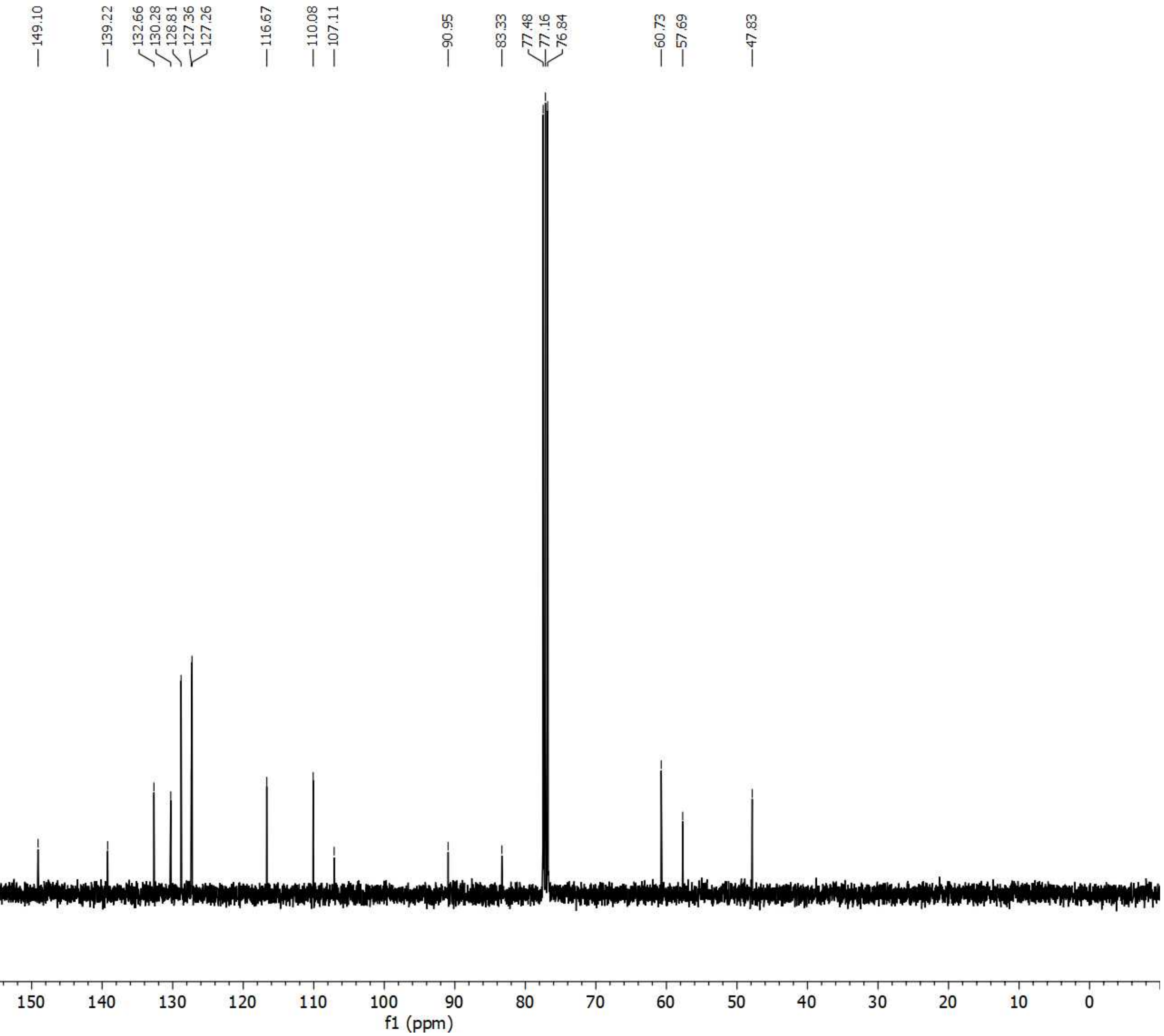

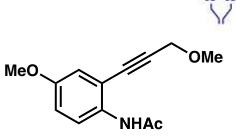


***1e***
$^1$H NMR
(400 MHz, $CDCl_3$)

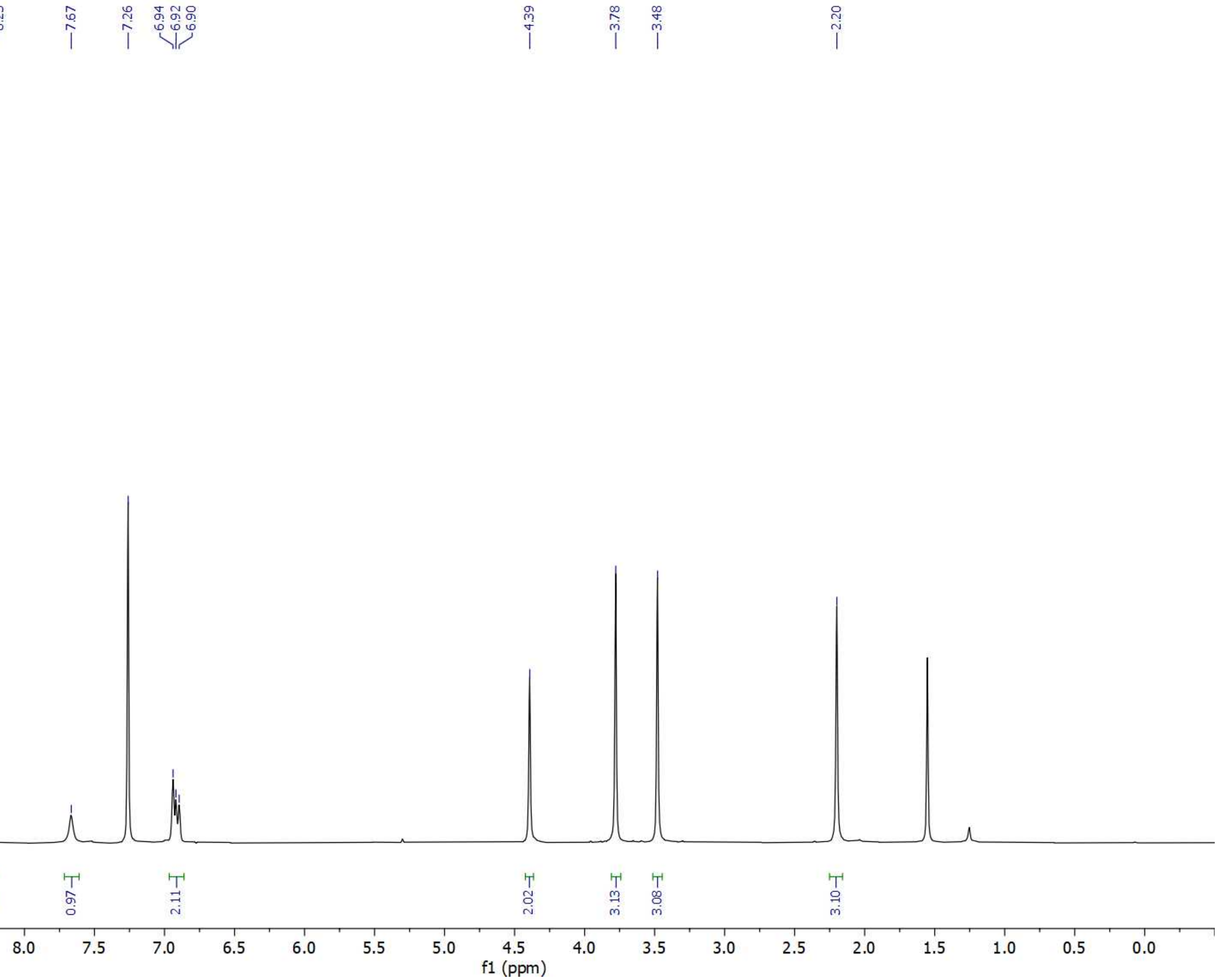

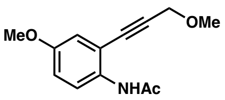


*1e*
$^{13}C$ NMR
(100 MHz, $CDCl_3$)

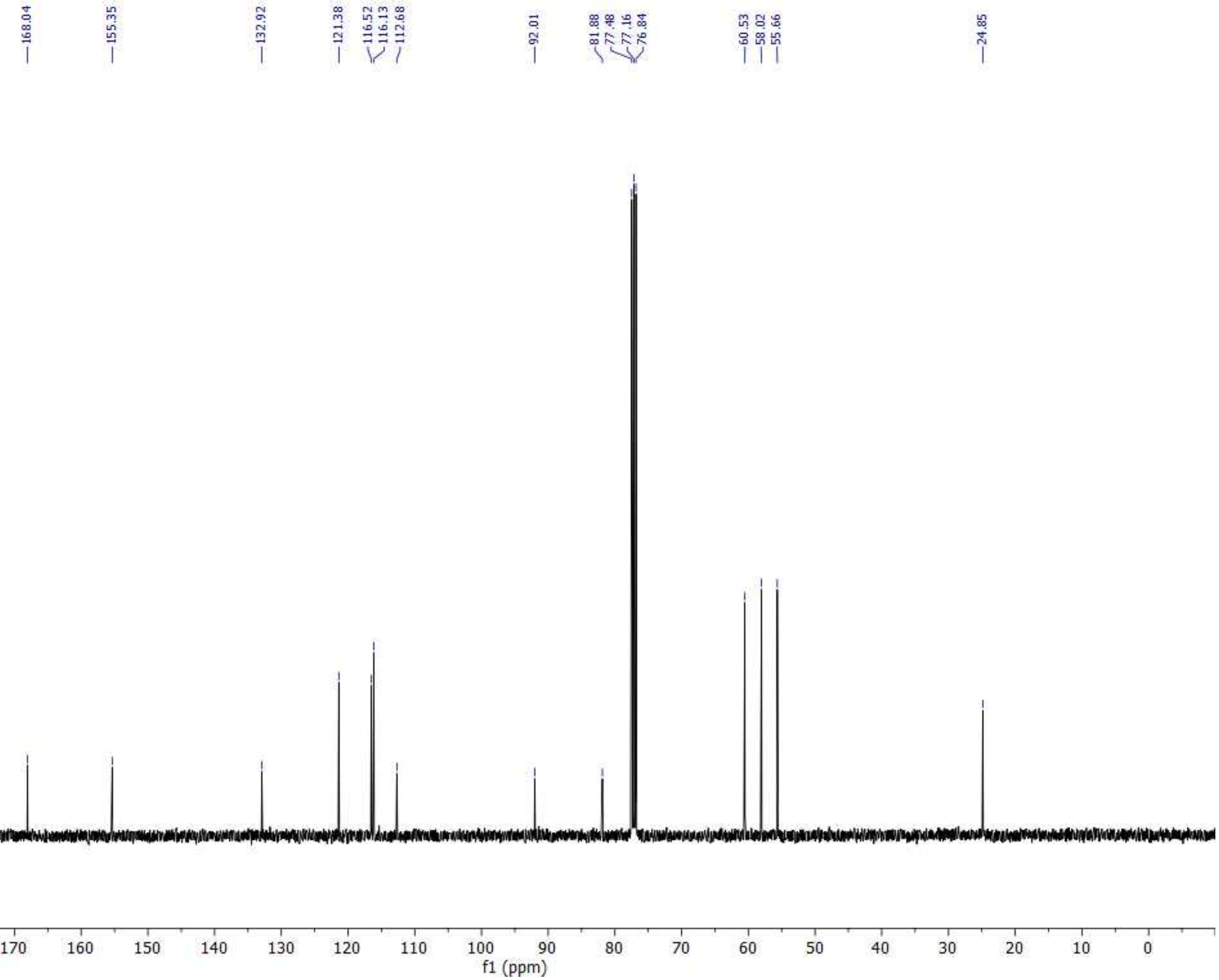

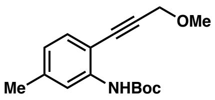


*1g*
$^1$H NMR
(400 MHz, $CDCl_3$)

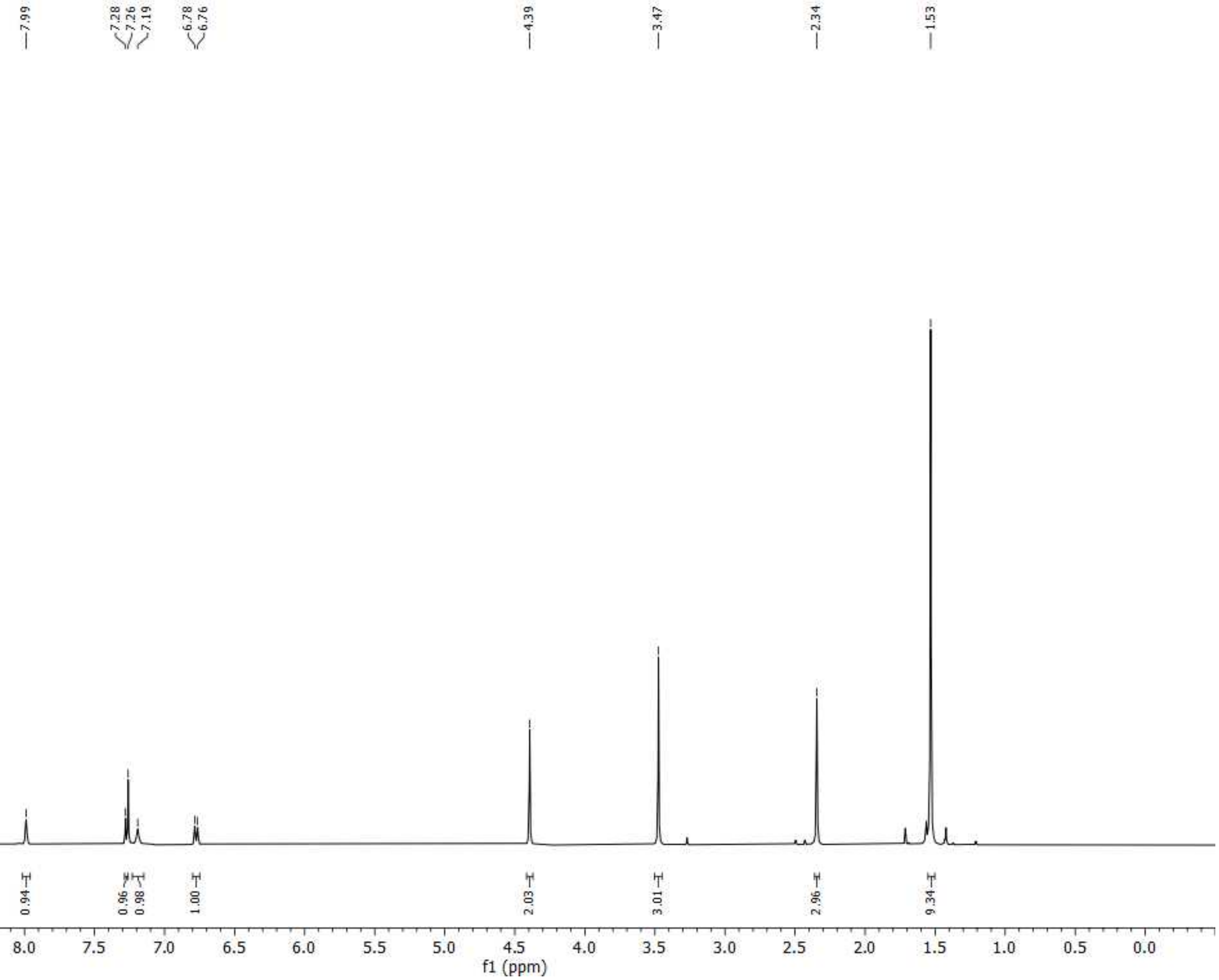

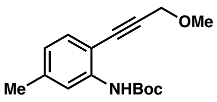

*1g*
$^{13}C$ NMR
(100 MHz, $CDCl_3$)

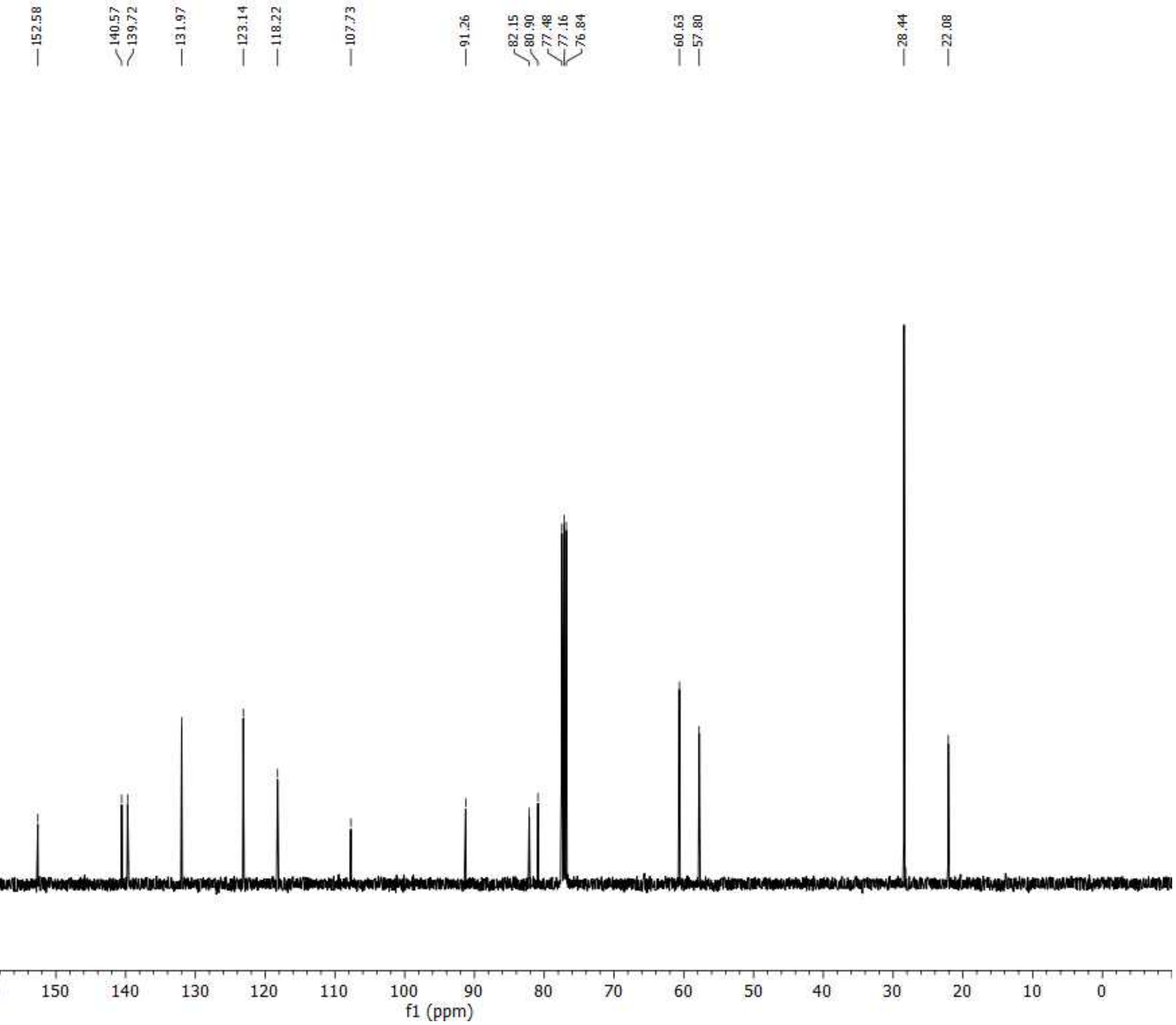

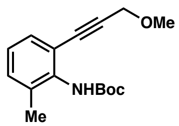


***1h***
$^1$H NMR
(400 MHz, $CDCl_3$)

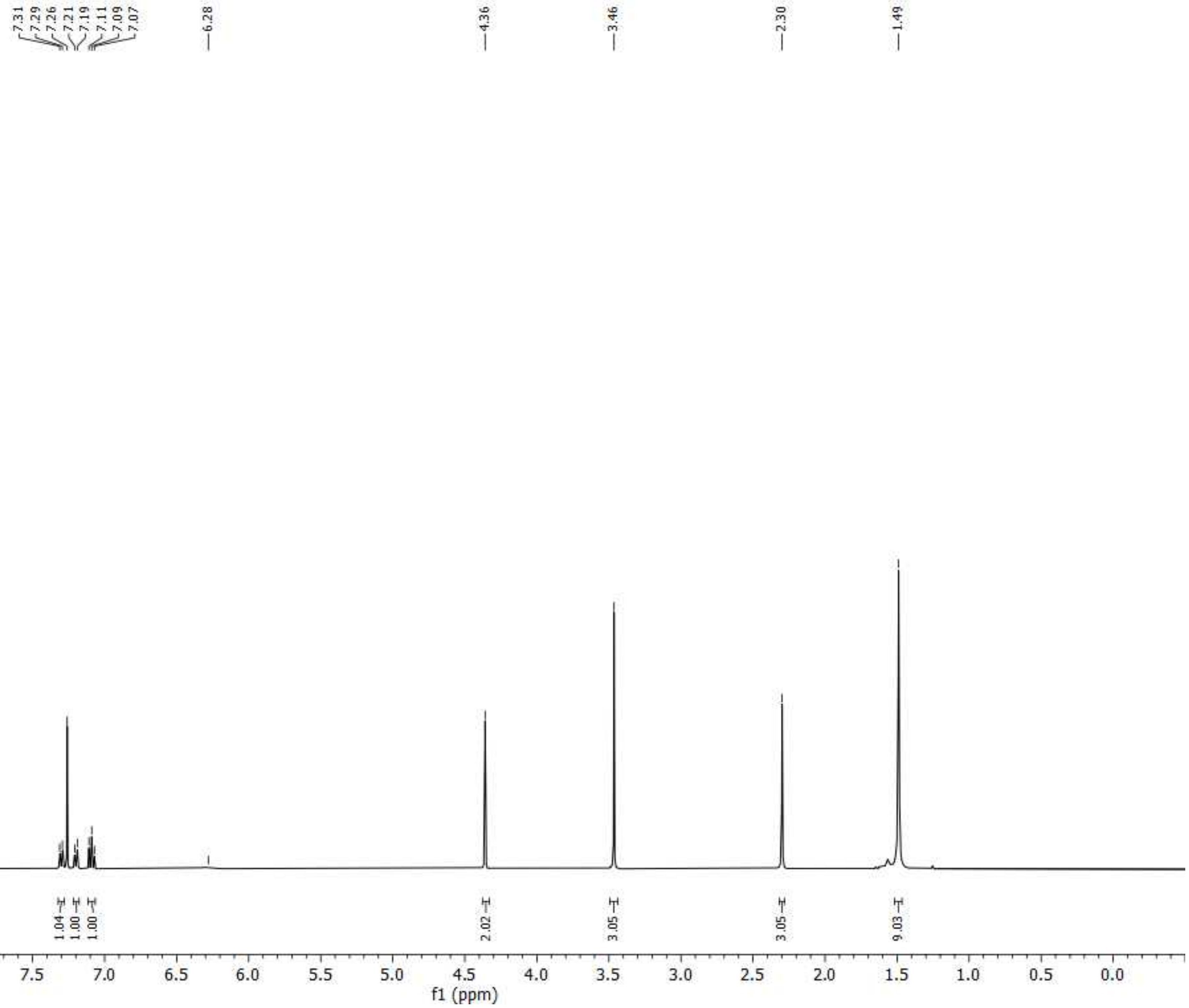

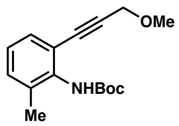

**1h**
**$^{13}C$ NMR**
**(100 MHz, $CDCl_3$)**

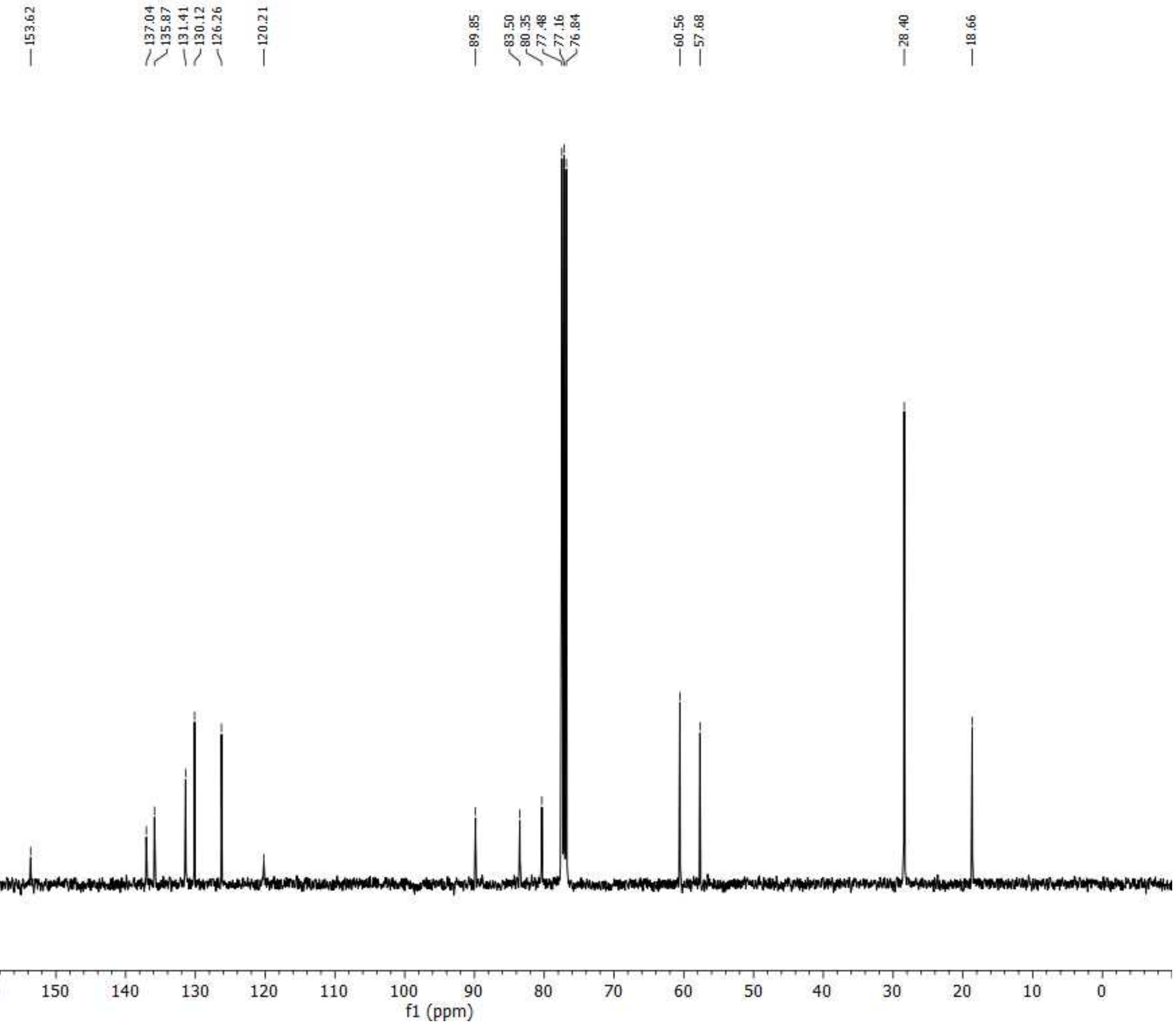

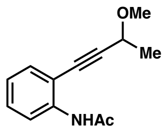


***1i***
$^{1}$H NMR
(400 MHz, $CDCl_3$)

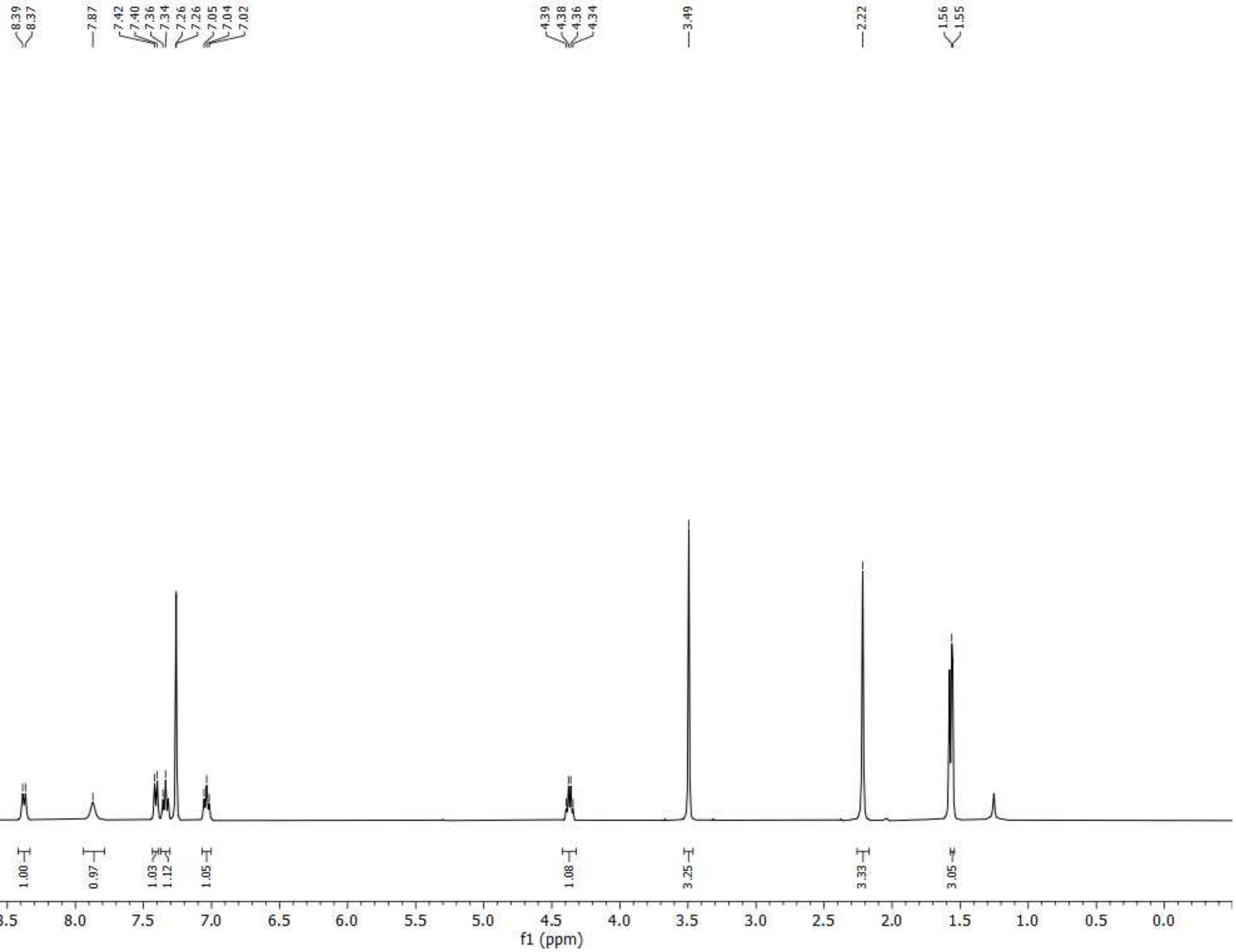

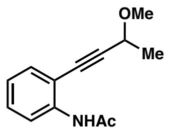


***1i***
$^{13}C$ NMR
(100 MHz, $CDCl_3$)

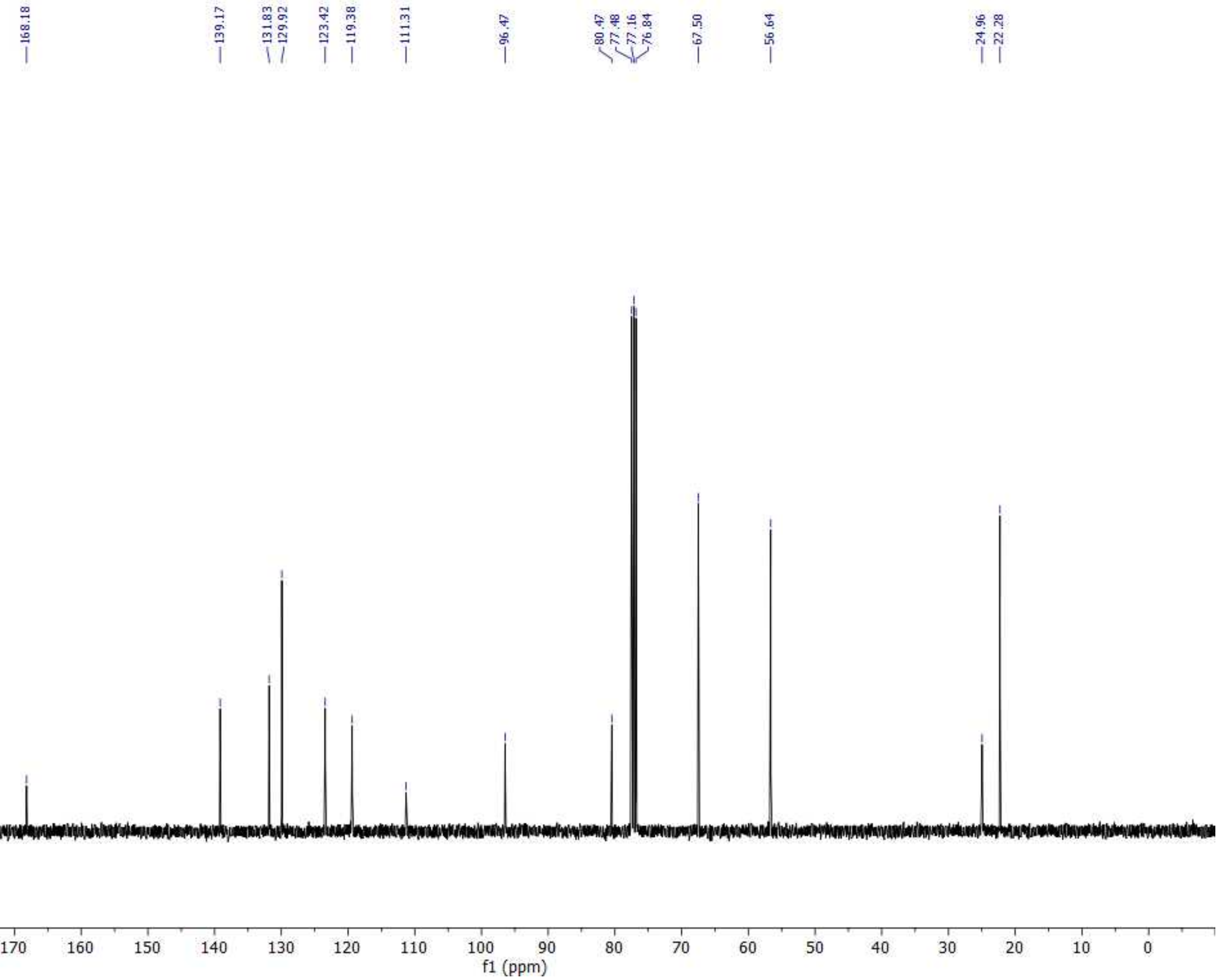

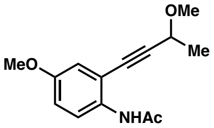


***1j***
$^1$H NMR
(400 MHz, $CDCl_3$)

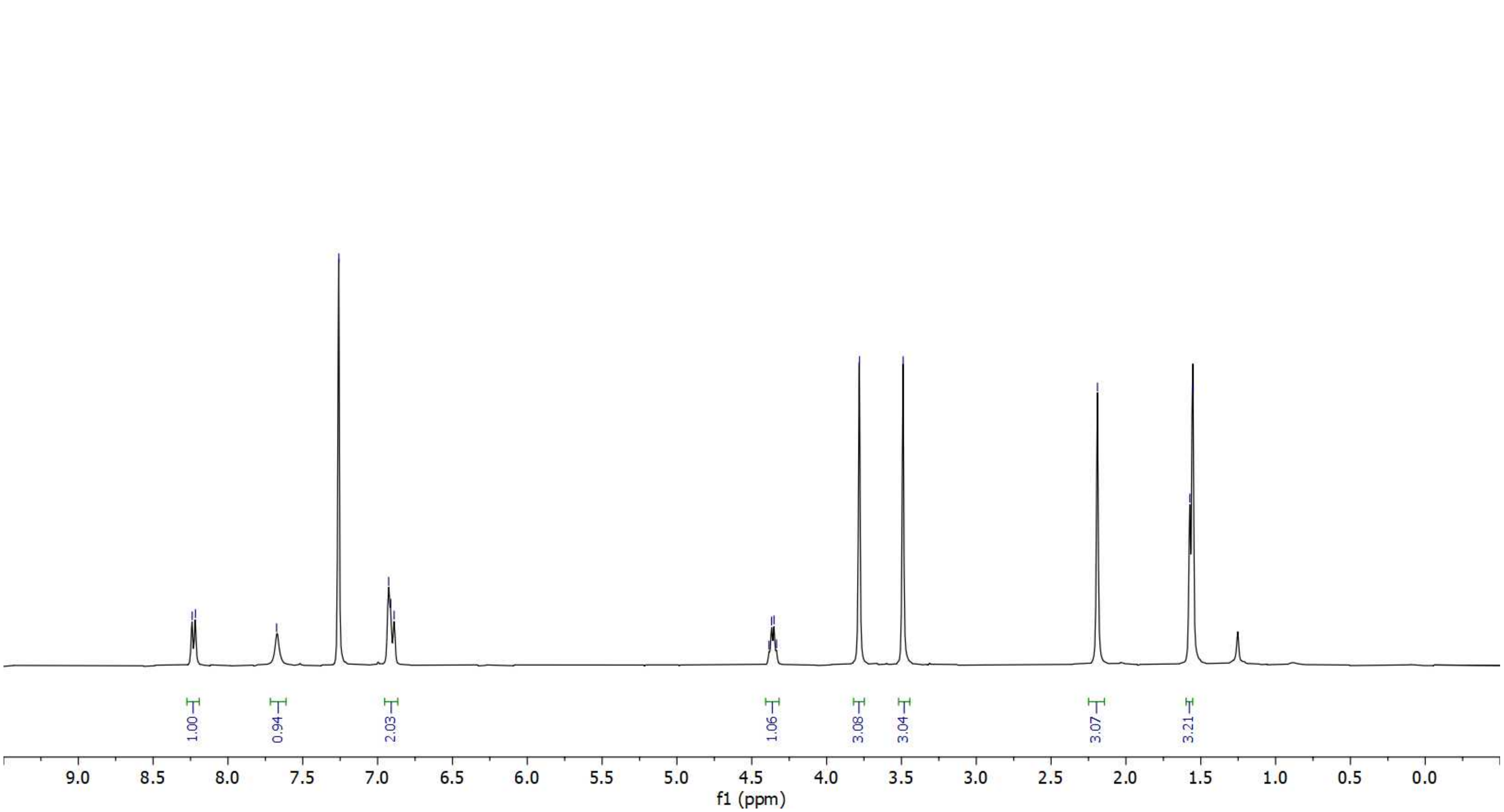

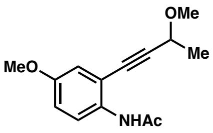

**1j**
**$^{13}C$ NMR**
**(100 MHz, $CDCl_3$)**

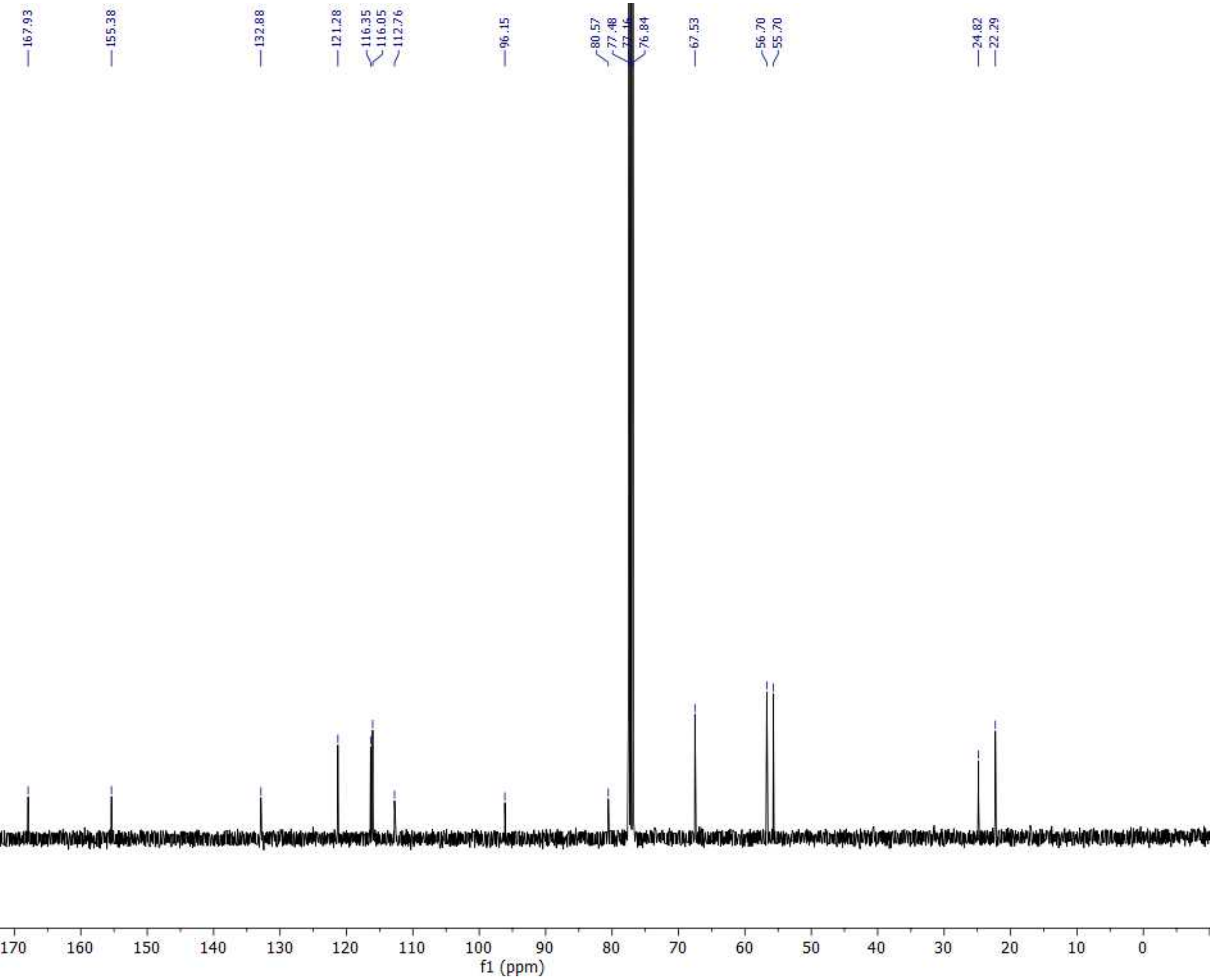

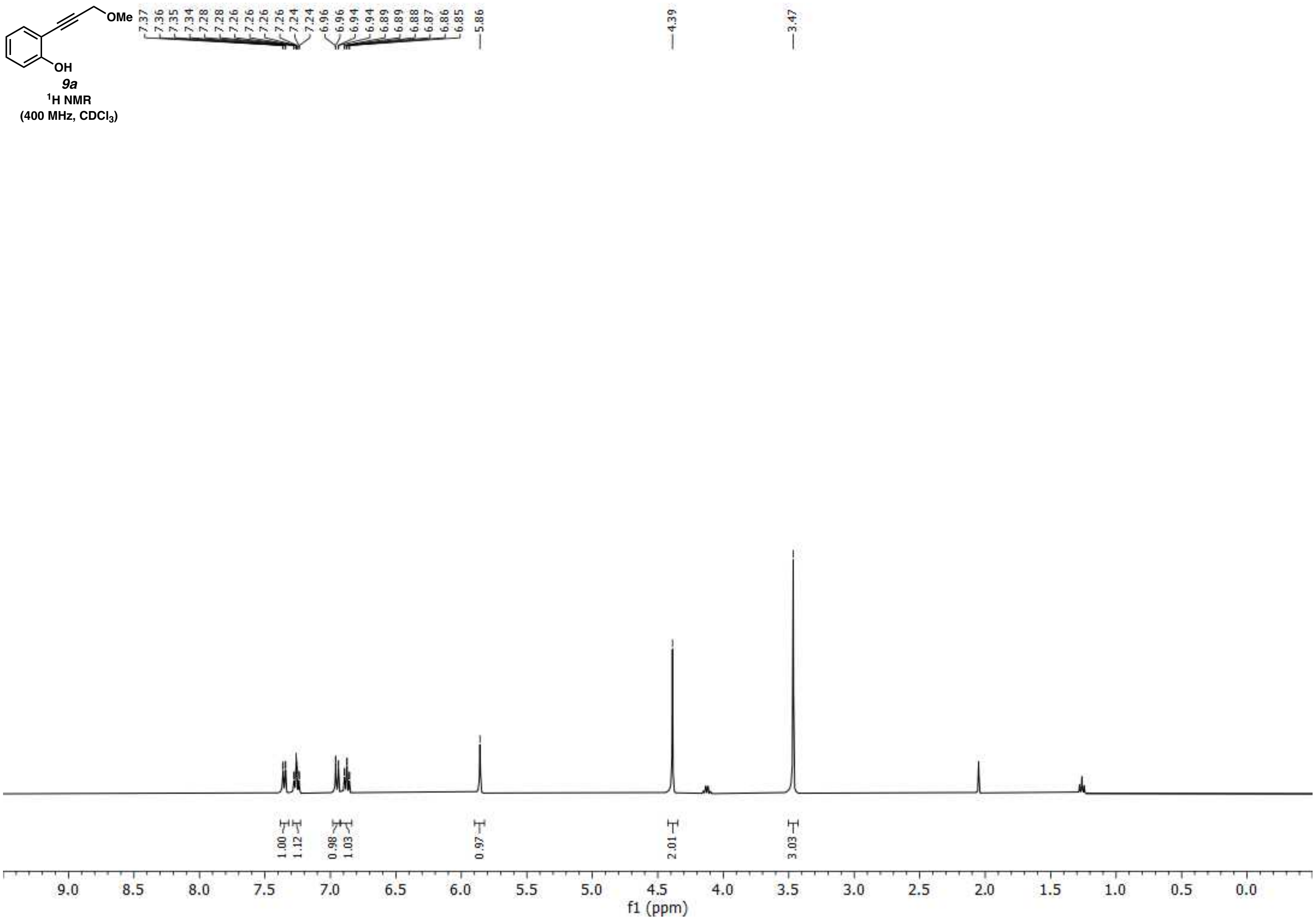
OMe
OH
9a
¹H NMR
(400 MHz, CDCl₃)
7.37
7.36
7.35
7.34
7.28
7.28
7.26
7.26
7.26
7.26
7.24
7.24
6.96
6.96
6.94
6.94
6.89
6.89
6.88
6.87
6.86
6.85
5.86
4.39
3.47
1.00
1.12
0.98
1.03
0.97
2.01
3.03
9.0
8.5
8.0
7.5
7.0
6.5
6.0
5.5
5.0
4.5
4.0
3.5
3.0
2.5
2.0
1.5
1.0
0.5
0.0
f1 (ppm)

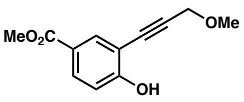


***9b***
$^1$H NMR
(400 MHz, $CDCl_3$)

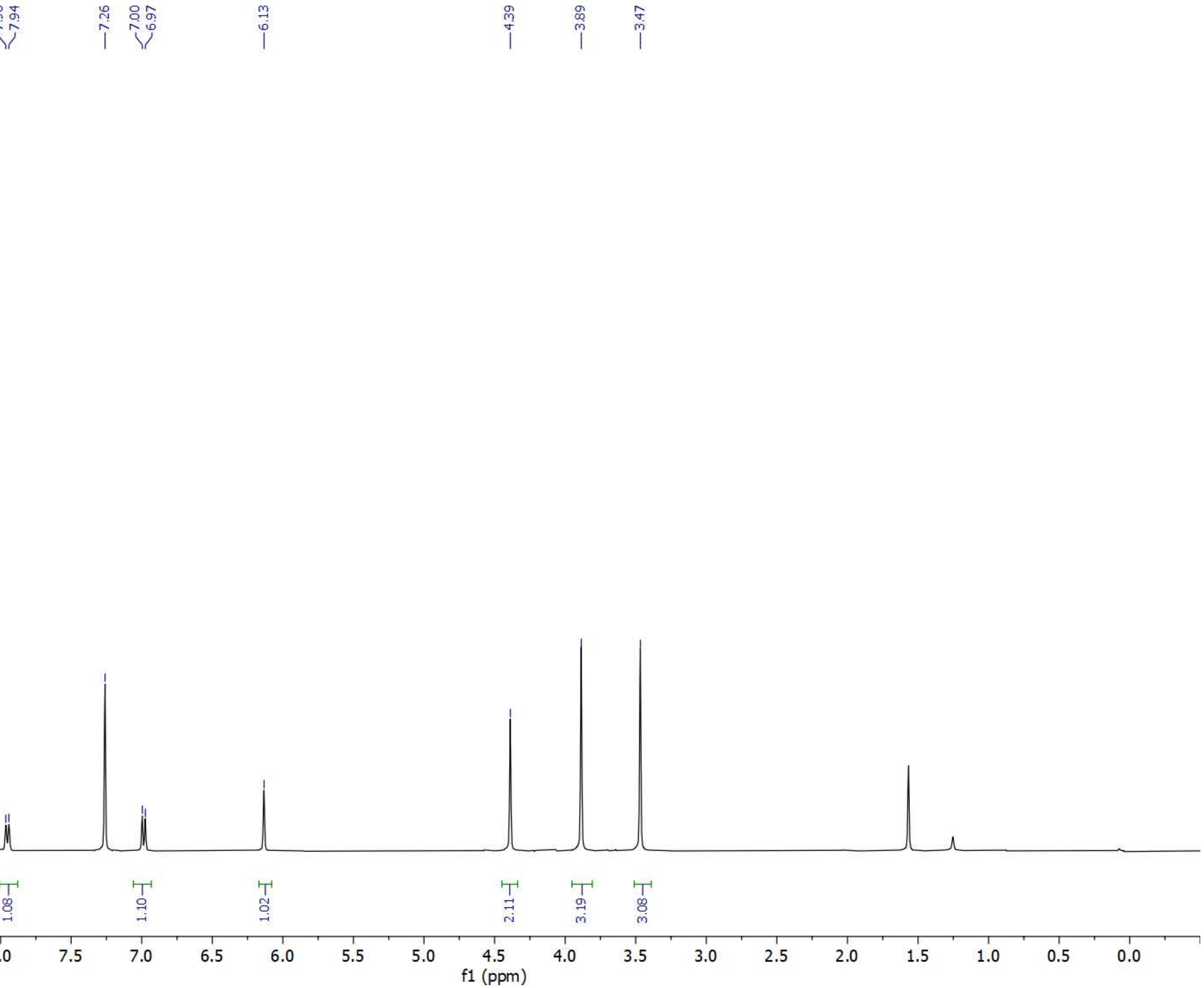

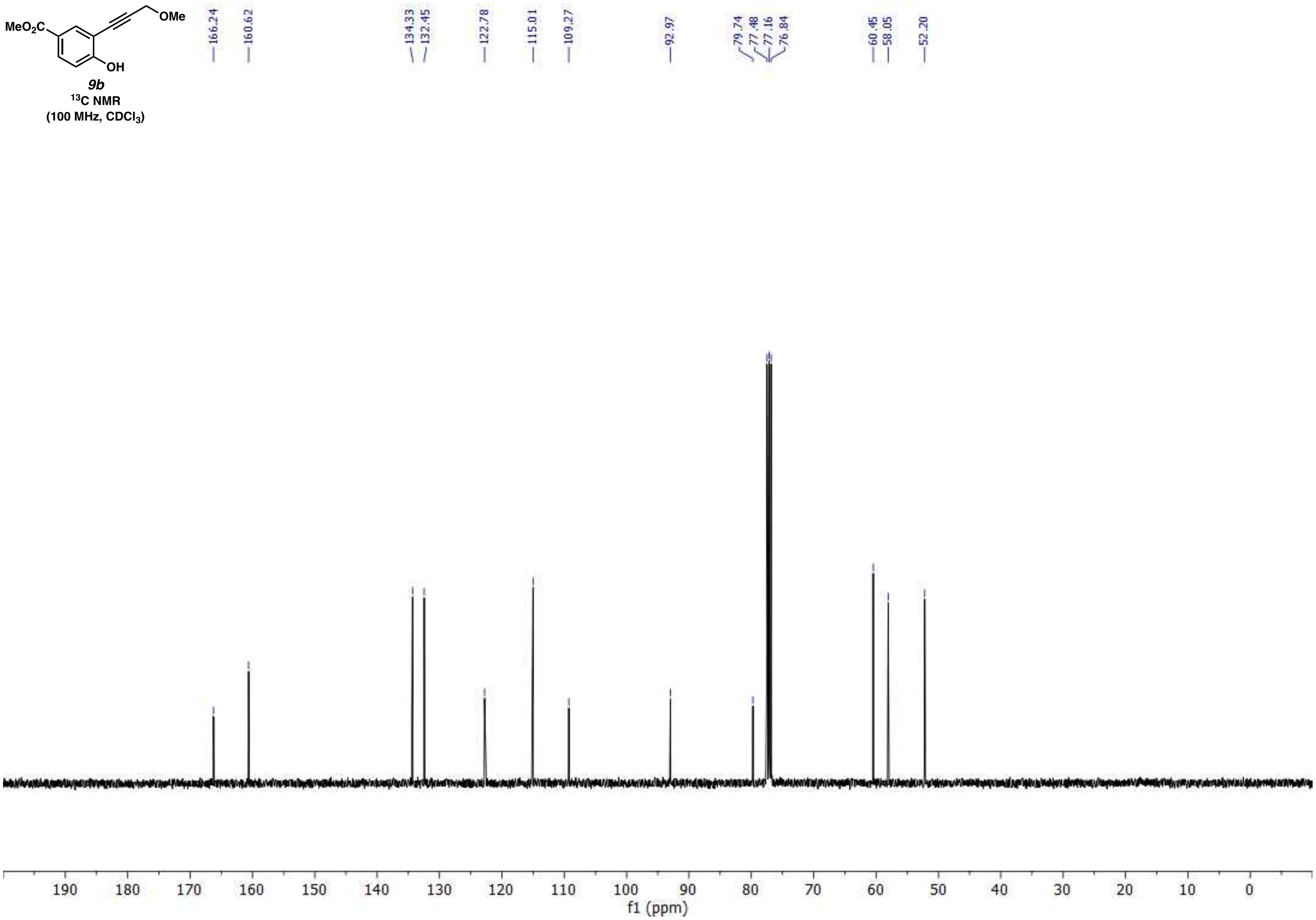


***9b***
**$^{13}C$ NMR**
**(100 MHz, $CDCl_3$)**

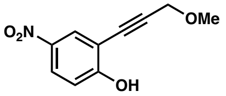


***9c***
$^1$H NMR
(400 MHz, $CDCl_3$)

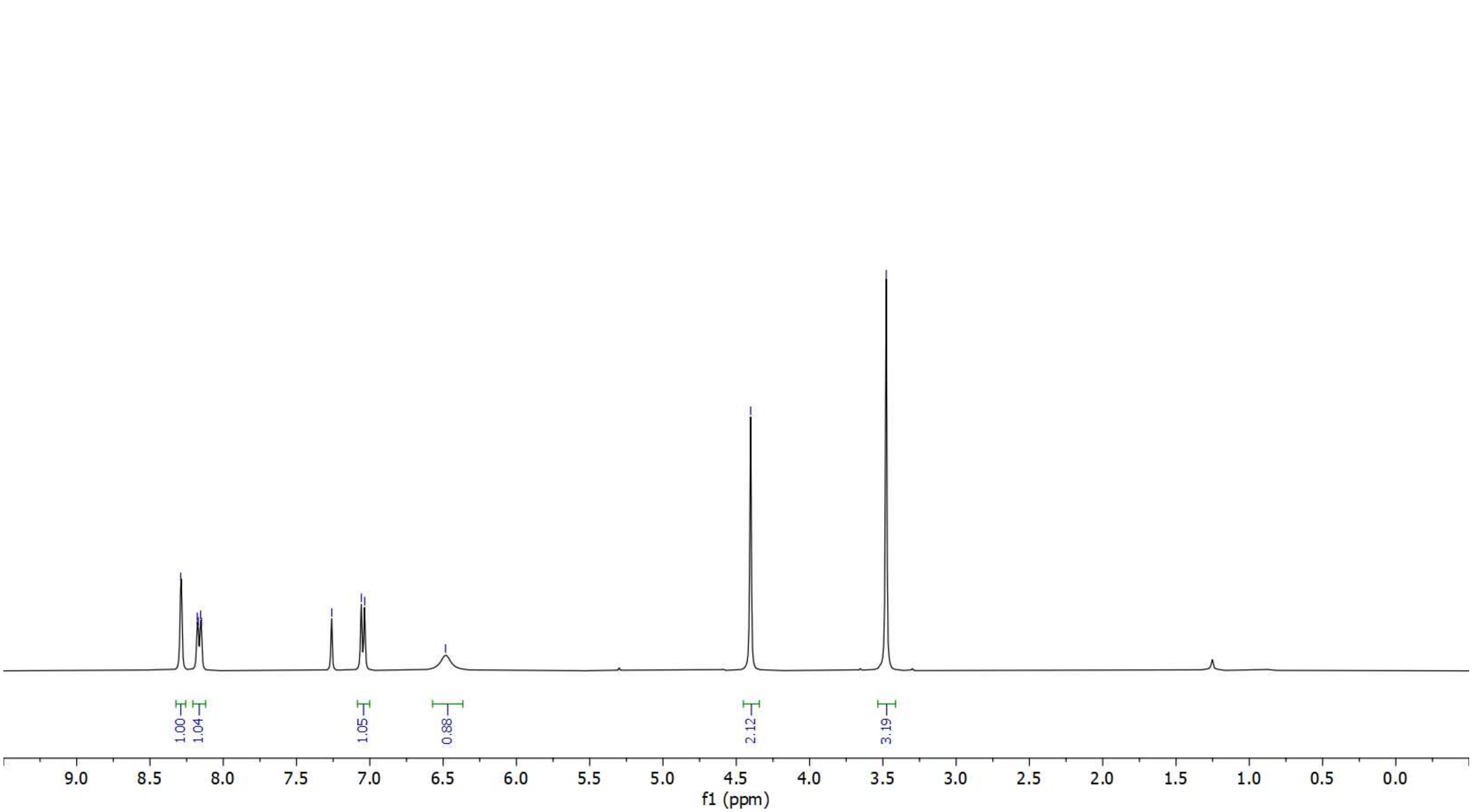

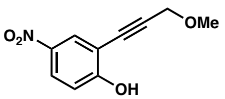


***9c***
**$^{13}C$ NMR**
**(100 MHz, $CDCl_3$)**

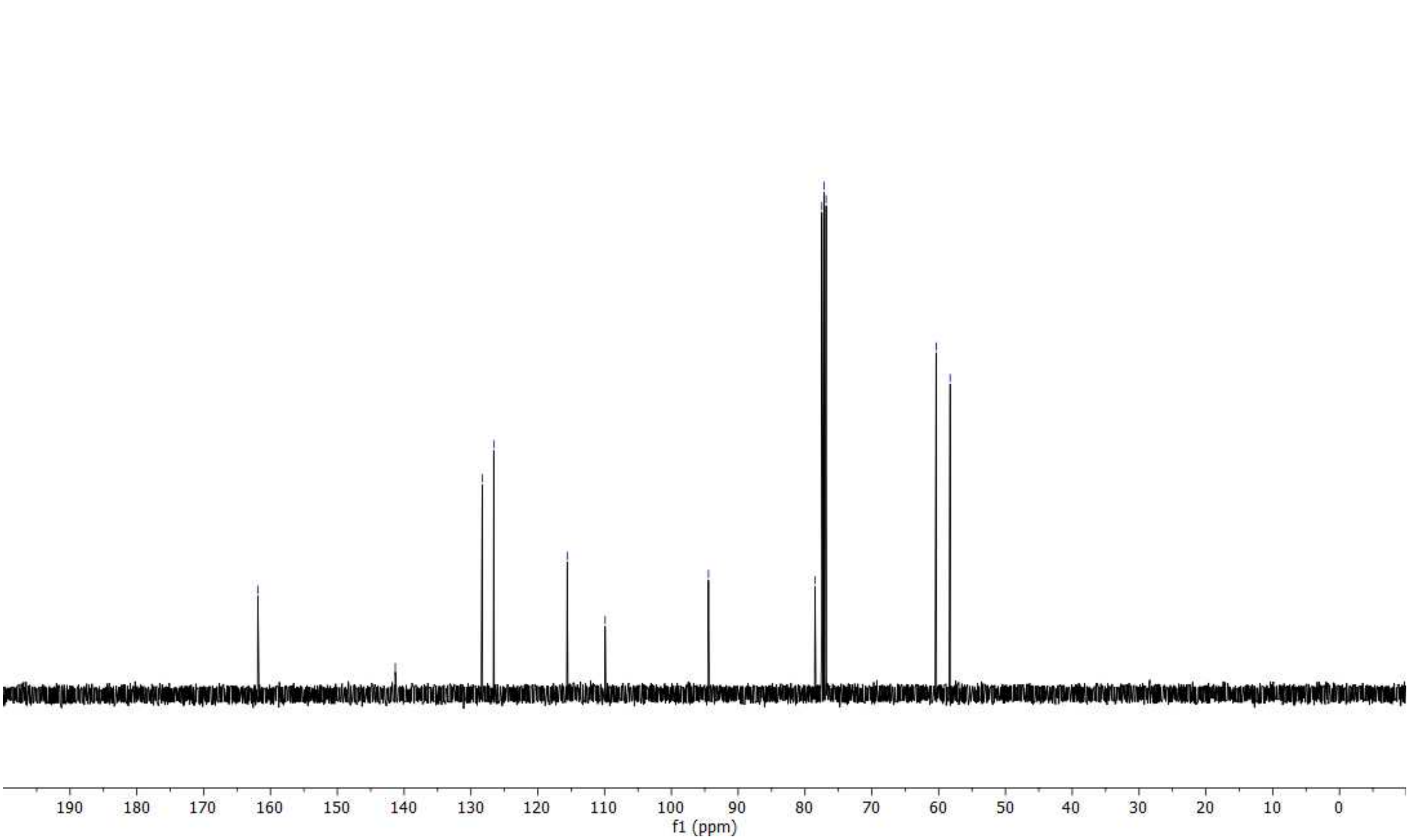

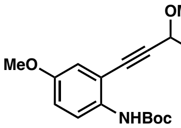


*1k*
$^1$H NMR
(400 MHz, $CDCl_3$)

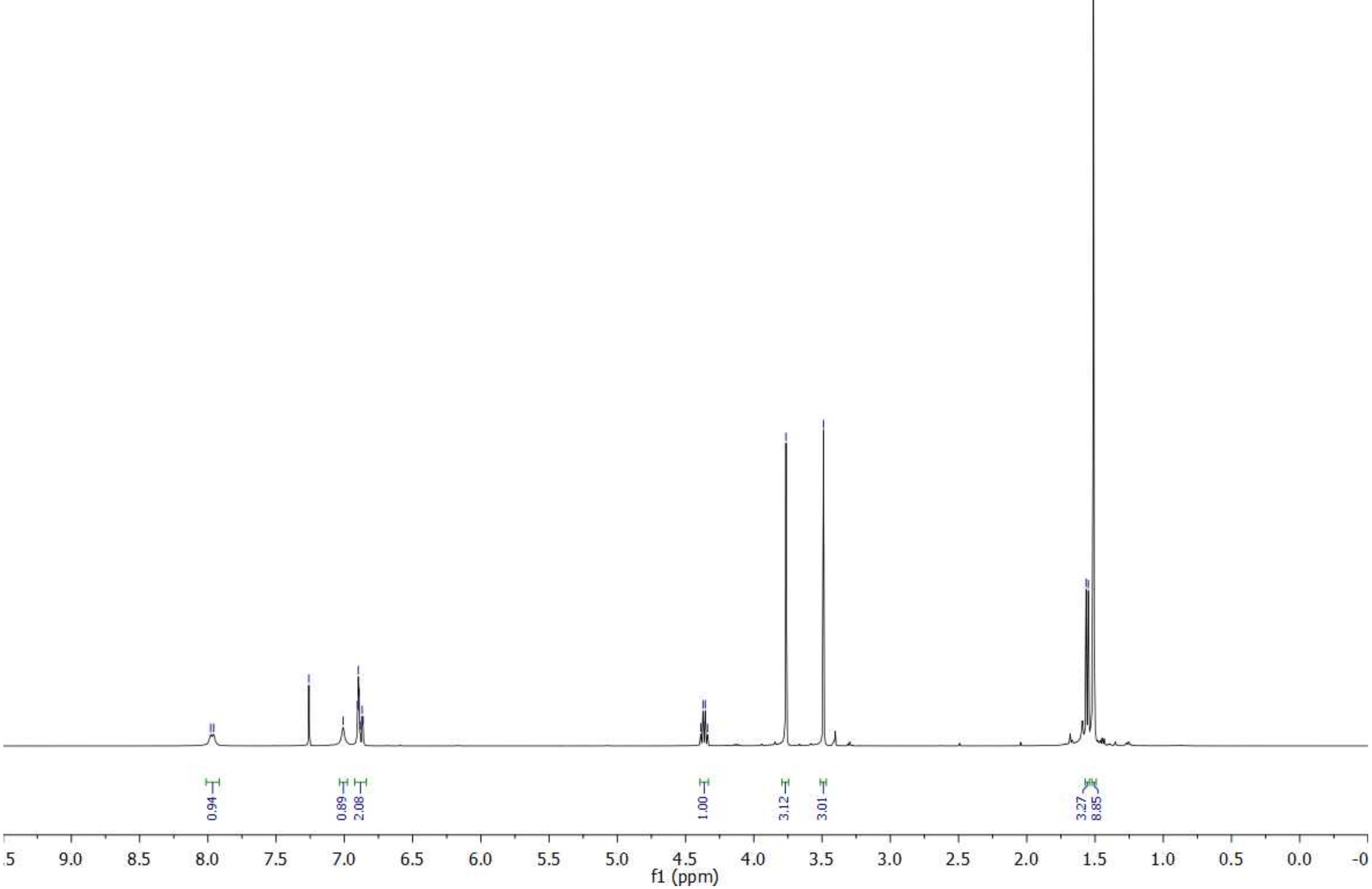

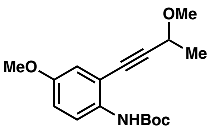


***1k***
$^{13}$C NMR
(100 MHz, $CDCl_3$)

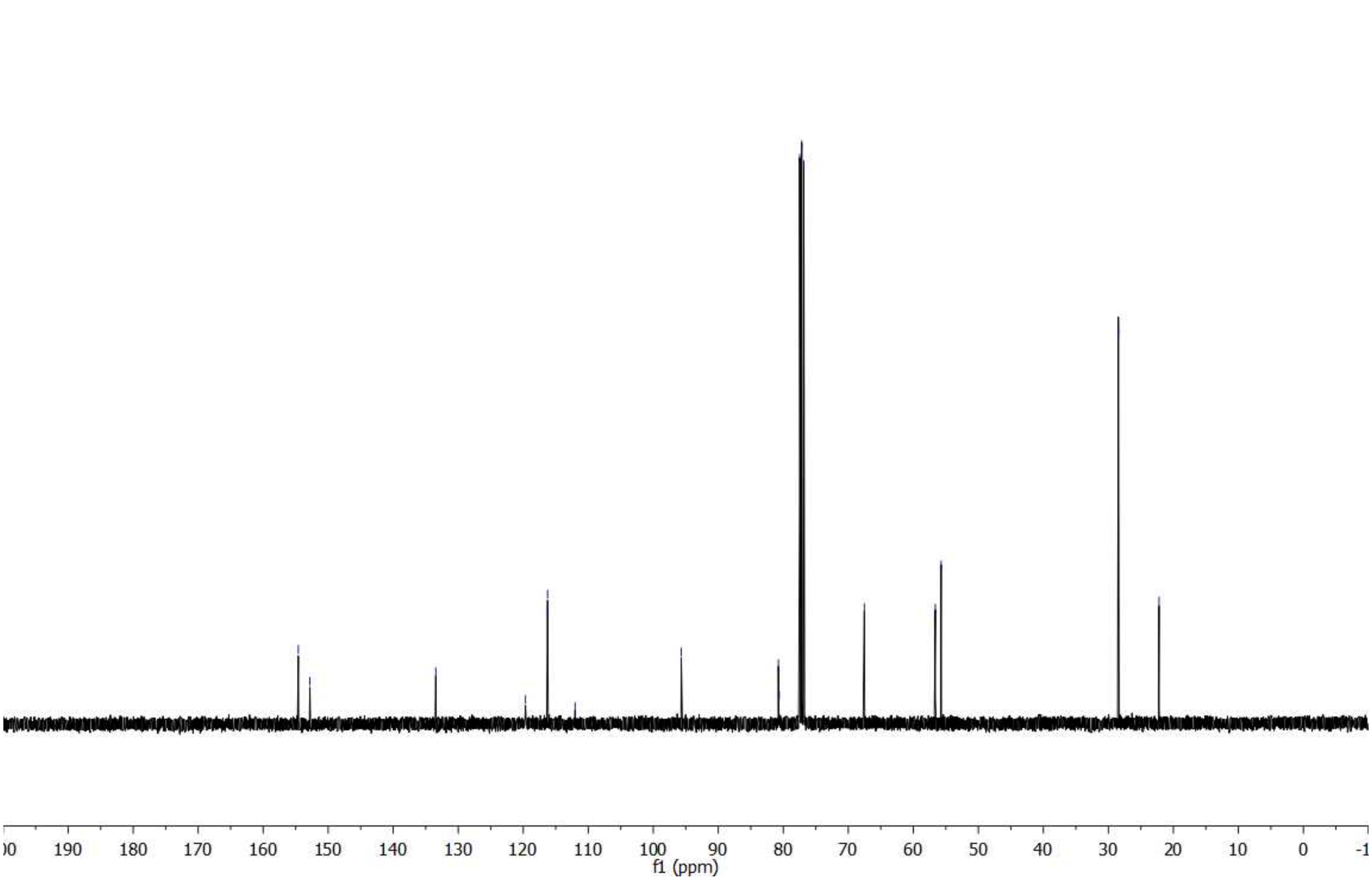

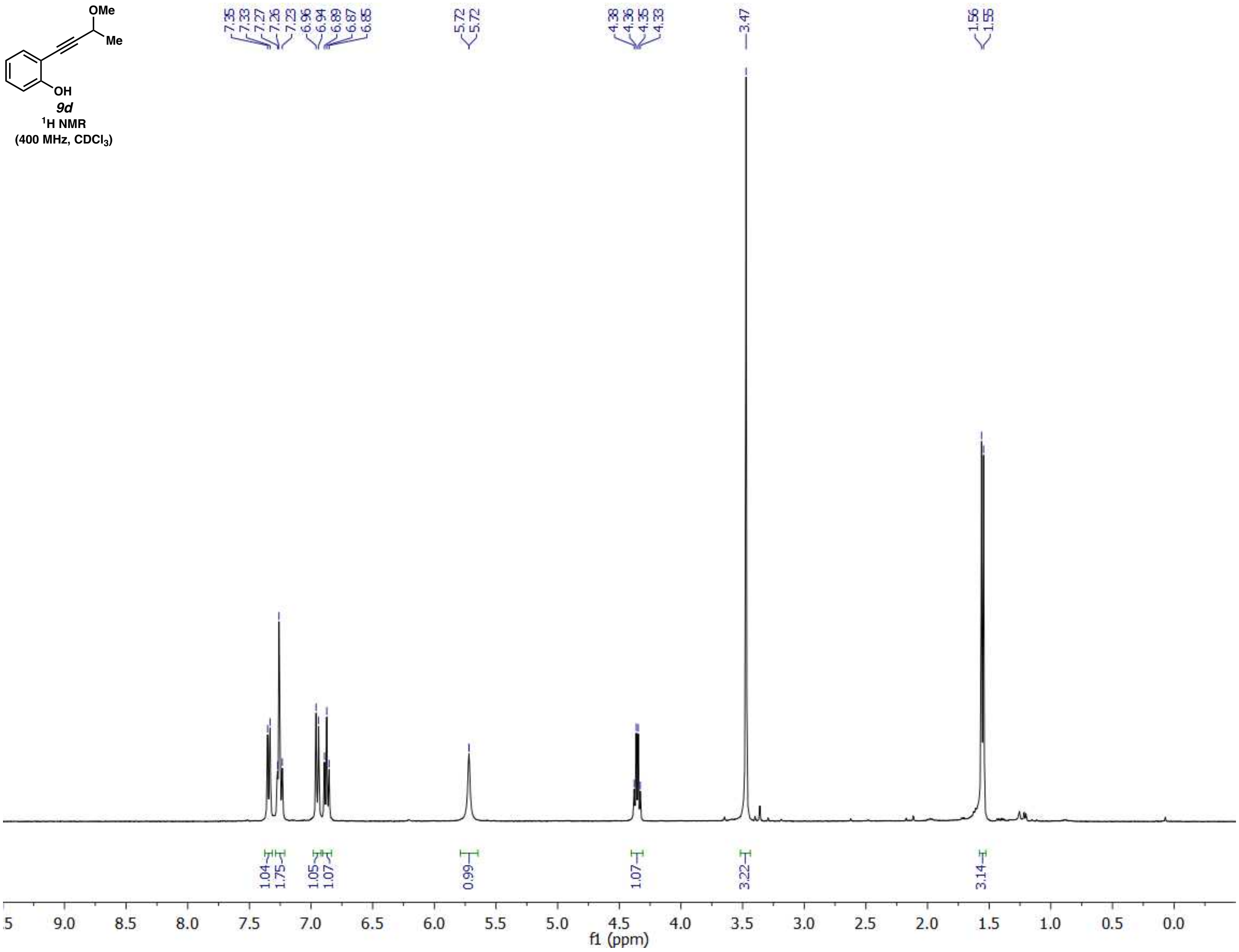
OMe
Me
OH
9d
$^1$H NMR
(400 MHz, CDCl$_3$)
7.35
7.33
7.27
7.26
7.23
6.96
6.94
6.89
6.87
6.85
5.72
5.72
4.38
4.36
4.35
4.33
3.47
1.56
1.55
1.04
1.75
1.05
1.07
0.99
1.07
3.22
3.14
9.0
8.5
8.0
7.5
7.0
6.5
6.0
5.5
5.0
4.5
4.0
3.5
3.0
2.5
2.0
1.5
1.0
0.5
0.0
f1 (ppm)

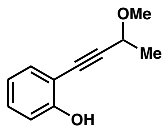

**9d**

$^{13}$C NMR

(100 MHz, $CDCl_3$)

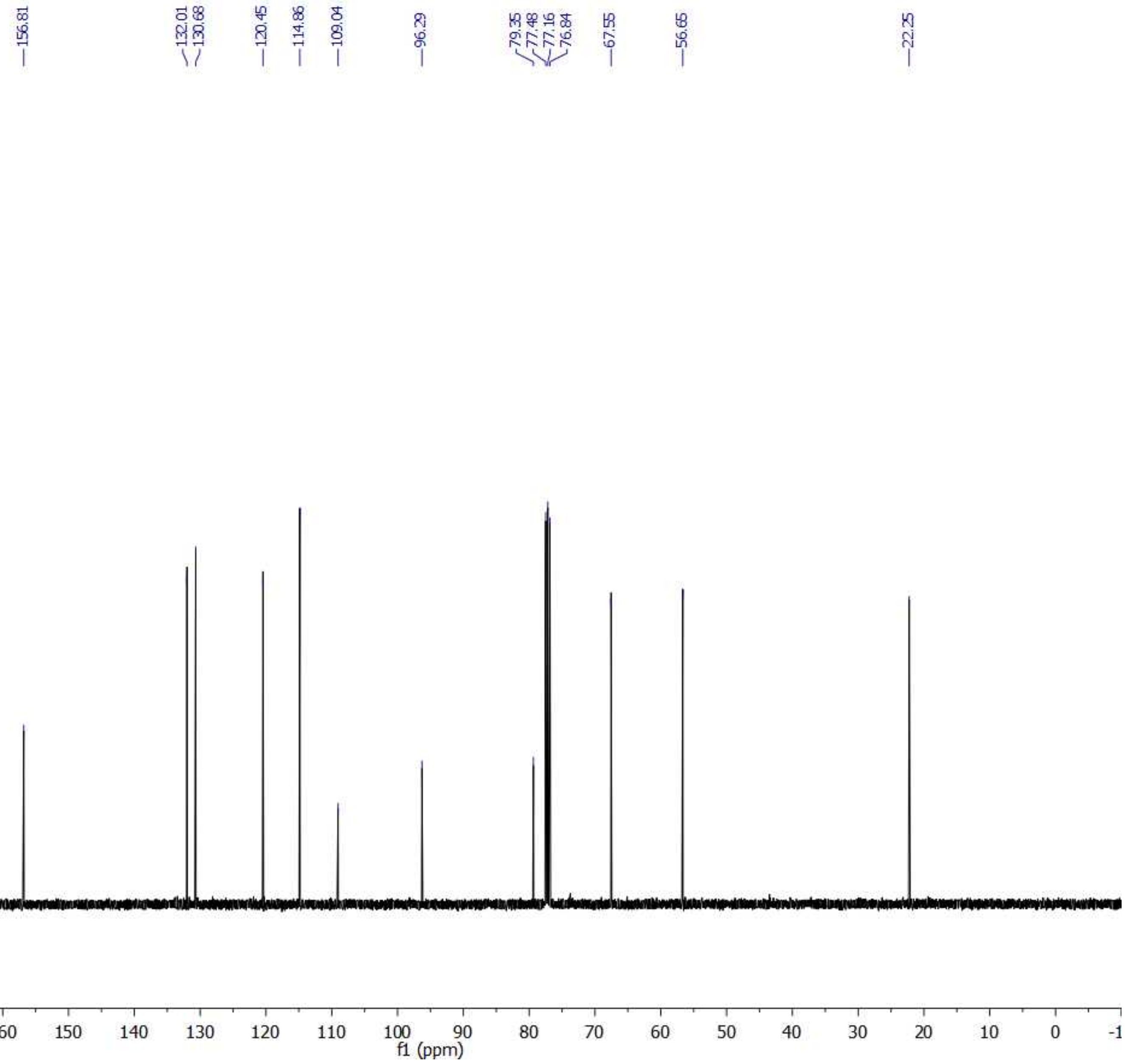

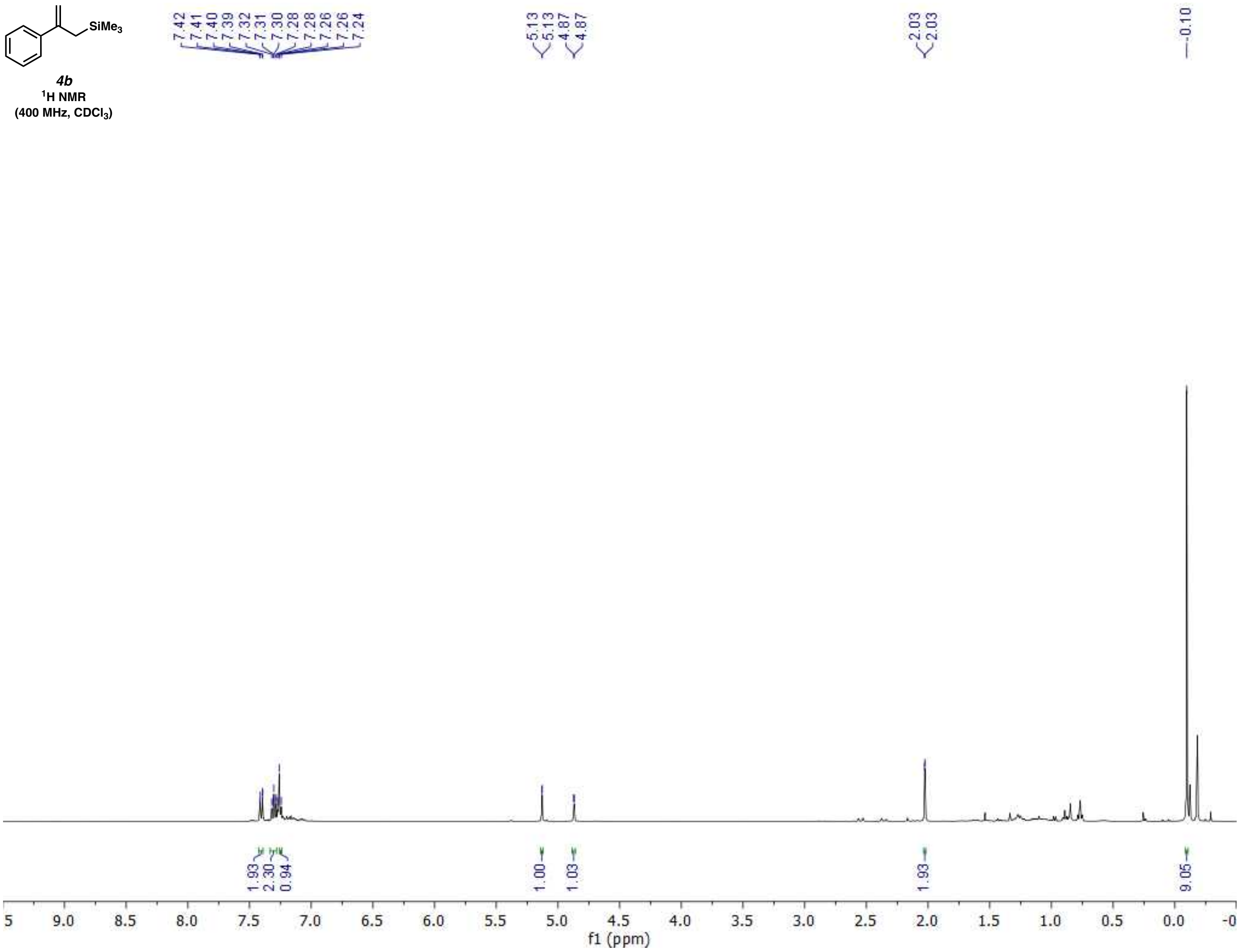


***4b***
**$^{1}$H NMR**
**(400 MHz, $CDCl_3$)**

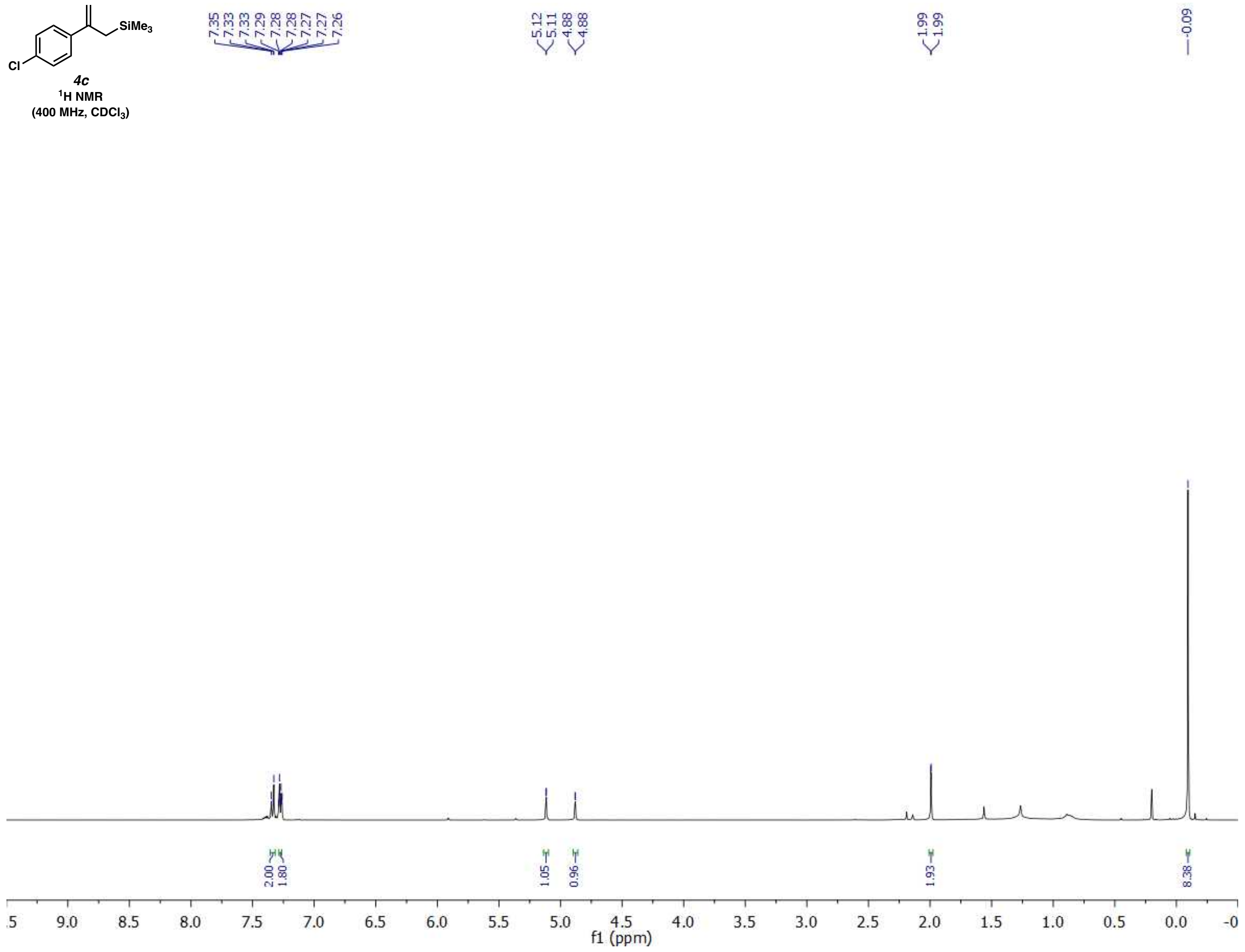
SiMe3
Cl
4c
1H NMR
(400 MHz, CDCl3)
7.35
7.33
7.33
7.29
7.28
7.28
7.27
7.27
7.26
5.12
5.11
4.88
4.88
1.99
1.99
-0.09
2.00
1.80
1.05
0.96
1.93
8.38
.5
9.0
8.5
8.0
7.5
7.0
6.5
6.0
5.5
5.0
4.5
4.0
3.5
3.0
2.5
2.0
1.5
1.0
0.5
0.0
-0
f1 (ppm)

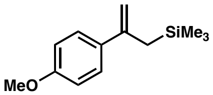

**4d**
$^1$H NMR
(400 MHz, $CDCl_3$)

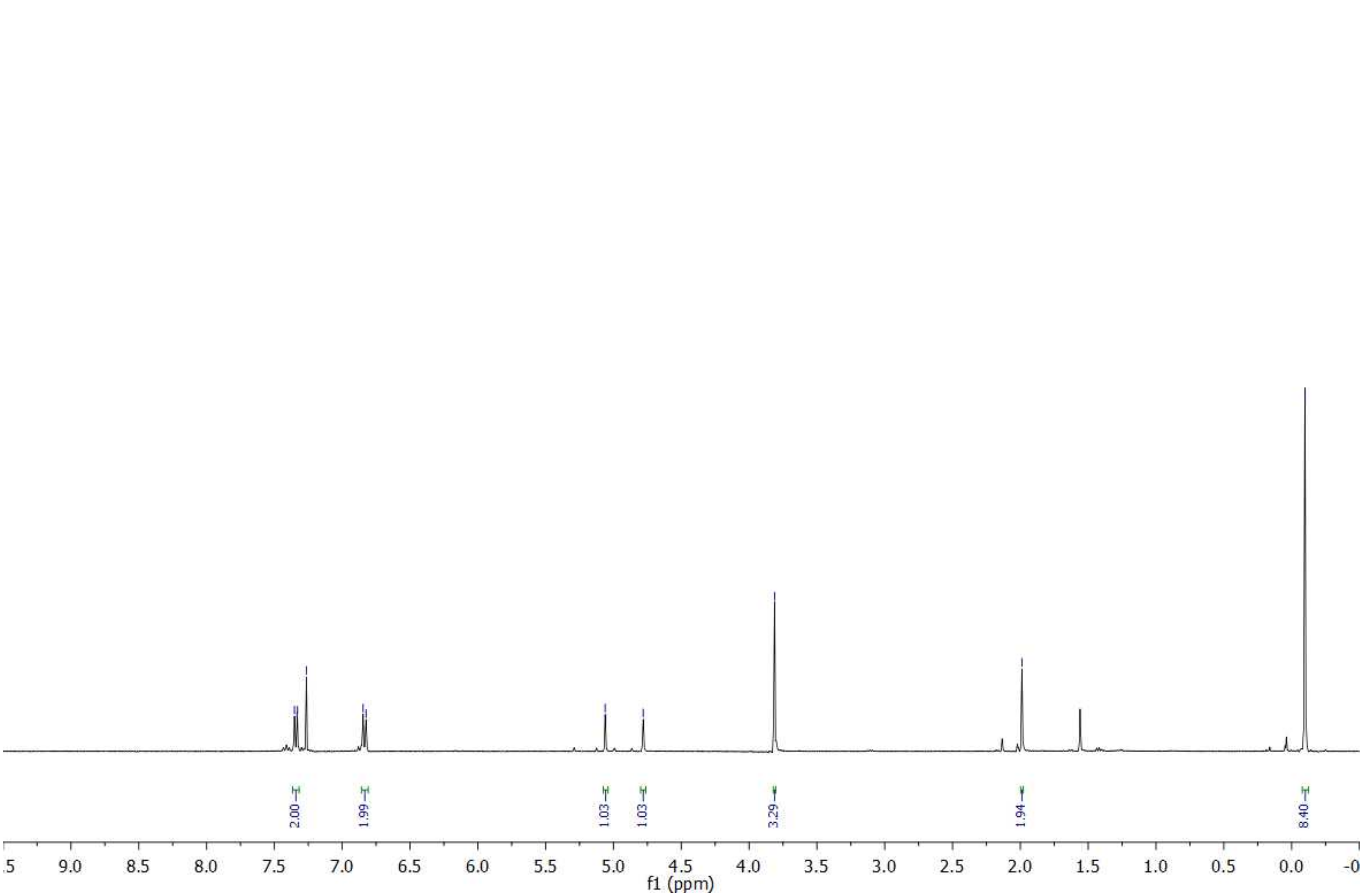

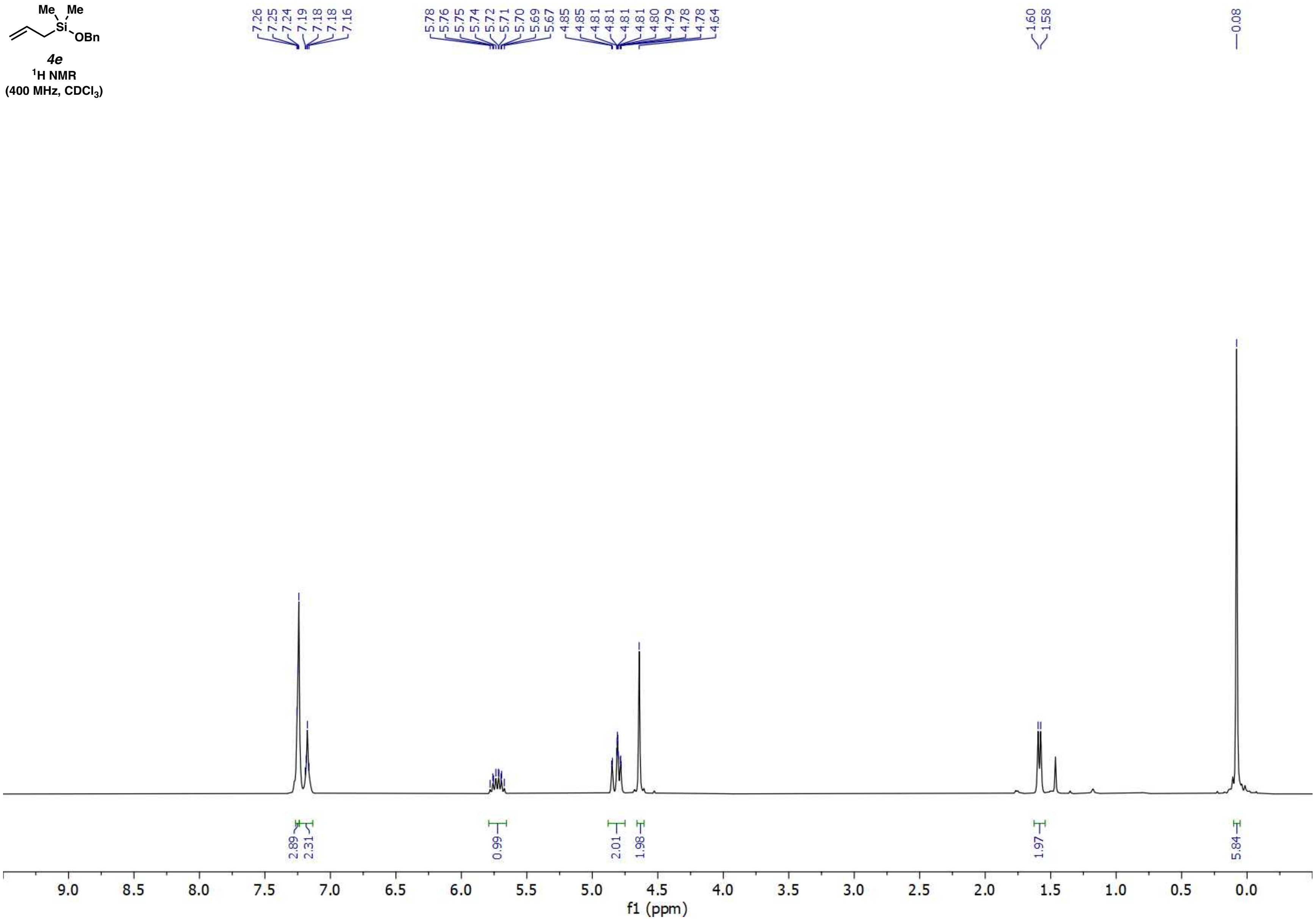
Me Me
Si
OBn
4e
¹H NMR
(400 MHz, CDCl₃)
7.26
7.25
7.24
7.19
7.18
7.18
7.16
5.78
5.76
5.75
5.74
5.72
5.71
5.70
5.69
5.67
4.85
4.85
4.81
4.81
4.81
4.81
4.80
4.79
4.78
4.78
4.64
1.60
1.58
0.08
2.89
2.31
0.99
2.01
1.98
1.97
5.84
9.0
8.5
8.0
7.5
7.0
6.5
6.0
5.5
5.0
4.5
4.0
3.5
3.0
2.5
2.0
1.5
1.0
0.5
0.0
f1 (ppm)

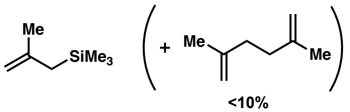


***4f***
$^1$H NMR
(400 MHz, $CDCl_3$)

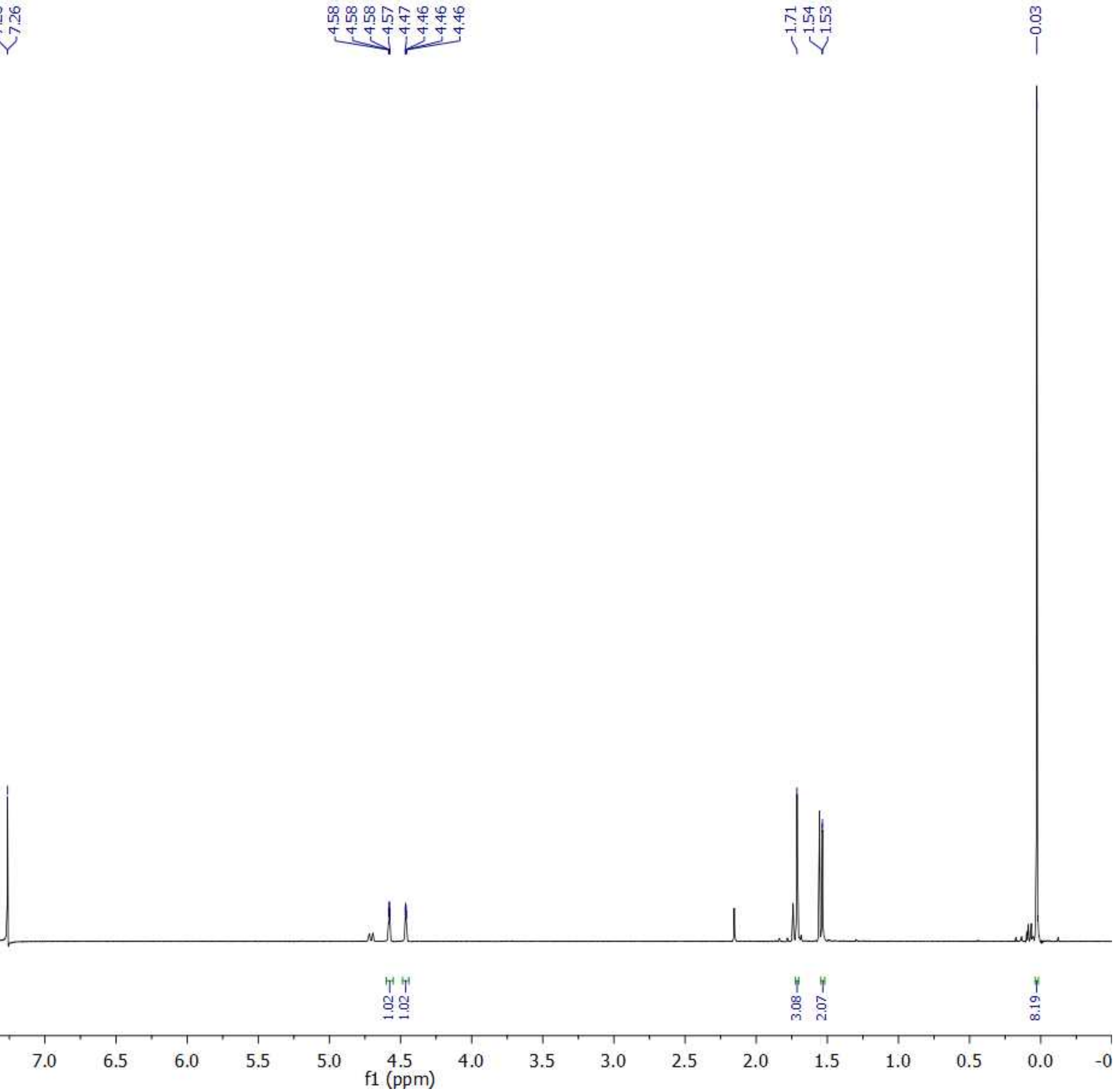

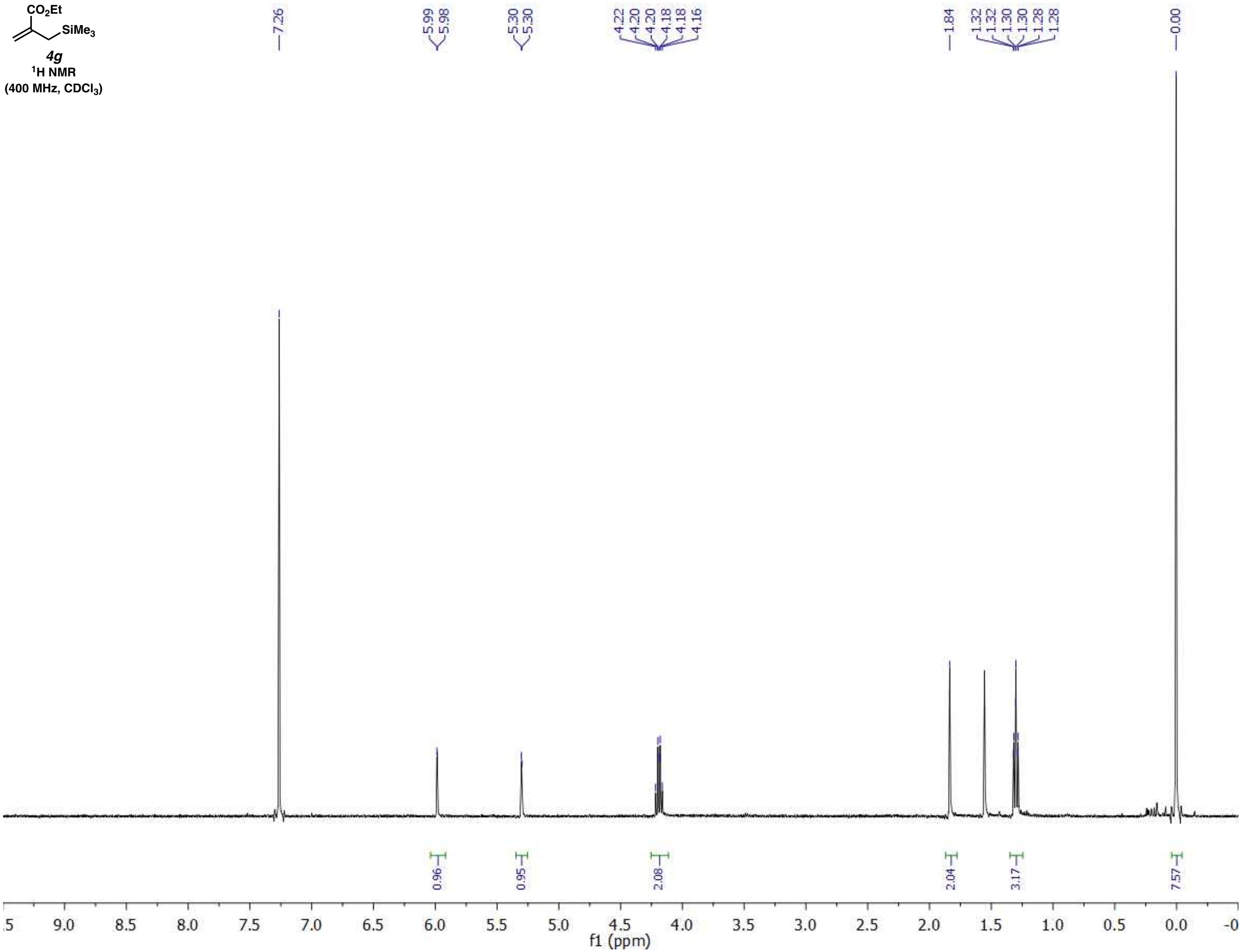
CO2Et
SiMe3
4g
1H NMR
(400 MHz, CDCl3)
7.26
5.99
5.98
5.30
5.30
4.22
4.20
4.20
4.18
4.18
4.16
1.84
1.32
1.32
1.30
1.30
1.28
1.28
0.00
0.96
0.95
2.08
2.04
3.17
7.57
f1 (ppm)

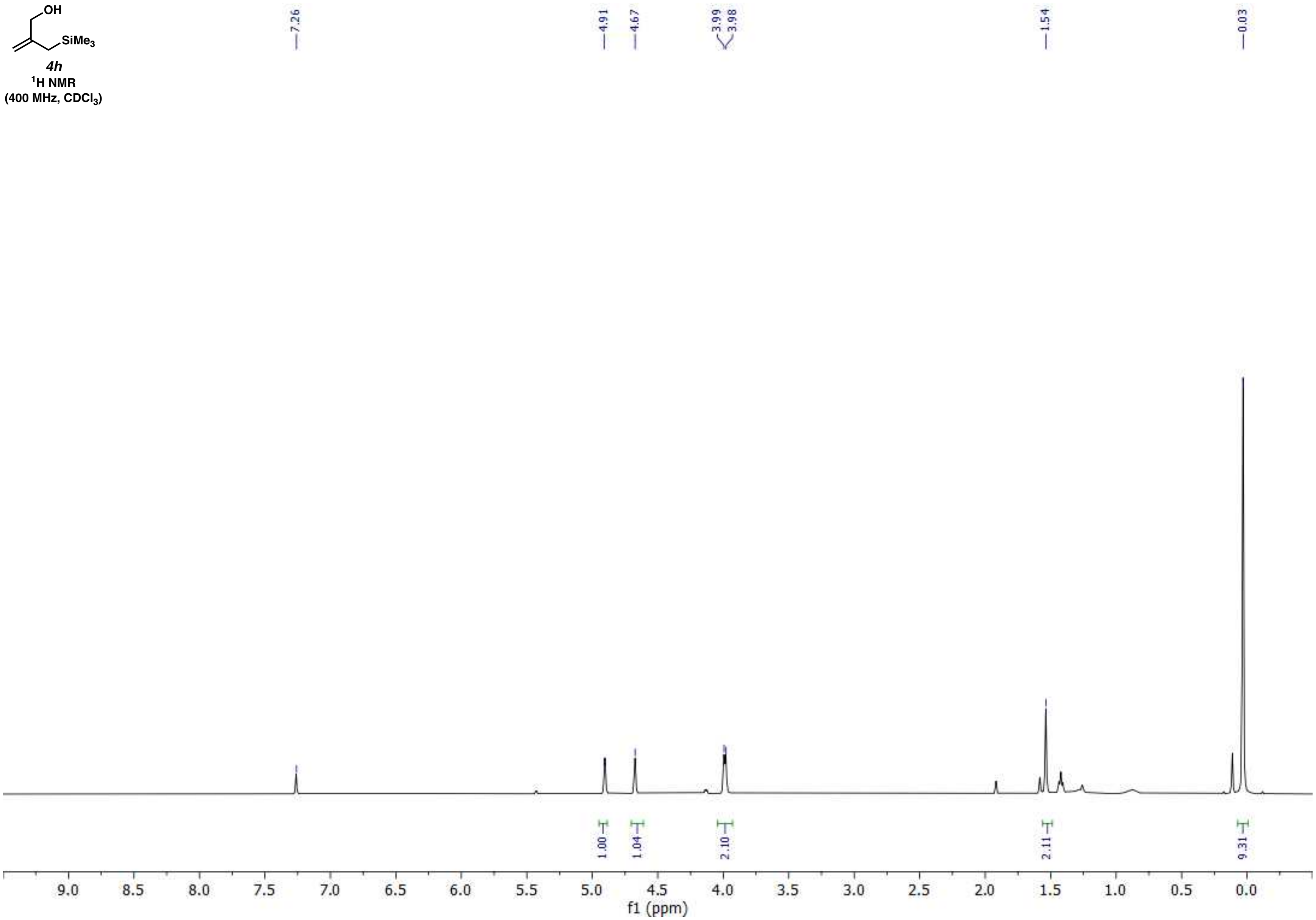
OH
SiMe3
4h
1H NMR
(400 MHz, CDCl3)
7.26
4.91
4.67
3.99
3.98
1.54
0.03
1.00
1.04
2.10
2.11
9.31
9.0
8.5
8.0
7.5
7.0
6.5
6.0
5.5
5.0
4.5
4.0
3.5
3.0
2.5
2.0
1.5
1.0
0.5
0.0
f1 (ppm)

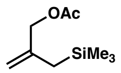

***4i***
$^1$H NMR
(400 MHz, $CDCl_3$)

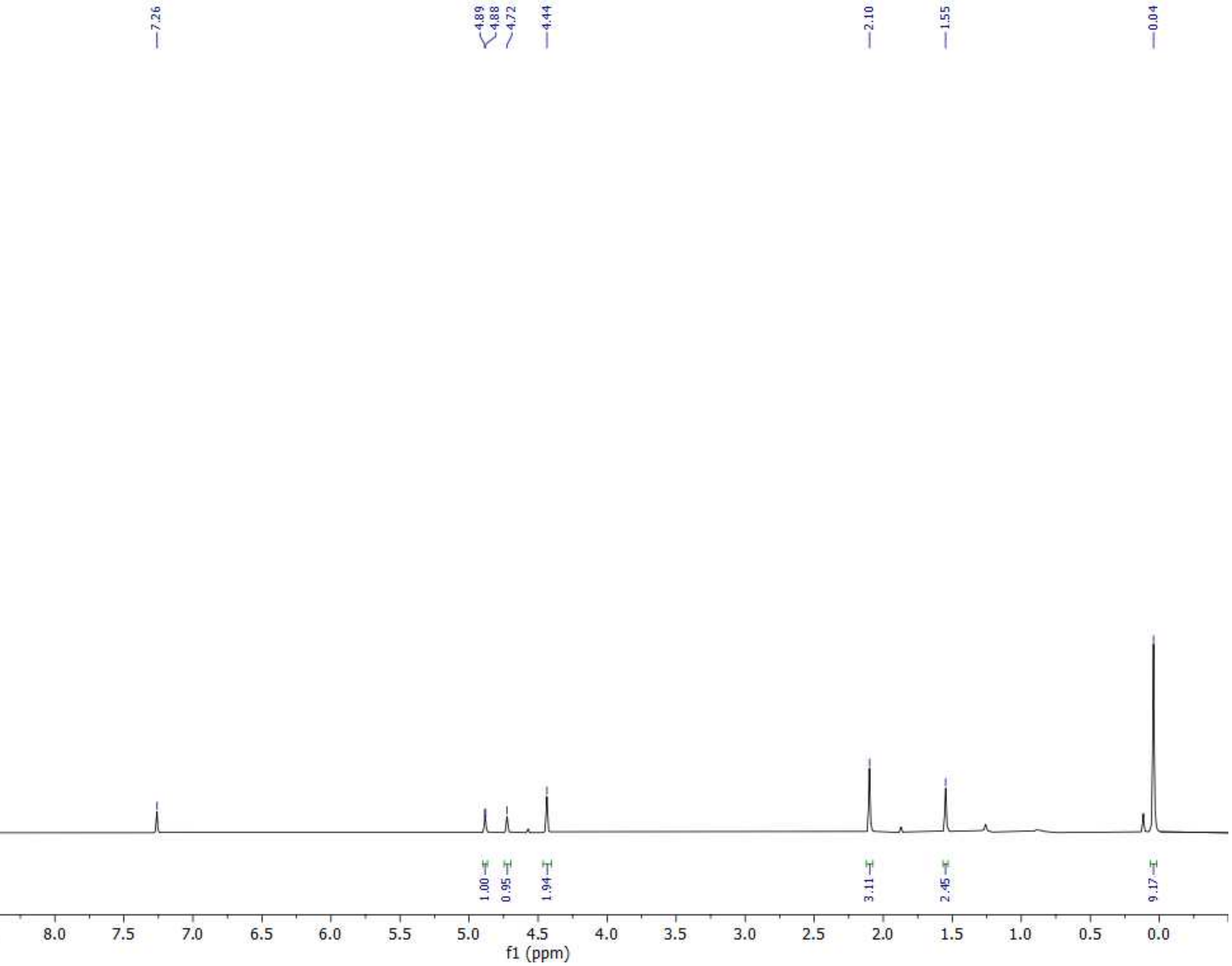

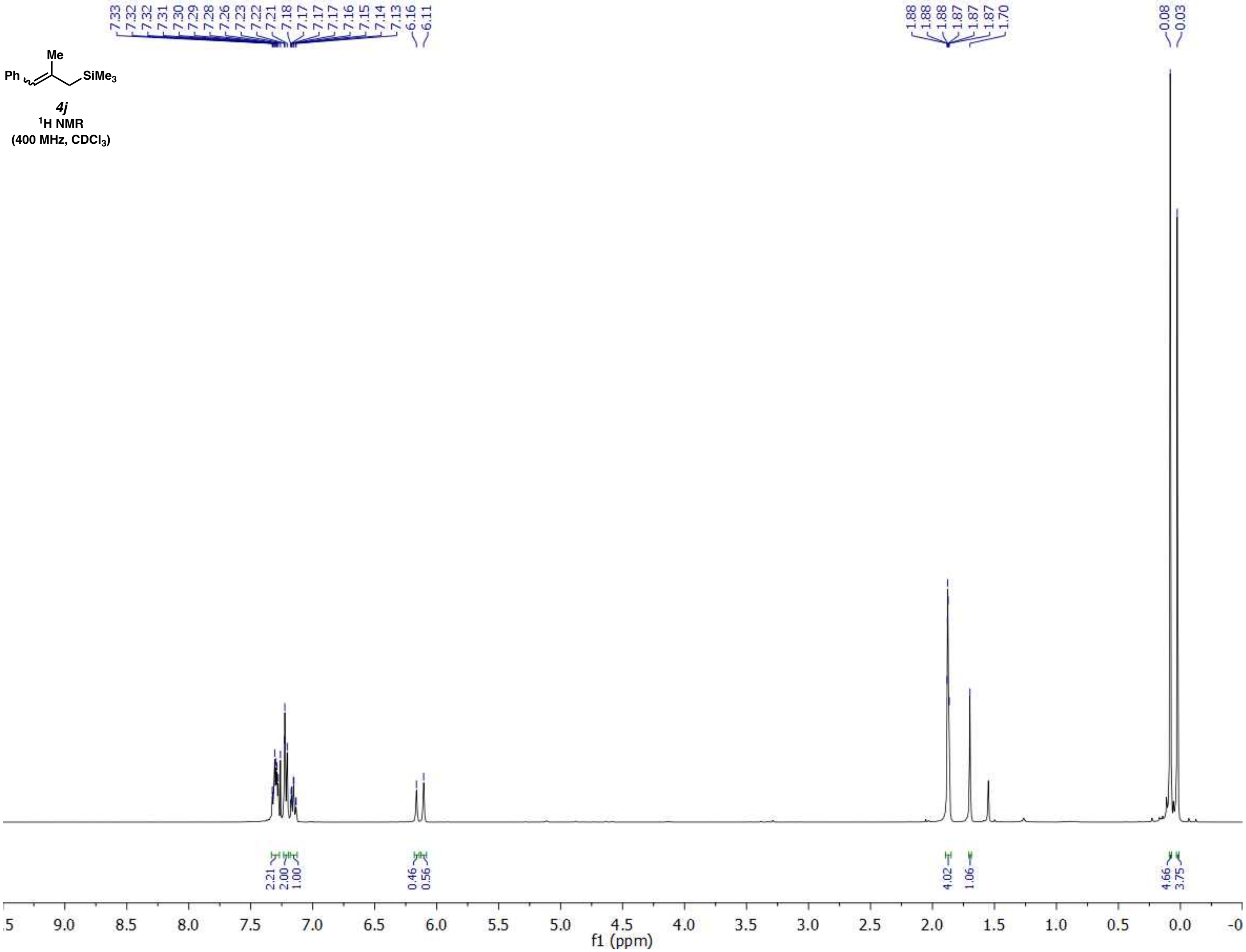
Me
Ph
SiMe3
4j
1H NMR
(400 MHz, CDCl3)
7.33
7.32
7.32
7.31
7.30
7.29
7.28
7.26
7.23
7.22
7.21
7.18
7.17
7.17
7.17
7.16
7.15
7.14
7.13
6.16
6.11
1.88
1.88
1.88
1.87
1.87
1.87
1.70
0.08
0.03
2.21
2.00
1.00
0.46
0.56
4.02
1.06
4.66
3.75
f1 (ppm)

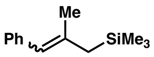


***4j***
$^{13}$C NMR
(100 MHz, $CDCl_3$)

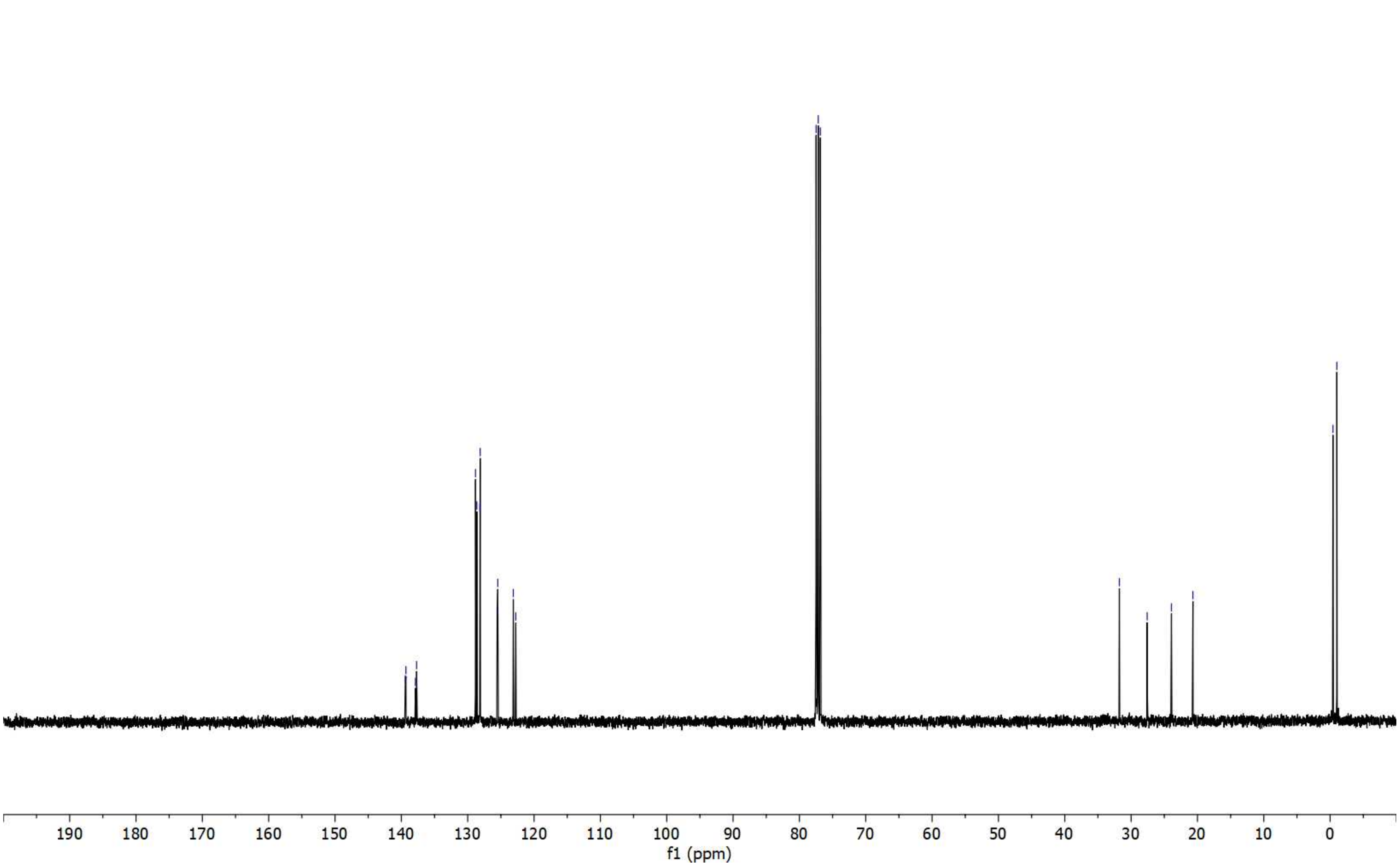

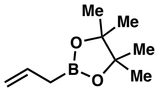

***19***
$^{1}H$ NMR
(400 MHz, $CDCl_3$)

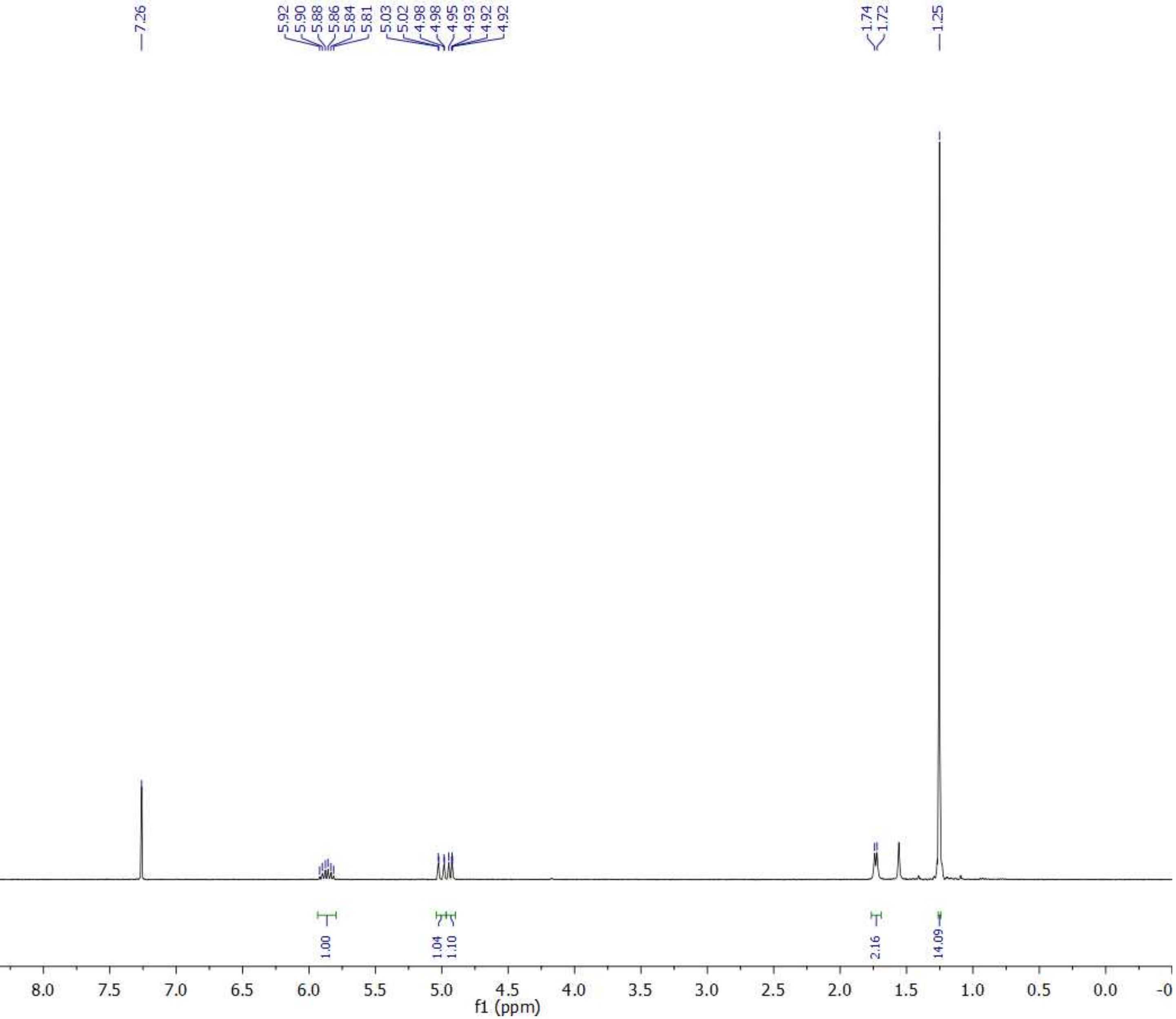

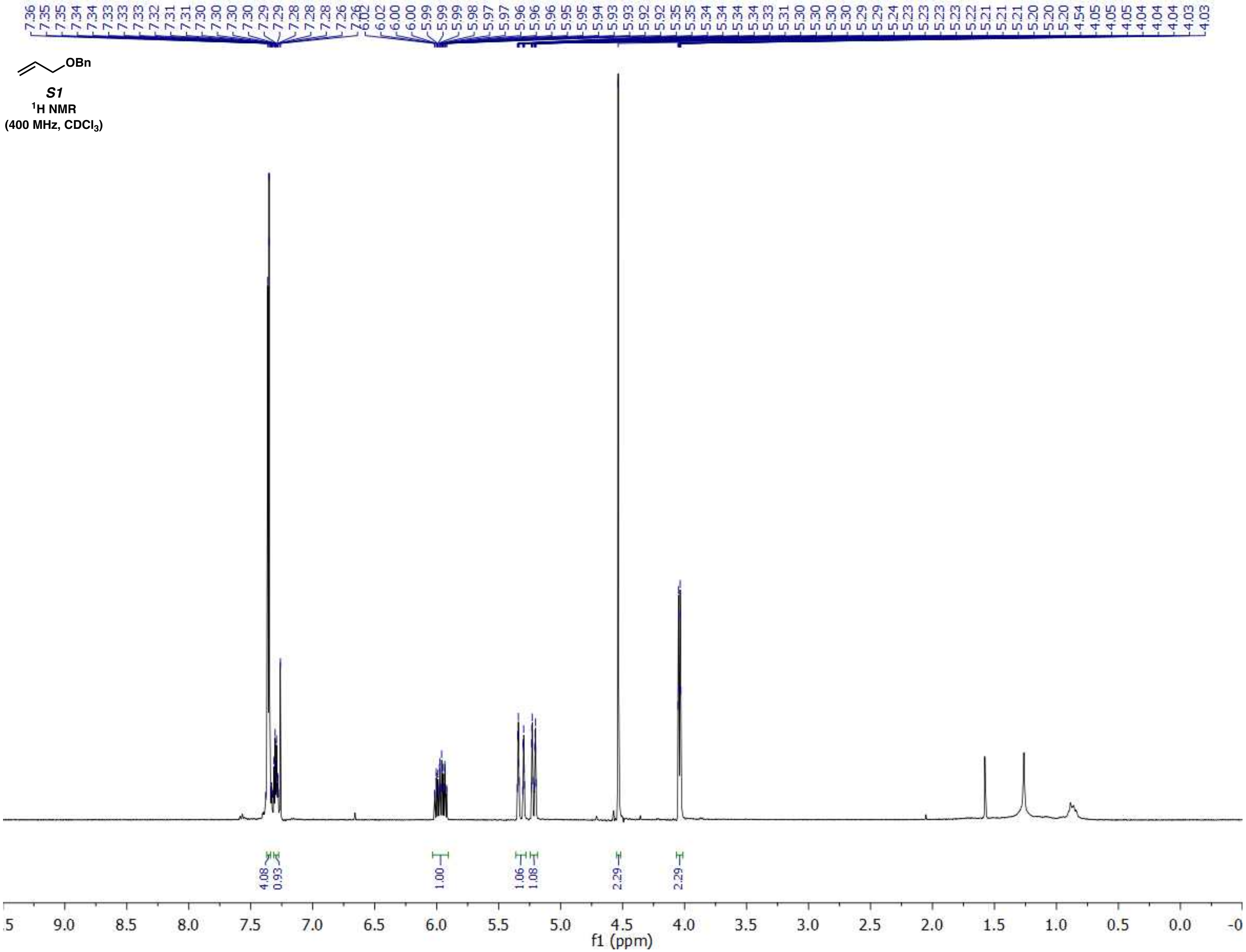
OBn
S1
1H NMR
(400 MHz, CDCl3)
7.36 7.35 7.35 7.34 7.34 7.33 7.33 7.33 7.32 7.31 7.31 7.30 7.30 7.30 7.29 7.29 7.28 7.28 7.26 7.26 6.02 6.02 6.00 5.99 5.99 5.99 5.98 5.97 5.97 5.96 5.96 5.96 5.95 5.95 5.94 5.93 5.93 5.92 5.92 5.35 5.35 5.34 5.34 5.34 5.34 5.33 5.31 5.30 5.30 5.30 5.30 5.29 5.29 5.24 5.23 5.23 5.23 5.23 5.22 5.21 5.21 5.21 5.20 5.20 5.20 4.54 4.05 4.05 4.05 4.04 4.04 4.04 4.03 4.03
4.08 0.93 1.00 1.06 1.08 2.29 2.29
9.0 8.5 8.0 7.5 7.0 6.5 6.0 5.5 5.0 4.5 4.0 3.5 3.0 2.5 2.0 1.5 1.0 0.5 0.0
f1 (ppm)
